# Cancer Curriculum for Appalachian Kentucky Middle and High Schools

**DOI:** 10.13023/jah.0301.05

**Published:** 2021-01-24

**Authors:** Lauren Hudson, Katherine Sharp, Chris Prichard, Melinda Ickes, Sahar Alameh, Nathan L. Vanderford

**Affiliations:** Markey Cancer Center, University of Kentucky; Department of STEM Education, College of Education, University of Kentucky; Markey Cancer Center, University of Kentucky; Department of Kinesiology and Health Promotion, College of Education, University of Kentucky; Department of STEM Education, College of Education, University of Kentucky; Markey Cancer Center, University of Kentucky, Department of Toxicology and Cancer Biology, College of Medicine, University of Kentucky

**Keywords:** Appalachia, cancer, school curriculum, middle school, high school, students, teachers

## Abstract

**Background:**

Appalachian Kentucky faces the highest cancer incidence and mortality rates in the country due to poor health behaviors and lifestyle choices. These poor health behaviors are facilitated by a lack of cancer education. Youth represent a vulnerable population that could be greatly impacted by increased cancer education. Teachers have the power to facilitate this learning.

**Purpose:**

This study examined the need for cancer education curriculum in Appalachian Kentucky middle and high schools from the perspective of educators.

**Methods:**

An online survey was conducted with science and health teachers (n=21) in Appalachian Kentucky, consisting of questions that investigated existing cancer education efforts, relevance of cancer education, and feasibility of such curriculum being delivered in the classroom. Content analysis was used to analyze teacher comments. A 3-part cancer education curriculum was developed that is culturally relevant and aligned with science and health education standards.

**Results:**

All participating teachers agree that cancer education is important to students’ lives. Teachers also agree that there is an inconsistent amount of cancer education within schools, and qualitative content analysis revealed that cancer education likely fits best in certain course subjects. Cancer education could feasibly be integrated into science and health classrooms, although the perception of needing to teach to the academic standards and having limited time to teach additional lessons outside of the standards are significant barriers. To combat this, a cancer curriculum that aligns with state and national science and health education standards was developed.

**Implications:**

Cancer education curriculum could play an important role in improving the cancer outlook in Appalachian Kentucky. Teachers have expressed a desire for increased cancer education in the classroom. By disseminating and implementing cancer curriculum in schools in the region and revising the curriculum-based on teacher and student feedback to better fit their needs, it has the potential to increase cancer literacy and improve related health behaviors and outcomes.

## INTRODUCTION

The U.S. has the fifth highest number of new cancer diagnoses in the world, with 352 out of every 100,000 citizens receiving a diagnosis. The U.S. also has the 6th highest number of diagnoses for men and the 8th highest for women at 393 cases and 321 cases per every 100,000 people, respectively.^1^ Certain areas of the U.S. observe a disproportionate percentage of cancer incidence and mortality rates, with Kentucky experiencing the highest statistics. Kentucky sees 181.6 cancer-related deaths per 100,000 total population, resulting in a total of 10,135 mortalities each year. The state also sees 510 new cancer cases per 100,00 citizens each year, which is 75 cases higher than the national average.^2^

The eastern, Appalachian region of Kentucky experiences disproportionate cancer rates.^3^ The cancer mortality rate in Appalachian Kentucky is 31% higher than the national average and 15% higher than the average in non-Appalachian counties in Kentucky.^3^ Many factors contribute to this disparity in Appalachia, including tobacco use, poor diet, sedentary lifestyle, and excessive sun exposure.^4^,^5^ Low education levels further these cancer-causing behaviors, as Appalachian citizens are not always aware that behaviors such as these increase cancer risk.^3^ Kentucky ranks 47th in the country for the percentage of adults who attended college,^6^ and nearly 1 in 3 adults have insufficient health literacy.^7^ Studies have shown that increased health literacy can improve health behaviors.^8^,^9^

Cancer literacy is defined as an individual’s ability to understand the advice of cancer healthcare professionals and make appropriate decisions related to cancer prevention, diagnosis, and treatment.^10^ Previous studies have been conducted showing that brief, one-time educational interventions can significantly improve the short-term and long-term cancer literacy in Appalachian Kentucky middle and high school students. In two studies, cancer professionals traveled to eleven schools in central and eastern Kentucky to implement a short presentation on cancer biology, disparities, and risk factors with emphasis on information specific to Appalachian Kentucky. Immediate short-term results revealed significant increases in the number of correct responses on a 10-item cancer literacy measure, and the number of correct responses remained significantly elevated at 3 months post-intervention compared to initial pretest scores.^11^,^12^ An increase in the number of correct responses suggests an increase in cancer knowledge. Students were also very likely to encourage a friend or family member to change their habits after attending the intervention.^12^ These pilot findings reinforce the notion that incorporating cancer education curriculum into schools can lead to further long-term increases in cancer literacy rates and promote healthy behaviors. The findings from these previous studies^12^, ^13^ and data reported herein led to the development of a cancer curriculum to educate students on cancer biology, disparities, and treatments. (Please note that “curriculum” is used throughout the text for both singular and plural mentions.)

Appalachian Kentucky youth represent a target population for this curriculum because they are at an age where they are forming habits that can impact their cancer risk (i.e., tobacco use). Despite the importance of impacting youth in this way, there have been very few attempts at incorporating long-term cancer education into curriculum. Existing studies have not targeted middle and high school students, and instead are targeting elementary school students or adults.^13^,^14^ Additionally, most existing curriculum has been created for nationwide dispersion,^15^,^16^ while culturally tailored curriculum may benefit those living in most at-risk areas. Curriculum that does target a specific population is typically short (1–2 hours in length) and not created for long-term, systematic integration into existing curriculum and not tailored to required educational standards.^17^

This study examines the need for and development of cancer education curriculum in Appalachian Kentucky middle and high schools. Based on teacher feedback, a culturally relevant and state and national science and health education standard–responsive cancer education curriculum was created that can be disseminated to schools within the Appalachian region of Kentucky. Such curriculum could increase students’ cancer literacy, and eventually decrease the cancer incidence and mortality rates in the Appalachian region. Using the following methods, responses from Appalachian Kentucky middle and high school teachers guided the development of culturally tailored cancer education curriculum.

## METHODS

A 6-question online survey (in Research Electronic Data Capture [REDCap]) was distributed to a group of science and health teachers in Appalachian Kentucky schools that had previously participated in studies measuring the impact of a brief cancer education intervention on middle and high school students.^11^,^12^ The survey consisted of three yes/no and three short answer questions that investigated existing cancer education efforts, relevance of cancer education, and feasibility of such curriculum being delivered in the classroom. Convenience sampling was used to recruit teachers (n=21) from 12 schools located in eastern, Appalachian Kentucky who taught chemistry, biology, anatomy, and/or health courses. Participants responded to the survey at their leisure. The participants and their schools were anonymized, but general demographics of the participants including gender, race, ethnicity, and grade level taught were collected (Appendix 1, see Additional Files). Frequency distributions were used to summarize the yes/no questions and all the short answer responses were reported as received with minor grammatical editing. Qualitative content analysis was used to categorize the participant responses and analyze themes present in the data. To complete the analysis, researchers manually reviewed teachers’ responses several times and divided them into themes based on the comments. Modifications were made as needed following initial thematization.

The study was conducted as part of an approved University of Kentucky Institutional Review Board Protocol (#44637). Participants consented through their engagement with the online survey.

Following data collection, a 3-part culturally relevant cancer education curriculum was developed for Appalachian Kentucky middle and high school students. This curriculum includes three sets of PowerPoint presentations, teacher guides, and pre/post quizzes. The topics covered in the curriculum were decided with Kentucky’s cancer disparities and risk factors in mind and based on the content of the initial cancer literacy intervention, which was proven to improve cancer-related knowledge.^11^,^12^ It was reviewed by healthcare professionals in the Markey Cancer Center and education professionals at the College of Education at the University of Kentucky. This curriculum was adapted to fit both the Kentucky Academic Standards for Science and national Next Generation Science Standards^18^ as well as the Kentucky Academic Standards for Health Education.^19^ The presentations, teacher’s guides, and evaluation questions are available as Appendix material (see Additional Files).

## RESULTS

### Participant Characteristics

Participating teachers (n=21) were 81% female and 19% male. There was a wide range of grades taught from 12 schools, with the most teachers educating 10^th^ grade students (38.1%). Five (23.8%) teachers taught 9^th^ grade, while 5 (23.8%) additional teachers taught 11^th^ grade and 1 (4.8%) teacher taught 12^th^ grade. Only 1 (4.8%) teacher taught 6^th^ grade. No 7^th^ grade teacher responded to the survey. All teachers (100%) identified as white and not of Hispanic or Latino origin (Appendix 1; see Additional Files). The teacher demographics align with the distribution of male and female teachers in Kentucky.^20^ Although demographics of the students of these teachers were not collected, the assumption is that they relatively align with the demographics of Kentucky, which is >85% white and non-Hispanic.^21^

### Teacher Responses

Teachers were asked three preliminary questions regarding existing cancer education in their schools. Thirty-three percent (33.7%) of teachers said that their school does not do anything to incorporate cancer education into the curriculum, and 66.7% of teachers said their school does take measures to incorporate cancer education into the curriculum. All (100%) the teachers believed that cancer education in schools would be beneficial, and 95.2% of teachers believed it would be feasible to incorporate cancer topics in the curriculum within their classroom (Table 1). Only 1 teacher (4.8%) noted that there was not sufficient time in her 11th grade chemistry classroom to incorporate cancer education topics into curriculum.

Teachers were also asked to provide short answers about existing efforts to incorporate cancer education into their curriculum (Table 2; see Additional Files). Content analysis of these responses revealed four main themes: medicine/health, biology, chemistry, and no existing curriculum/subject not specified. Teachers noted that biology courses touched on cancer mechanisms during chapters on the cell cycle, and chemistry courses discussed radiation and nuclear chemistry with respect to cancer development and treatment. Health and anatomy teachers noted that related topics are covered in biomedical courses. However, many teachers of all grade levels similarly said that these teachings were not in-depth. Some cancer education topics were only available for students in special programs such as Project Lead the Way,^22^ a hands-on program for students who wish to specialize in STEM, while others were presented to students in advanced courses.

Finally, teachers were asked to provide short answers that discussed whether or not cancer education would be beneficial in their schools (Table 3; see Additional Files). Content analysis of these responses revealed three main reasons that cancer education would be beneficial: prevention, cancer prevalence, and general awareness. These themes were similar in all participants’ responses, regardless of grade level or subject taught. First, the majority of teachers mentioned that cancer is extremely prevalent in their community, impacting most, if not all, their students’ lives. The widespread prevalence of cancer in Appalachian Kentucky makes it a very important topic of instruction. Second, teachers recognize the importance of cancer prevention. They know that certain cancer risk factors include modifiable behaviors and believe that curriculum may encourage students to make healthy lifestyle choices. Lastly, teachers agree that students could generally benefit from increased awareness and note that real-world application will engage students and may spark interest in a previously unfamiliar field.

Content analysis of the final question, which asked if teachers believed incorporating cancer topics into the curriculum would be feasible (Table 4; see Additional Files), revealed five topics: implementation of the curriculum in medicine/health, biology, and chemistry, curriculum implementation dependent on materials provided, and already implemented. One significant barrier to cancer education was instruction time in the classroom. Some teachers found it difficult to teach all of the state and national science standards within the school year and worried there would be no additional time for topics outside of those standards, such as cancer education. Additionally, very few teachers mentioned that their curriculum incorporated education on cancer risk factors, and none mentioned whether or not this curriculum was culturally tailored or aligned with science and health education standards.

### Curriculum Development

In response to these data, a 3-part cancer education curriculum was developed to help teachers educate students on cancer biology, disparities, and treatments in Kentucky. This curriculum includes three lessons with corresponding PowerPoint presentations. The first lesson discusses cancer biology and disparities especially those relevant to Appalachian Kentucky; the second focuses on cancer risk factors in Kentucky, and the third teaches about cancer treatment. Lesson three is offered in three versions: a full length, in-depth version; the full-length version split into two sessions; and a shorter, abbreviated version. Different versions of lesson three were created to ensure that the lesson, which is much longer than the first two, is accessible for all classrooms regardless of time constraints. The lessons are available as appendix material (Appendixes 2–7; see Additional Files). Teacher guides or lesson plans were developed for each PowerPoint to better equip teachers to present this material to their students (Appendixes 8–13; see Additional Files). Each lesson plan contains a 10-question pre- and post-test survey to evaluate students’ cancer literacy before and after the delivery of the curriculum.

This curriculum was adapted to fit the Kentucky Academic Standards for Science and national Next Generation Science Standards as well as the Kentucky Academic Standards for Health Education. The curriculum fulfills the standards listed in Appendix 14 (see Additional Files). Because teachers noted that instruction time due to state and national science standards is a barrier to incorporating cancer curriculum, it was essential to make this curriculum align closely with these standards while still providing students with necessary cancer-related healthcare information. Additionally, because teachers noted that students are more engaged when the material is relevant to their lives, all curriculum was developed with Appalachian community cancer disparities in mind. This includes a tailored curriculum discussing Appalachia’s high cancer rates and specific cancer-causing risk factors. The curriculum acts as a starting point for instruction on cancer and its impact in Appalachian Kentucky. Future evaluation of both teachers and students will be used to update and adapt the curriculum.

## IMPLICATIONS

This study presents Kentucky science and health teachers’ perspectives on the need for cancer education curriculum in Appalachian Kentucky middle and high schools. The responses show that all teachers agreed that cancer education would be beneficial for students, and most teachers agreed that the education would be feasible in a classroom setting. Content analysis shows that while some teachers already attempt to incorporate cancer education in the classroom, not all students in the school are educated and information provided may be limited.

As discussed above, teachers’ responses were used to assist in the development of an Appalachian Kentucky–specific cancer education curriculum. Two factors must be considered: (1) state and national standards and (2) cultural influences.

Because teachers are required to teach the topics listed on the state and national education standards, there could be a perception that there simply is not enough time in the classroom to fit additional material, such as cancer education. By aligning with these standards and providing real-world examples related to cancer, students are receiving practical, useful teachings that have the potential to improve their health and the health of those around them. They are also receiving additional science education that can be realistically applied to their everyday lives. Additionally, very few teachers mentioned educating students about behavioral cancer risk factors, but they noted that certain cancer types can be prevented through lifestyle changes. Because Kentucky has so many specific risk factors (i.e., tobacco use, excessive sun exposure, healthcare neglect), it is necessary for cancer education curriculum to be centered around Kentucky’s cancer disparities. Nationwide cancer education campaigns will teach students about the basics of cancer biology and treatment, but do not educate students about how prevalent cancer is in Kentucky and what can be done to improve the incidence and mortality rates in the Appalachian region of the state in a way that is framed around Kentucky’s major risk factors. By also aligning the curriculum with health education standards and by presenting students with statistics and risk factors that heavily affect their community, students will be more likely to understand and retain this information when making lifestyle choices.^23^

The curriculum developed in this study is novel because it is ongoing and tailored to a specific population that is in a pivotal stage for developing life-long habits. Existing school cancer curriculum typically does not properly address these factors in specific cultural and regional populations. A 1996 study sought to determine the effectiveness of skin cancer prevention curriculum in 4th, 5th, and 6th grade students.^13^ A similar study incorporated general cancer education curriculum for elementary schoolers.^14^ The National Science Teaching Association created The Wonderkids Program, which teaches STEM and cancer-related topics to underserved elementary school students in the Los Angeles Unified School District.^24^ Although the former studies reported an increase in student knowledge following the curriculum, it is difficult to tell if this curriculum will truly influence students’ behavior because the students are so young at the time of implementation. The American Cancer Society pilot-tested cancer curriculum in secondary schools around the country.^15^,^16^ Although students and teachers reacted positively to the cancer curriculum, the curriculum was generalized for the entire U.S. population and not culturally tailored to a particular region. A study involving Hispanic adolescents examined the effectiveness of cervical cancer curriculum and saw a significant change in cancer knowledge.^18^ However, this curriculum only consisted of a 1-hour intervention. One school-based breast cancer curriculum created using resources from Susan G. Komen for the Cure was ongoing and culturally tailored. It sought to teach Latino high school students about breast cancer through 5 units. Despite the fact that it was pilot tested, this paper reports about the methods and strategies used within the curriculum but does not report how effective it is for improving cancer knowledge.^25^ In order to make an impactful change on the health outcomes of a community, cancer curriculum should be in-depth and broad, targeted towards an impressionable population regarding behavioral changes, and culturally tailored.^26^

This work should be carefully considered in the context of its limitations. One limitation is the sample size. Through the convenience sampling, only 21 teachers in Appalachian Kentucky completed the questionnaire, which means these results may not be reflective of all teachers in Appalachia or of teachers outside of Appalachia. Additionally, the questionnaire was given to different types of science teachers, including health, chemistry, anatomy, and biology teachers. Cancer education has the potential to be incorporated in different ways in each of these courses as discovered via content analysis, which provided heterogeneity in the responses. Some teachers incorporated cancer education into their curriculum in some level of depth, while others did not at all. Separating out teachers’ responses based on subject area taught is useful in deciding which classroom cancer education curriculum would fit best. Furthermore, teachers’ perceptions may have influenced their willingness to incorporate cancer education into their classrooms. They note that there is not enough time to teach outside of the standards, which may have made some unwilling to discuss incorporation of cancer education further. This can be combated by aligning the curriculum with state and national standards. Lastly, we have yet to investigate the effectiveness of this curriculum by measuring the short- and long-term impact on cancer literacy. Future research will examine students’ pre- and post-test scores to determine if it truly improves cancer literacy and promotes healthy behaviors. Within this future study, students’ and teachers’ feedback can be received to better facilitate program evaluation and further improve the curriculum for the population. In a similar future study, the curriculum can be evaluated based on its applicability to a diverse group of learners, such as the black and Latinx populations in Appalachian Kentucky. Despite the limitations and the need for future evaluation, cancer curriculum development and additional cancer literacy work has great potential to aid in addressing the cancer disparities in Appalachian Kentucky.

**SUMMARY BOX**

**What is already known about this topic?** Educating Appalachian youth about cancer-related topics could be a critical step in improving the cancer outlook in eastern Kentucky. Previous research shows that there have been very few attempts to provide middle and high school students with an ongoing, culturally tailored cancer education curriculum.

**What is added by this report?** This study surveyed science teachers in Appalachian Kentucky to determine the current cancer education efforts in schools, the relevance of cancer education in students’ lives, and the feasibility of long-term cancer education curriculum in the classroom. The results show that the current cancer education levels in schools vary, with some students not receiving any cancer-related instruction at all. Teachers believe that cancer education is essential to students’ lives, and many believe it would be possible to incorporate into classroom instruction, but available classroom time is a significant barrier. A 3-part cancer curriculum was developed that is culturally tailored and education standard–responsive.

**What are the implications for future research?** Future research should examine students’ pre- and post-test scores to determine if the curriculum truly increases students’ cancer literacy and influences healthy behaviors.

## Supplementary Information

































## Figures and Tables

**Table 1:** Cancer curriculum in schools

Variable	Yes % (n)	No % (n)
Do you or your school do anything to incorporate cancer education into the curriculum?	66.7 (14)	33.3 (7)
Do you believe cancer education in schools would be beneficial?	100 (21)	0
Do you believe incorporating cancer topics into the curriculum would be feasible within your classroom/school?	95.2 (20)	4.8 (1)
