# Supplementary material for: Cancer Curriculum for Appalachian Kentucky Middle and High Schools
Source: J Appalach Health. 2021 Jan 24;3(1):43–55. doi: 10.13023/jah.0301.05 (PMC8830599; doi:10.13023/jah.0301.05)
Supplement: Supplementary file 1 [file Appendix2-3.1.5Hudson.pptx]

## Slide 1
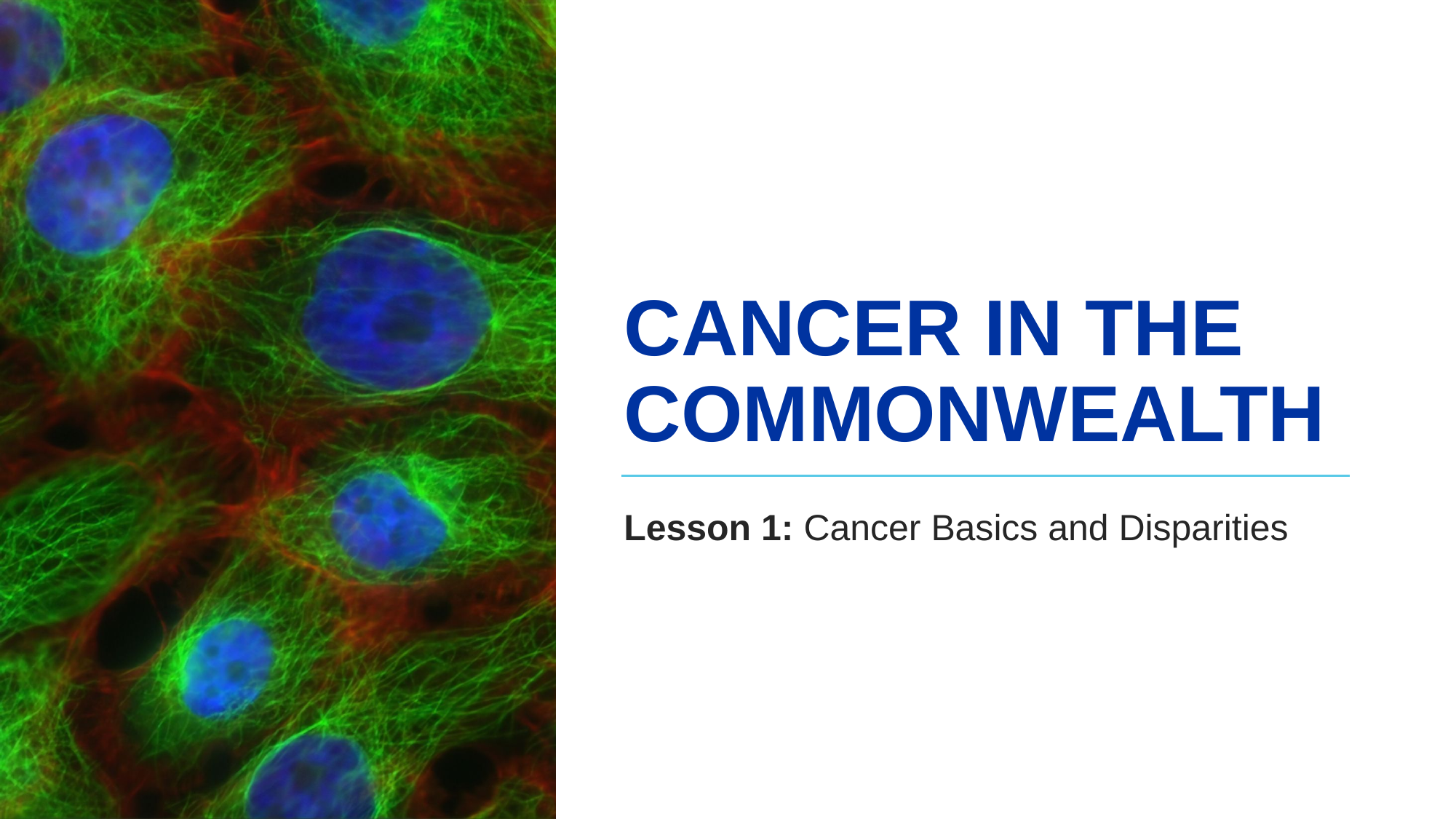

# CANCER IN THE COMMONWEALTH
Lesson 1: Cancer Basics and Disparities

## Slide 2
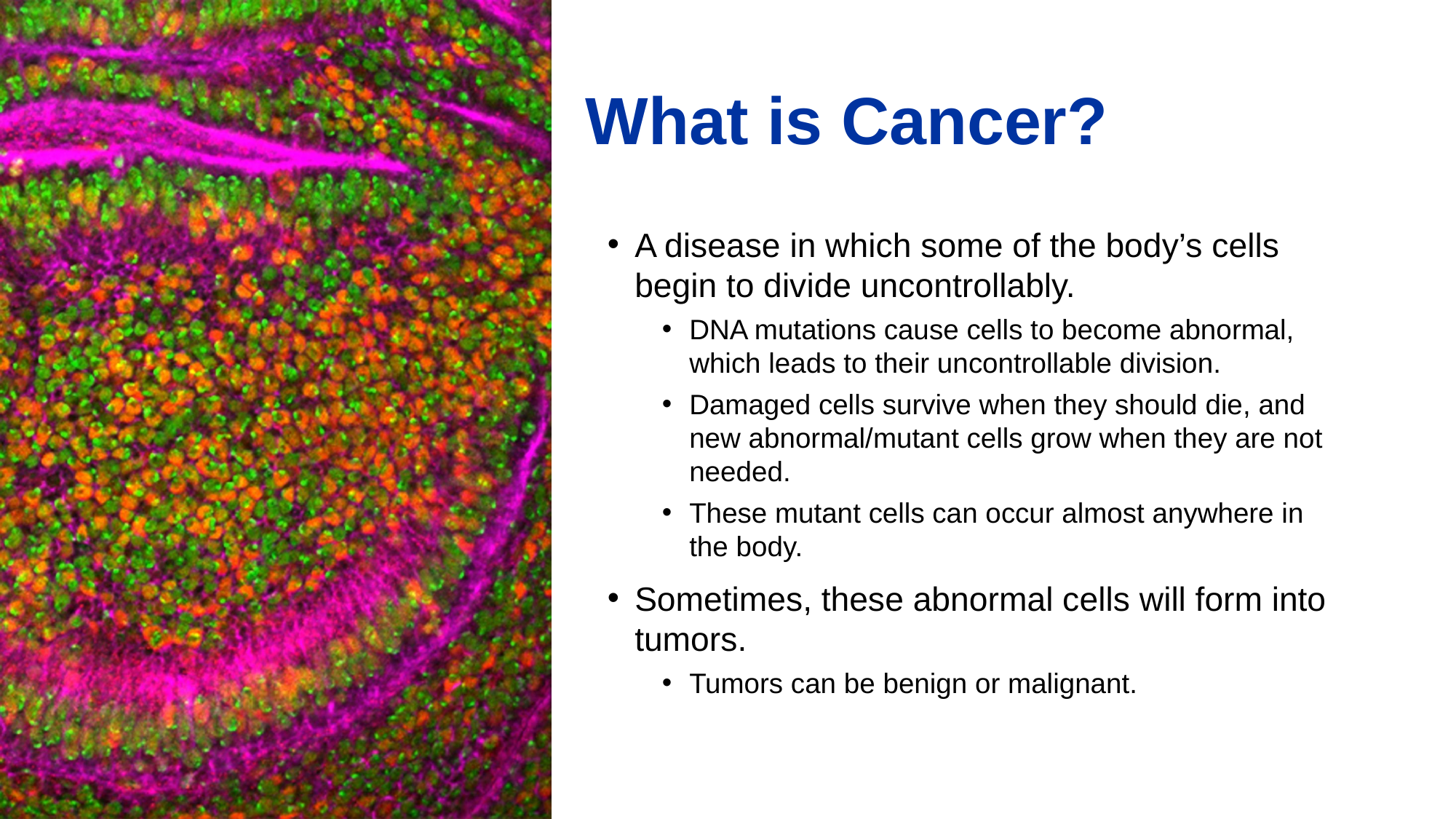

# What is Cancer?
A disease in which some of the body’s cells begin to divide uncontrollably.
DNA mutations cause cells to become abnormal, which leads to their uncontrollable division.
Damaged cells survive when they should die, and new abnormal/mutant cells grow when they are not needed.
These mutant cells can occur almost anywhere in the body.
Sometimes, these abnormal cells will form into tumors.
Tumors can be benign or malignant.

## Slide 3
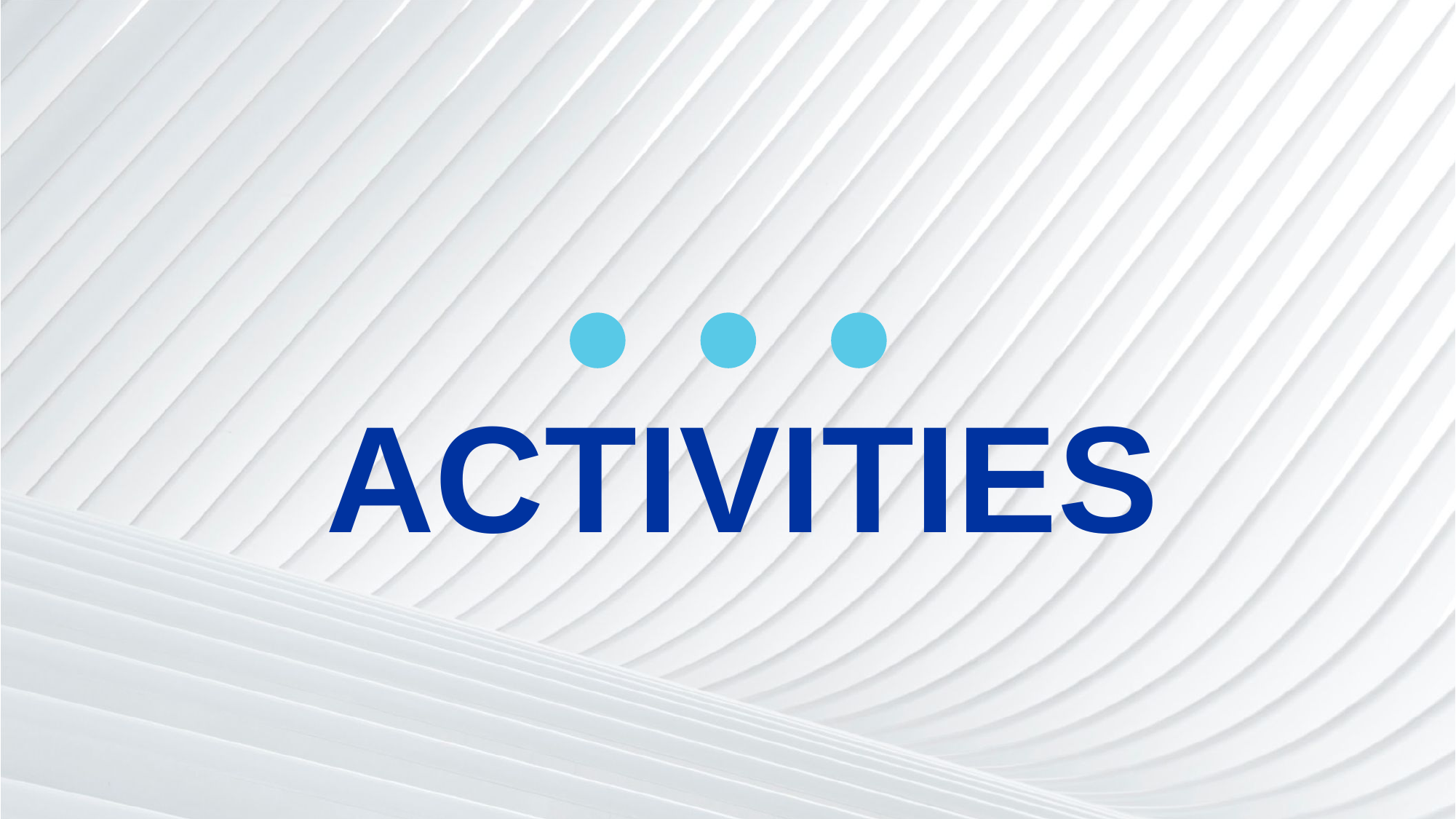

# ACTIVITIES

## Slide 4
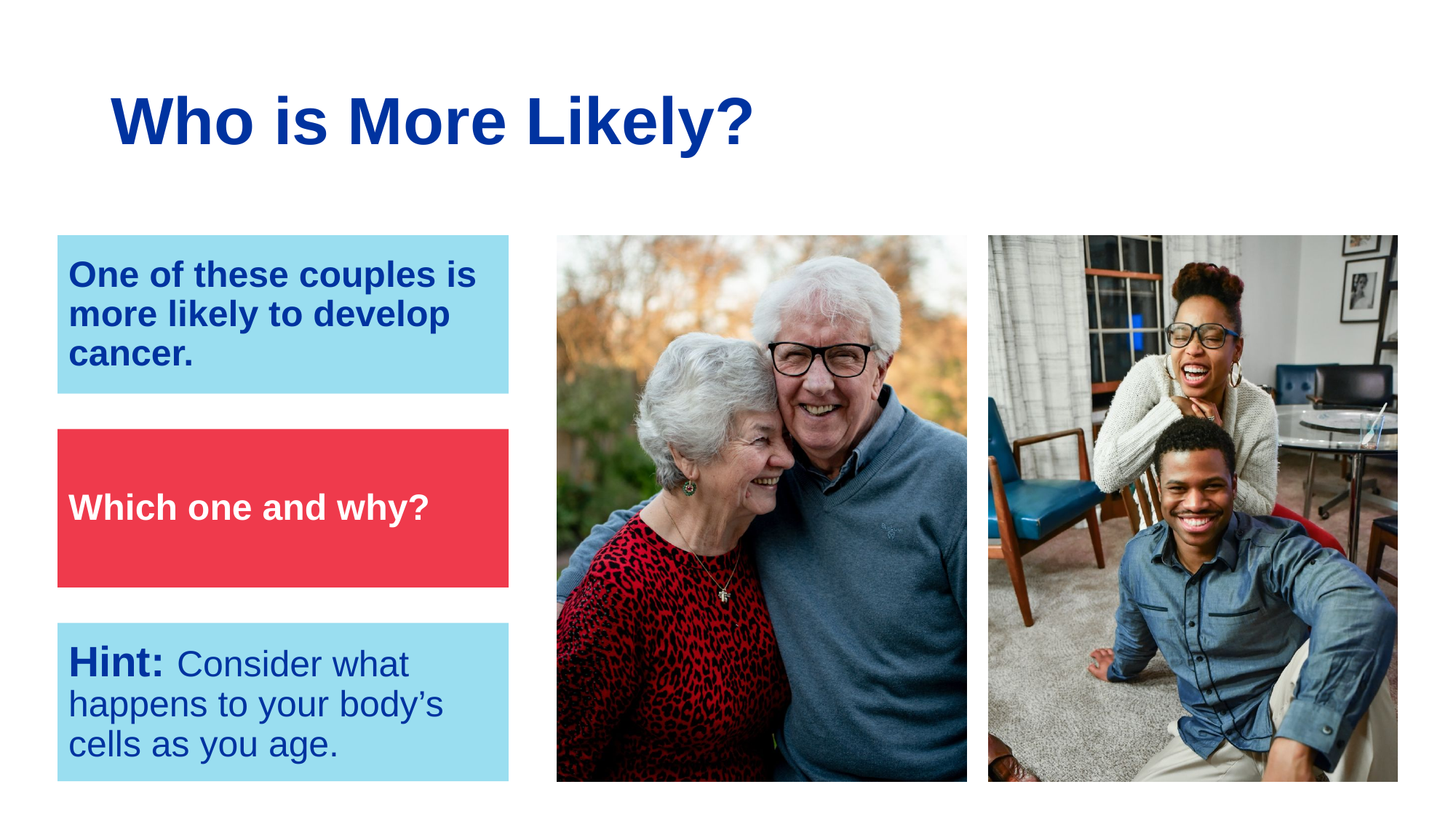

# Who is More Likely?
One of these couples is more likely to develop cancer.
Which one and why?
Hint: Consider what happens to your body’s cells as you age.

## Slide 5
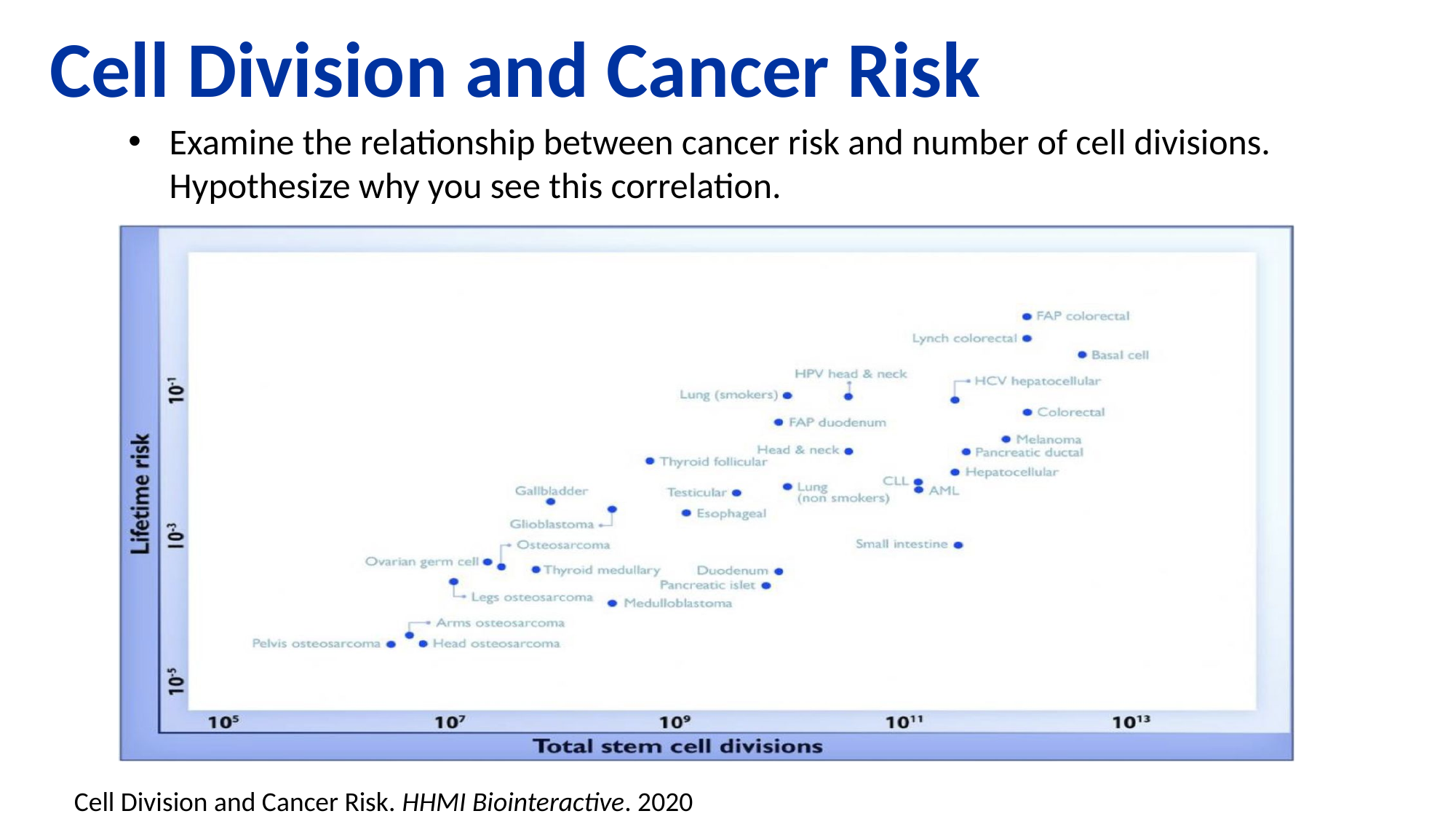

# Cell Division and Cancer Risk
Examine the relationship between cancer risk and number of cell divisions. Hypothesize why you see this correlation.
Cell Division and Cancer Risk. HHMI Biointeractive. 2020

## Slide 6
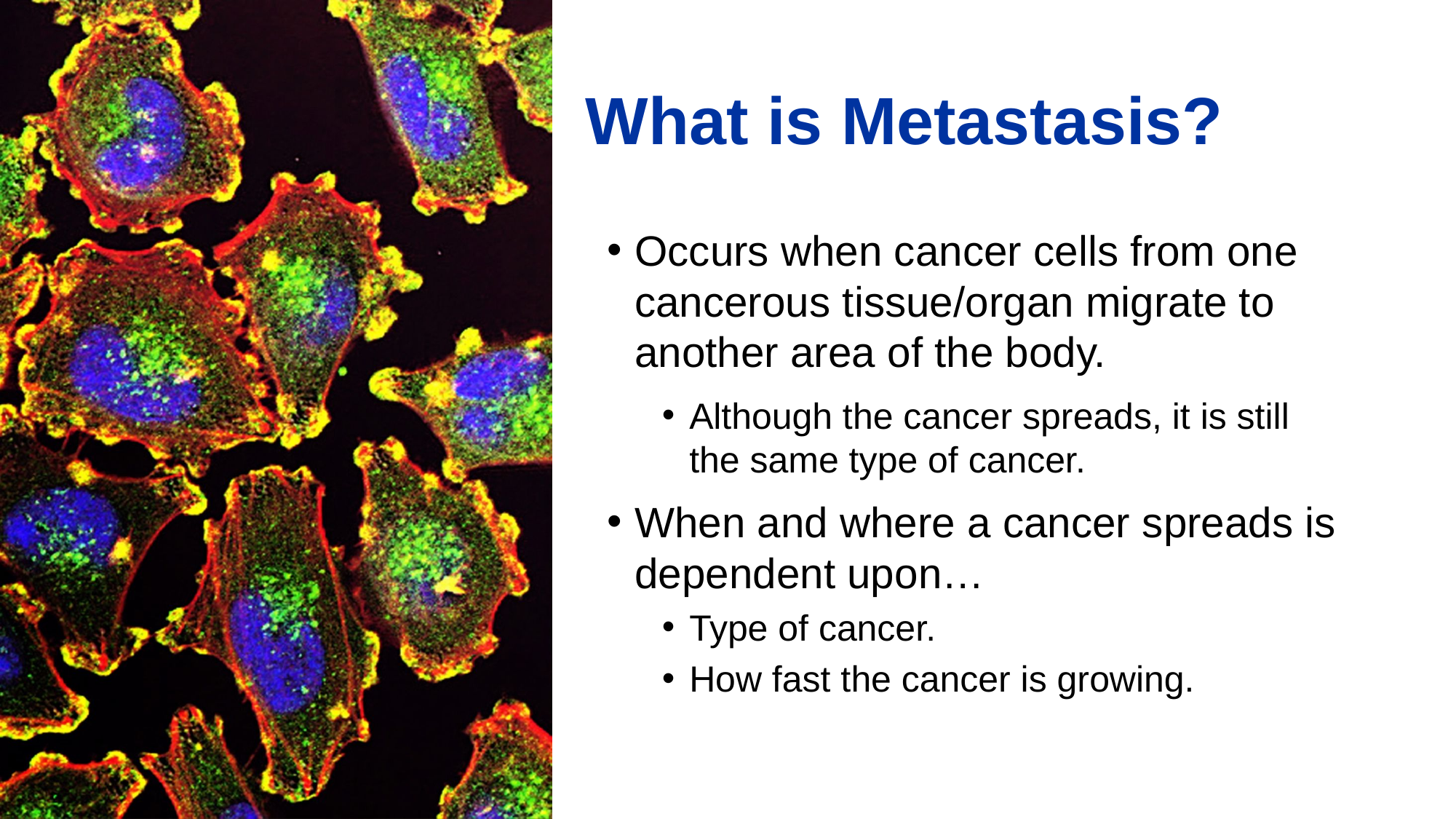

# What is Metastasis?
Occurs when cancer cells from one cancerous tissue/organ migrate to another area of the body.
Although the cancer spreads, it is still the same type of cancer.
When and where a cancer spreads is dependent upon…
Type of cancer.
How fast the cancer is growing.

## Slide 7
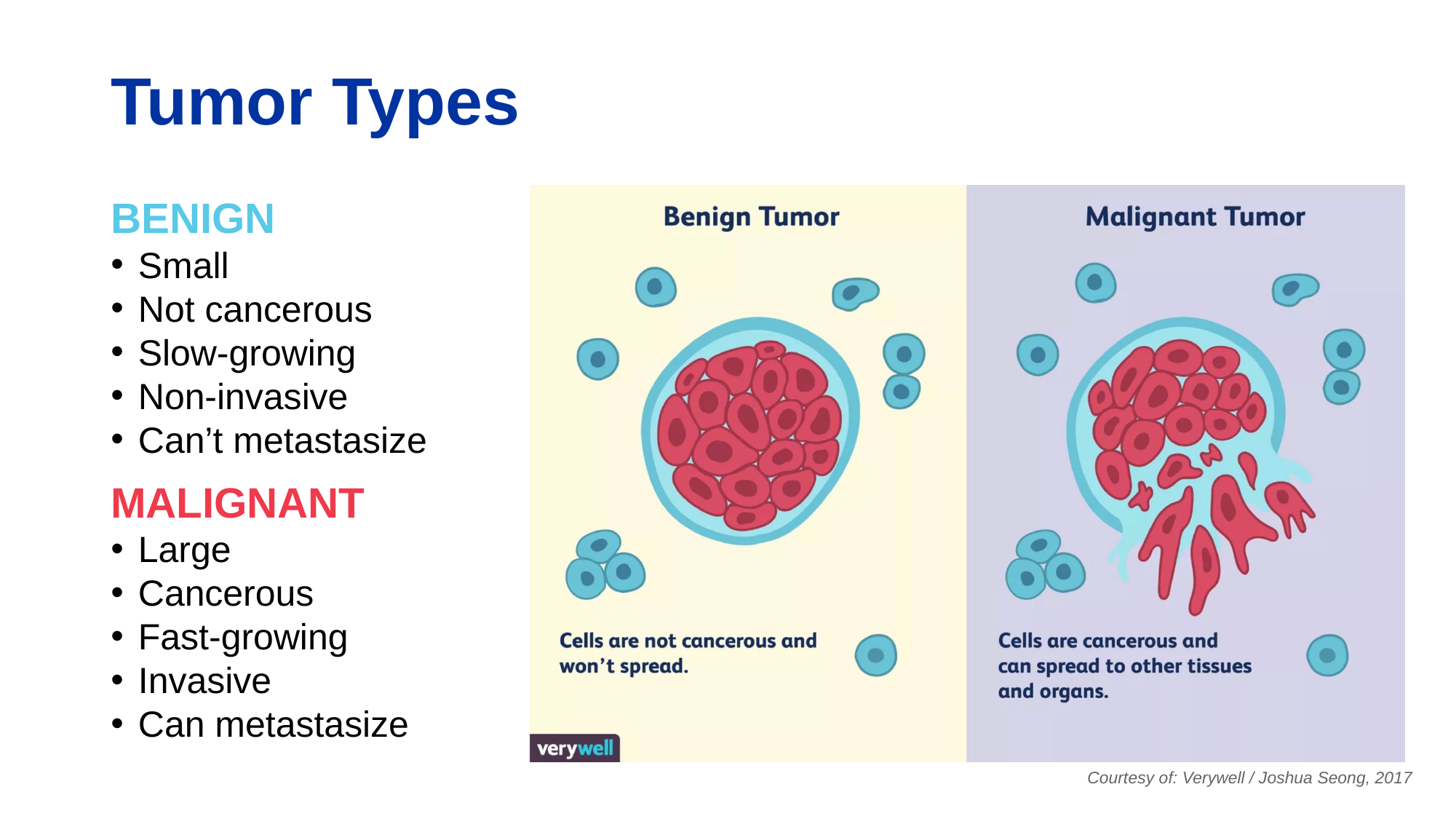

# Tumor Types
BENIGN
Small
Not cancerous
Slow-growing
Non-invasive
Can’t metastasize
MALIGNANT
Large
Cancerous
Fast-growing
Invasive
Can metastasize
Courtesy of: Verywell / Joshua Seong, 2017

## Slide 8
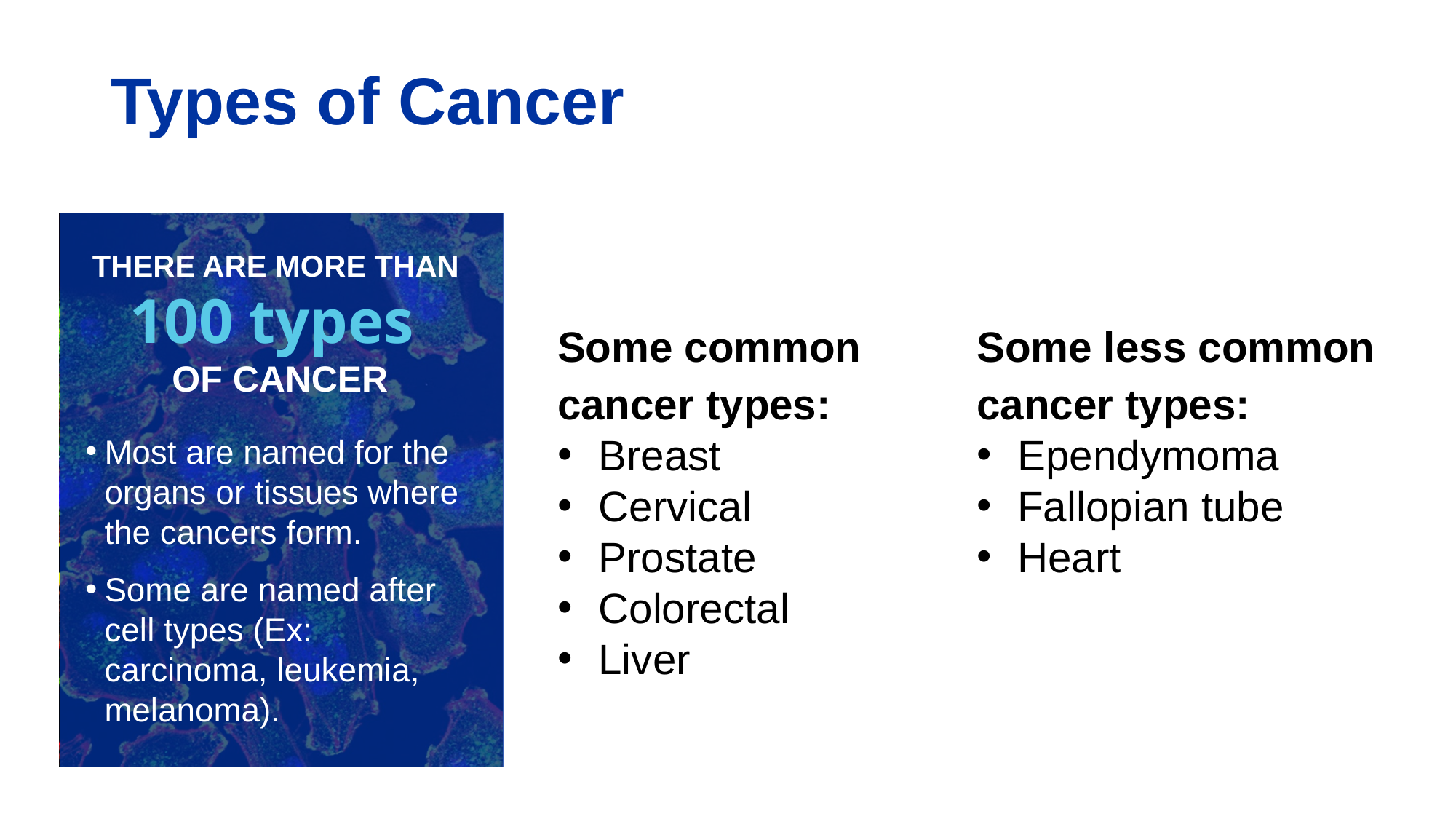

# Types of Cancer
THERE ARE MORE THAN
100 types
OF CANCER
Some common cancer types:
Breast
Cervical
Prostate
Colorectal
Liver
Some less common cancer types:
Ependymoma
Fallopian tube
Heart
Most are named for the organs or tissues where the cancers form.
Some are named after cell types (Ex: carcinoma, leukemia, melanoma).

## Slide 9
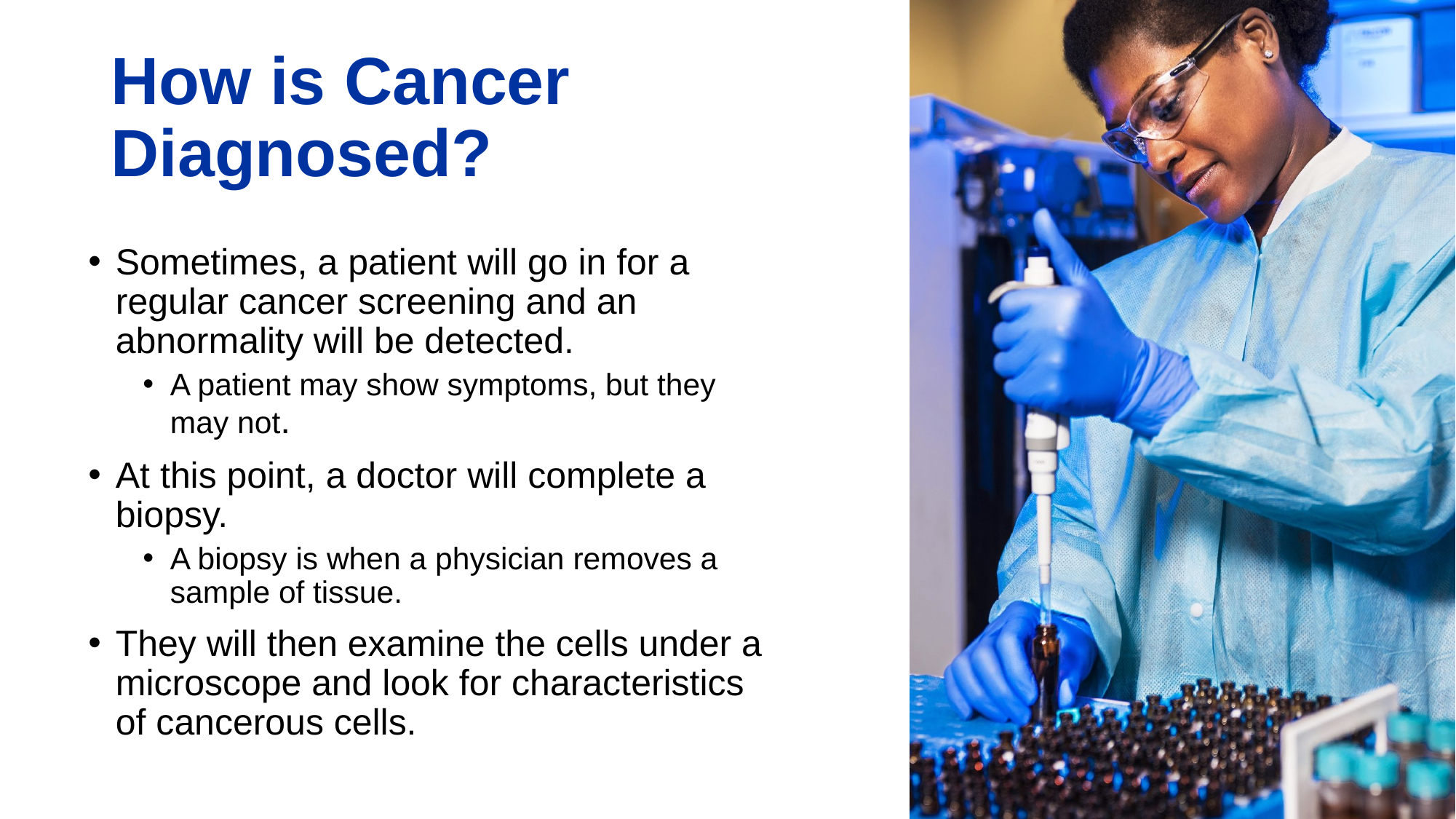

# How is Cancer Diagnosed?
Sometimes, a patient will go in for a regular cancer screening and an abnormality will be detected.
A patient may show symptoms, but they may not.
At this point, a doctor will complete a biopsy.
A biopsy is when a physician removes a sample of tissue.
They will then examine the cells under a microscope and look for characteristics of cancerous cells.

## Slide 10
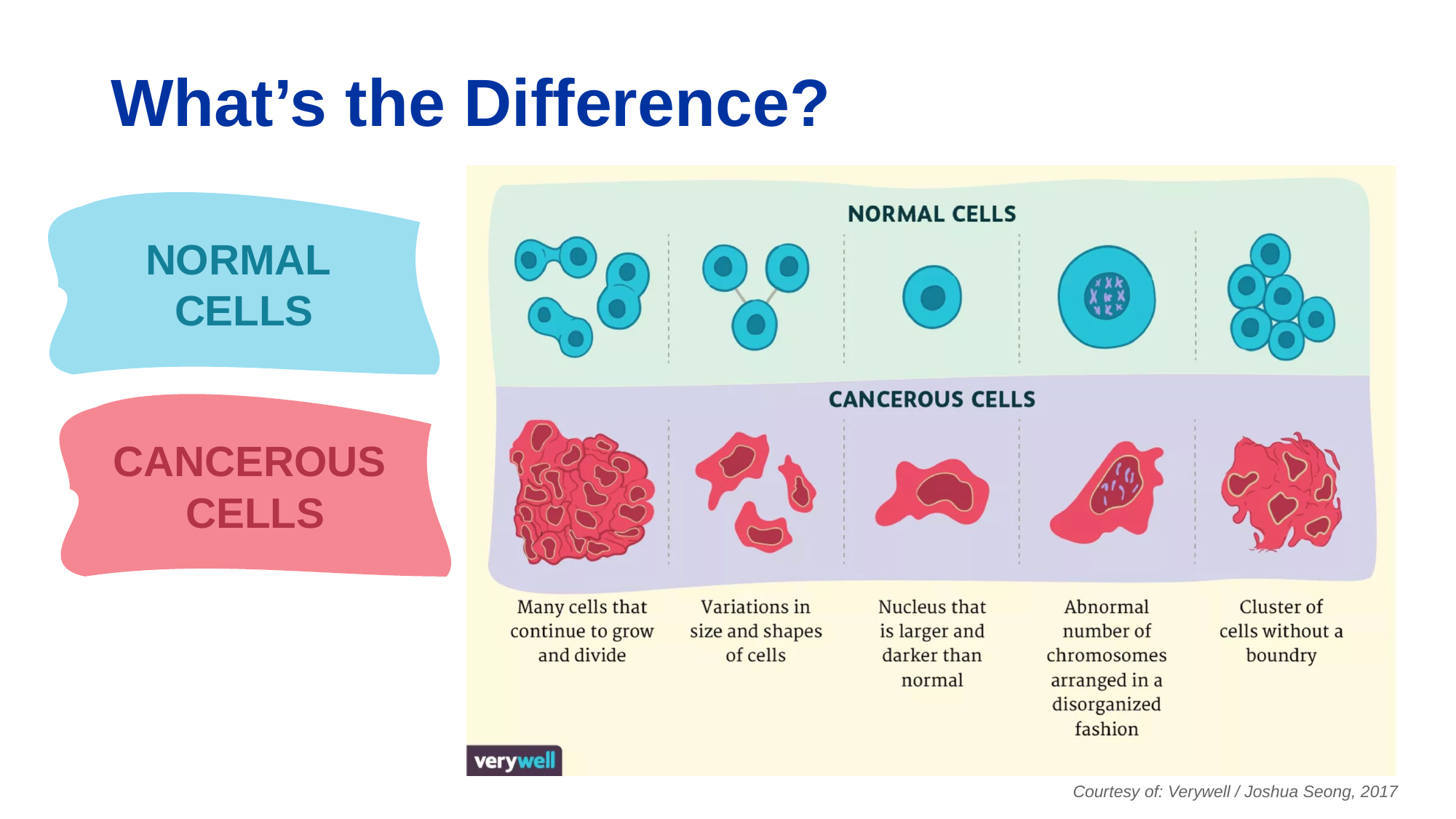

# What’s the Difference?
NORMAL
CELLS
CANCEROUS
CELLS
Courtesy of: Verywell / Joshua Seong, 2017

## Slide 11
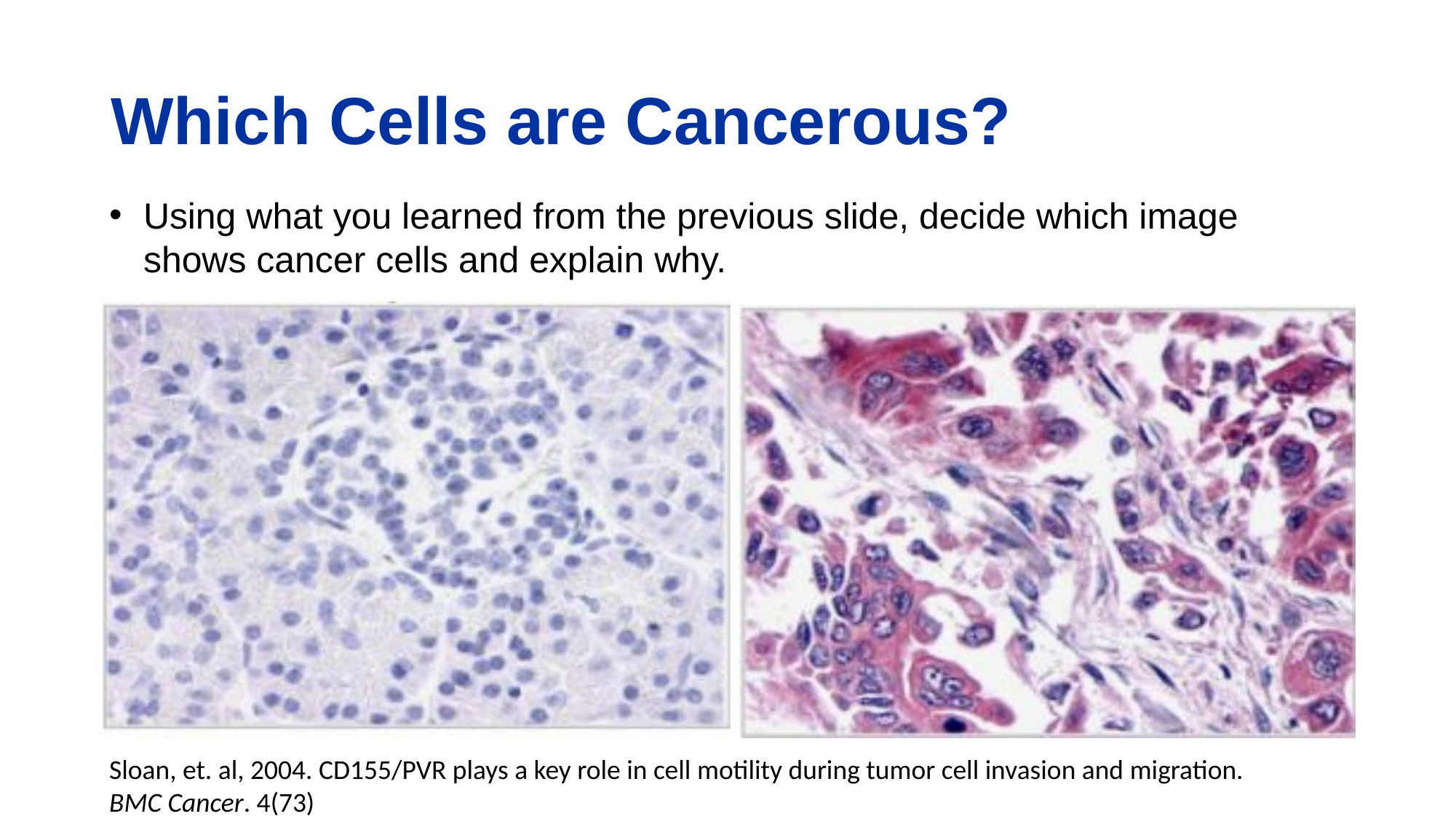

# Which Cells are Cancerous?
Using what you learned from the previous slide, decide which image shows cancer cells and explain why.
Sloan, et. al, 2004. CD155/PVR plays a key role in cell motility during tumor cell invasion and migration. BMC Cancer. 4(73)

## Slide 12
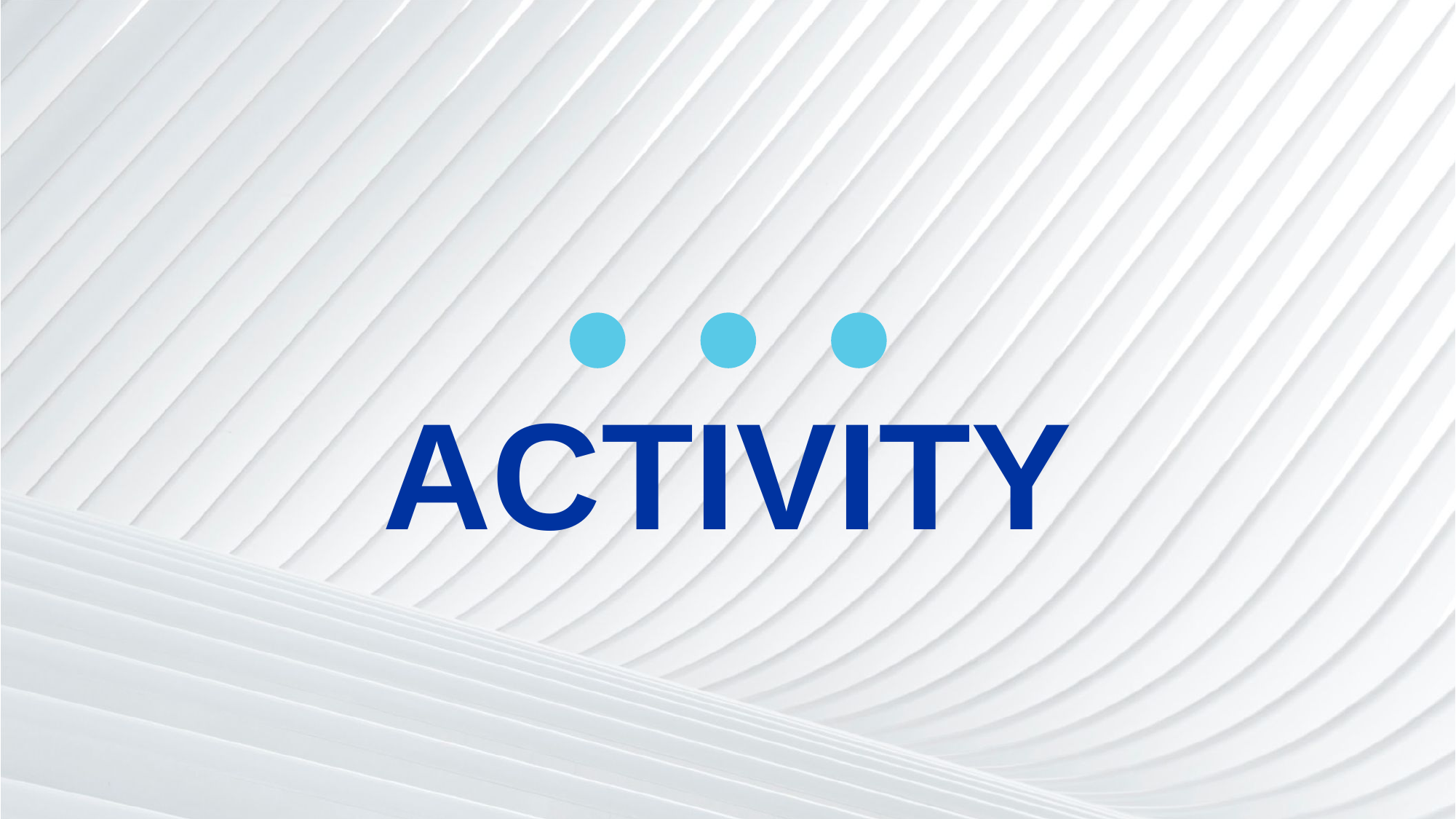

# ACTIVITY

## Slide 13
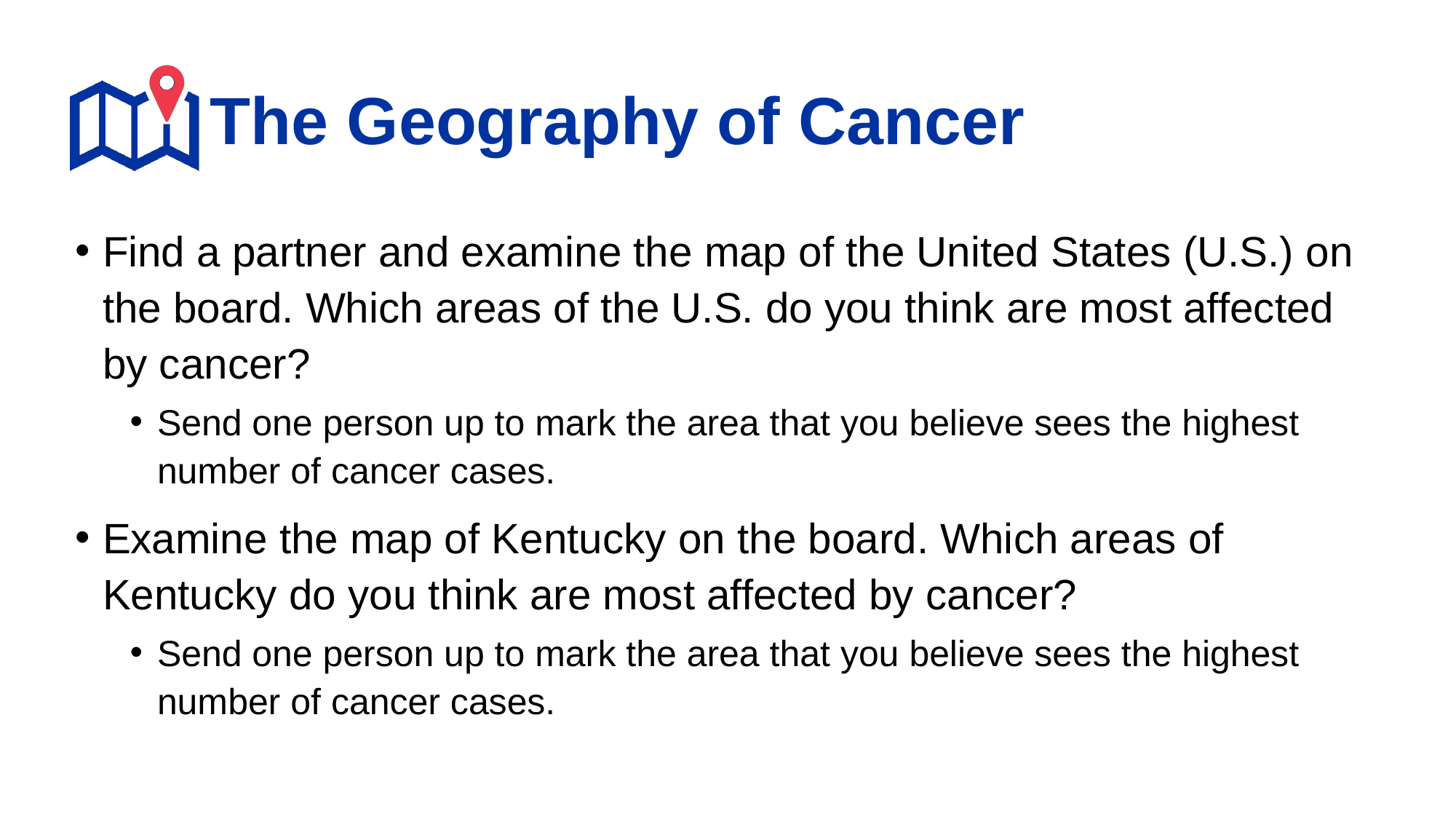

# The Geography of Cancer
Find a partner and examine the map of the United States (U.S.) on the board. Which areas of the U.S. do you think are most affected by cancer?
Send one person up to mark the area that you believe sees the highest number of cancer cases.
Examine the map of Kentucky on the board. Which areas of Kentucky do you think are most affected by cancer?
Send one person up to mark the area that you believe sees the highest number of cancer cases.

## Slide 14
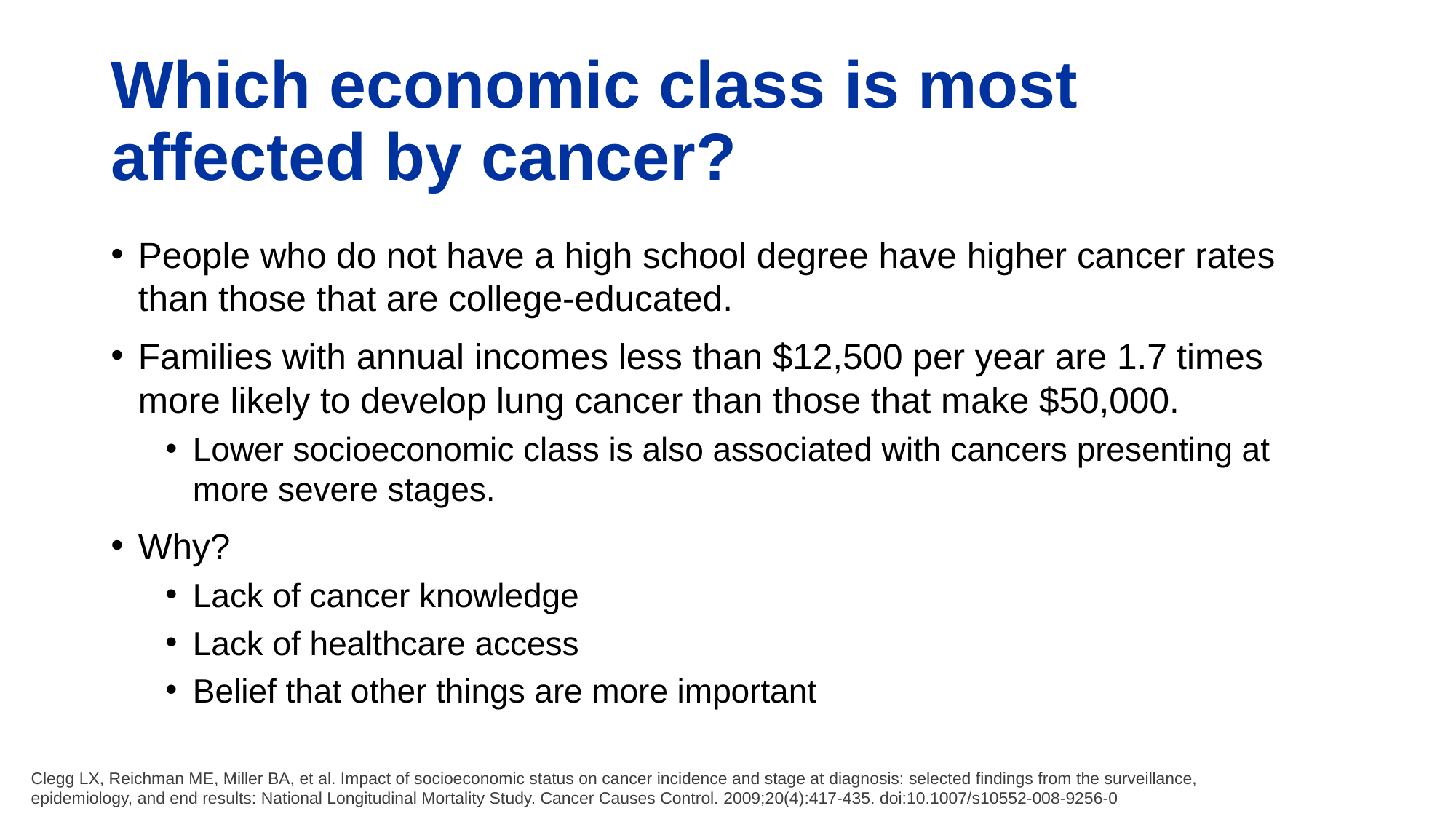

# Which economic class is most affected by cancer?
People who do not have a high school degree have higher cancer rates than those that are college-educated.
Families with annual incomes less than $12,500 per year are 1.7 times more likely to develop lung cancer than those that make $50,000.
Lower socioeconomic class is also associated with cancers presenting at more severe stages.
Why?
Lack of cancer knowledge
Lack of healthcare access
Belief that other things are more important
Clegg LX, Reichman ME, Miller BA, et al. Impact of socioeconomic status on cancer incidence and stage at diagnosis: selected findings from the surveillance, epidemiology, and end results: National Longitudinal Mortality Study. Cancer Causes Control. 2009;20(4):417-435. doi:10.1007/s10552-008-9256-0

## Slide 15
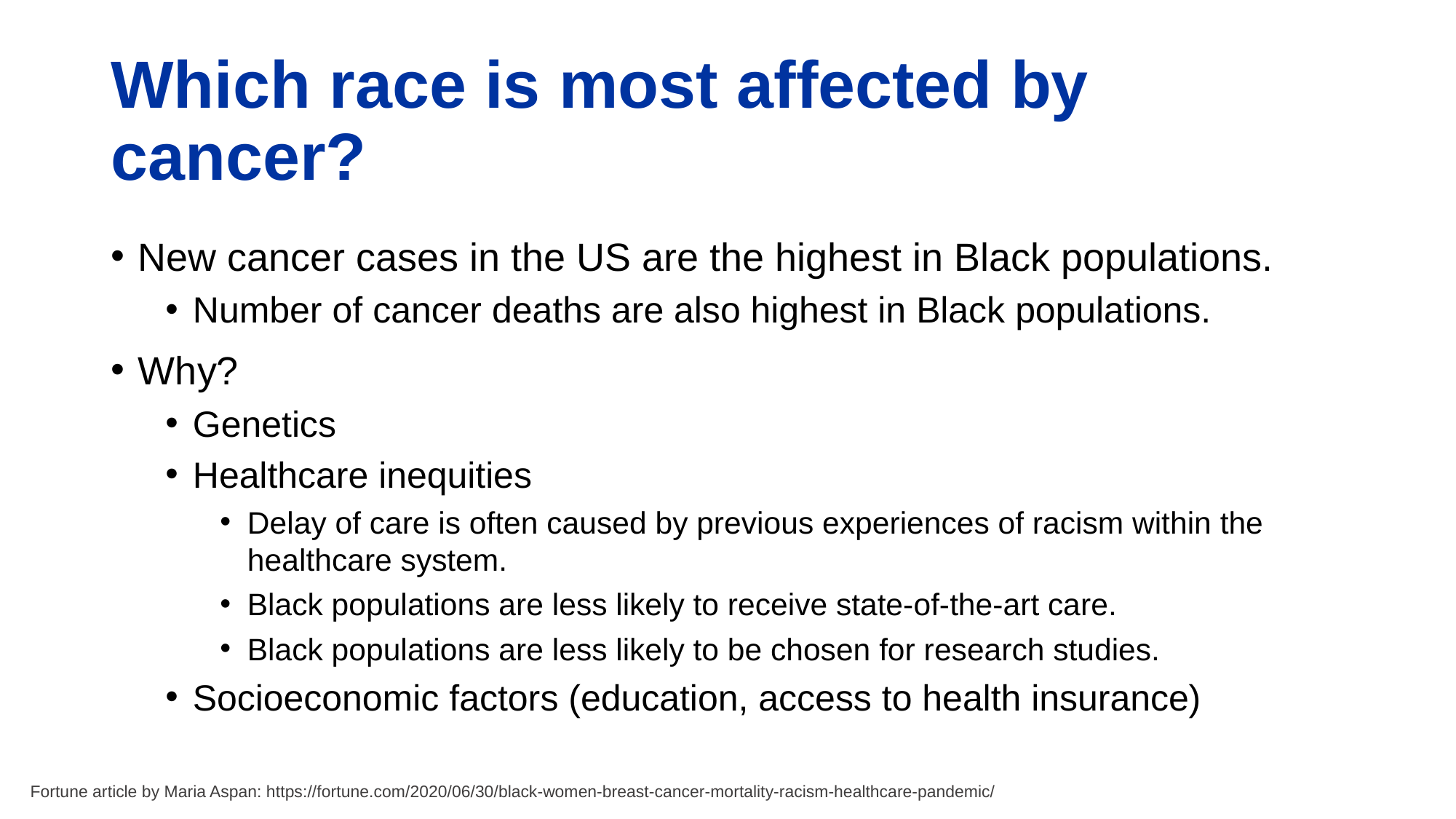

# Which race is most affected by cancer?
New cancer cases in the US are the highest in Black populations.
Number of cancer deaths are also highest in Black populations.
Why?
Genetics
Healthcare inequities
Delay of care is often caused by previous experiences of racism within the healthcare system.
Black populations are less likely to receive state-of-the-art care.
Black populations are less likely to be chosen for research studies.
Socioeconomic factors (education, access to health insurance)
Fortune article by Maria Aspan: https://fortune.com/2020/06/30/black-women-breast-cancer-mortality-racism-healthcare-pandemic/

## Slide 16
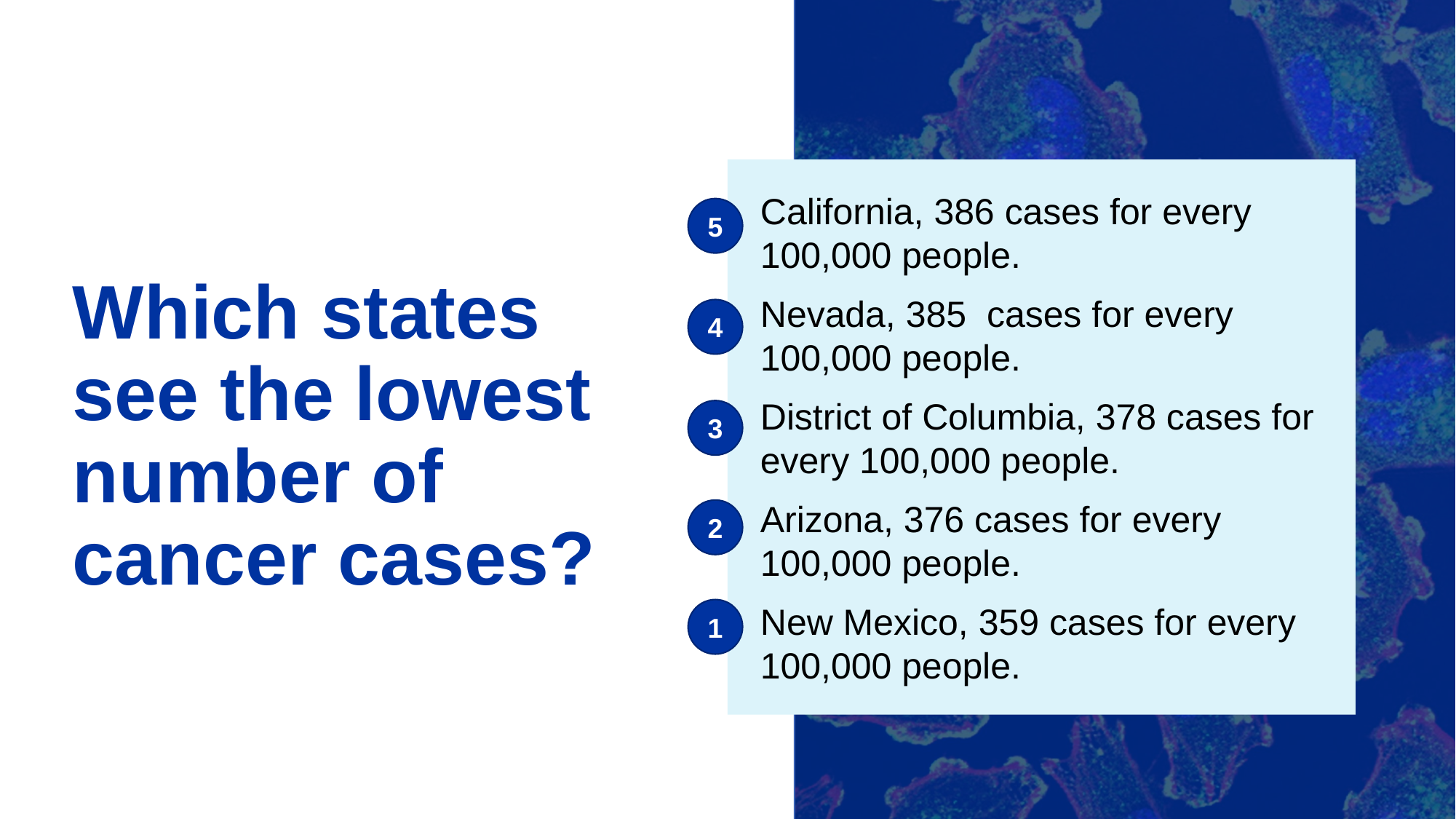

California, 386 cases for every 100,000 people.
Nevada, 385 cases for every 100,000 people.
District of Columbia, 378 cases for every 100,000 people.
Arizona, 376 cases for every 100,000 people.
New Mexico, 359 cases for every 100,000 people.
# Which states see the lowest number of cancer cases?
5
4
3
2
1

## Slide 17
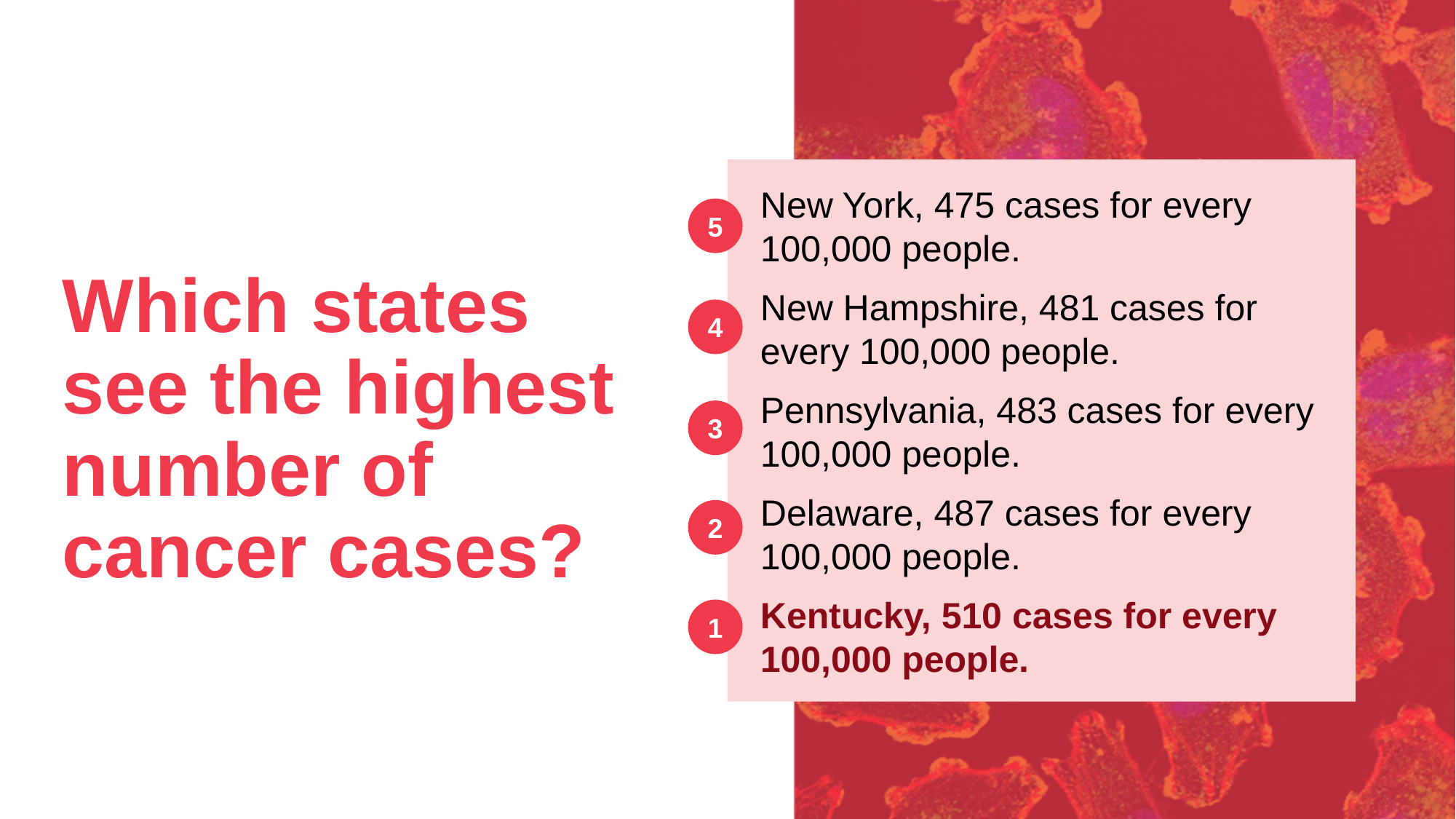

New York, 475 cases for every 100,000 people.
New Hampshire, 481 cases for every 100,000 people.
Pennsylvania, 483 cases for every 100,000 people.
Delaware, 487 cases for every 100,000 people.
Kentucky, 510 cases for every 100,000 people.
# Which states see the highest number of cancer cases?
5
4
3
2
1

## Slide 18
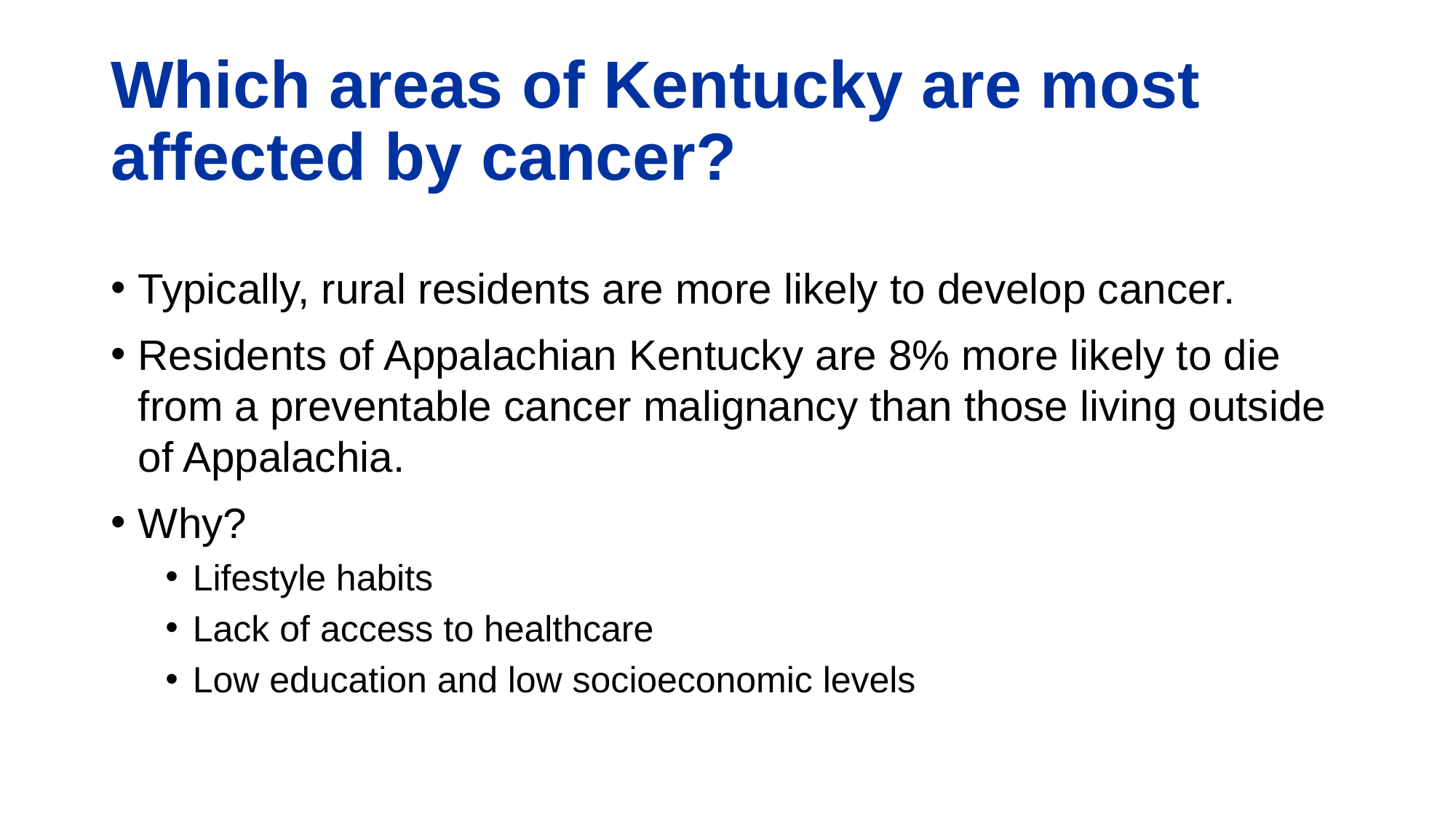

# Which areas of Kentucky are most affected by cancer?
Typically, rural residents are more likely to develop cancer.
Residents of Appalachian Kentucky are 8% more likely to die from a preventable cancer malignancy than those living outside of Appalachia.
Why?
Lifestyle habits
Lack of access to healthcare
Low education and low socioeconomic levels

## Slide 19
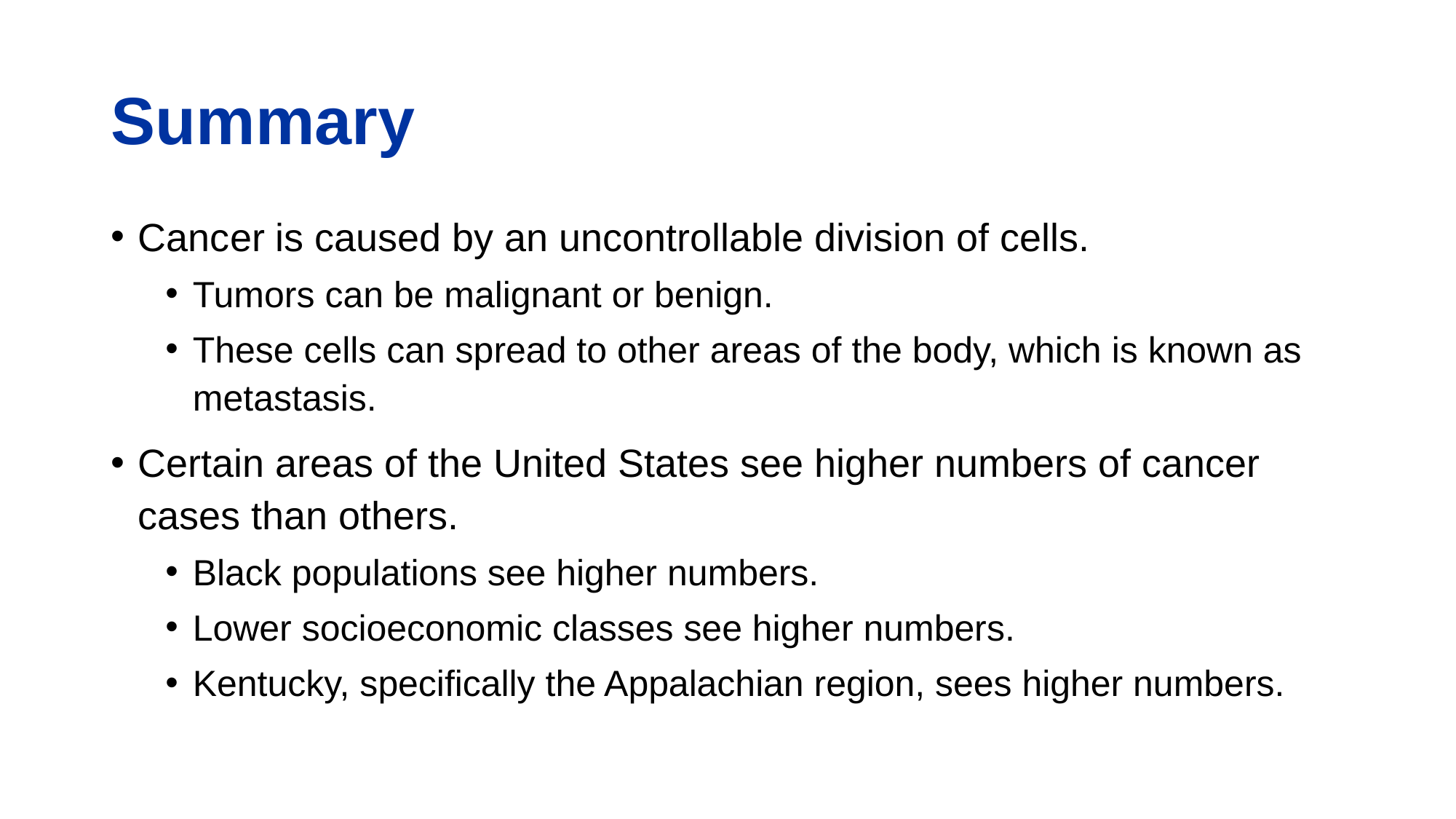

# Summary
Cancer is caused by an uncontrollable division of cells.
Tumors can be malignant or benign.
These cells can spread to other areas of the body, which is known as metastasis.
Certain areas of the United States see higher numbers of cancer cases than others.
Black populations see higher numbers.
Lower socioeconomic classes see higher numbers.
Kentucky, specifically the Appalachian region, sees higher numbers.

## Slide 20
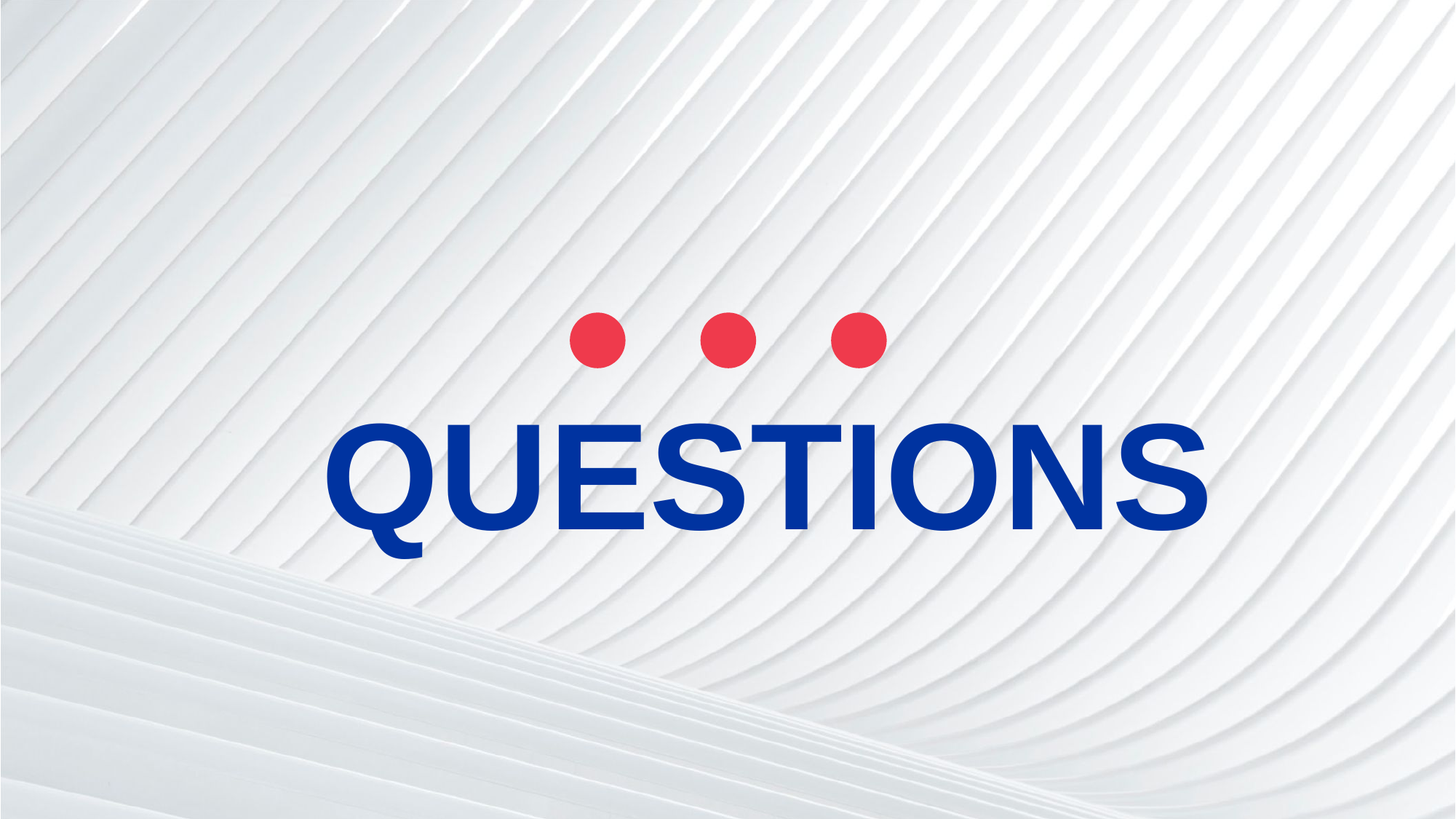

# QUESTIONS
