# Supplementary material for: Cancer Curriculum for Appalachian Kentucky Middle and High Schools
Source: J Appalach Health. 2021 Jan 24;3(1):43–55. doi: 10.13023/jah.0301.05 (PMC8830599; doi:10.13023/jah.0301.05)
Supplement: Supplementary file 2 [file Appendix3-3.1.5Hudson.pptx]

## Slide 1
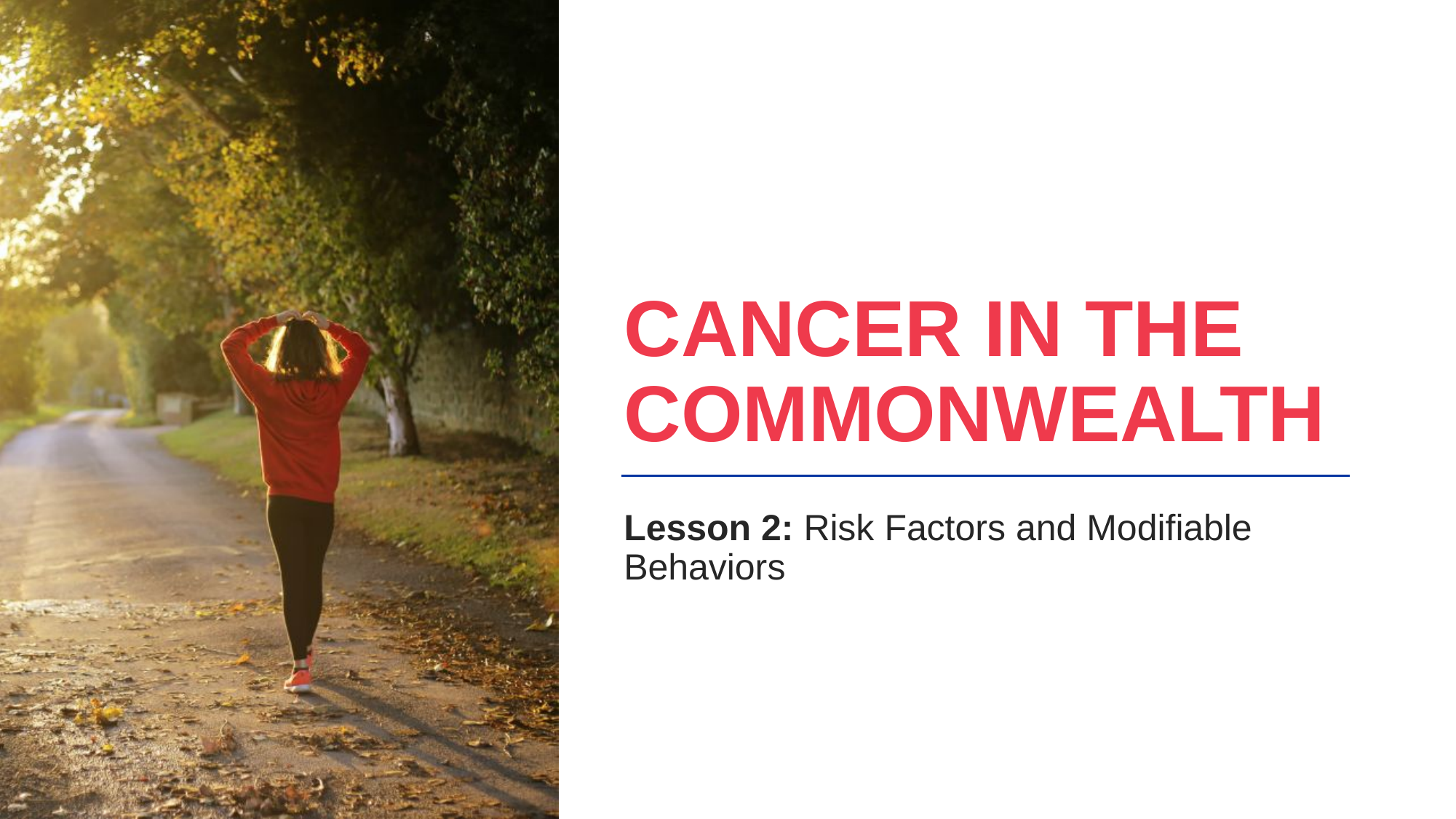

# CANCER IN THE COMMONWEALTH
Lesson 2: Risk Factors and Modifiable Behaviors

## Slide 2
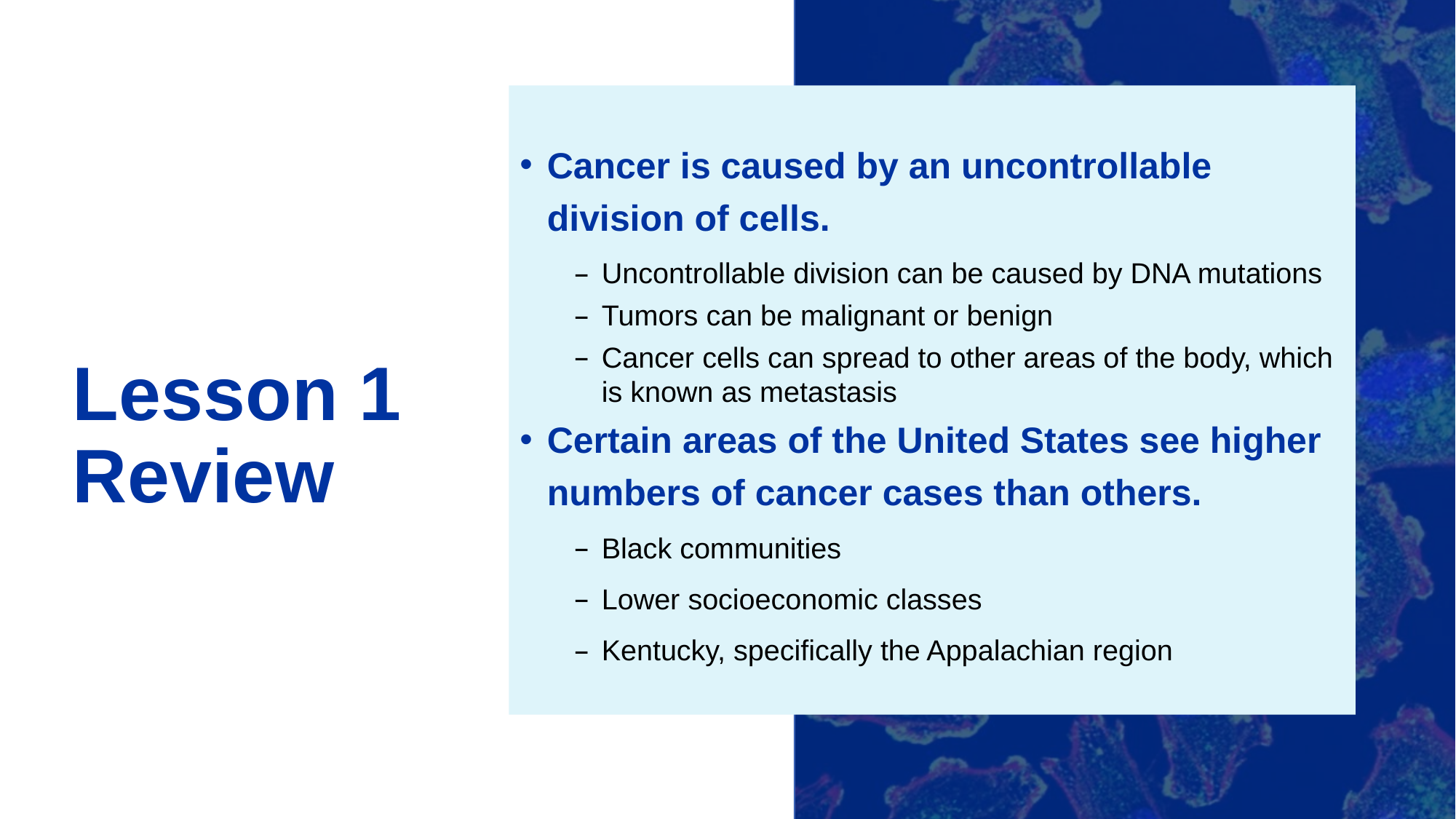

Cancer is caused by an uncontrollable division of cells.
Uncontrollable division can be caused by DNA mutations
Tumors can be malignant or benign
Cancer cells can spread to other areas of the body, which is known as metastasis
Certain areas of the United States see higher numbers of cancer cases than others.
Black communities
Lower socioeconomic classes
Kentucky, specifically the Appalachian region
# Lesson 1 Review

## Slide 3
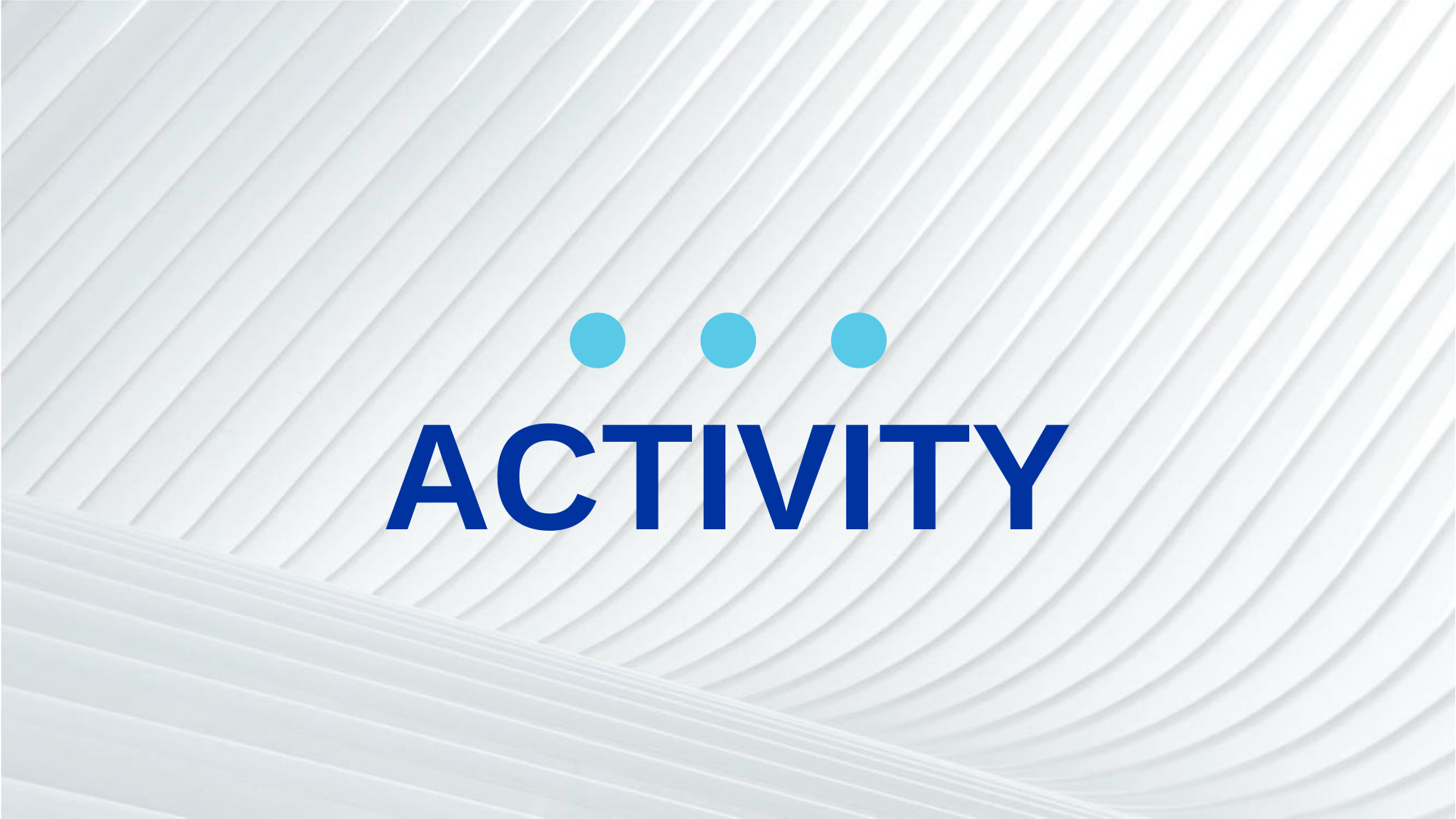

# ACTIVITY

## Slide 4
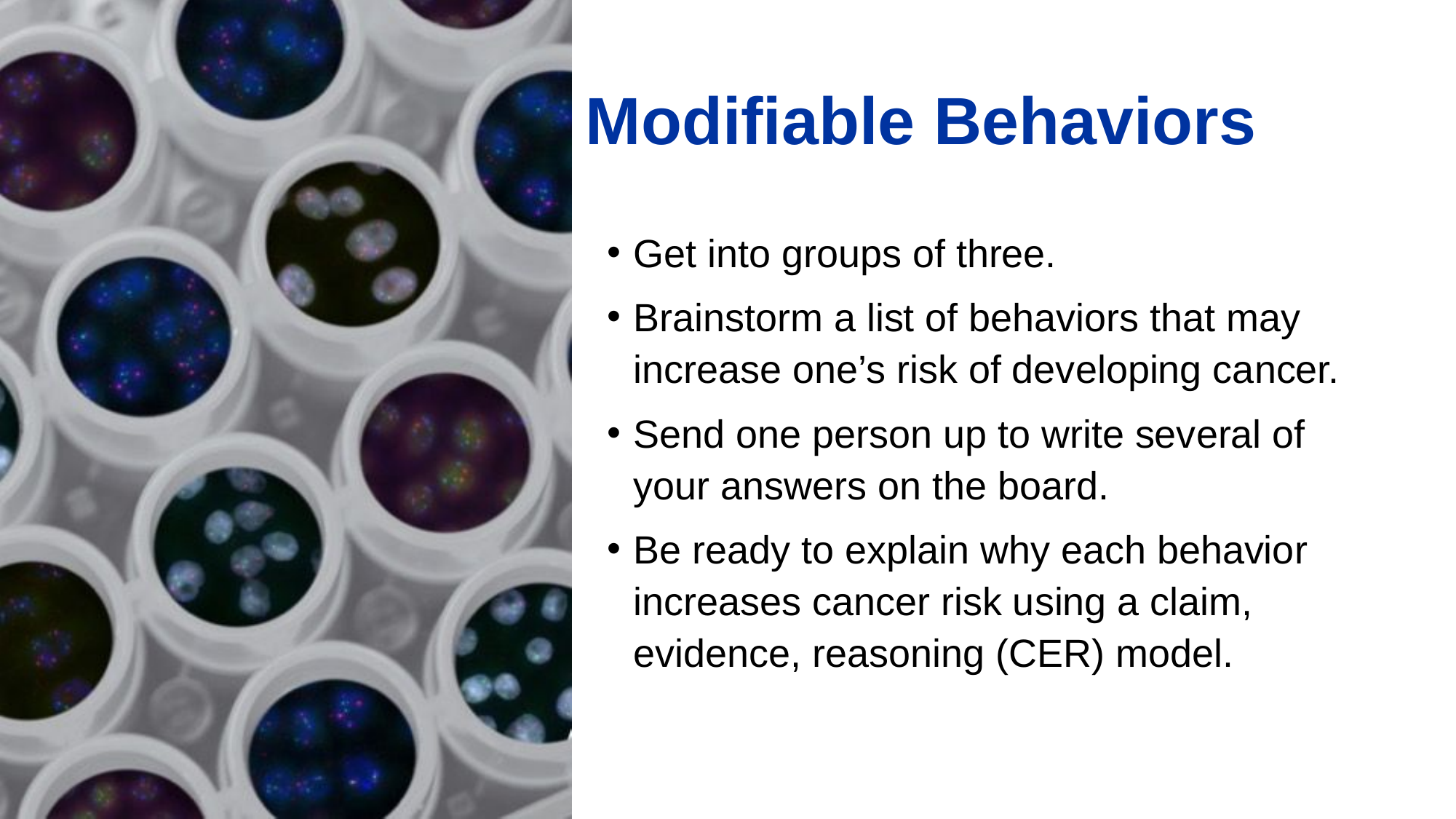

# Modifiable Behaviors
Get into groups of three.
Brainstorm a list of behaviors that may increase one’s risk of developing cancer.
Send one person up to write several of your answers on the board.
Be ready to explain why each behavior increases cancer risk using a claim, evidence, reasoning (CER) model.

## Slide 5
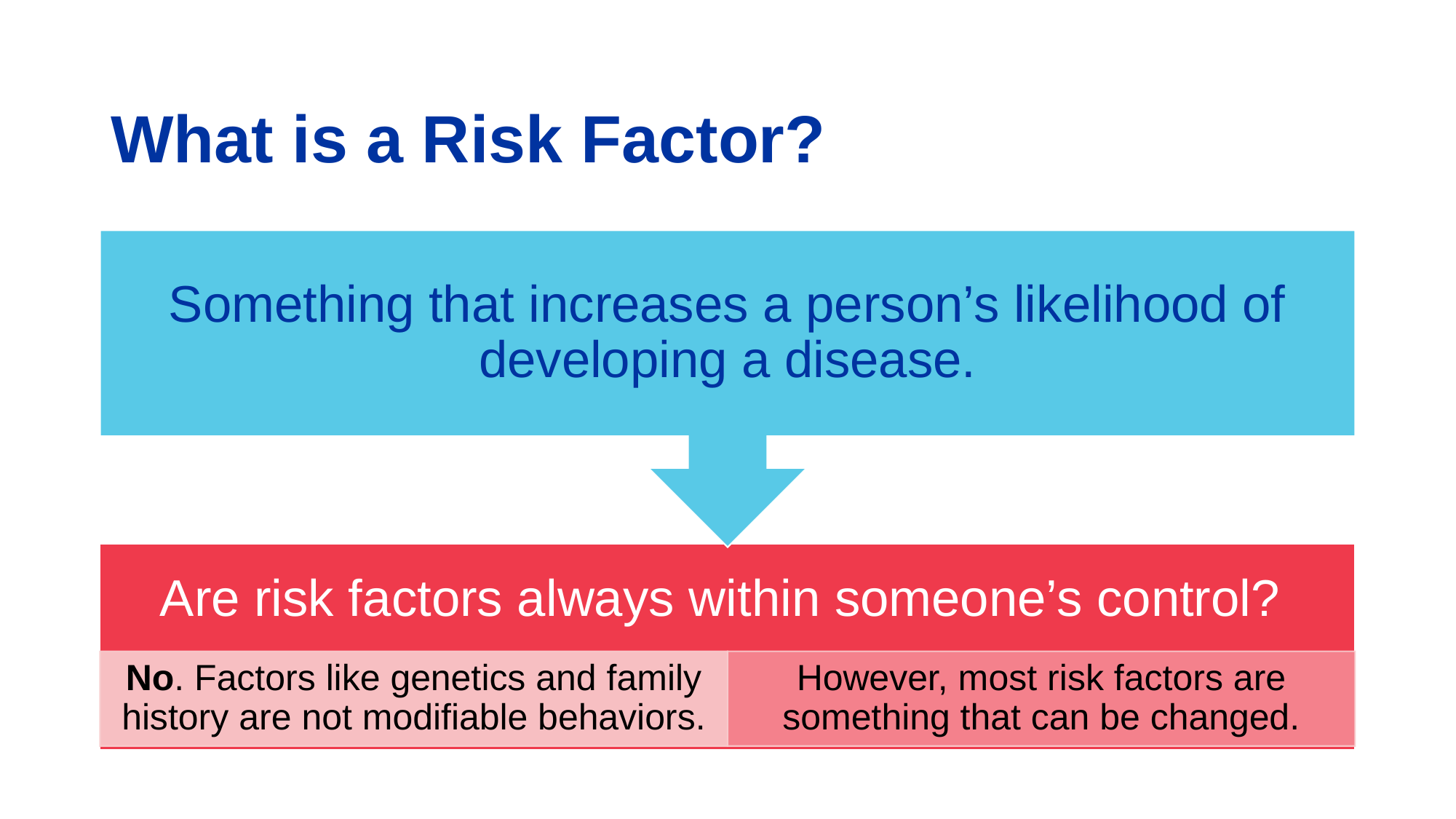

What is a Risk Factor?

## Slide 6
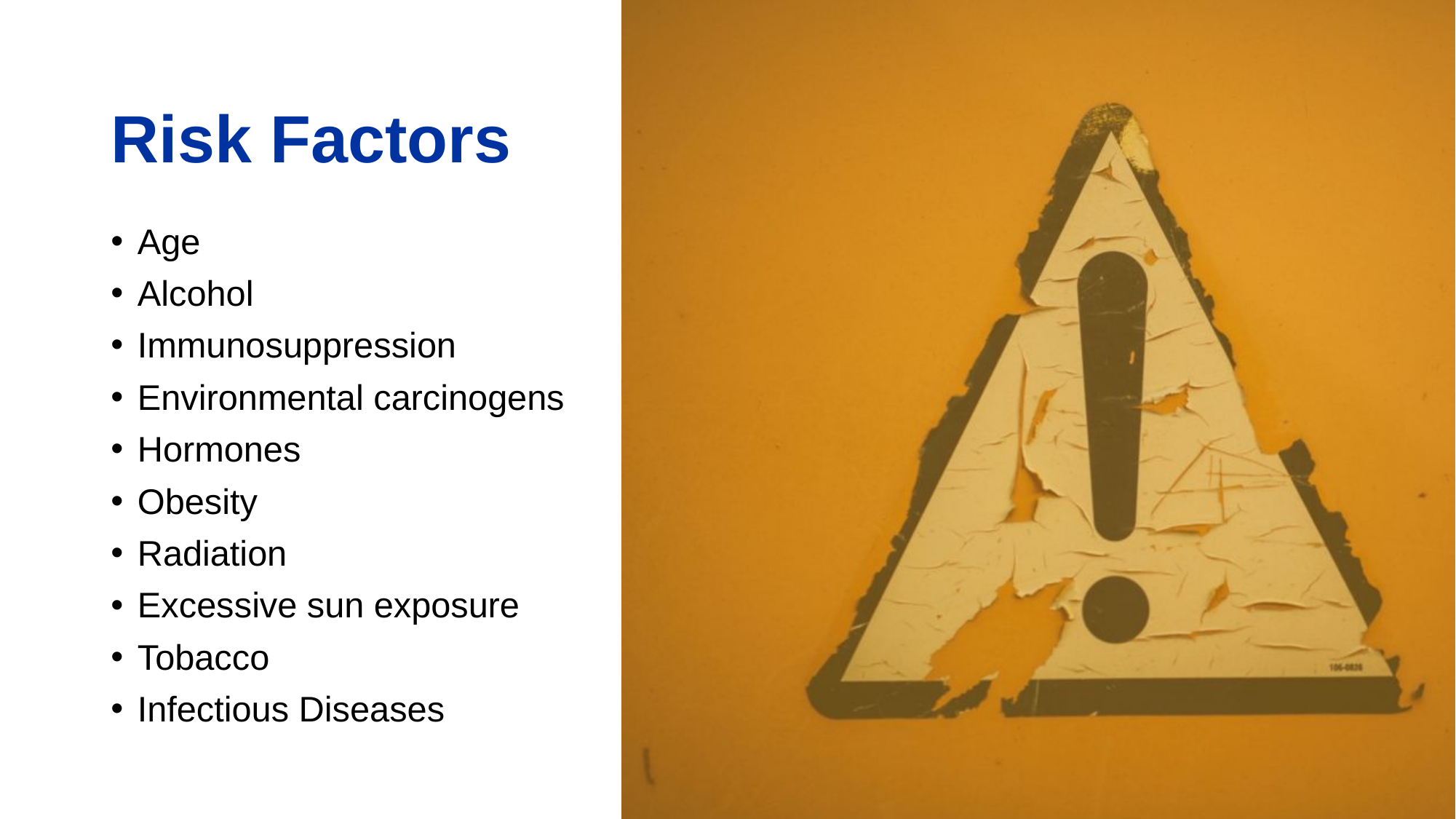

Risk Factors
Age
Alcohol
Immunosuppression
Environmental carcinogens
Hormones
Obesity
Radiation
Excessive sun exposure
Tobacco
Infectious Diseases

## Slide 7
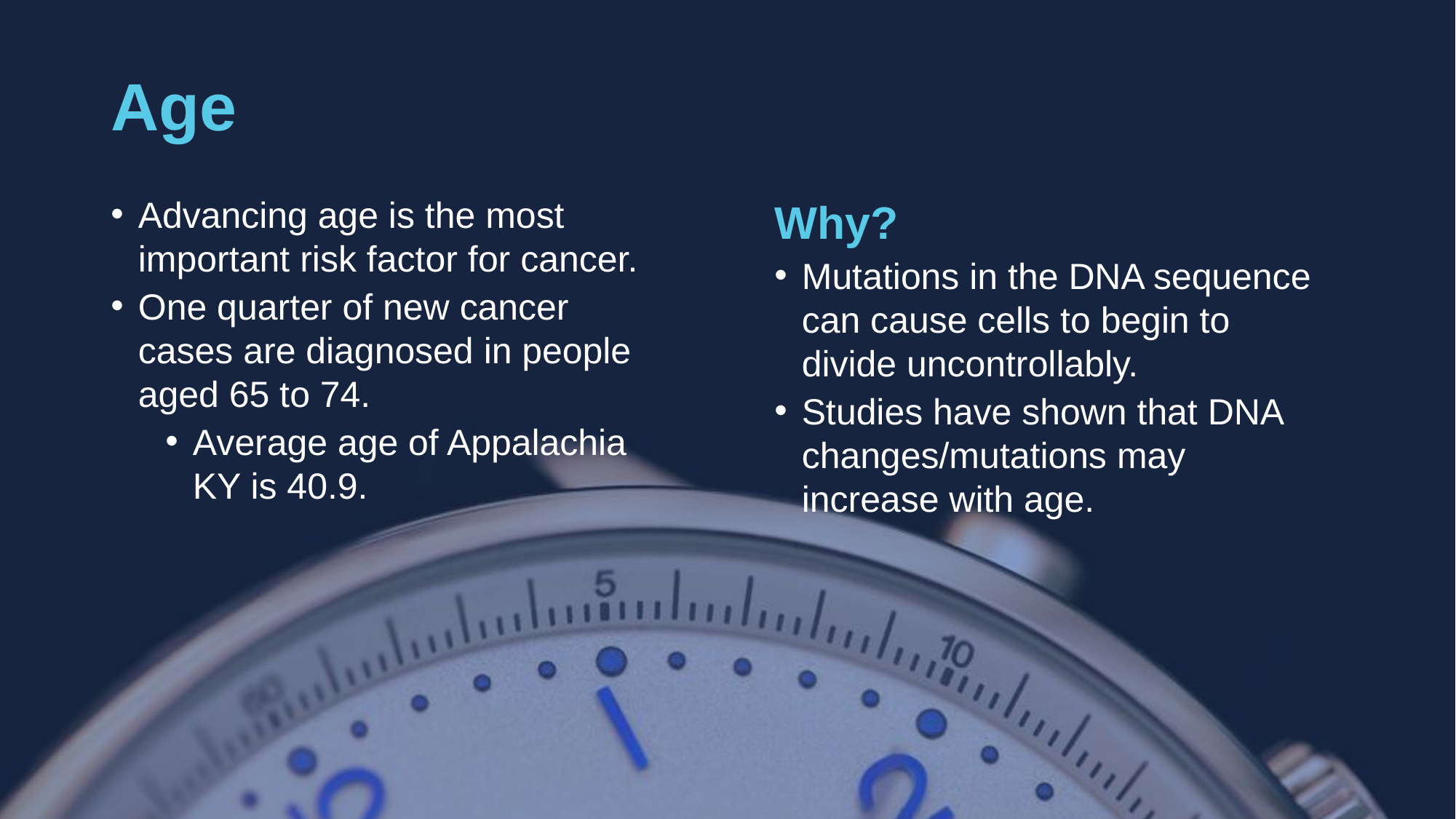

Age
Advancing age is the most important risk factor for cancer.
One quarter of new cancer cases are diagnosed in people aged 65 to 74.
Average age of Appalachia KY is 40.9.
Why?
Mutations in the DNA sequence can cause cells to begin to divide uncontrollably.
Studies have shown that DNA changes/mutations may increase with age.

## Slide 8
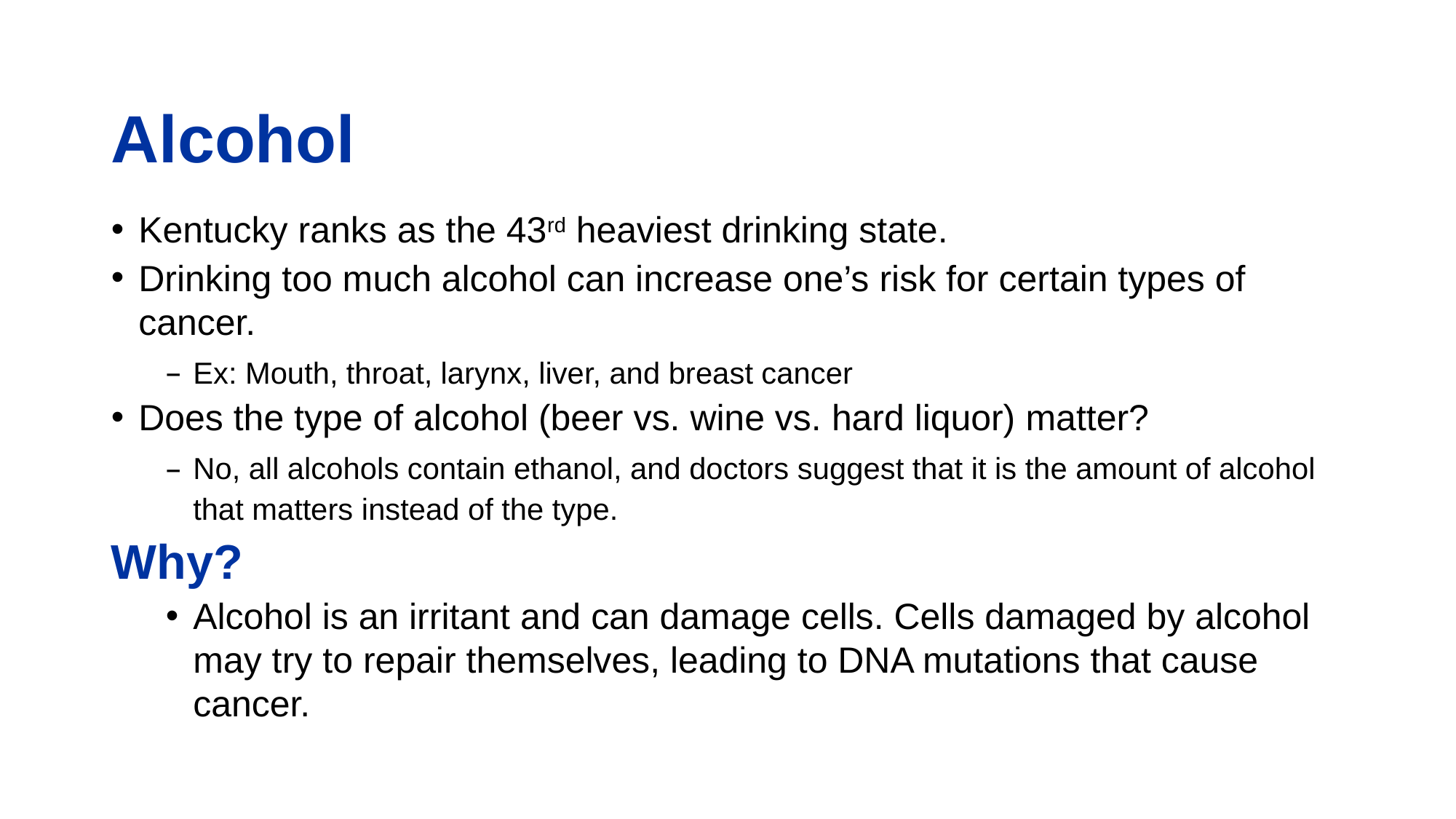

Alcohol
Kentucky ranks as the 43rd heaviest drinking state.
Drinking too much alcohol can increase one’s risk for certain types of cancer.
Ex: Mouth, throat, larynx, liver, and breast cancer
Does the type of alcohol (beer vs. wine vs. hard liquor) matter?
No, all alcohols contain ethanol, and doctors suggest that it is the amount of alcohol that matters instead of the type.
Why?
Alcohol is an irritant and can damage cells. Cells damaged by alcohol may try to repair themselves, leading to DNA mutations that cause cancer.

## Slide 9
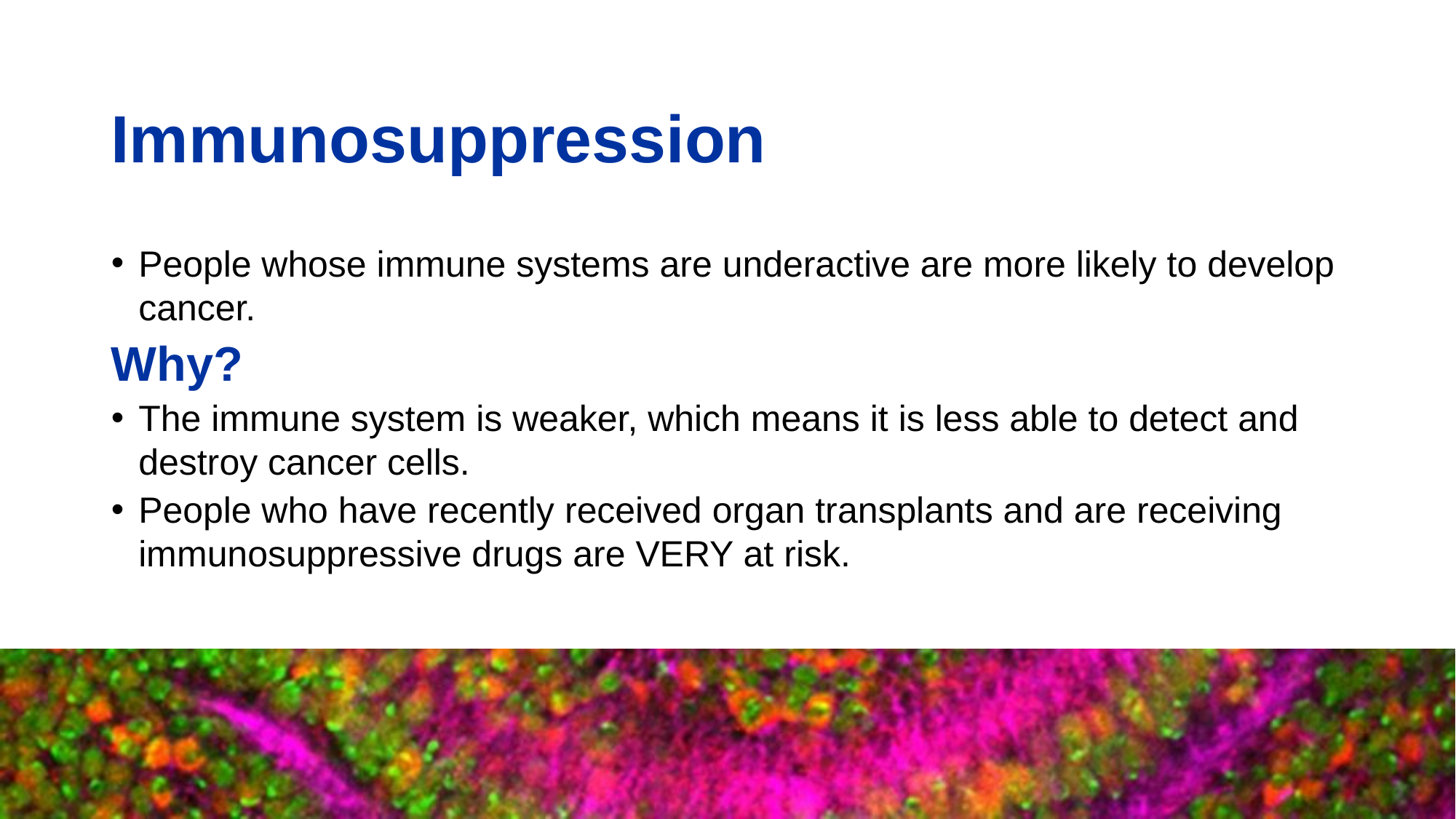

Immunosuppression
People whose immune systems are underactive are more likely to develop cancer.
Why?
The immune system is weaker, which means it is less able to detect and destroy cancer cells.
People who have recently received organ transplants and are receiving immunosuppressive drugs are VERY at risk.

## Slide 10
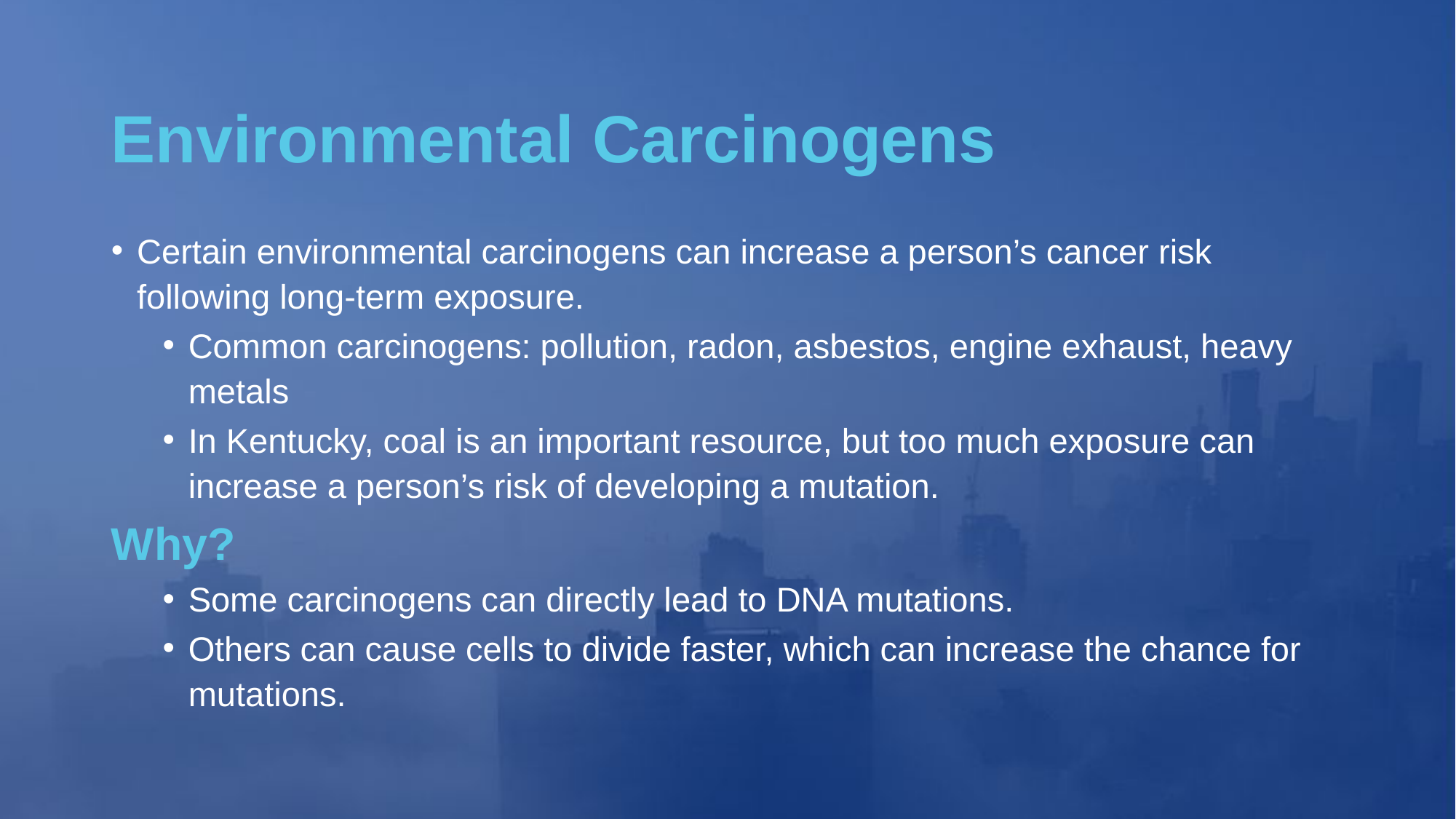

Environmental Carcinogens
Certain environmental carcinogens can increase a person’s cancer risk following long-term exposure.
Common carcinogens: pollution, radon, asbestos, engine exhaust, heavy metals
In Kentucky, coal is an important resource, but too much exposure can increase a person’s risk of developing a mutation.
Why?
Some carcinogens can directly lead to DNA mutations.
Others can cause cells to divide faster, which can increase the chance for mutations.

## Slide 11
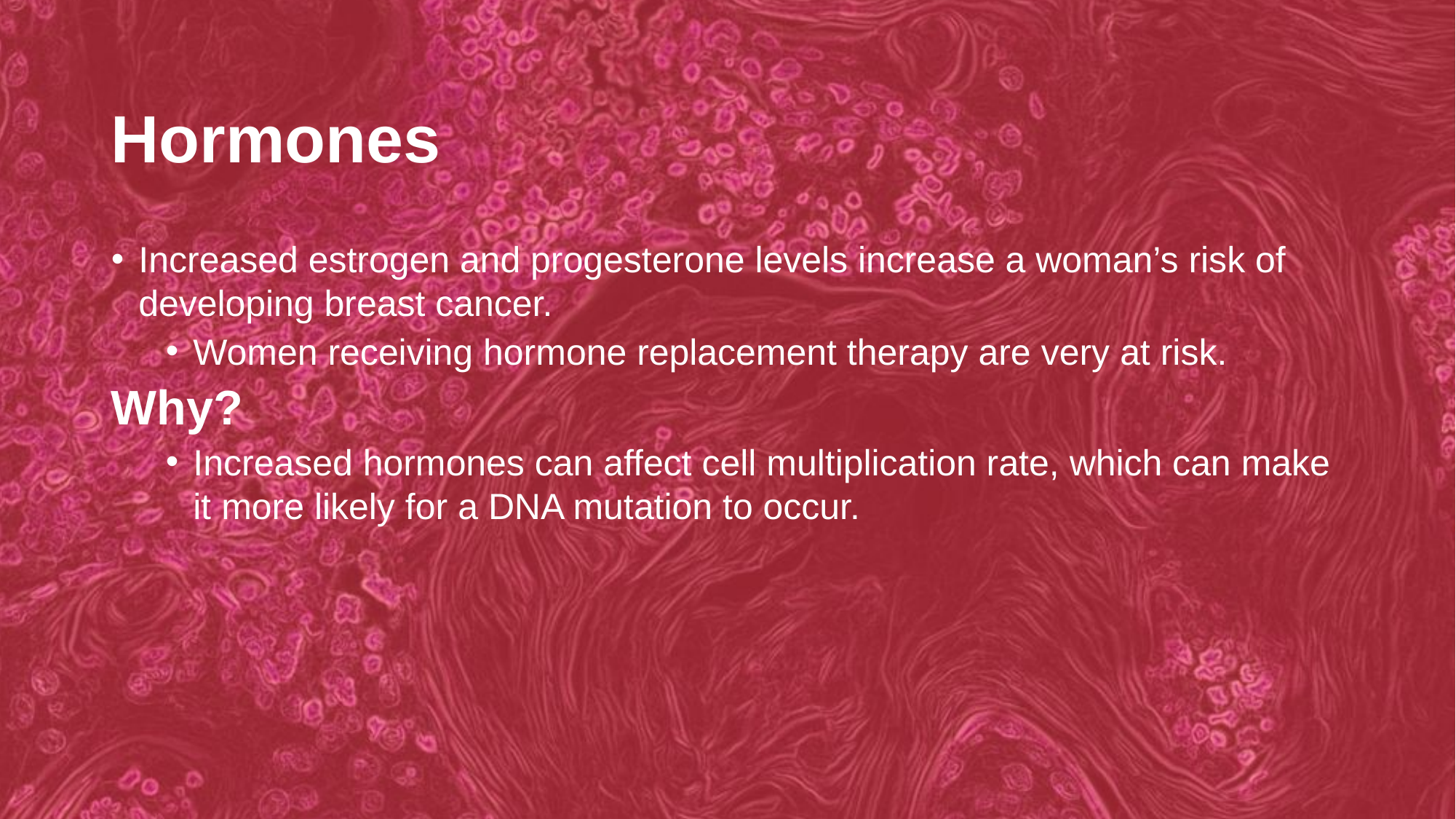

Hormones
Increased estrogen and progesterone levels increase a woman’s risk of developing breast cancer.
Women receiving hormone replacement therapy are very at risk.
Why?
Increased hormones can affect cell multiplication rate, which can make it more likely for a DNA mutation to occur.

## Slide 12
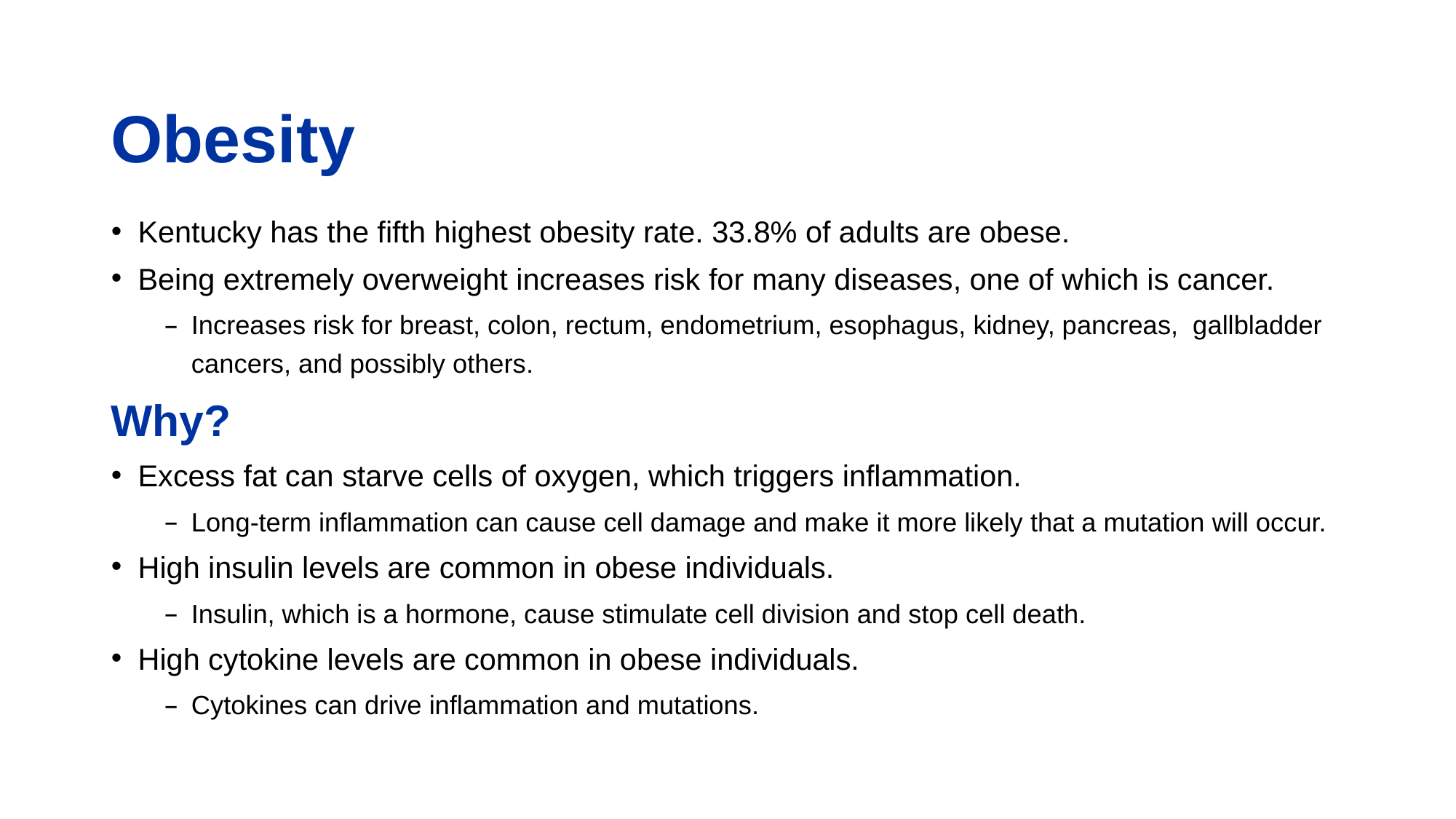

Obesity
Kentucky has the fifth highest obesity rate. 33.8% of adults are obese.
Being extremely overweight increases risk for many diseases, one of which is cancer.
Increases risk for breast, colon, rectum, endometrium, esophagus, kidney, pancreas, gallbladder cancers, and possibly others.
Why?
Excess fat can starve cells of oxygen, which triggers inflammation.
Long-term inflammation can cause cell damage and make it more likely that a mutation will occur.
High insulin levels are common in obese individuals.
Insulin, which is a hormone, cause stimulate cell division and stop cell death.
High cytokine levels are common in obese individuals.
Cytokines can drive inflammation and mutations.

## Slide 13
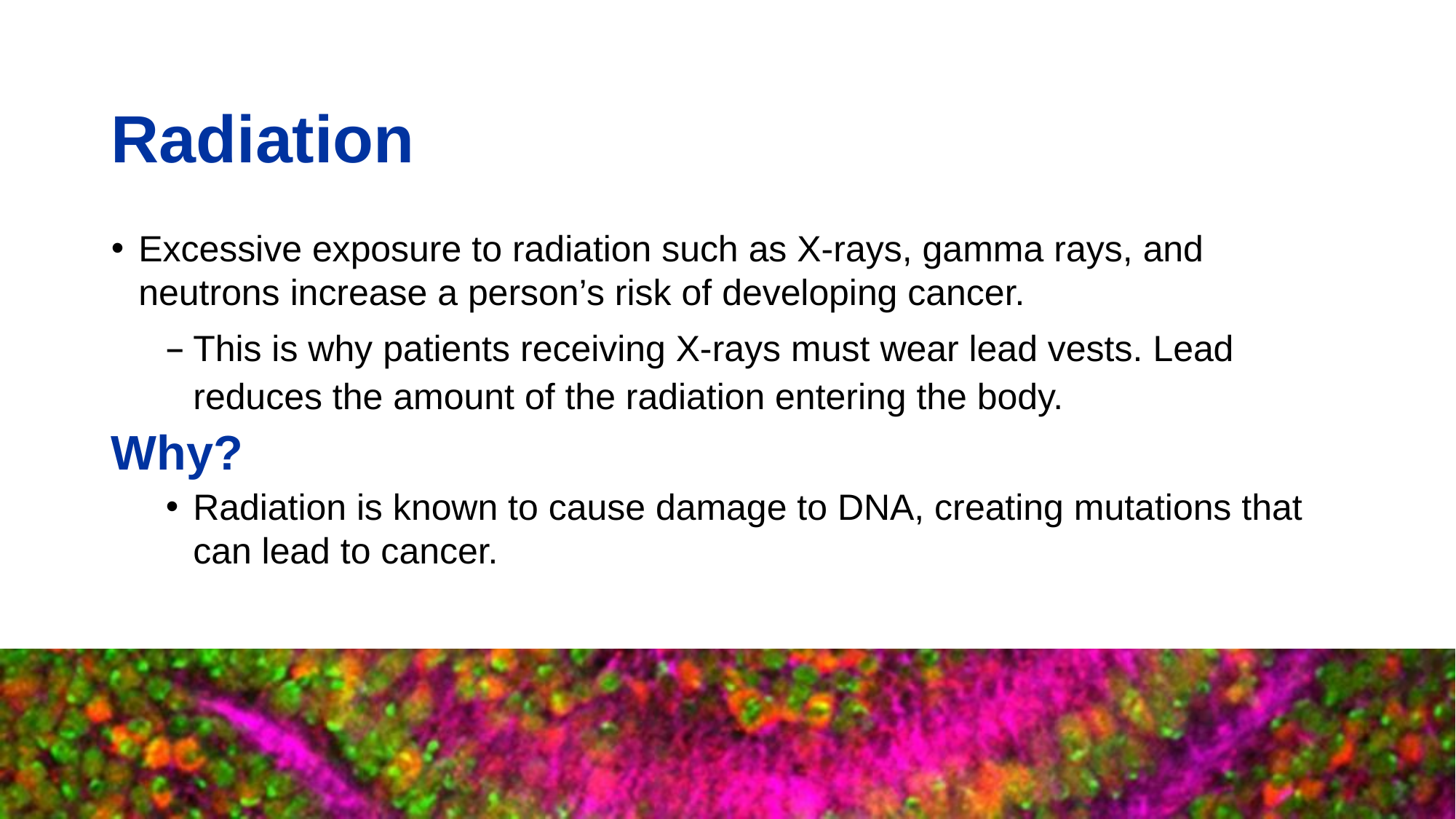

Radiation
Excessive exposure to radiation such as X-rays, gamma rays, and neutrons increase a person’s risk of developing cancer.
This is why patients receiving X-rays must wear lead vests. Lead reduces the amount of the radiation entering the body.
Why?
Radiation is known to cause damage to DNA, creating mutations that can lead to cancer.

## Slide 14
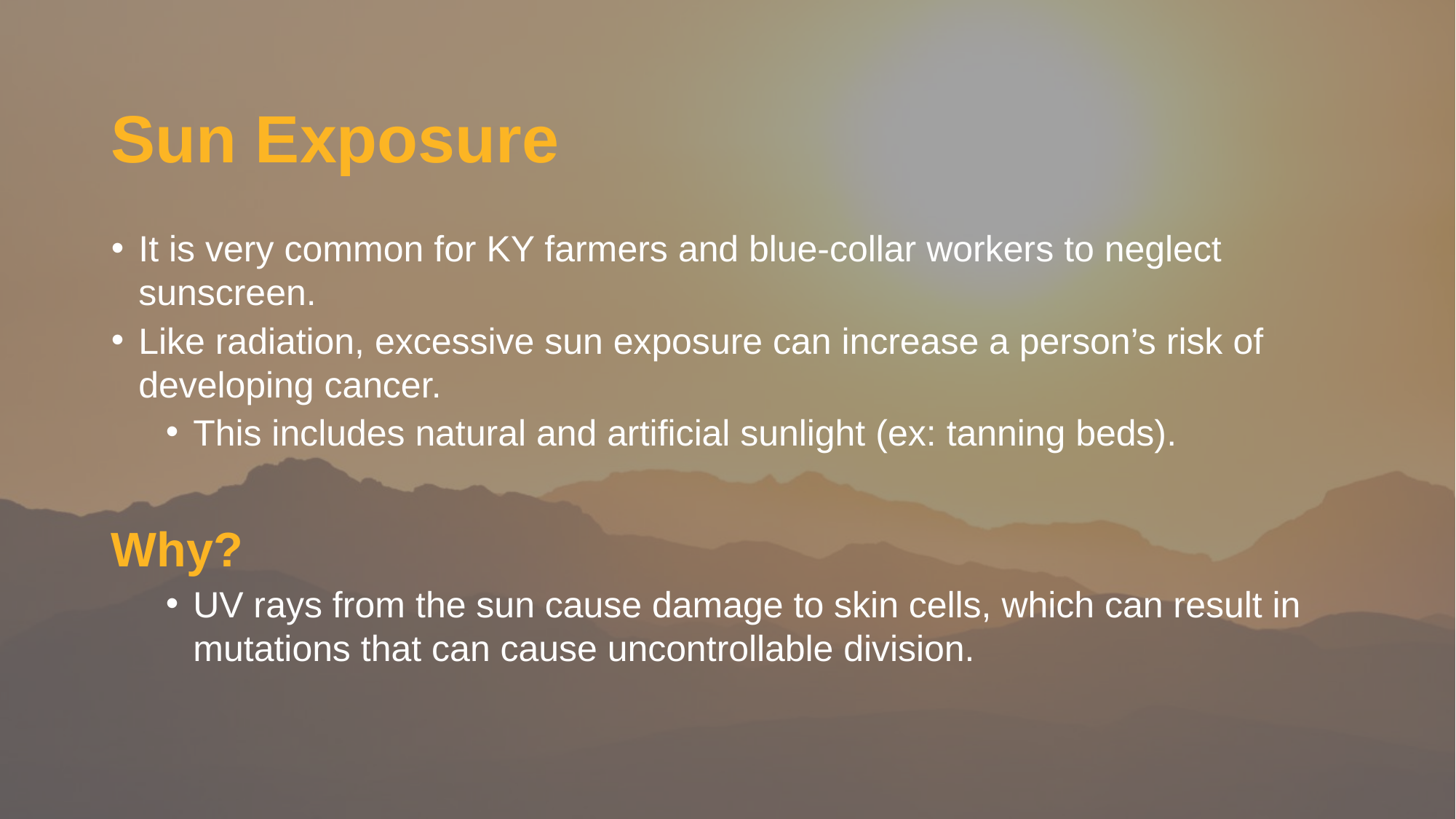

Sun Exposure
It is very common for KY farmers and blue-collar workers to neglect sunscreen.
Like radiation, excessive sun exposure can increase a person’s risk of developing cancer.
This includes natural and artificial sunlight (ex: tanning beds).
Why?
UV rays from the sun cause damage to skin cells, which can result in mutations that can cause uncontrollable division.

## Slide 15
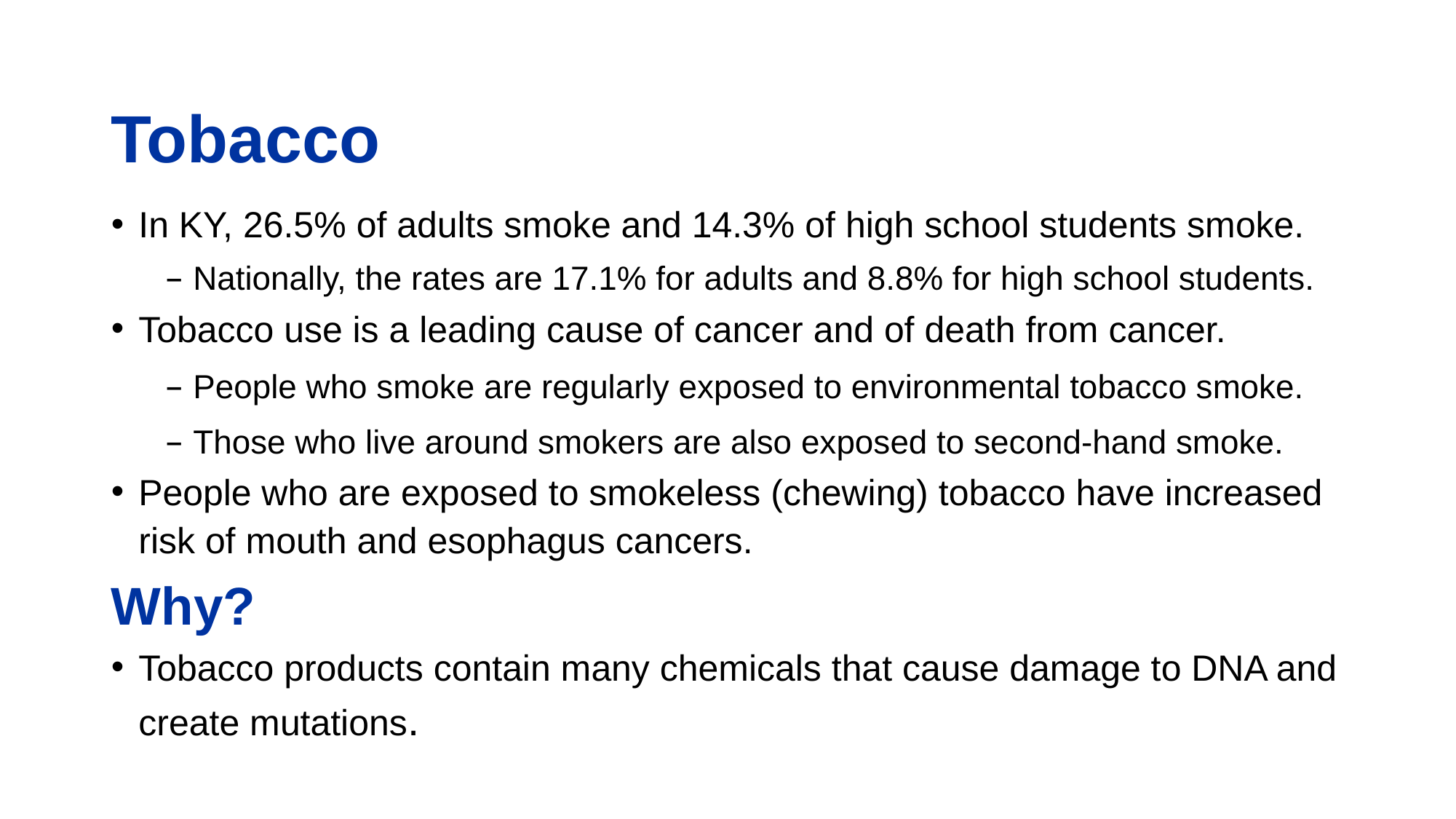

Tobacco
In KY, 26.5% of adults smoke and 14.3% of high school students smoke.
Nationally, the rates are 17.1% for adults and 8.8% for high school students.
Tobacco use is a leading cause of cancer and of death from cancer.
People who smoke are regularly exposed to environmental tobacco smoke.
Those who live around smokers are also exposed to second-hand smoke.
People who are exposed to smokeless (chewing) tobacco have increased risk of mouth and esophagus cancers.
Why?
Tobacco products contain many chemicals that cause damage to DNA and create mutations.

## Slide 16
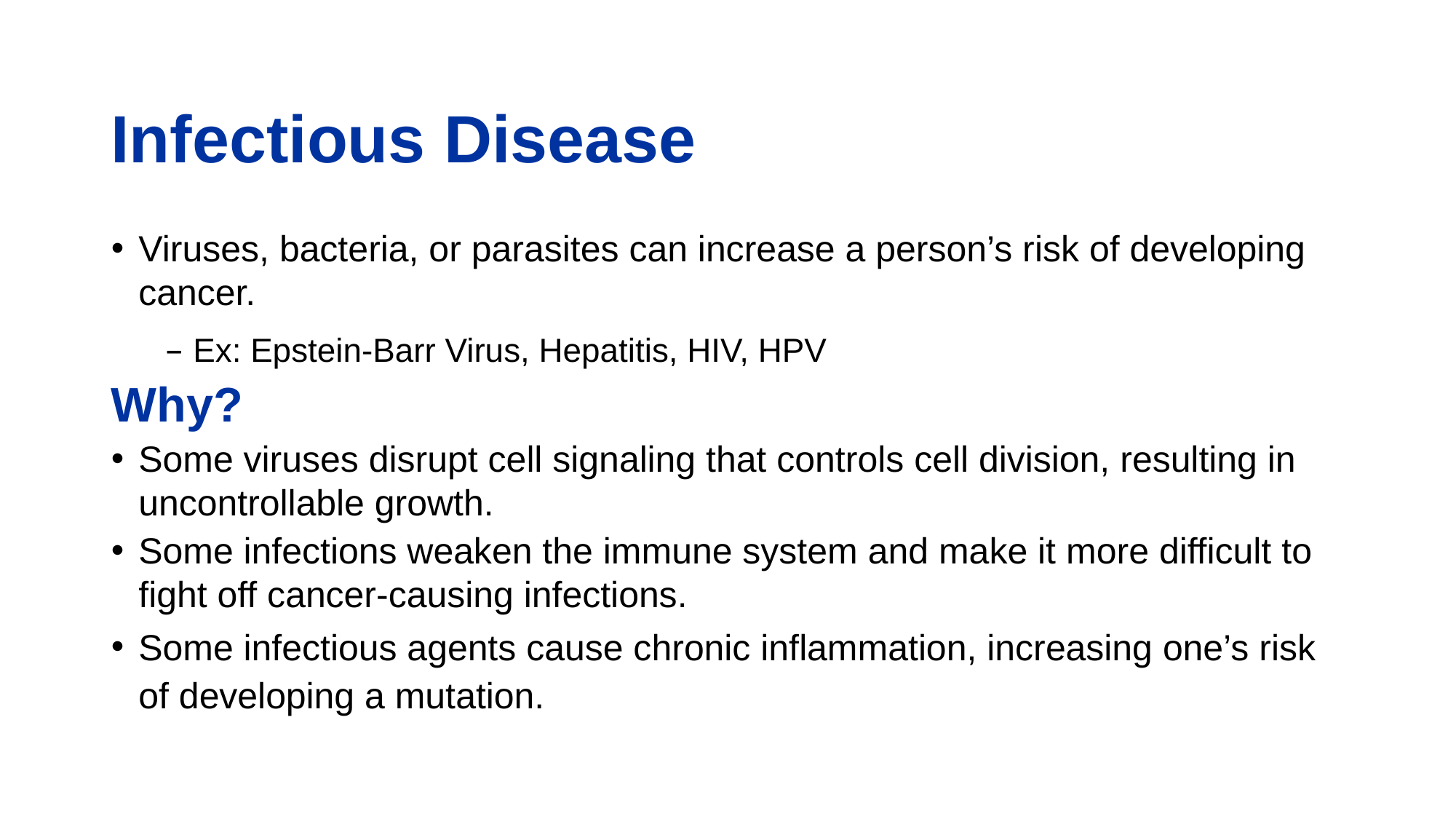

Infectious Disease
Viruses, bacteria, or parasites can increase a person’s risk of developing cancer.
Ex: Epstein-Barr Virus, Hepatitis, HIV, HPV
Why?
Some viruses disrupt cell signaling that controls cell division, resulting in uncontrollable growth.
Some infections weaken the immune system and make it more difficult to fight off cancer-causing infections.
Some infectious agents cause chronic inflammation, increasing one’s risk of developing a mutation.

## Slide 17
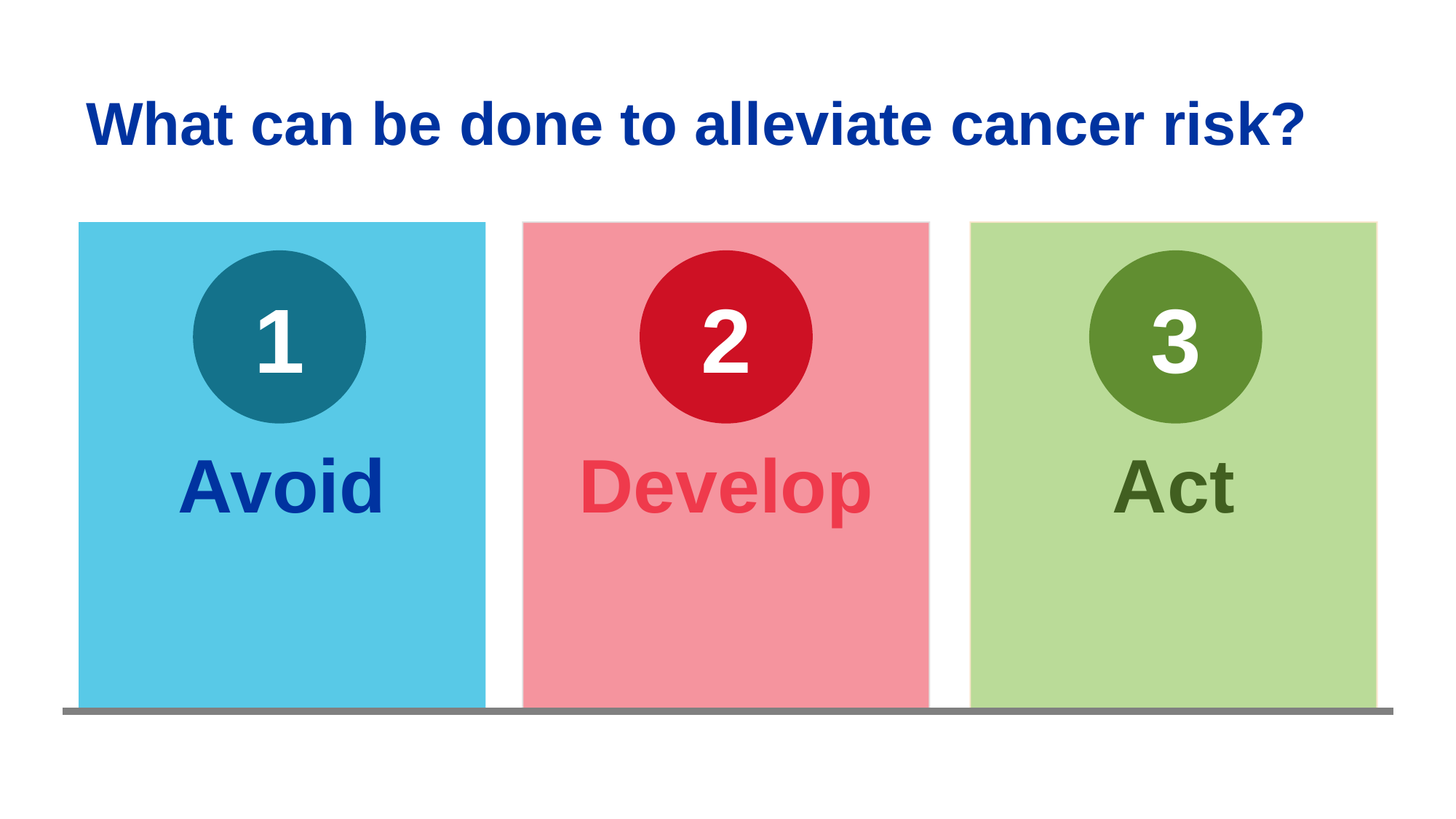

# What can be done to alleviate cancer risk?
1
2
3

## Slide 18
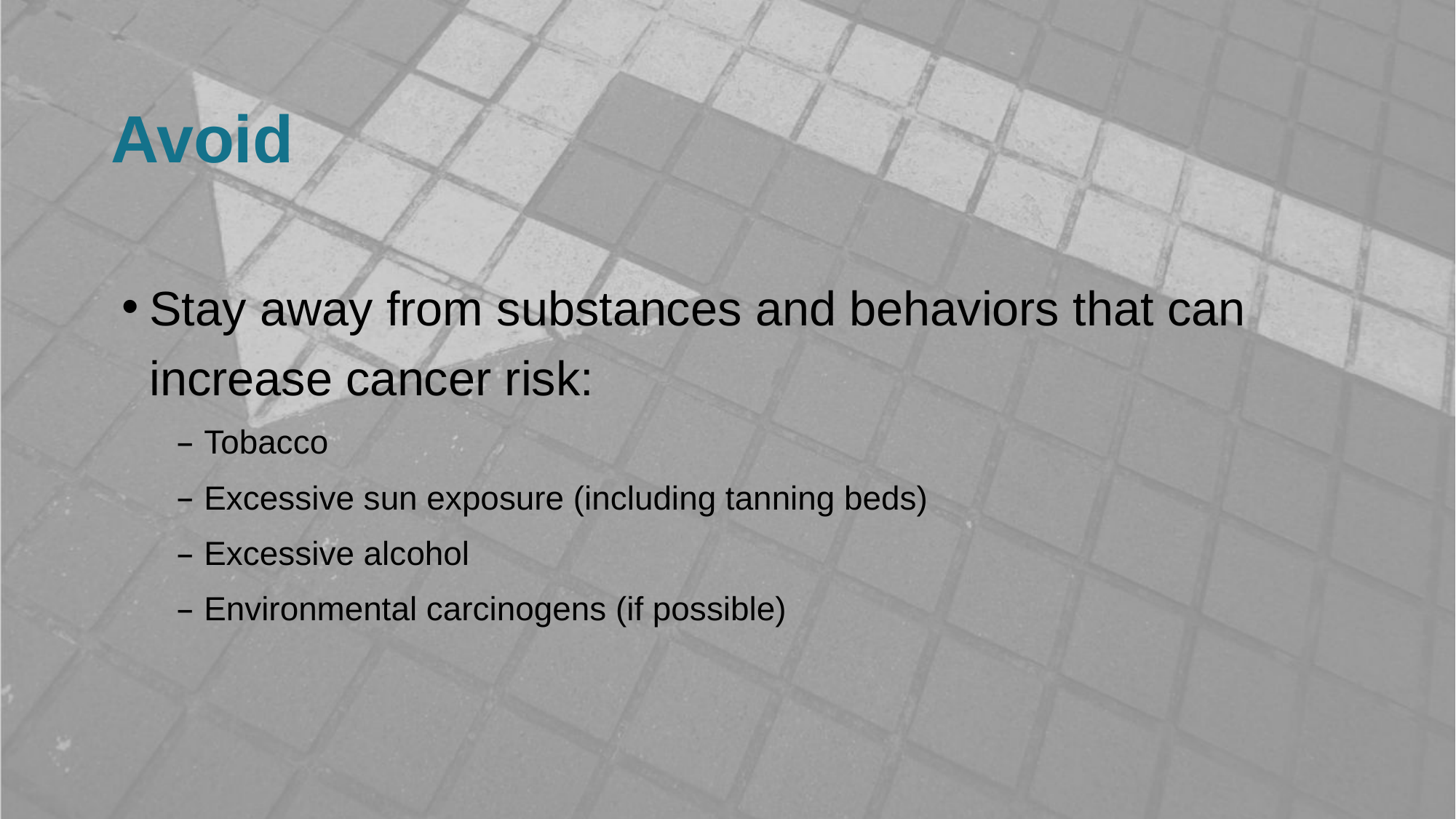

Avoid
Stay away from substances and behaviors that can increase cancer risk:
Tobacco
Excessive sun exposure (including tanning beds)
Excessive alcohol
Environmental carcinogens (if possible)

## Slide 19
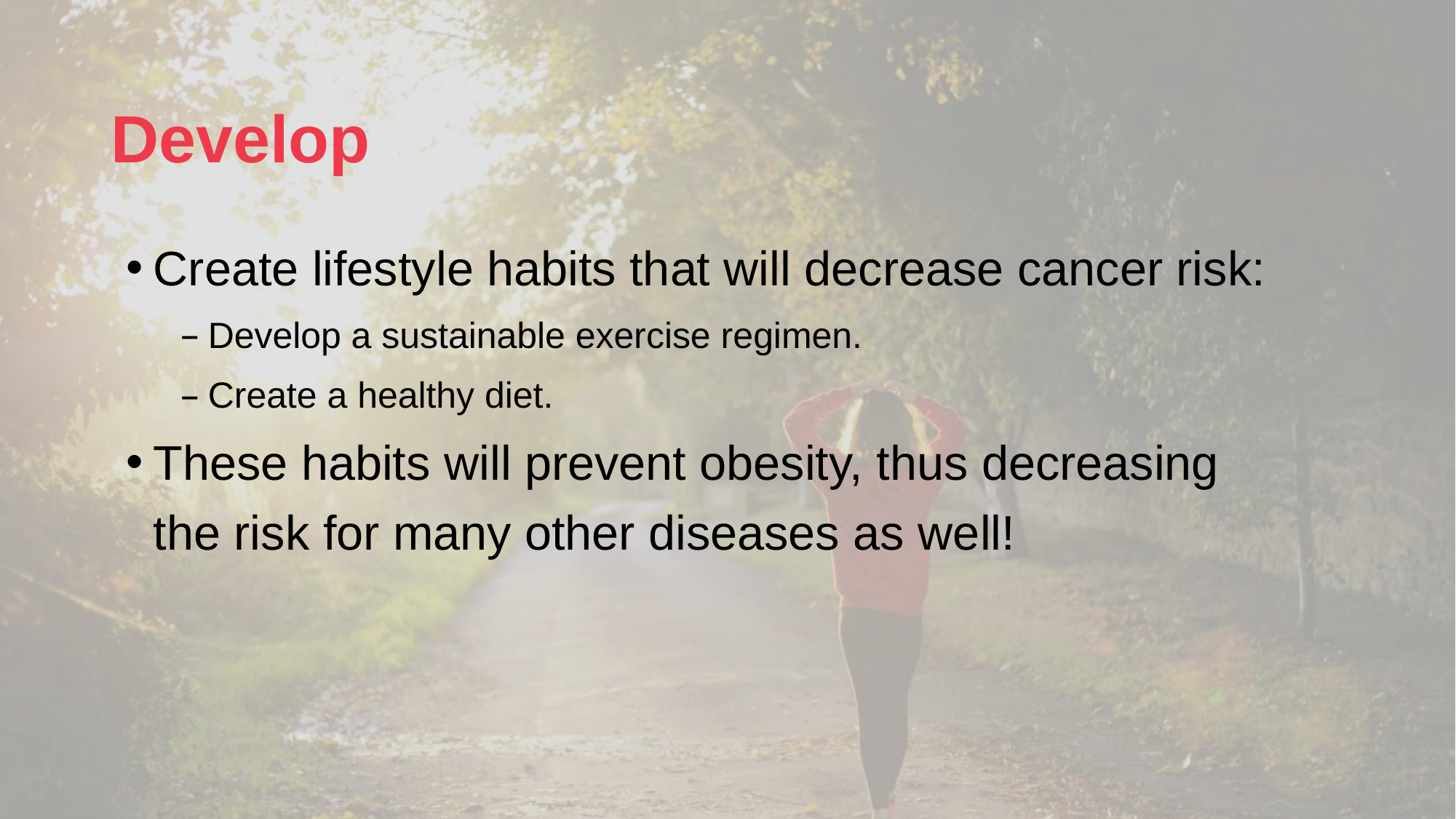

Develop
Create lifestyle habits that will decrease cancer risk:
Develop a sustainable exercise regimen.
Create a healthy diet.
These habits will prevent obesity, thus decreasing the risk for many other diseases as well!

## Slide 20
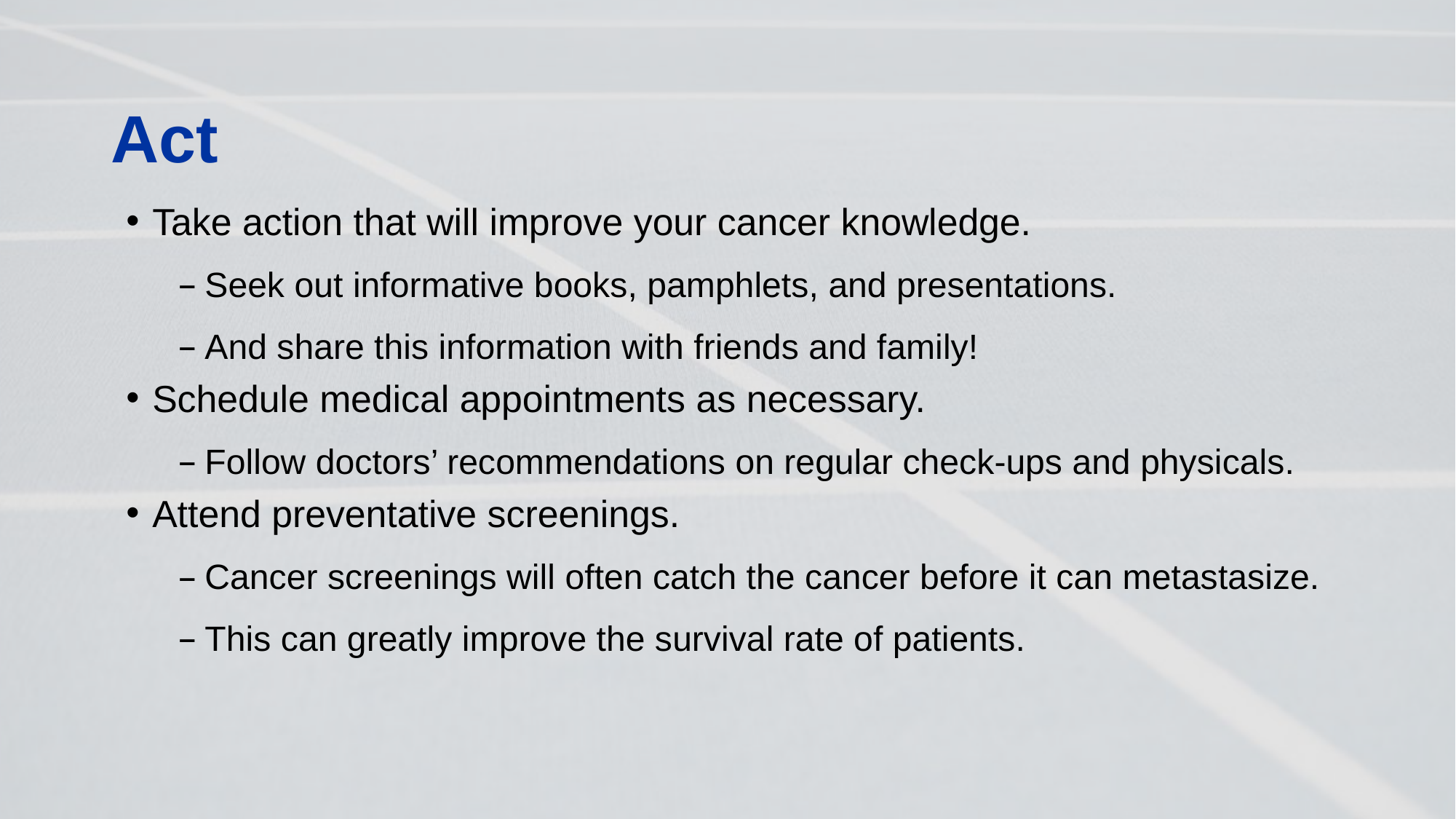

Act
Take action that will improve your cancer knowledge.
Seek out informative books, pamphlets, and presentations.
And share this information with friends and family!
Schedule medical appointments as necessary.
Follow doctors’ recommendations on regular check-ups and physicals.
Attend preventative screenings.
Cancer screenings will often catch the cancer before it can metastasize.
This can greatly improve the survival rate of patients.

## Slide 21
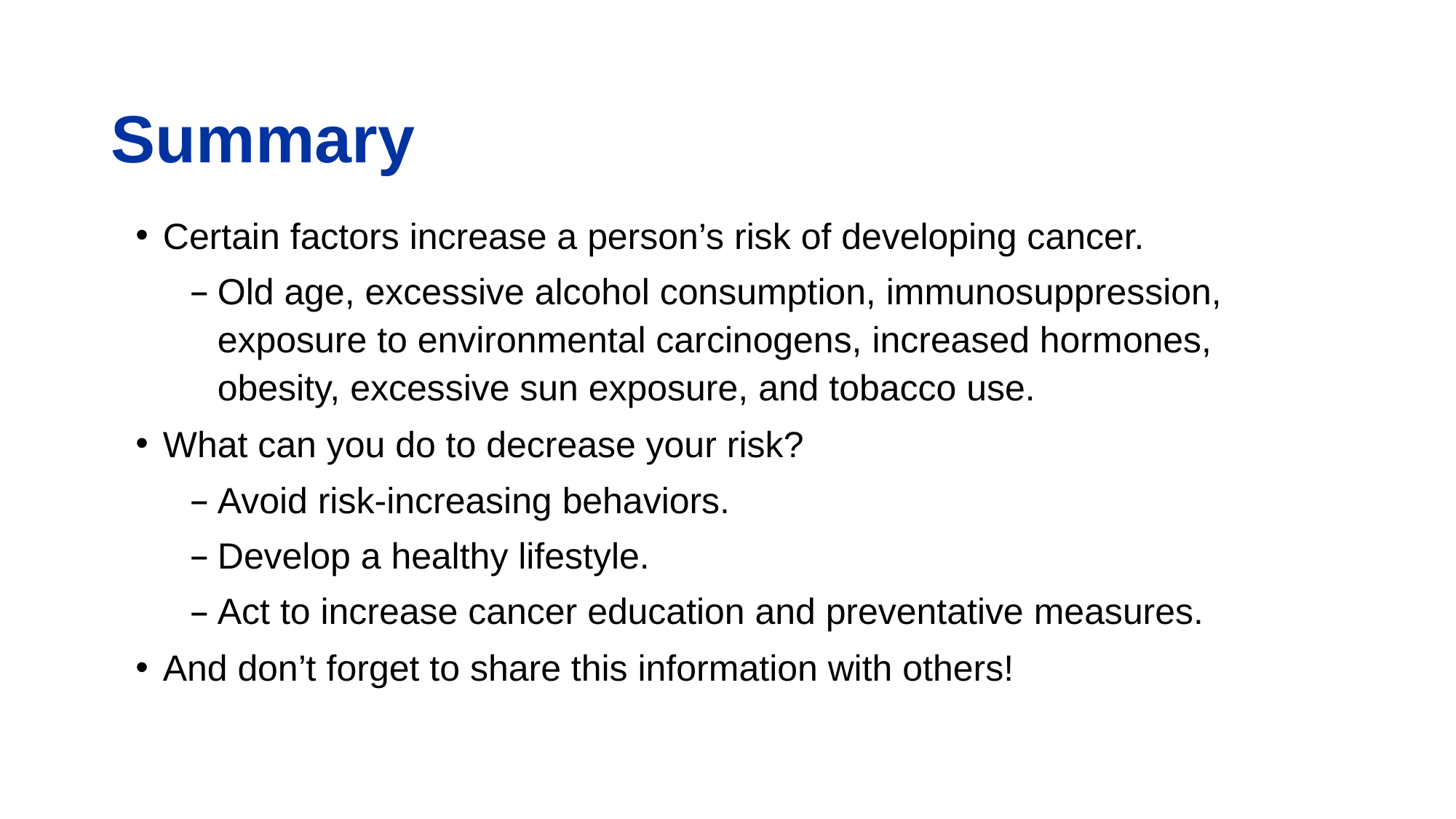

Summary
Certain factors increase a person’s risk of developing cancer.
Old age, excessive alcohol consumption, immunosuppression, exposure to environmental carcinogens, increased hormones, obesity, excessive sun exposure, and tobacco use.
What can you do to decrease your risk?
Avoid risk-increasing behaviors.
Develop a healthy lifestyle.
Act to increase cancer education and preventative measures.
And don’t forget to share this information with others!

## Slide 22
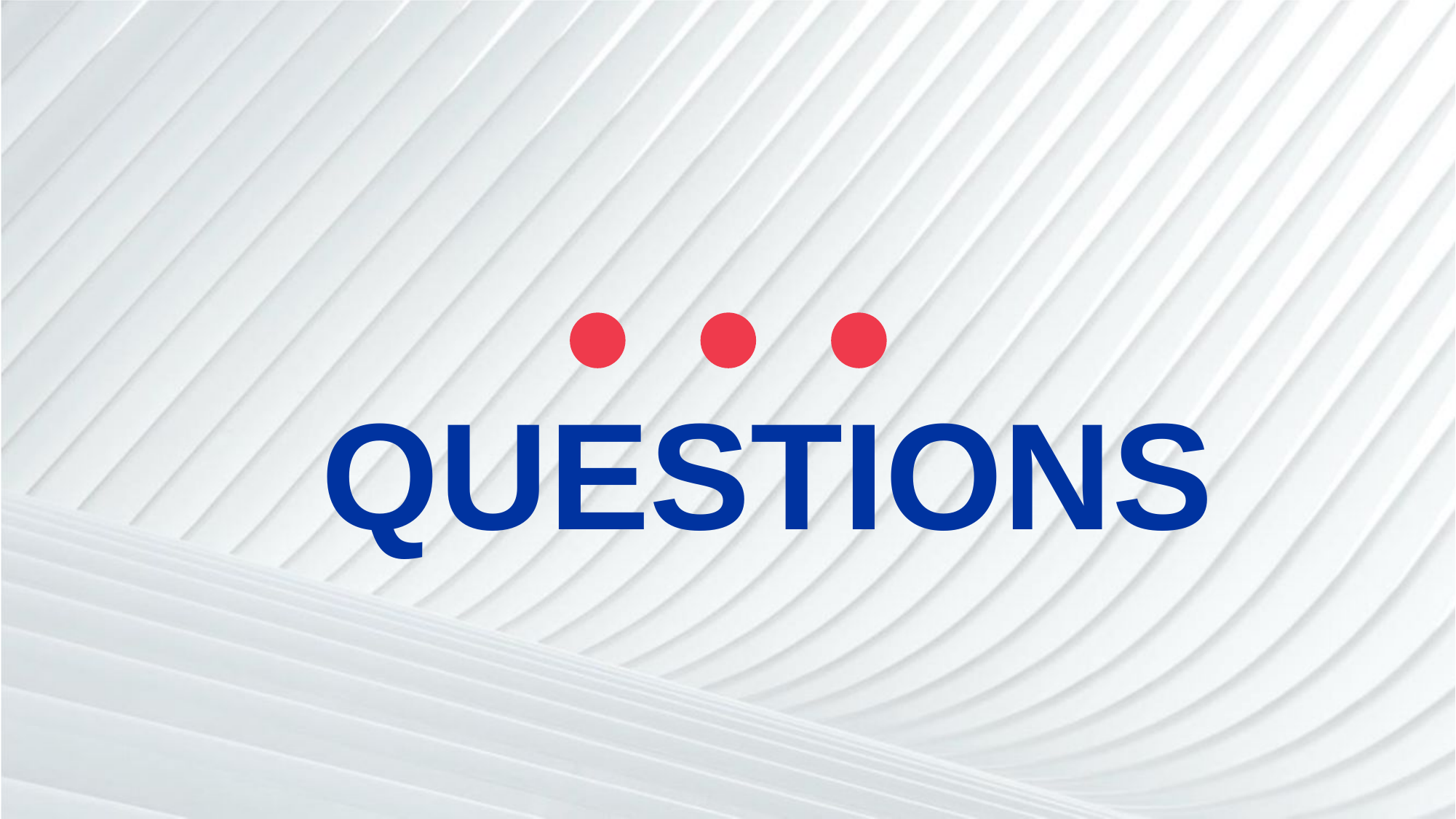

# QUESTIONS
