# Supplementary material for: Cancer Curriculum for Appalachian Kentucky Middle and High Schools
Source: J Appalach Health. 2021 Jan 24;3(1):43–55. doi: 10.13023/jah.0301.05 (PMC8830599; doi:10.13023/jah.0301.05)
Supplement: Supplementary file 4 [file Appendix5-3.1.5Hudson.pptx]

## Slide 1
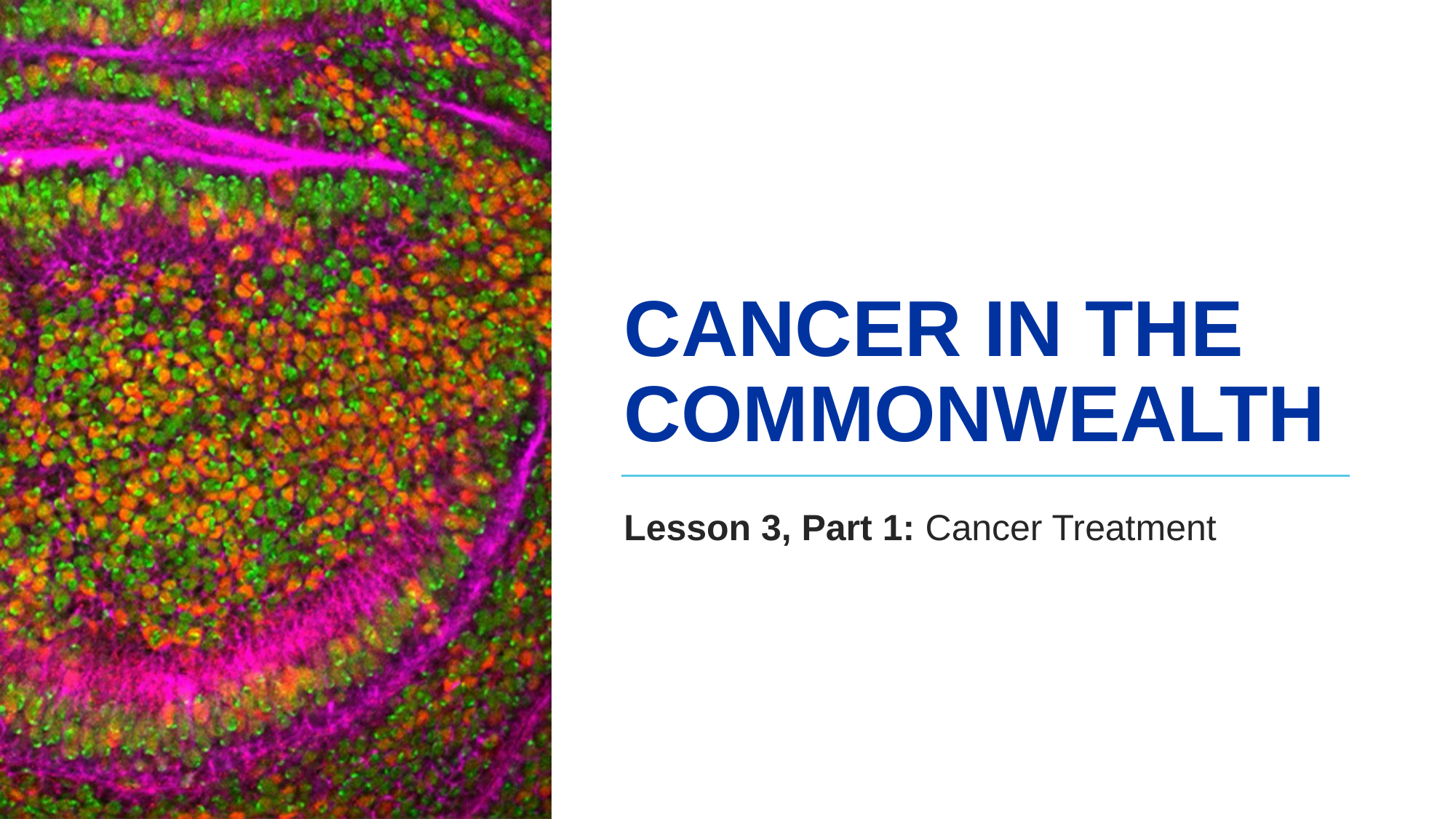

# CANCER IN THE COMMONWEALTH
Lesson 3, Part 1: Cancer Treatment

## Slide 2
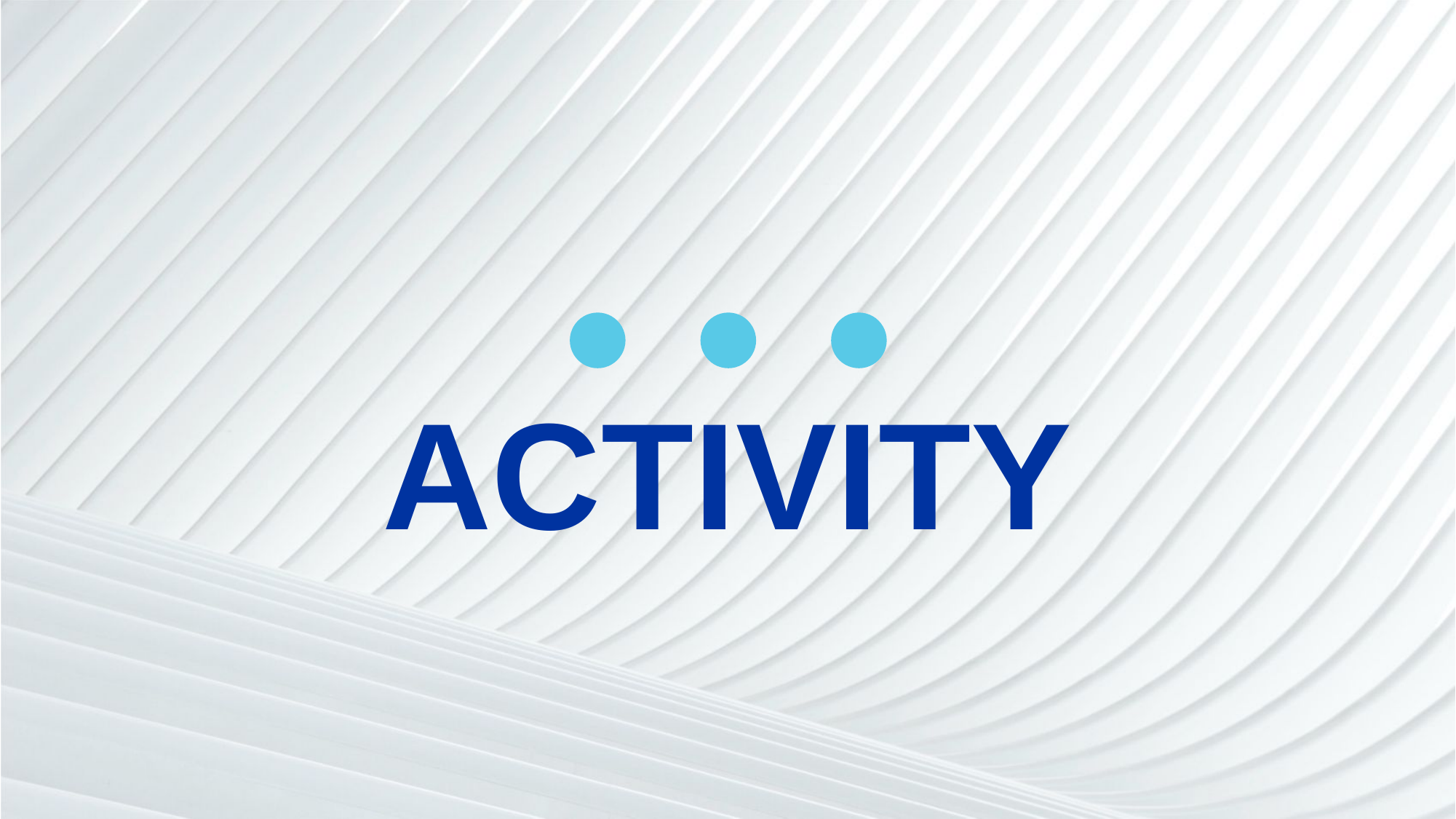

# ACTIVITY

## Slide 3
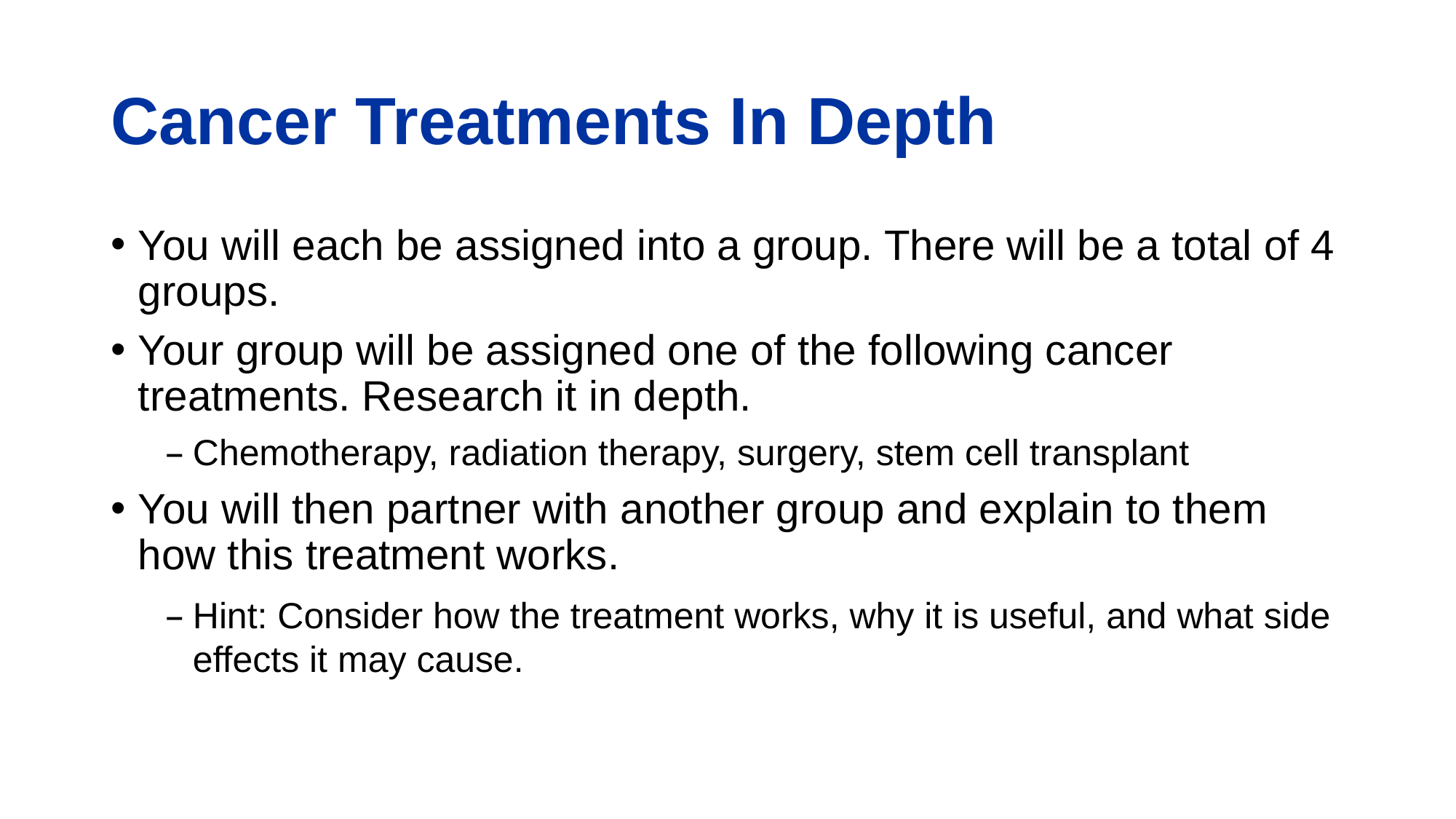

# Cancer Treatments In Depth
You will each be assigned into a group. There will be a total of 4 groups.
Your group will be assigned one of the following cancer treatments. Research it in depth.
Chemotherapy, radiation therapy, surgery, stem cell transplant
You will then partner with another group and explain to them how this treatment works.
Hint: Consider how the treatment works, why it is useful, and what side effects it may cause.

## Slide 4
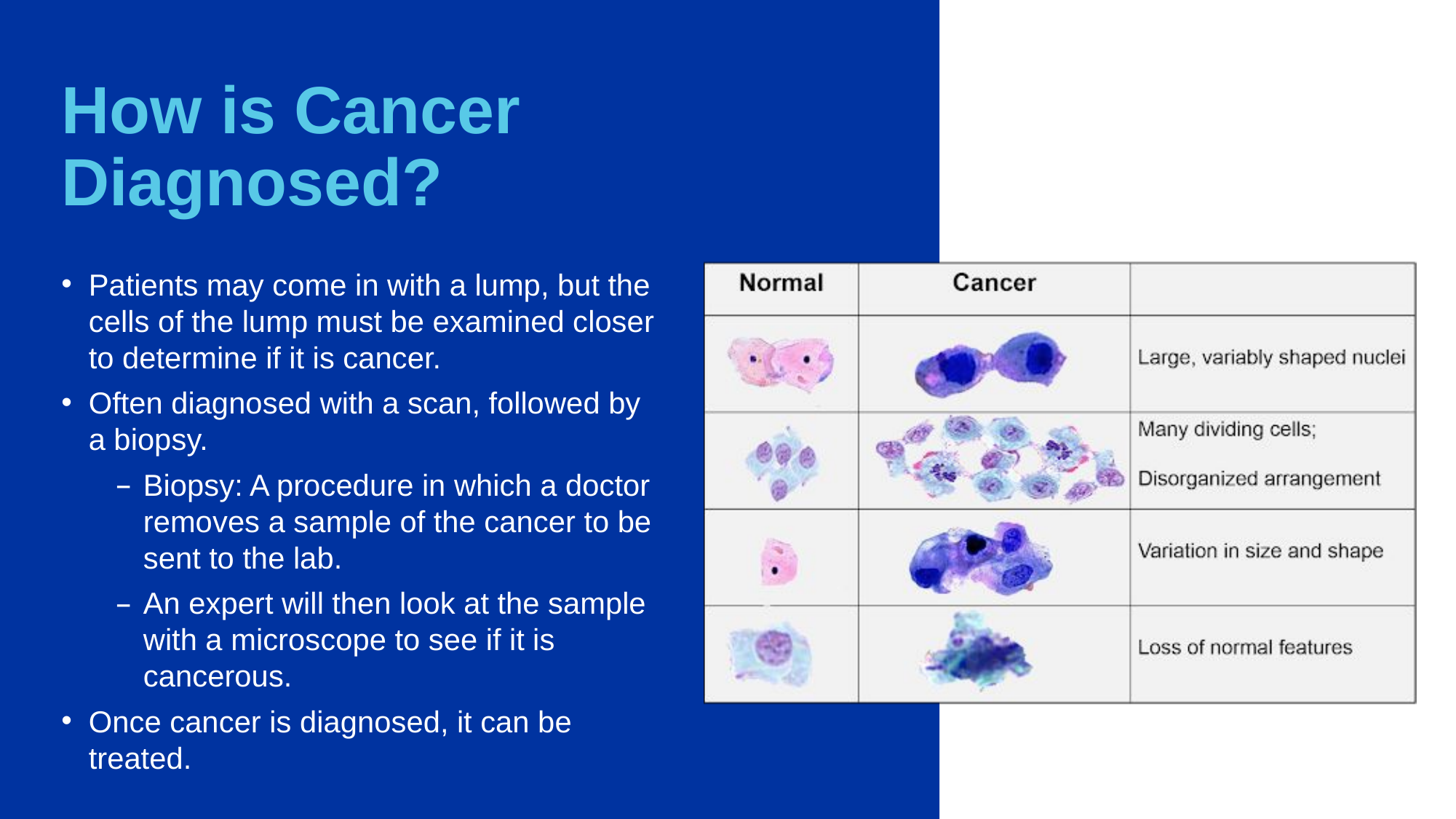

# How is Cancer Diagnosed?
Patients may come in with a lump, but the cells of the lump must be examined closer to determine if it is cancer.
Often diagnosed with a scan, followed by a biopsy.
Biopsy: A procedure in which a doctor removes a sample of the cancer to be sent to the lab.
An expert will then look at the sample with a microscope to see if it is cancerous.
Once cancer is diagnosed, it can be treated.

## Slide 5
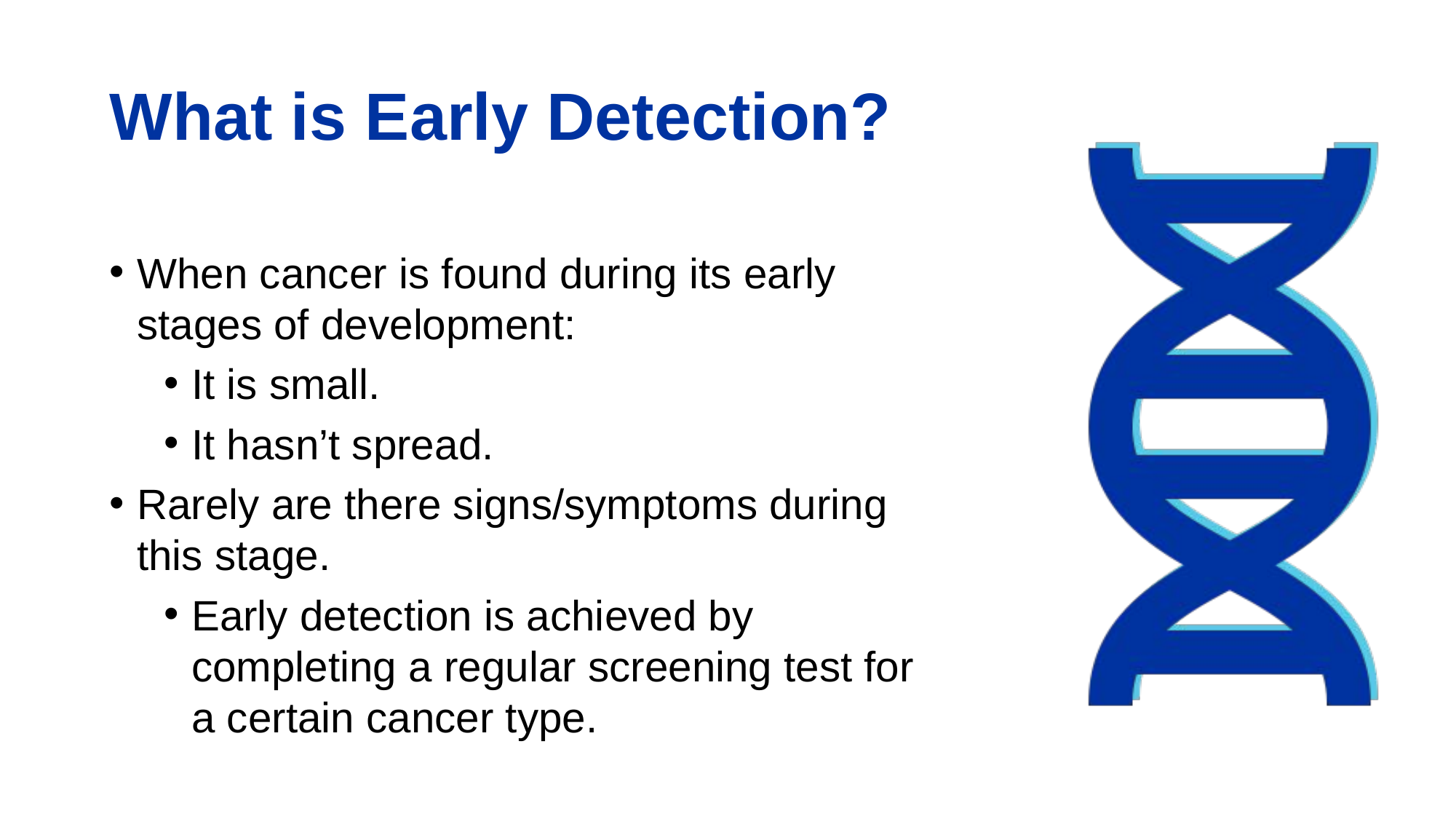

# What is Early Detection?
When cancer is found during its early stages of development:
It is small.
It hasn’t spread.
Rarely are there signs/symptoms during this stage.
Early detection is achieved by completing a regular screening test for a certain cancer type.

## Slide 6
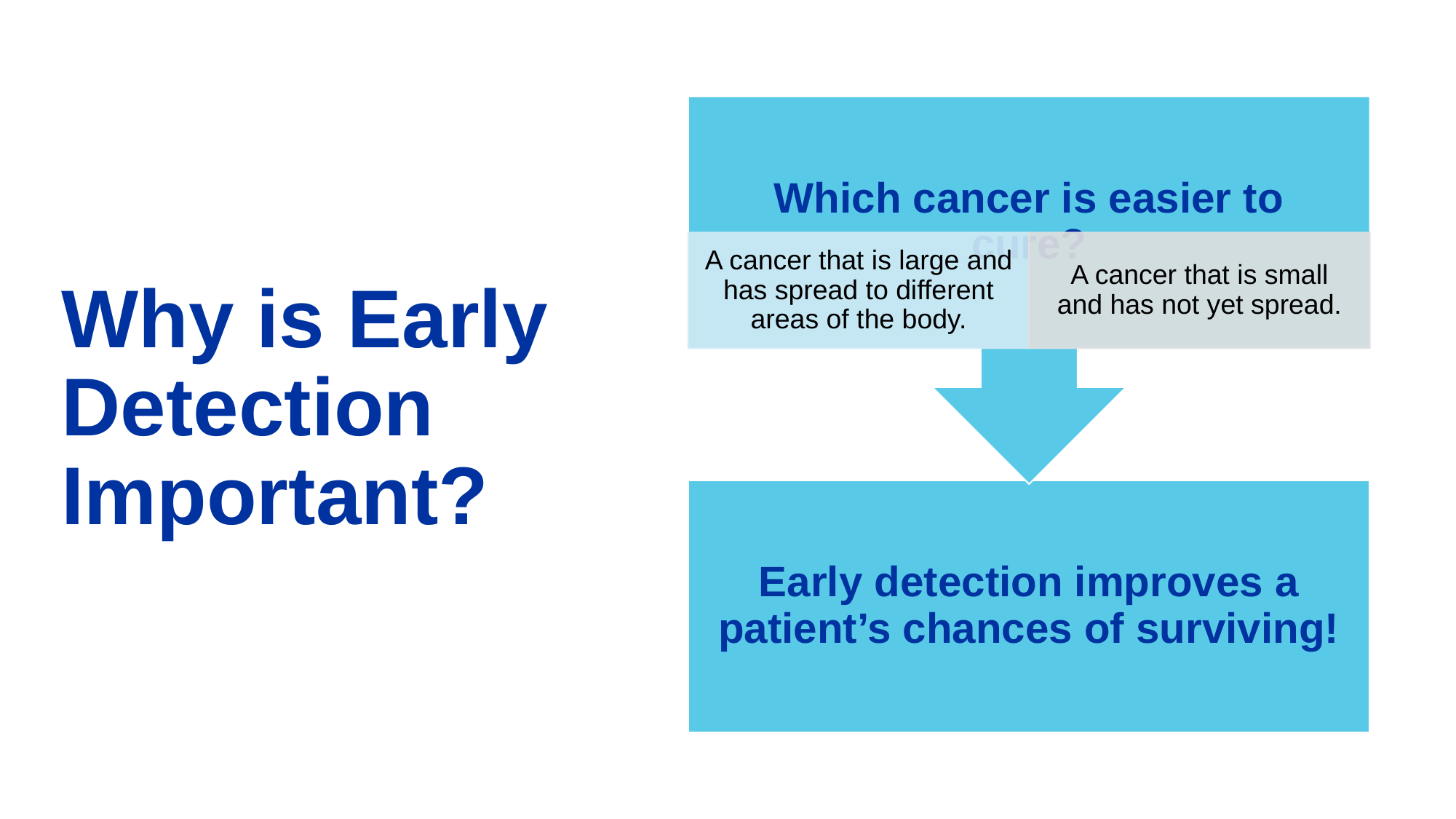

# Why is Early Detection Important?

## Slide 7
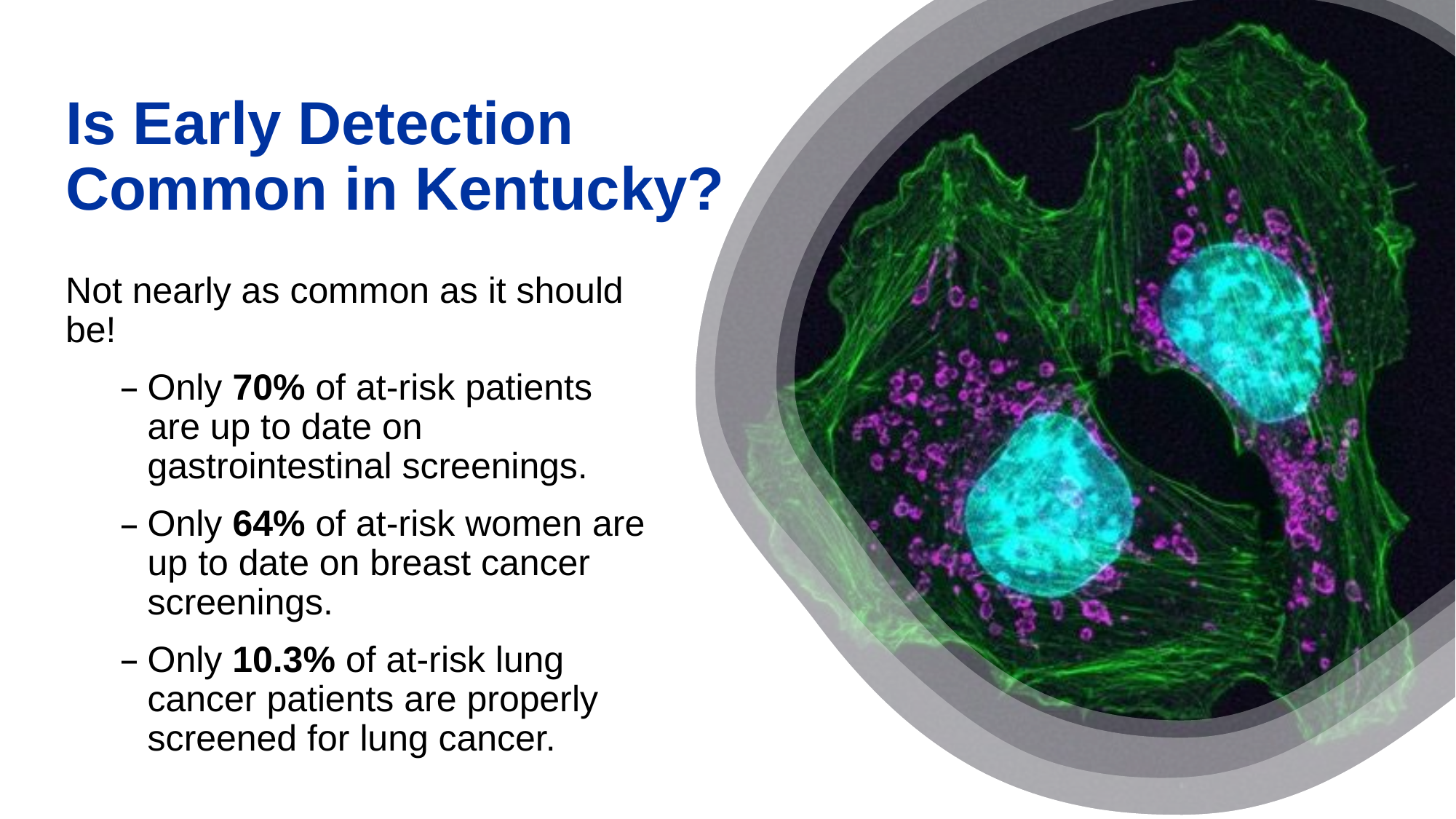

# Is Early Detection Common in Kentucky?
Not nearly as common as it should be!
Only 70% of at-risk patients are up to date on gastrointestinal screenings.
Only 64% of at-risk women are up to date on breast cancer screenings.
Only 10.3% of at-risk lung cancer patients are properly screened for lung cancer.

## Slide 8
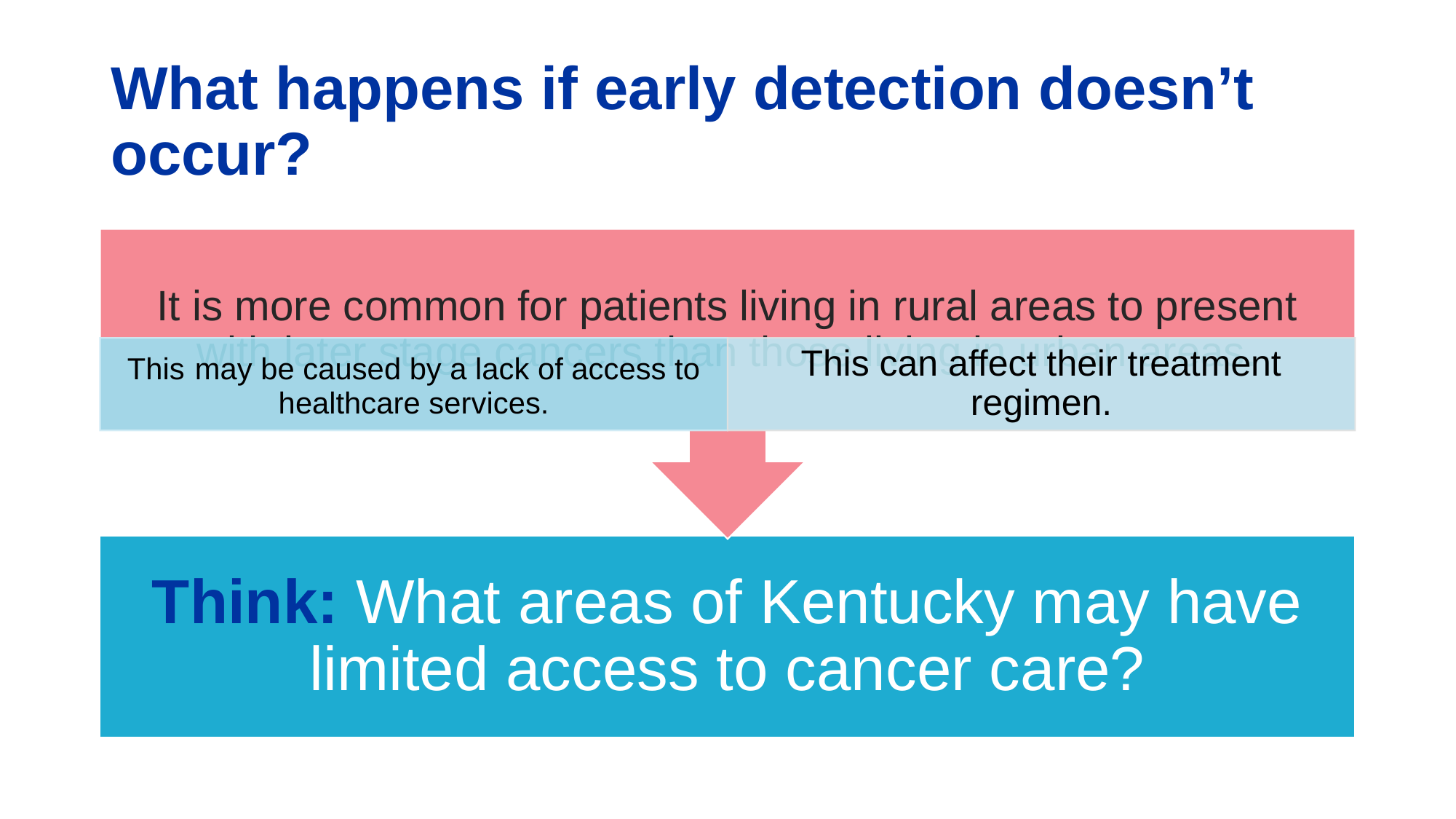

# What happens if early detection doesn’t occur?

## Slide 9
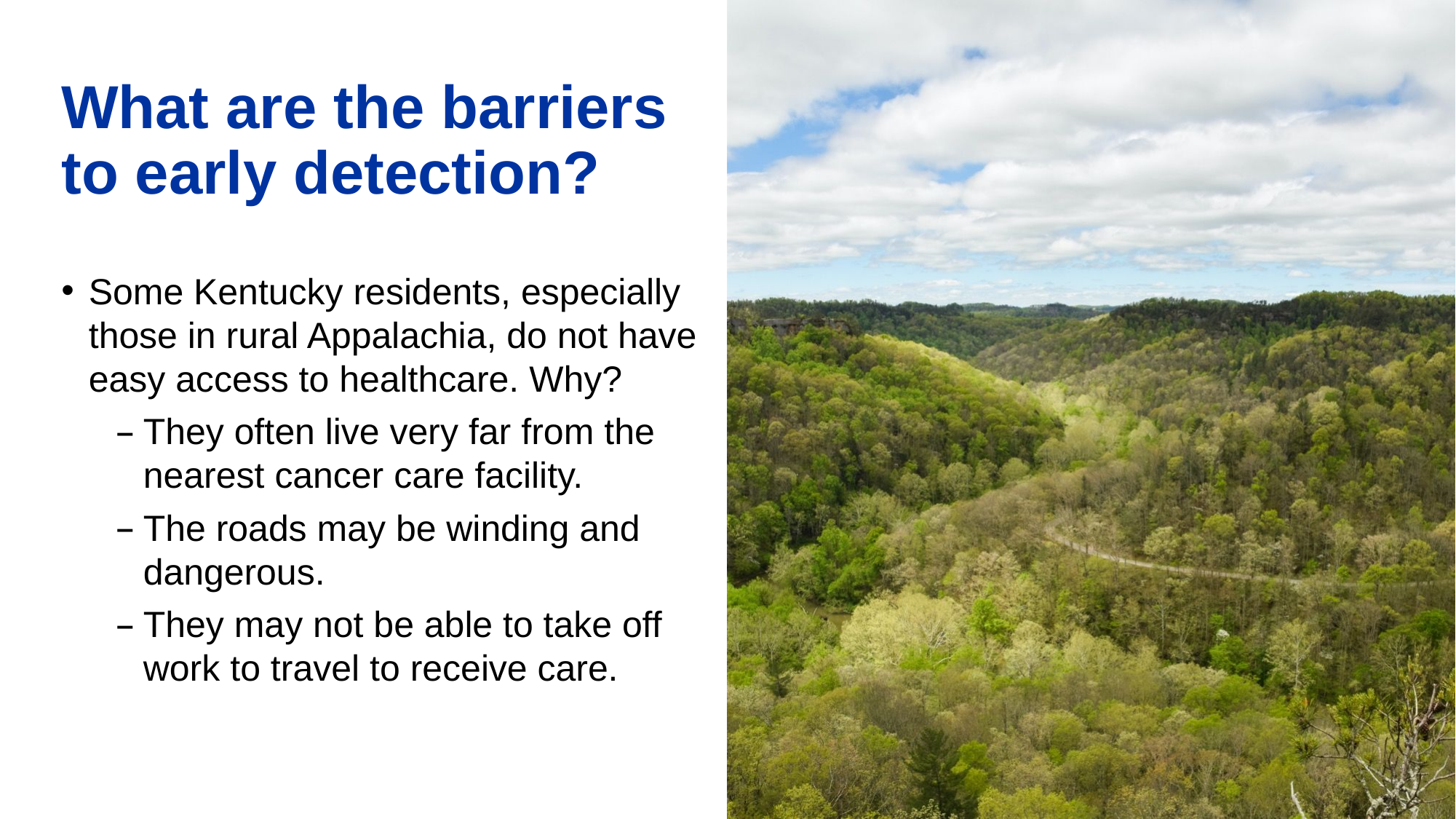

# What are the barriers to early detection?
Some Kentucky residents, especially those in rural Appalachia, do not have easy access to healthcare. Why?
They often live very far from the nearest cancer care facility.
The roads may be winding and dangerous.
They may not be able to take off work to travel to receive care.

## Slide 10
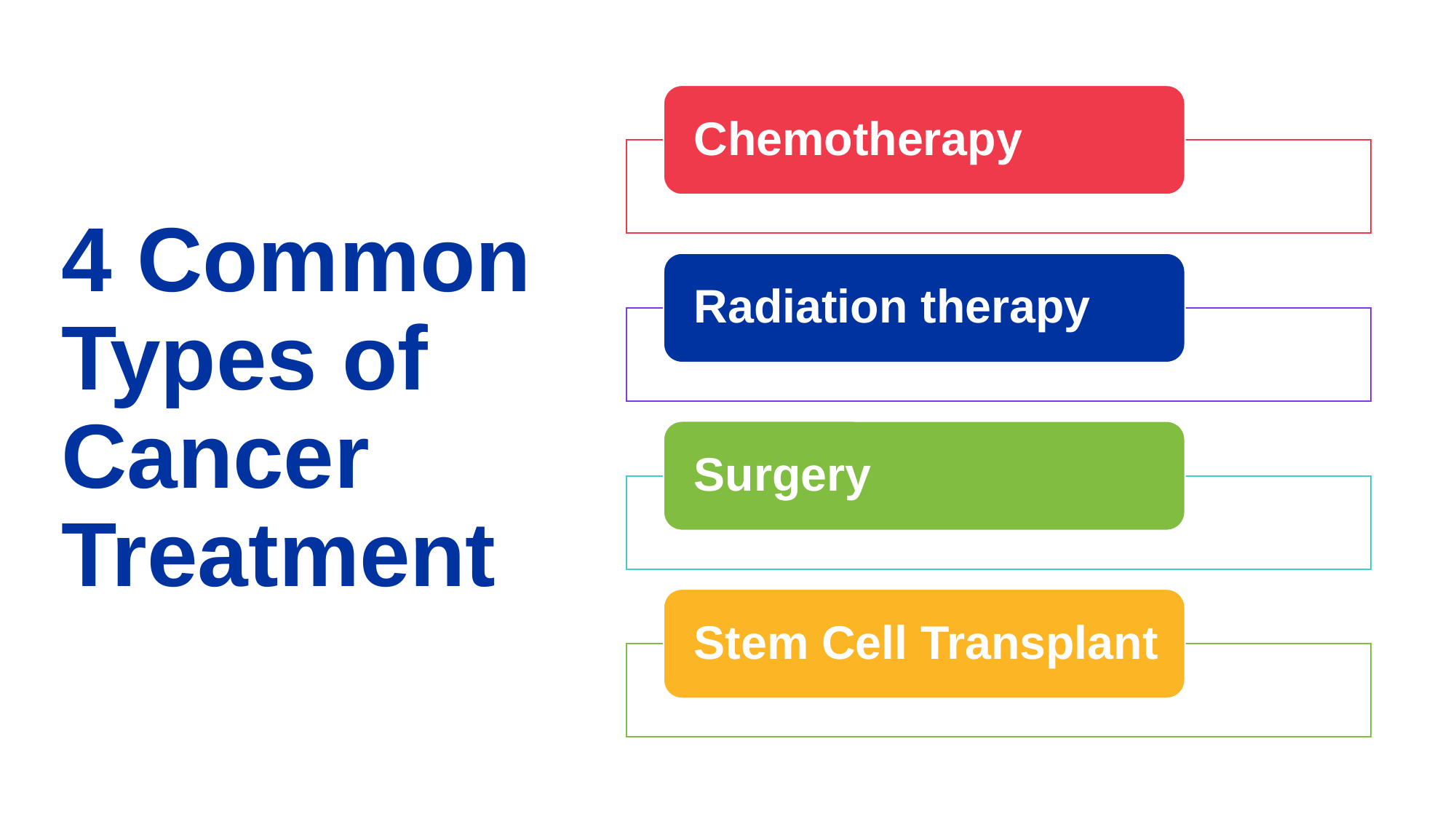

# 4 Common Types of Cancer Treatment

## Slide 11
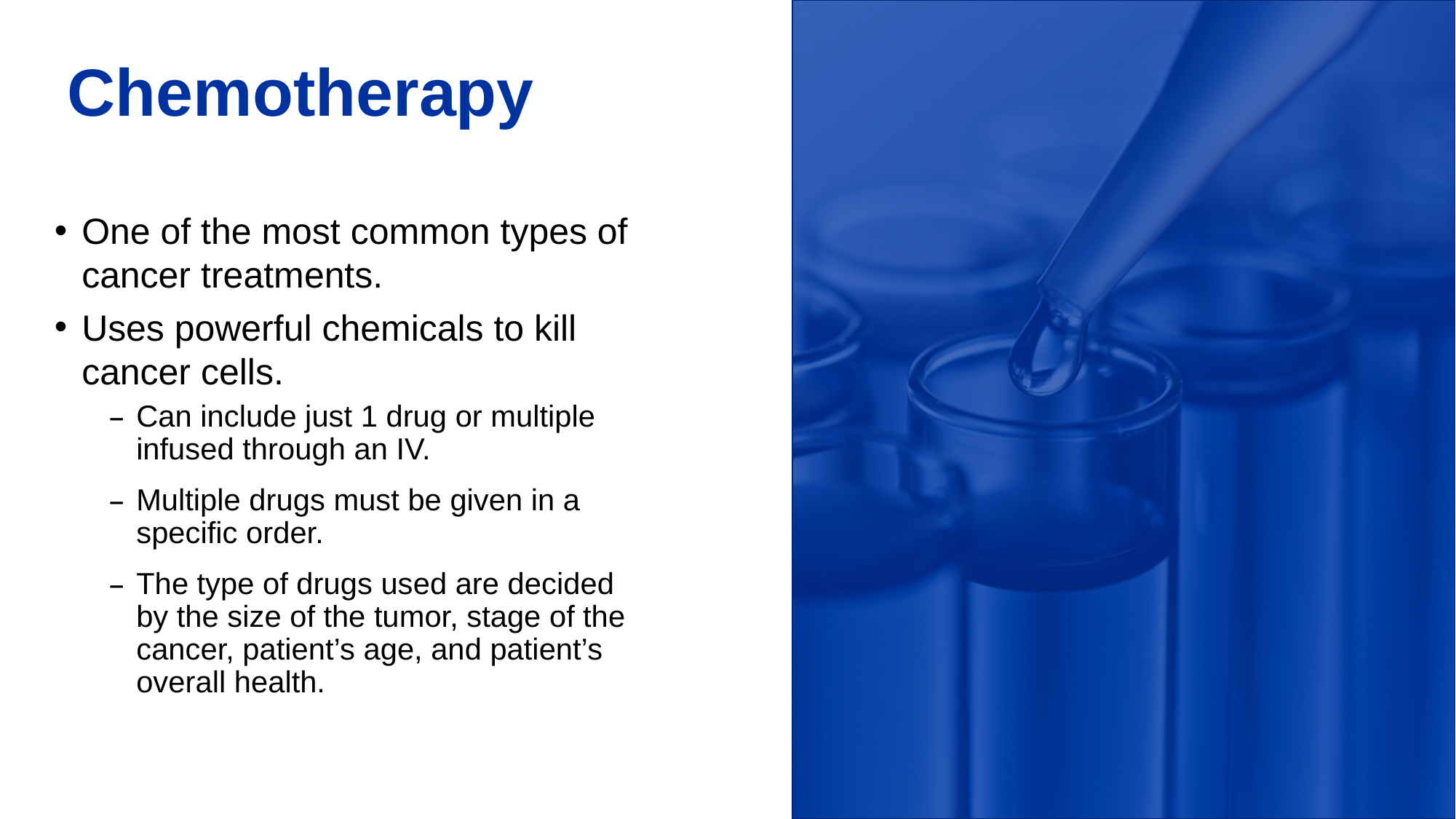

# Chemotherapy
One of the most common types of cancer treatments.
Uses powerful chemicals to kill cancer cells.
Can include just 1 drug or multiple infused through an IV.
Multiple drugs must be given in a specific order.
The type of drugs used are decided by the size of the tumor, stage of the cancer, patient’s age, and patient’s overall health.

## Slide 12
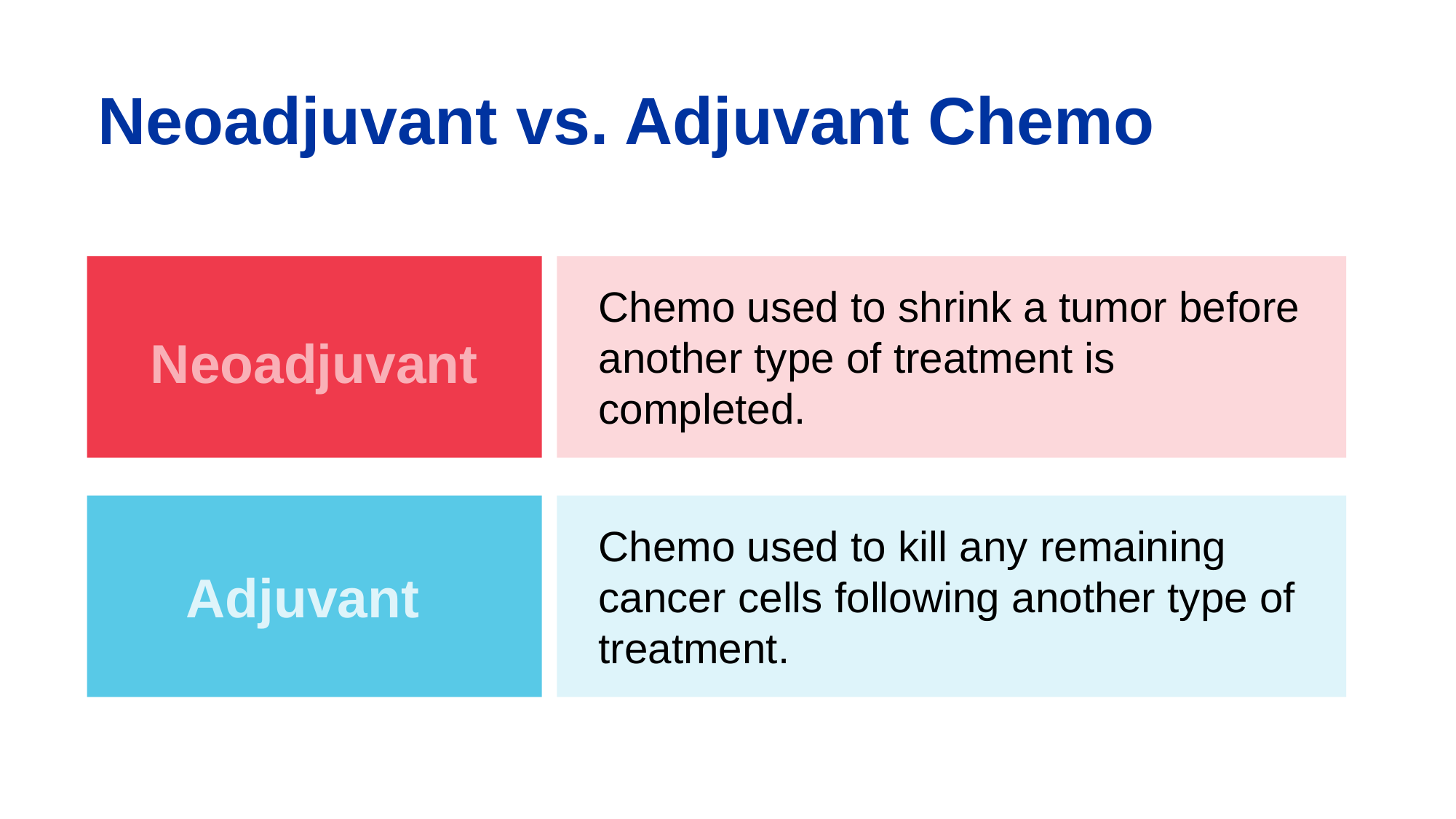

# Neoadjuvant vs. Adjuvant Chemo
Chemo used to shrink a tumor before another type of treatment is completed.
Neoadjuvant
Chemo used to kill any remaining cancer cells following another type of treatment.
Adjuvant

## Slide 13
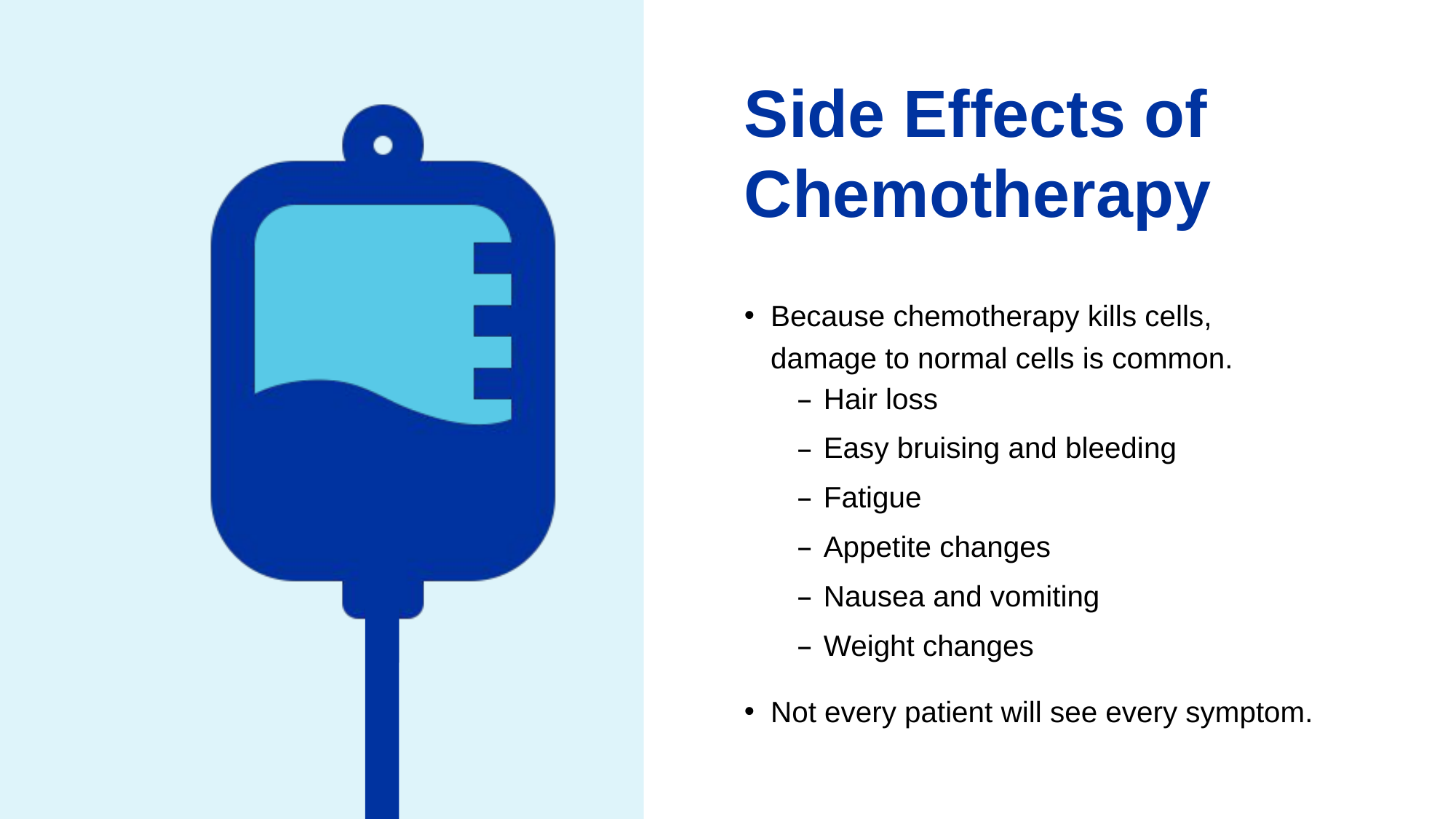

# Side Effects of Chemotherapy
Because chemotherapy kills cells, damage to normal cells is common.
Hair loss
Easy bruising and bleeding
Fatigue
Appetite changes
Nausea and vomiting
Weight changes
Not every patient will see every symptom.

## Slide 14
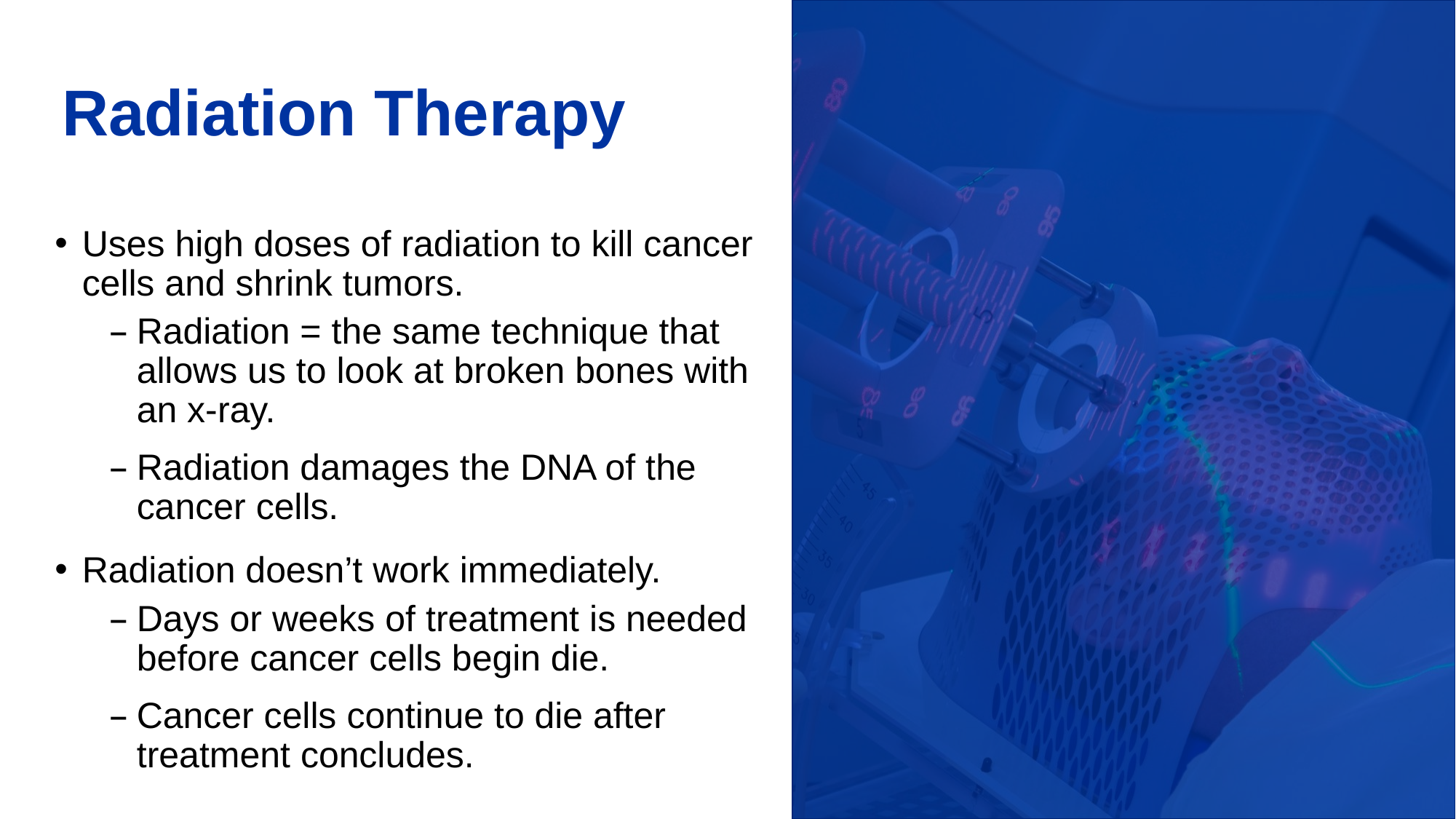

# Radiation Therapy
Uses high doses of radiation to kill cancer cells and shrink tumors.
Radiation = the same technique that allows us to look at broken bones with an x-ray.
Radiation damages the DNA of the cancer cells.
Radiation doesn’t work immediately.
Days or weeks of treatment is needed before cancer cells begin die.
Cancer cells continue to die after treatment concludes.

## Slide 15
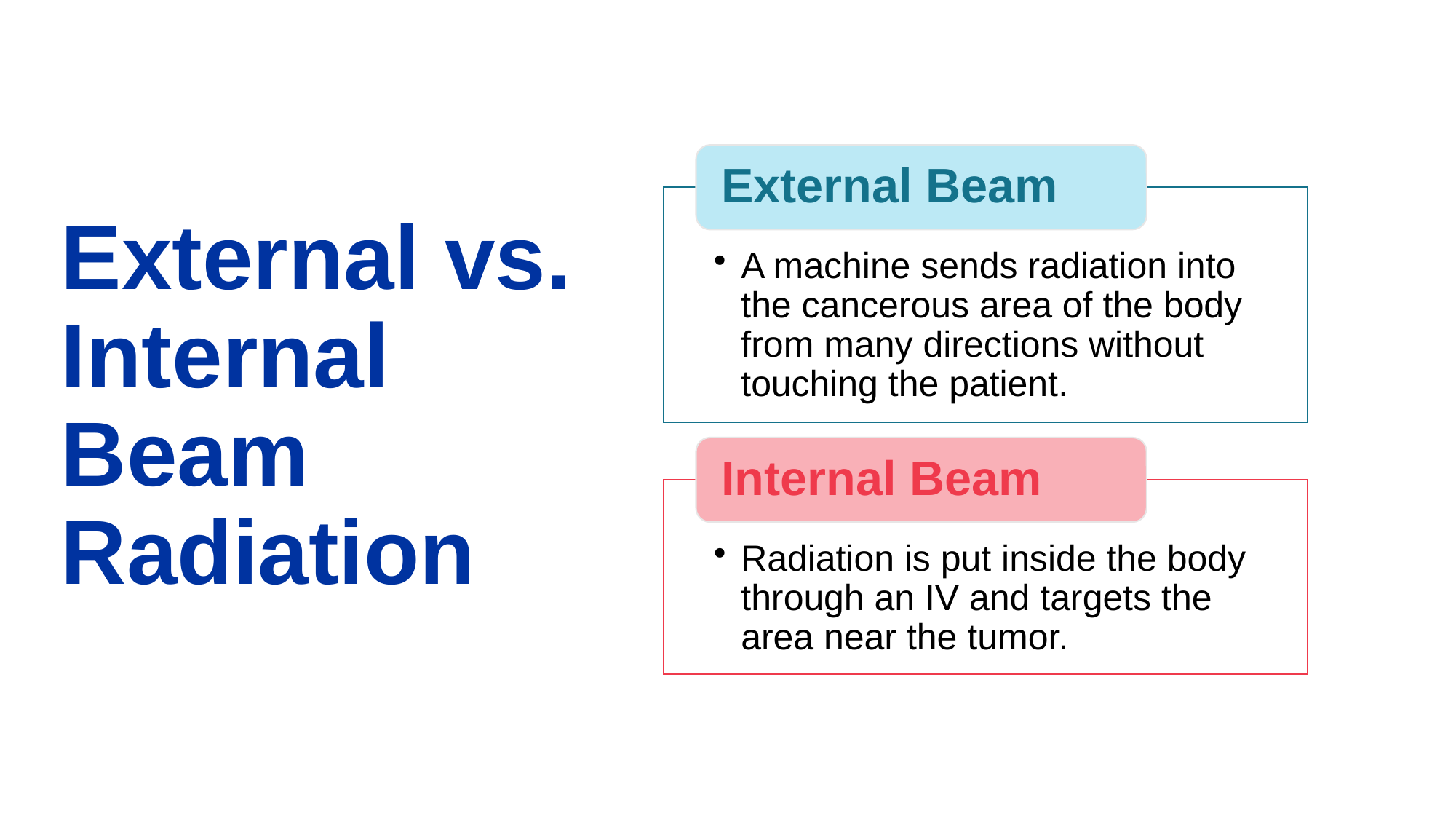

# External vs. Internal Beam Radiation

## Slide 16
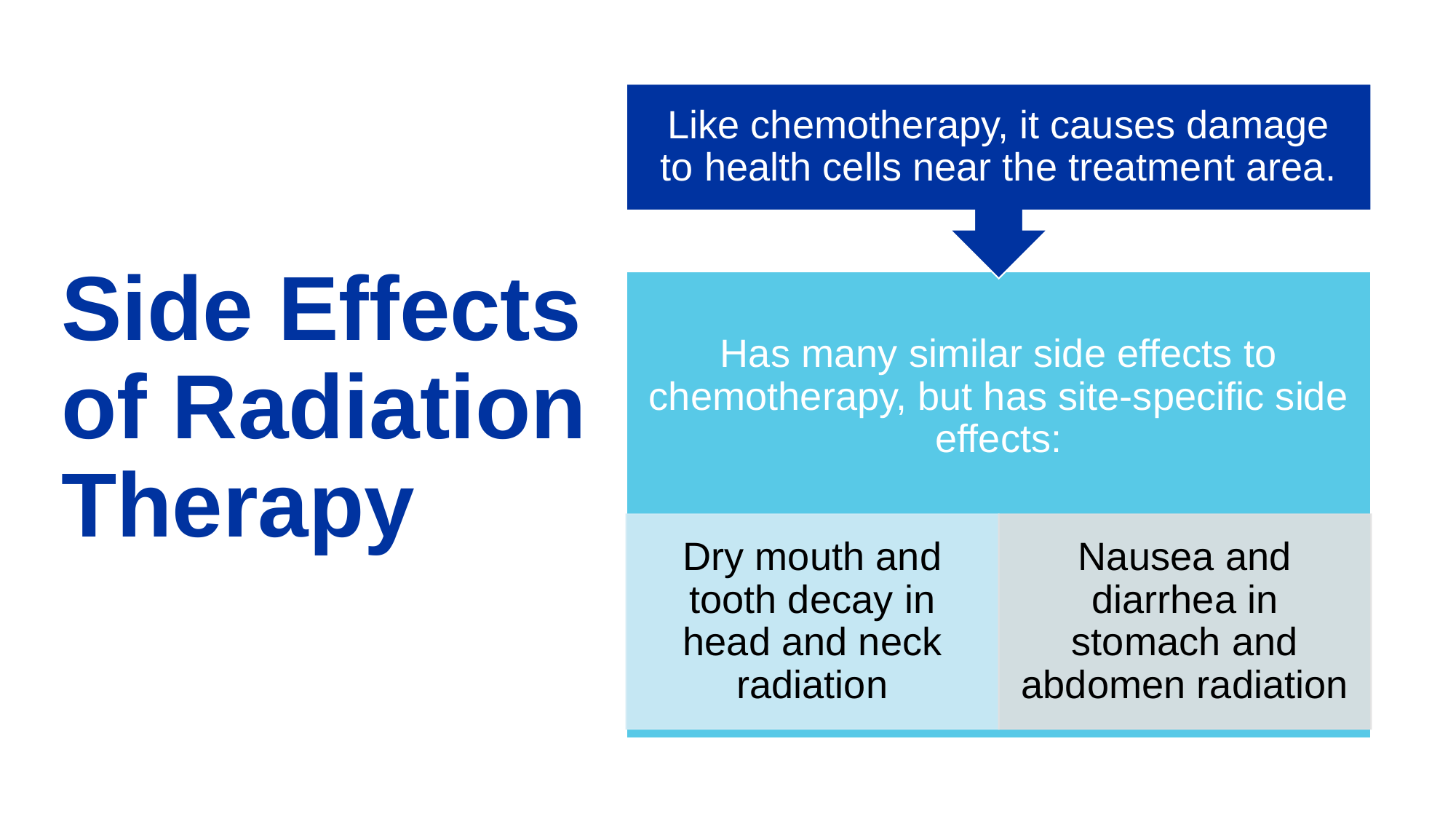

# Side Effects of Radiation Therapy

## Slide 17
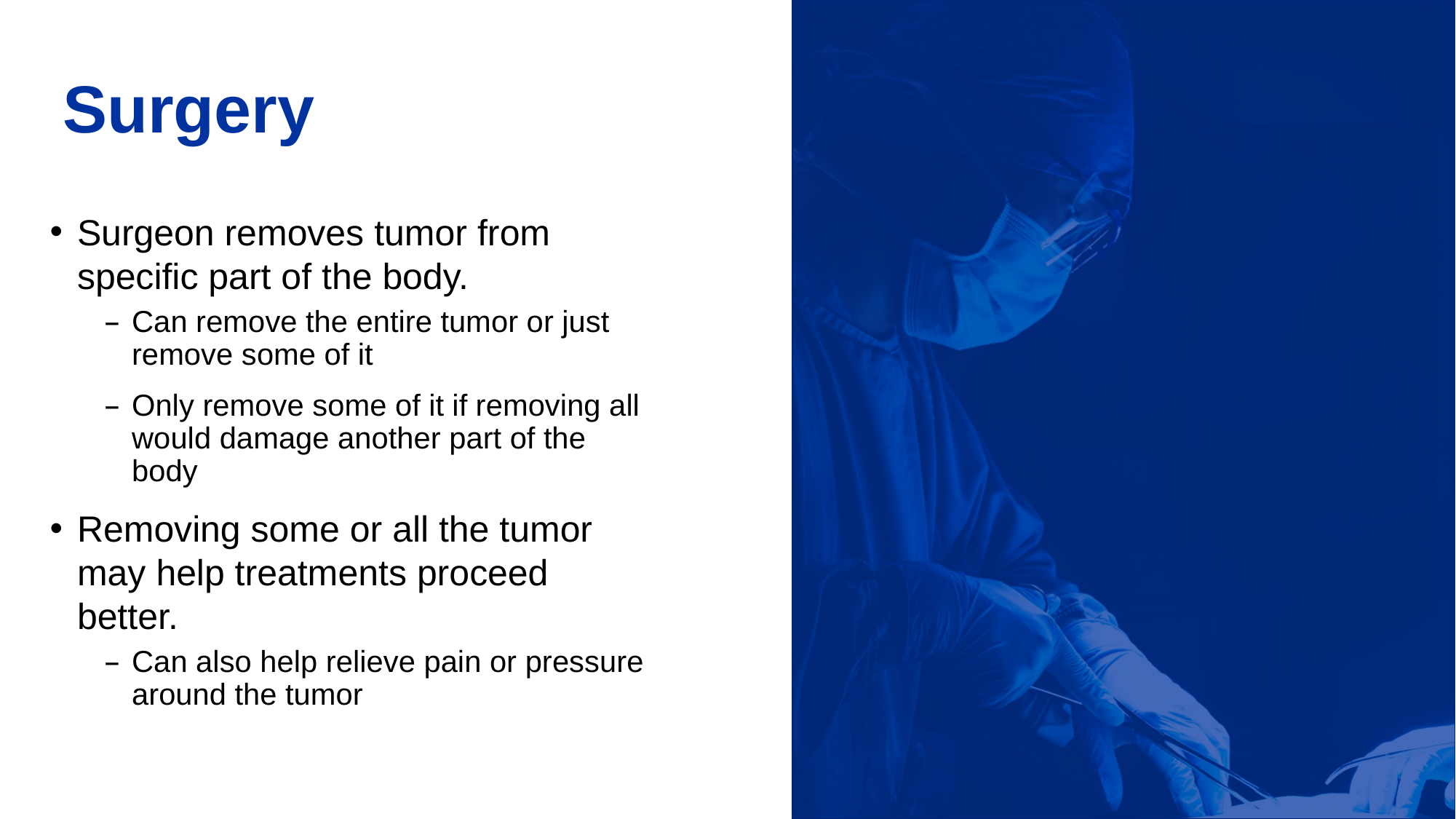

# Surgery
Surgeon removes tumor from specific part of the body.
Can remove the entire tumor or just remove some of it
Only remove some of it if removing all would damage another part of the body
Removing some or all the tumor may help treatments proceed better.
Can also help relieve pain or pressure around the tumor

## Slide 18
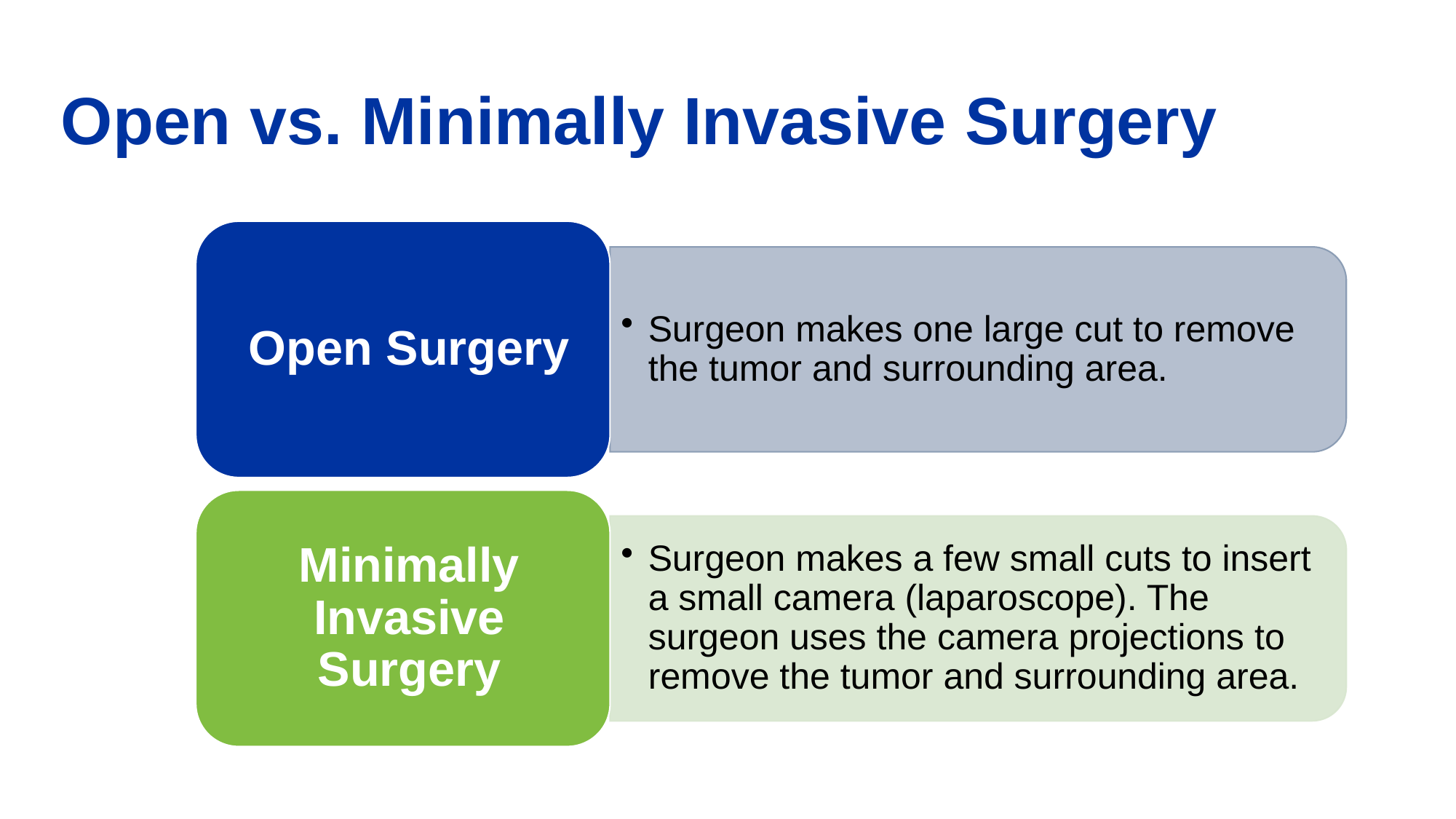

# Open vs. Minimally Invasive Surgery

## Slide 19
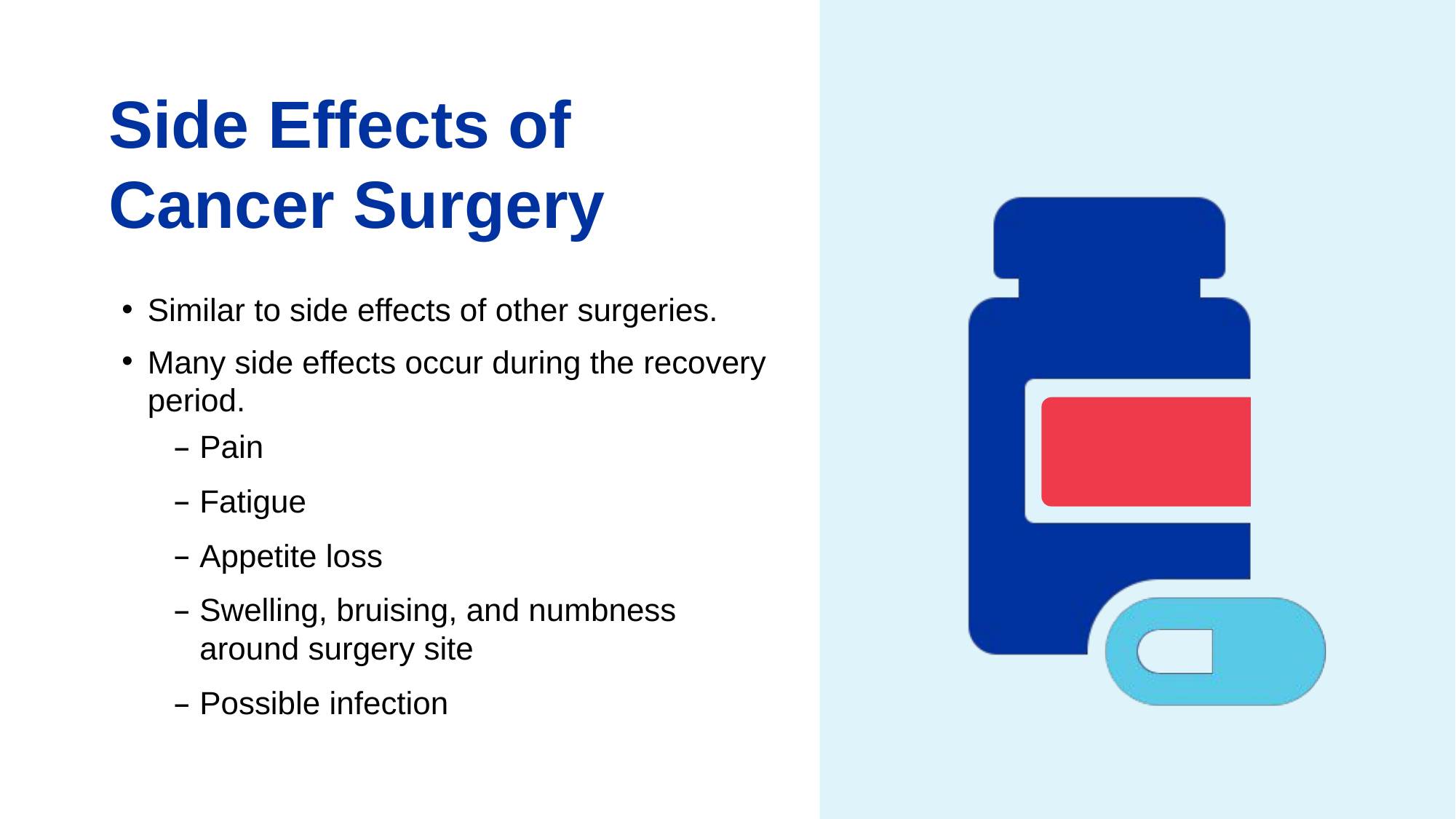

# Side Effects of Cancer Surgery
Similar to side effects of other surgeries.
Many side effects occur during the recovery period.
Pain
Fatigue
Appetite loss
Swelling, bruising, and numbness around surgery site
Possible infection

## Slide 20
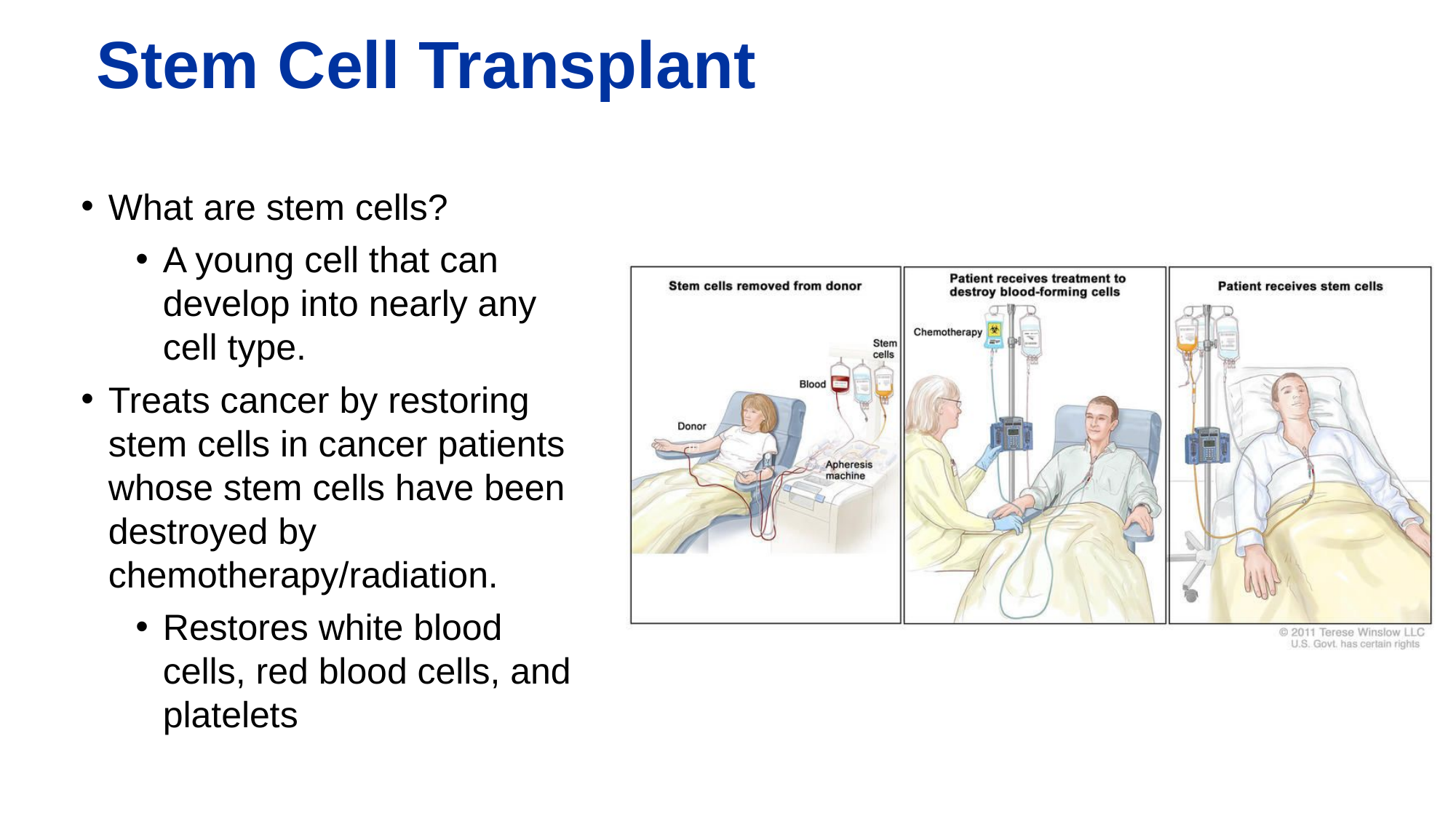

# Stem Cell Transplant
What are stem cells?
A young cell that can develop into nearly any cell type.
Treats cancer by restoring stem cells in cancer patients whose stem cells have been destroyed by chemotherapy/radiation.
Restores white blood cells, red blood cells, and platelets

## Slide 21
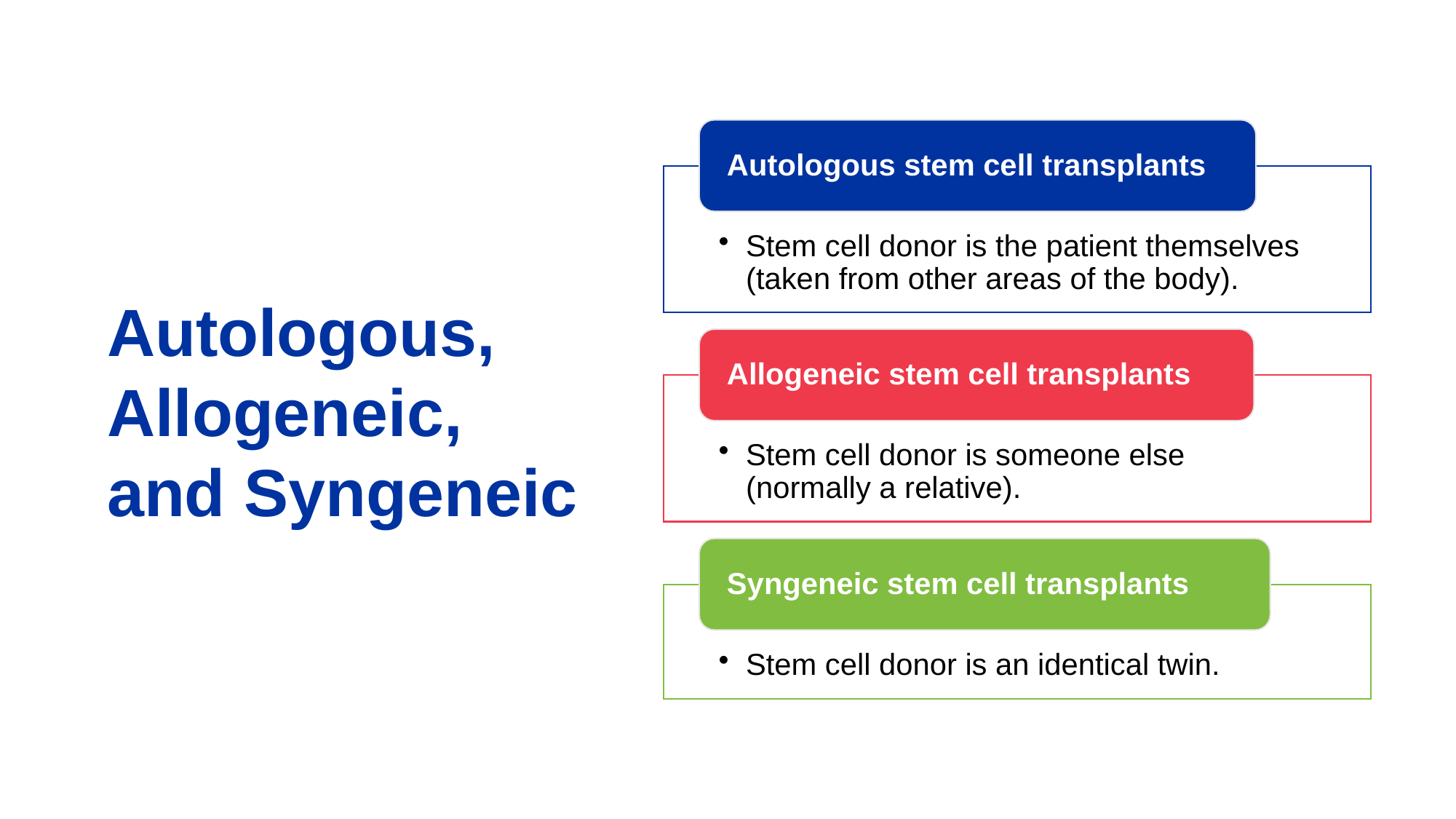

# Autologous, Allogeneic, and Syngeneic

## Slide 22
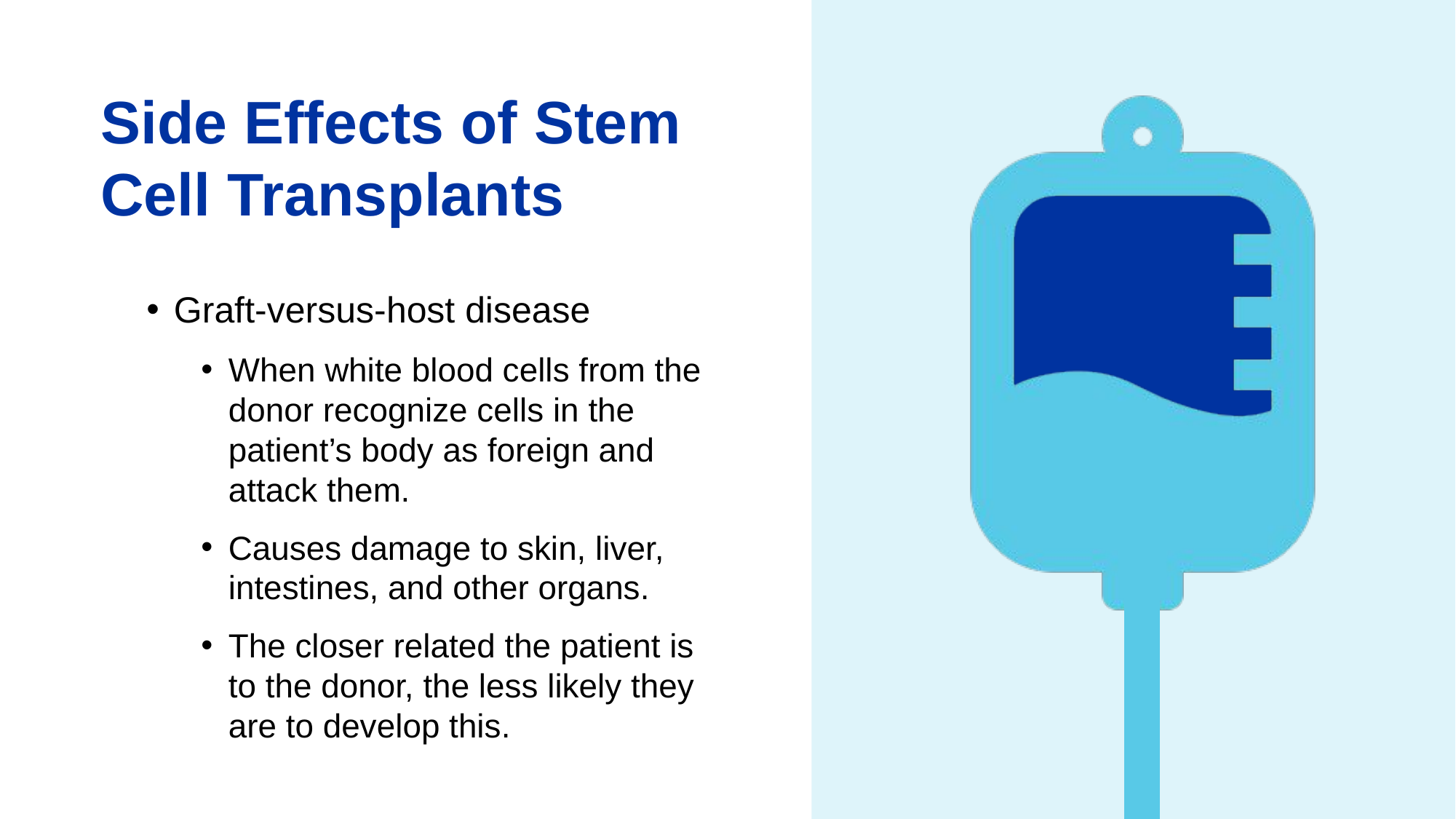

# Side Effects of Stem Cell Transplants
Graft-versus-host disease
When white blood cells from the donor recognize cells in the patient’s body as foreign and attack them.
Causes damage to skin, liver, intestines, and other organs.
The closer related the patient is to the donor, the less likely they are to develop this.

## Slide 23
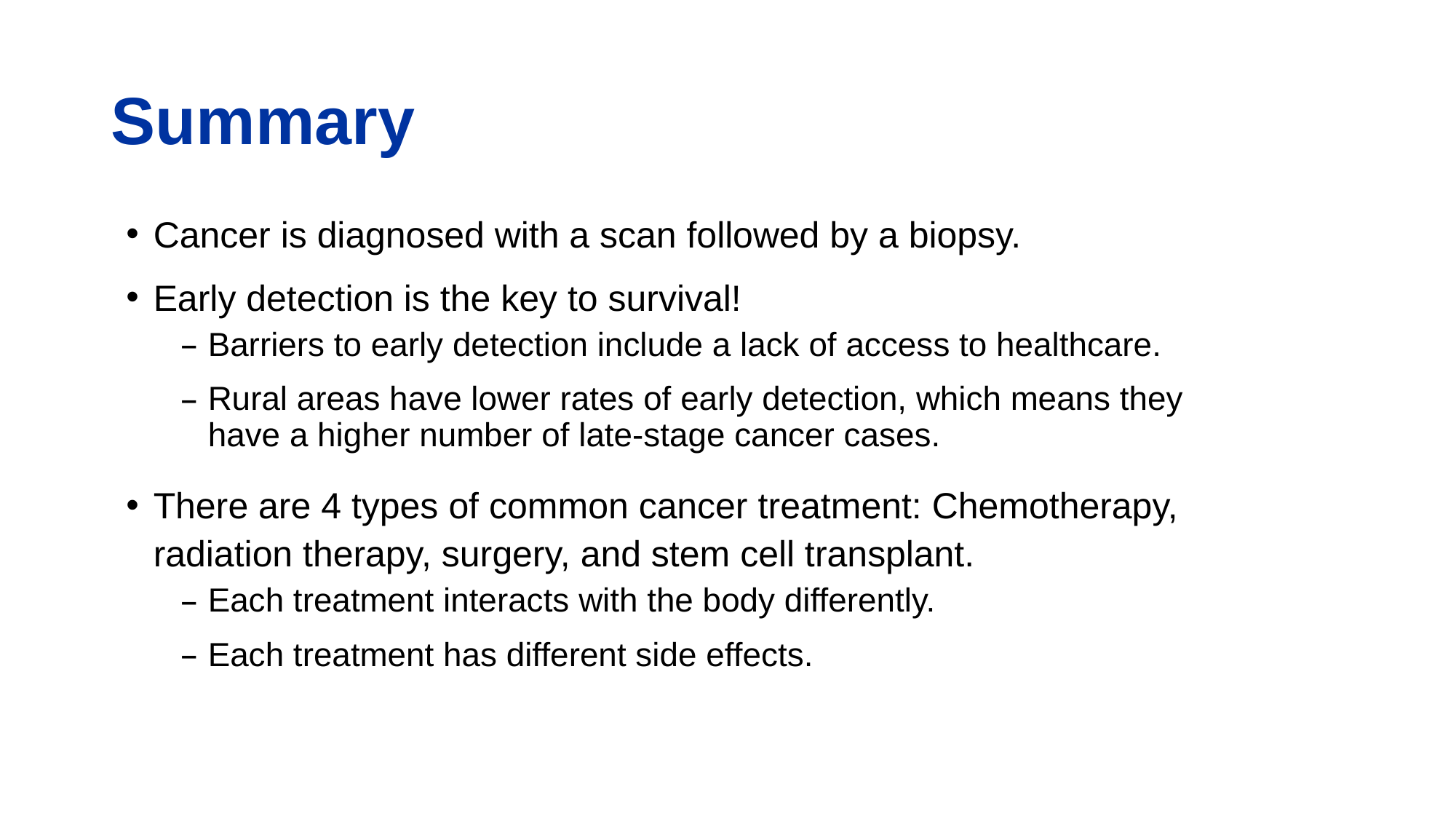

# Summary
Cancer is diagnosed with a scan followed by a biopsy.
Early detection is the key to survival!
Barriers to early detection include a lack of access to healthcare.
Rural areas have lower rates of early detection, which means they have a higher number of late-stage cancer cases.
There are 4 types of common cancer treatment: Chemotherapy, radiation therapy, surgery, and stem cell transplant.
Each treatment interacts with the body differently.
Each treatment has different side effects.

## Slide 24
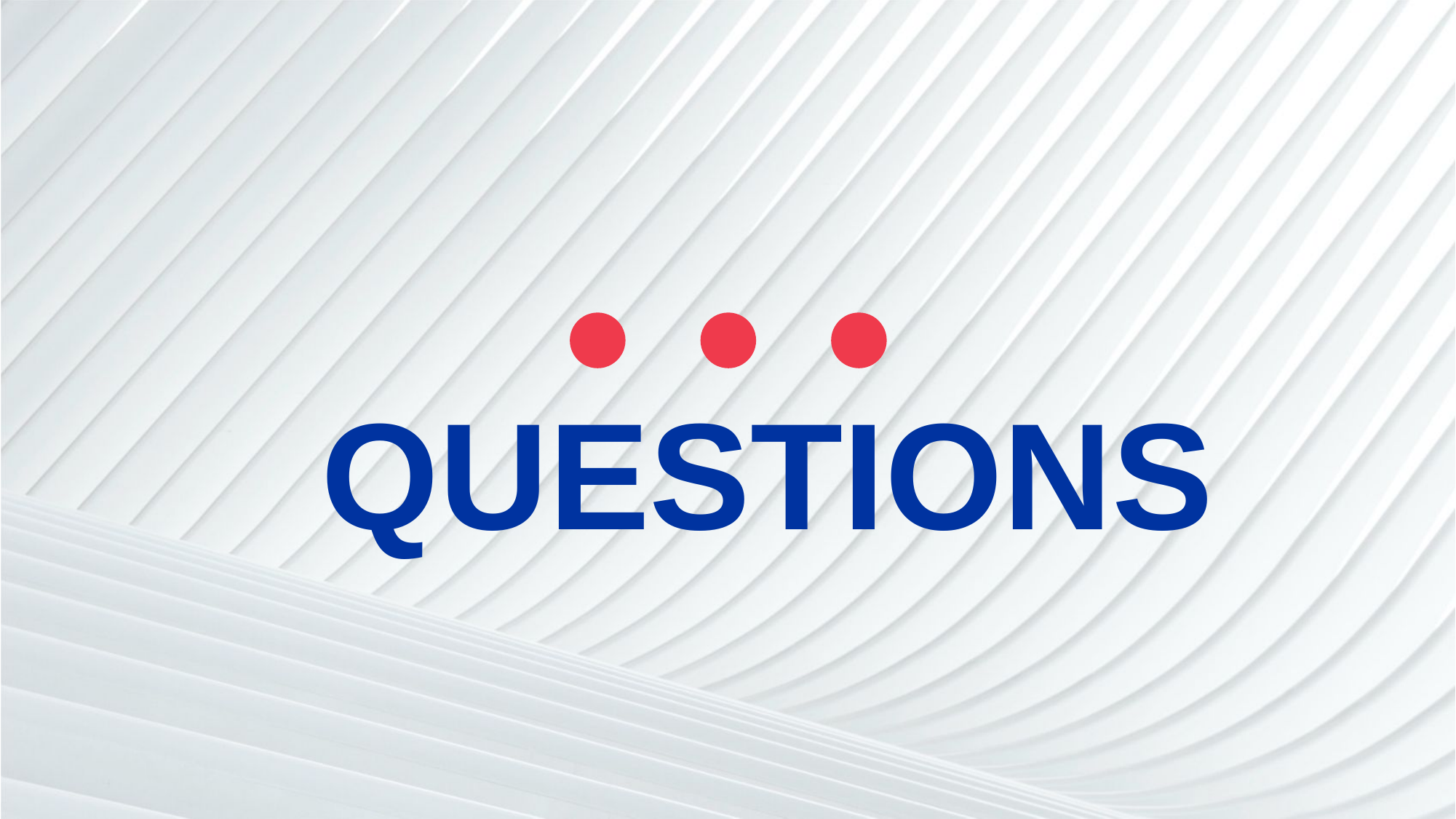

# QUESTIONS
