# Supplementary material for: Cancer Curriculum for Appalachian Kentucky Middle and High Schools
Source: J Appalach Health. 2021 Jan 24;3(1):43–55. doi: 10.13023/jah.0301.05 (PMC8830599; doi:10.13023/jah.0301.05)
Supplement: Supplementary file 5 [file Appendix6-3.1.5Hudson.pptx]

## Slide 1
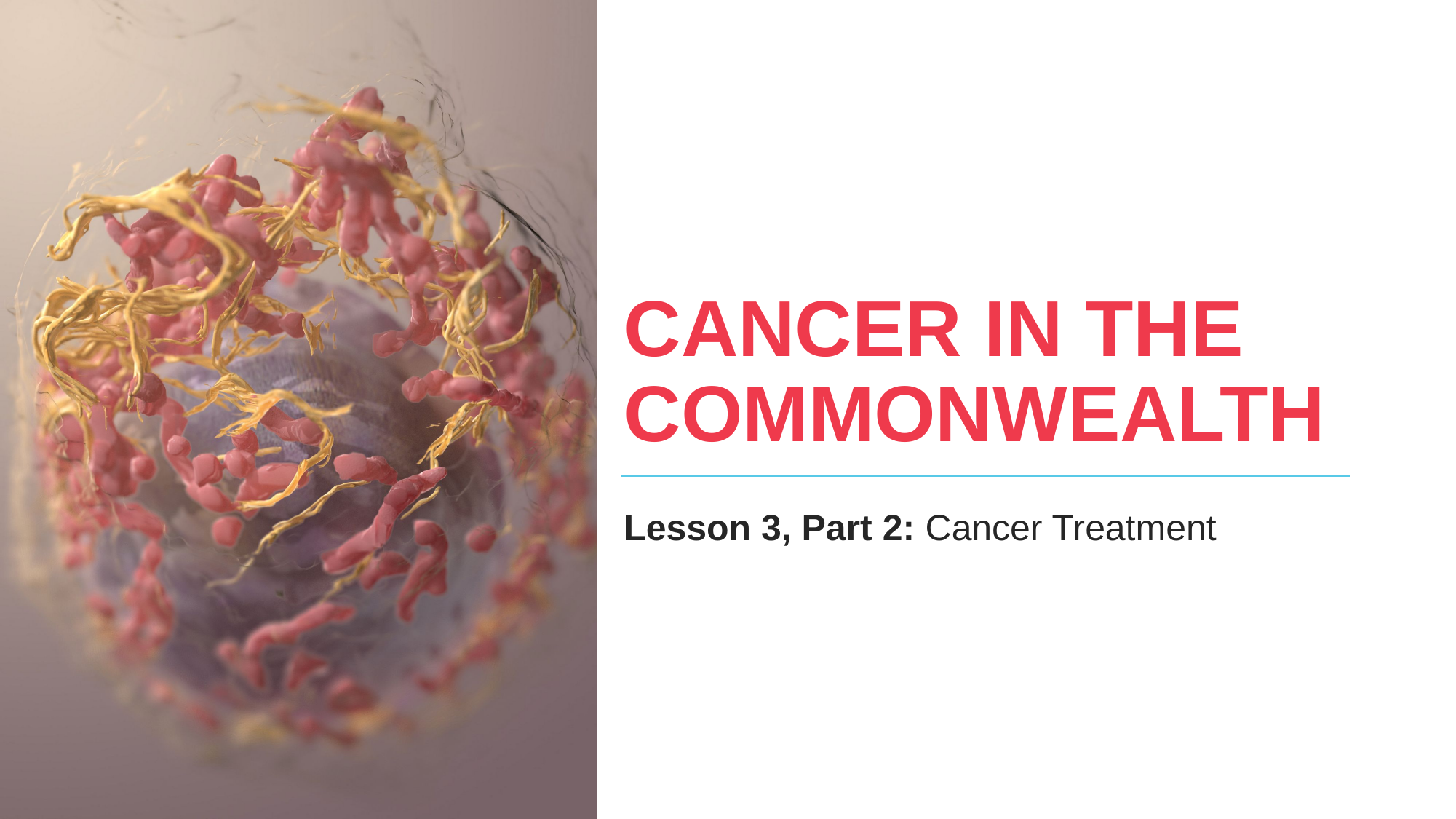

# CANCER IN THE COMMONWEALTH
Lesson 3, Part 2: Cancer Treatment

## Slide 2
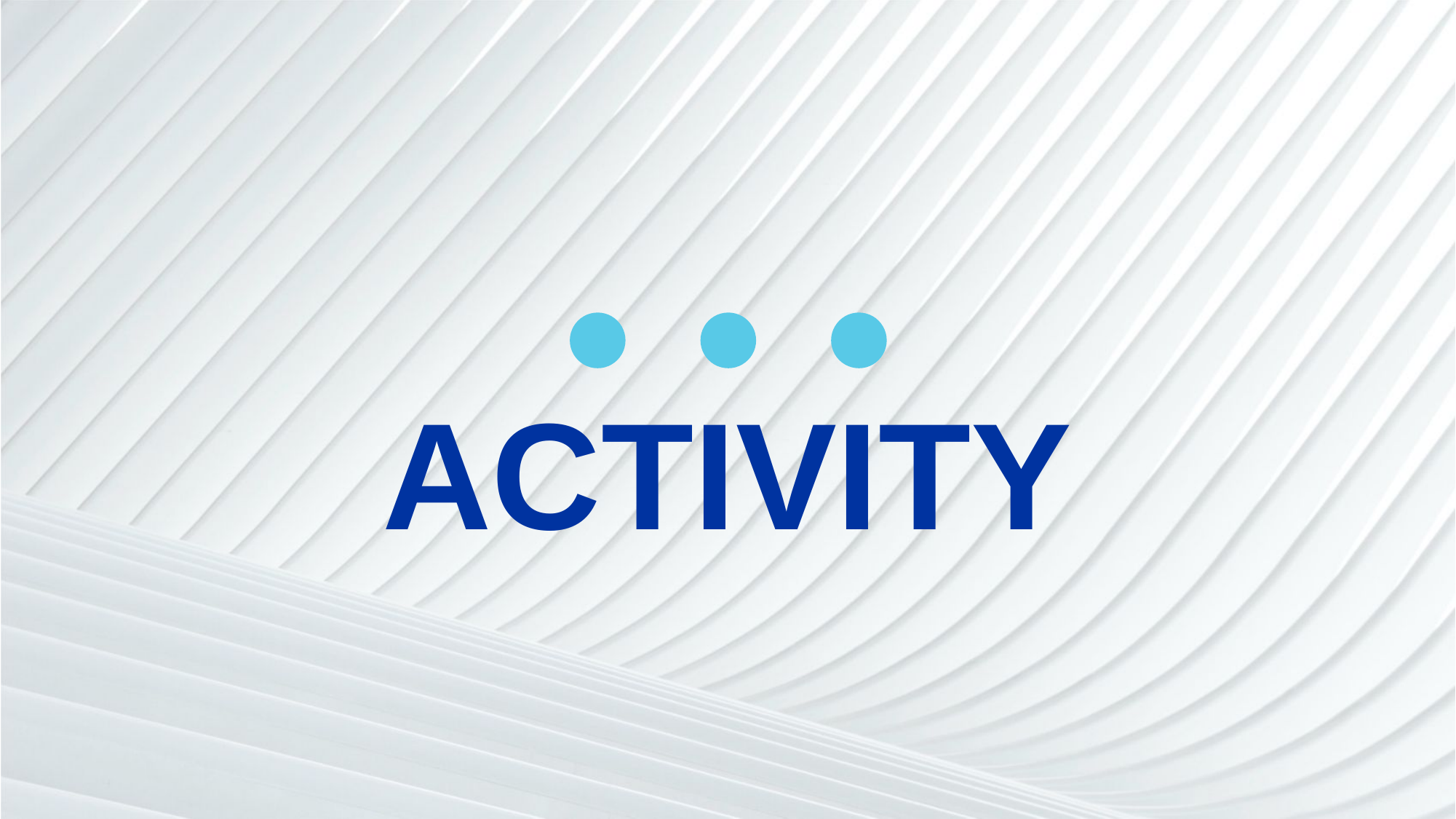

# ACTIVITY

## Slide 3
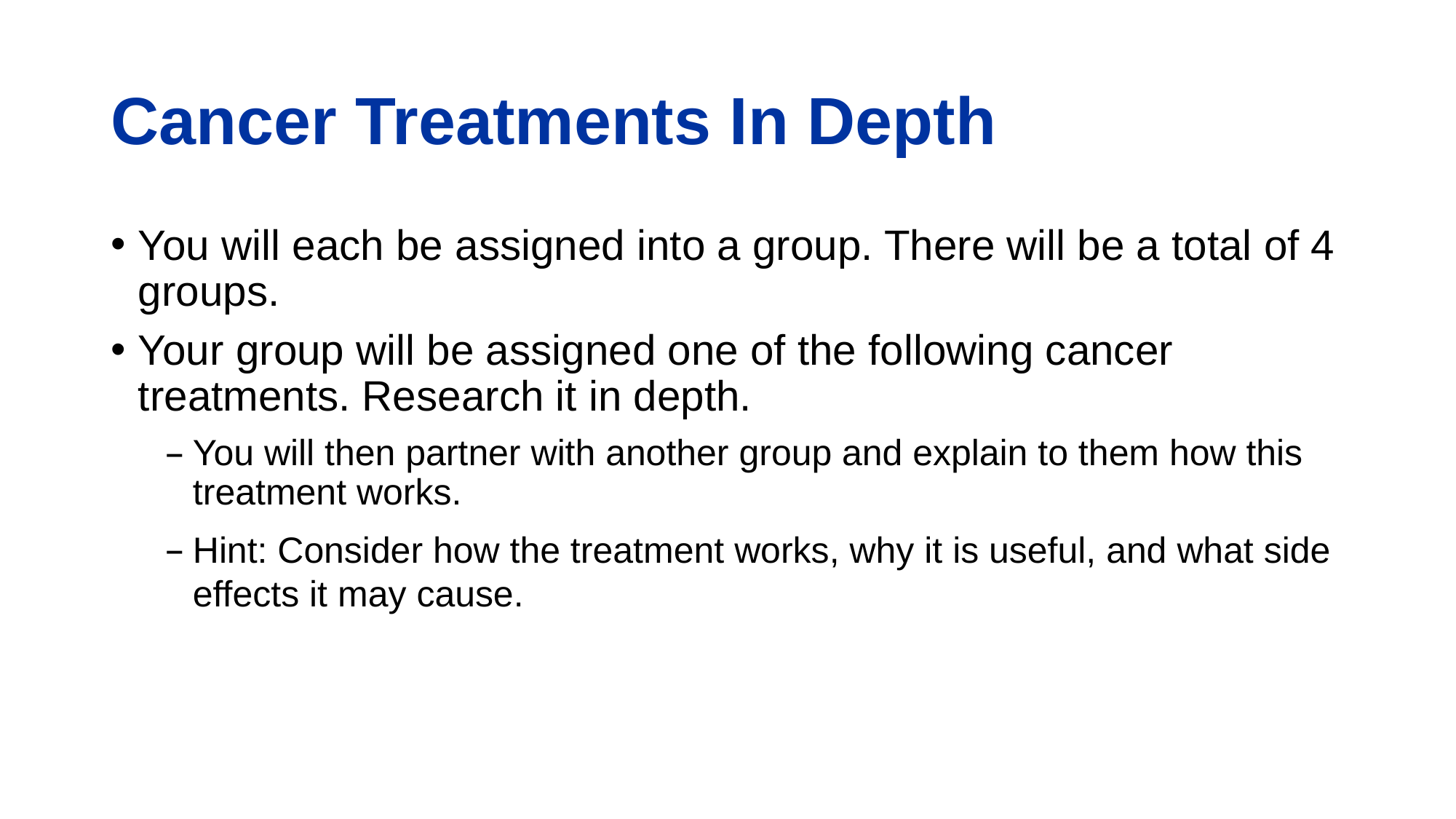

# Cancer Treatments In Depth
You will each be assigned into a group. There will be a total of 4 groups.
Your group will be assigned one of the following cancer treatments. Research it in depth.
You will then partner with another group and explain to them how this treatment works.
Hint: Consider how the treatment works, why it is useful, and what side effects it may cause.

## Slide 4
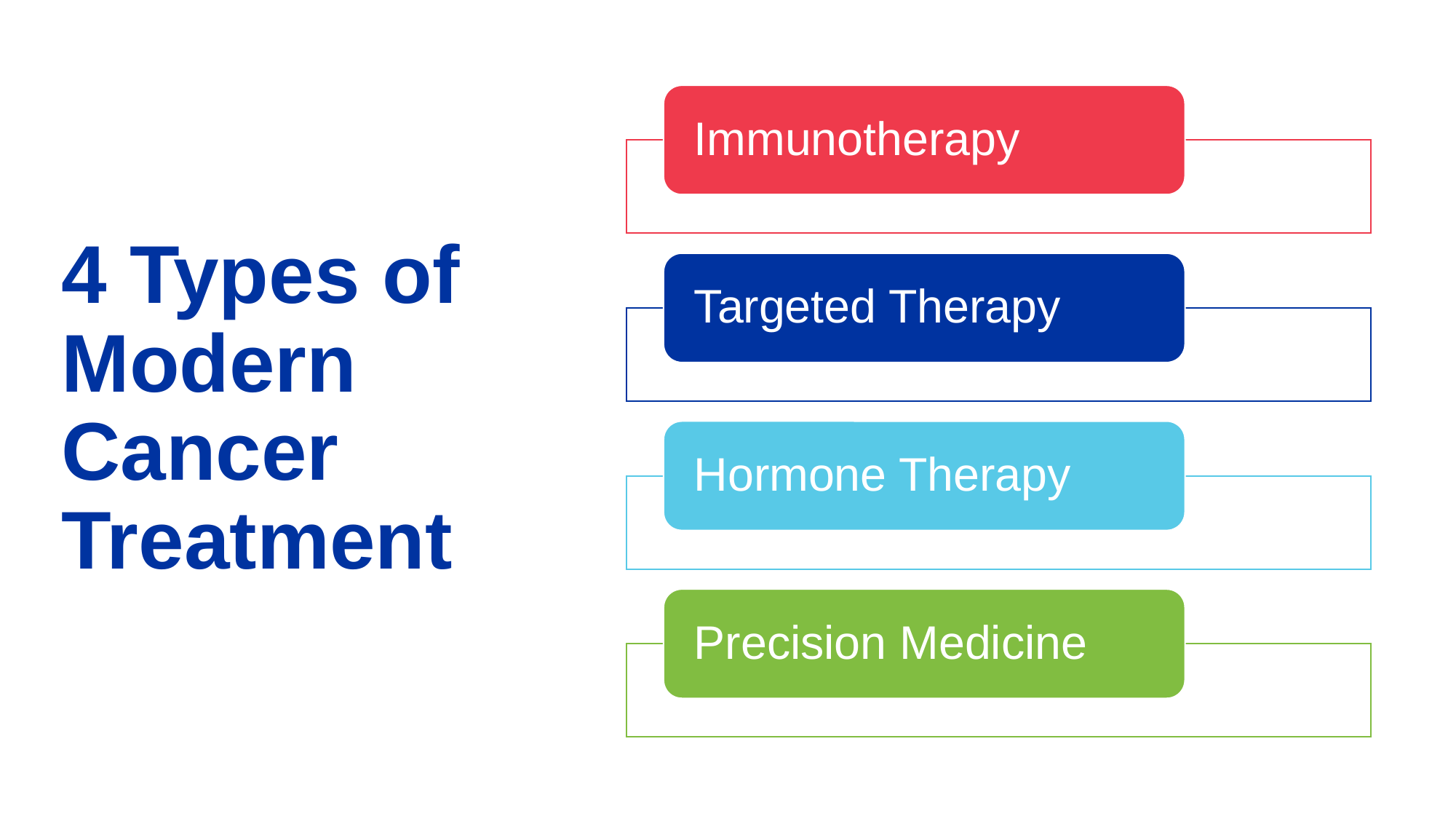

# 4 Types of Modern Cancer Treatment

## Slide 5
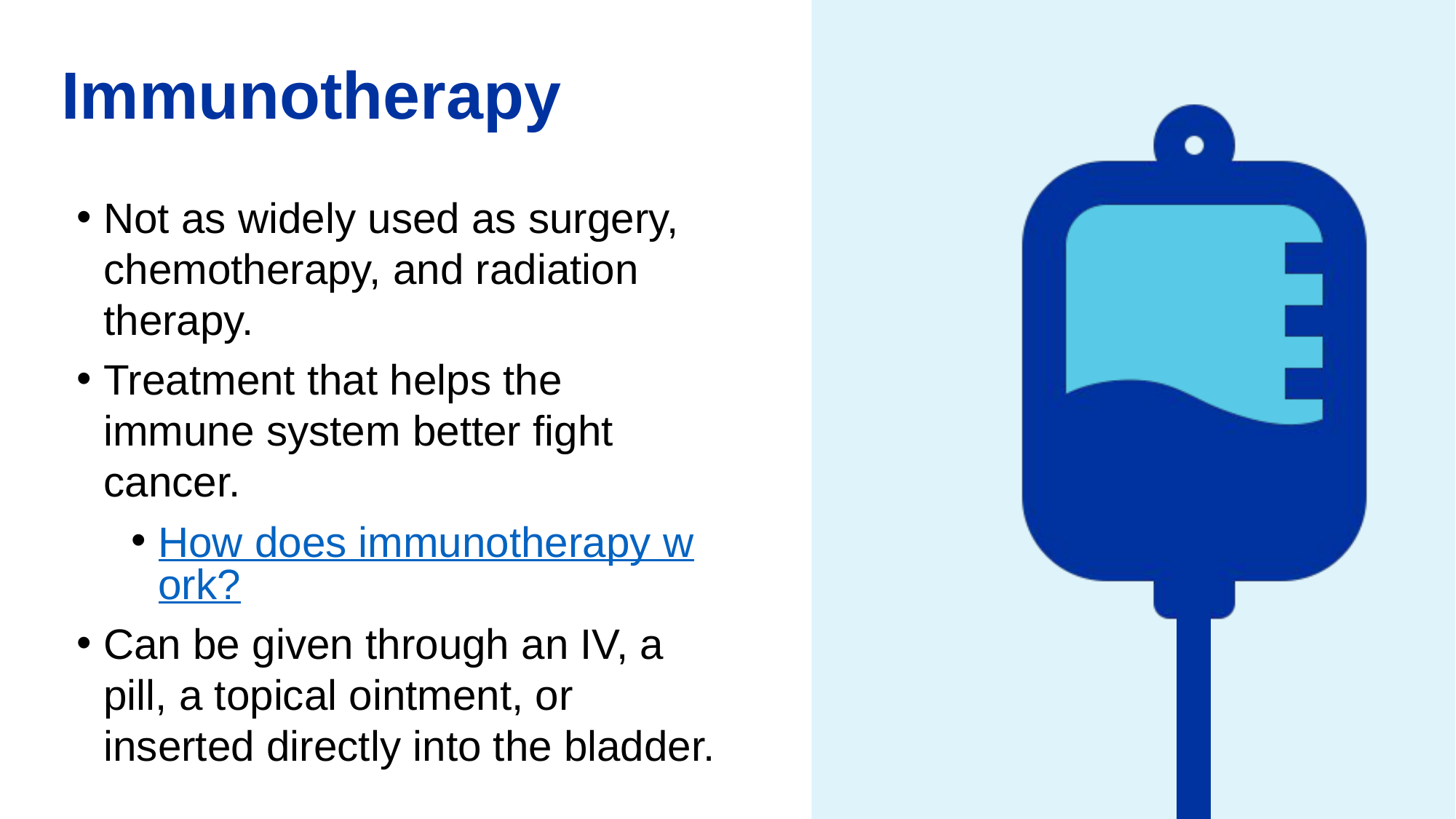

# Immunotherapy
Not as widely used as surgery, chemotherapy, and radiation therapy.
Treatment that helps the immune system better fight cancer.
How does immunotherapy work?
Can be given through an IV, a pill, a topical ointment, or inserted directly into the bladder.

## Slide 6
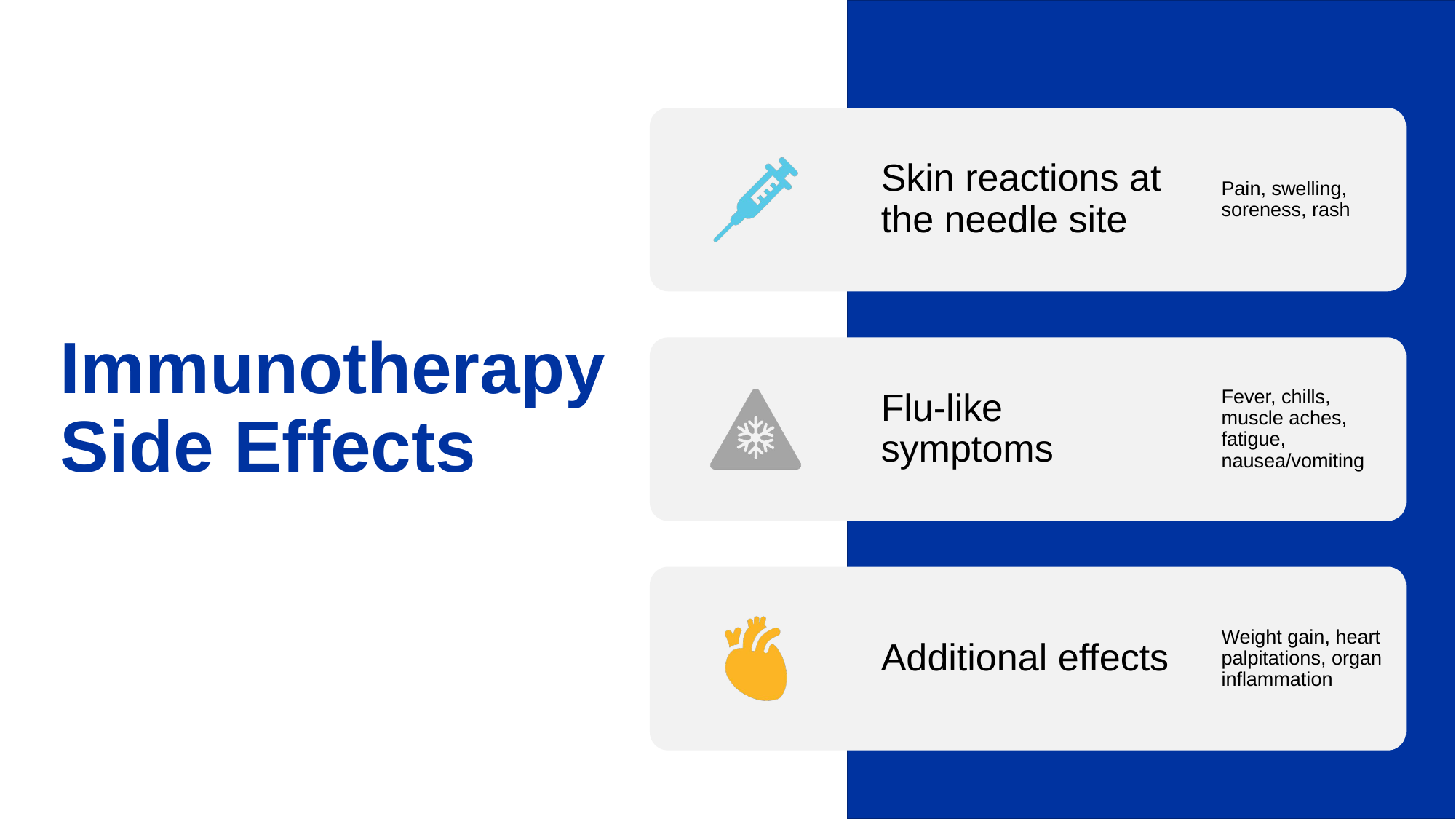

# Immunotherapy Side Effects

## Slide 7
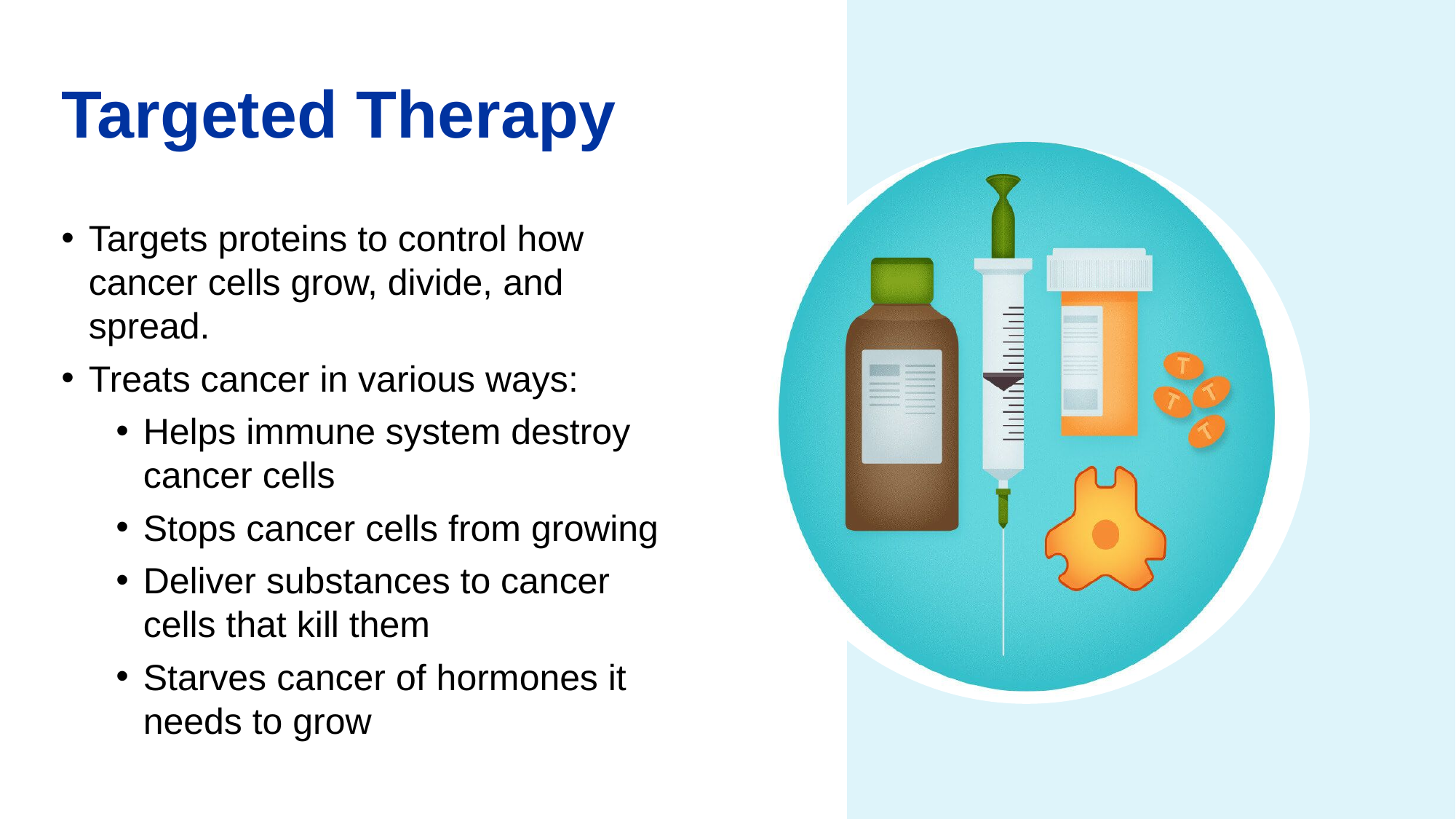

# Targeted Therapy
Targets proteins to control how cancer cells grow, divide, and spread.
Treats cancer in various ways:
Helps immune system destroy cancer cells
Stops cancer cells from growing
Deliver substances to cancer cells that kill them
Starves cancer of hormones it needs to grow

## Slide 8
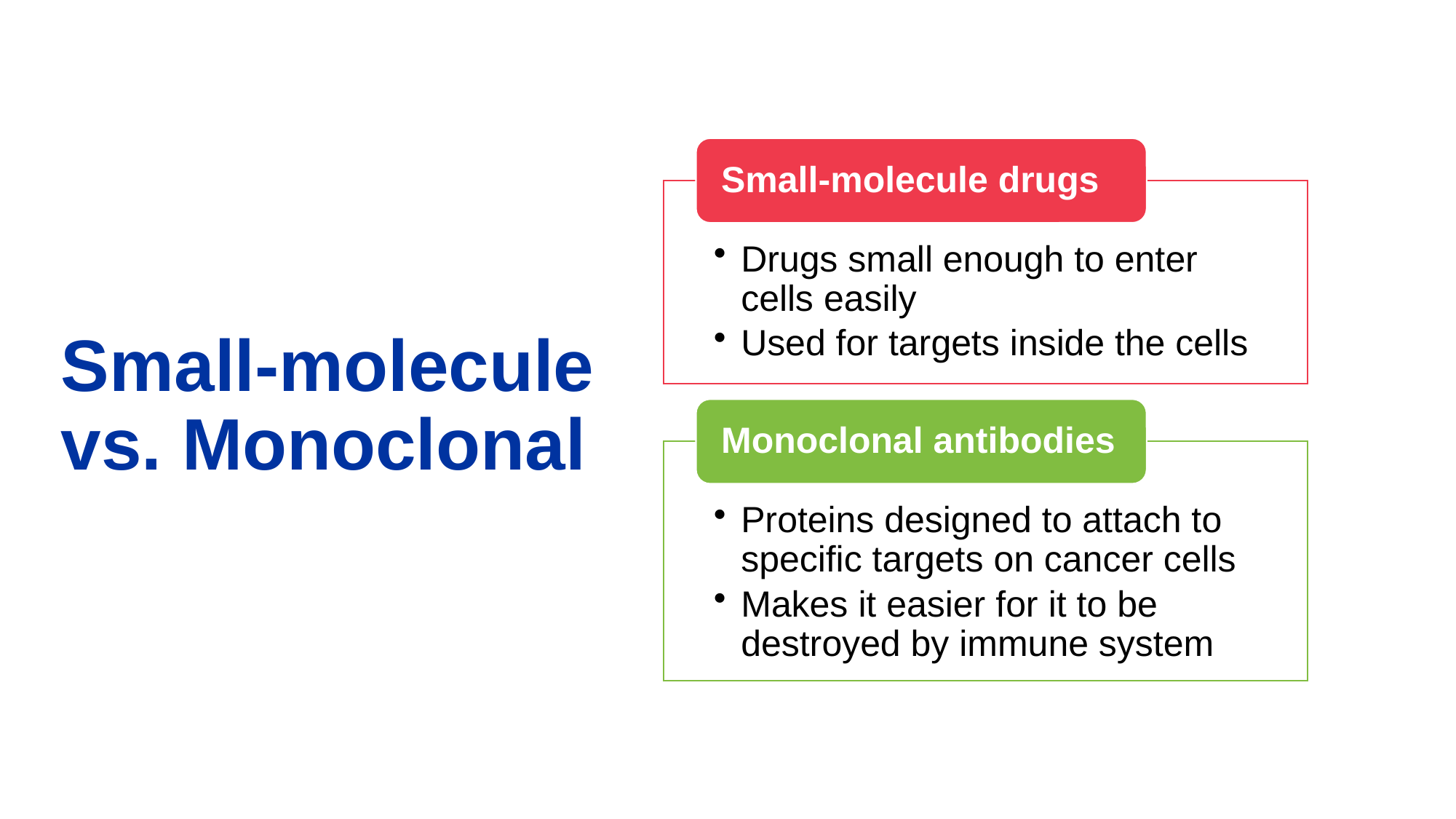

# Small-molecule vs. Monoclonal

## Slide 9
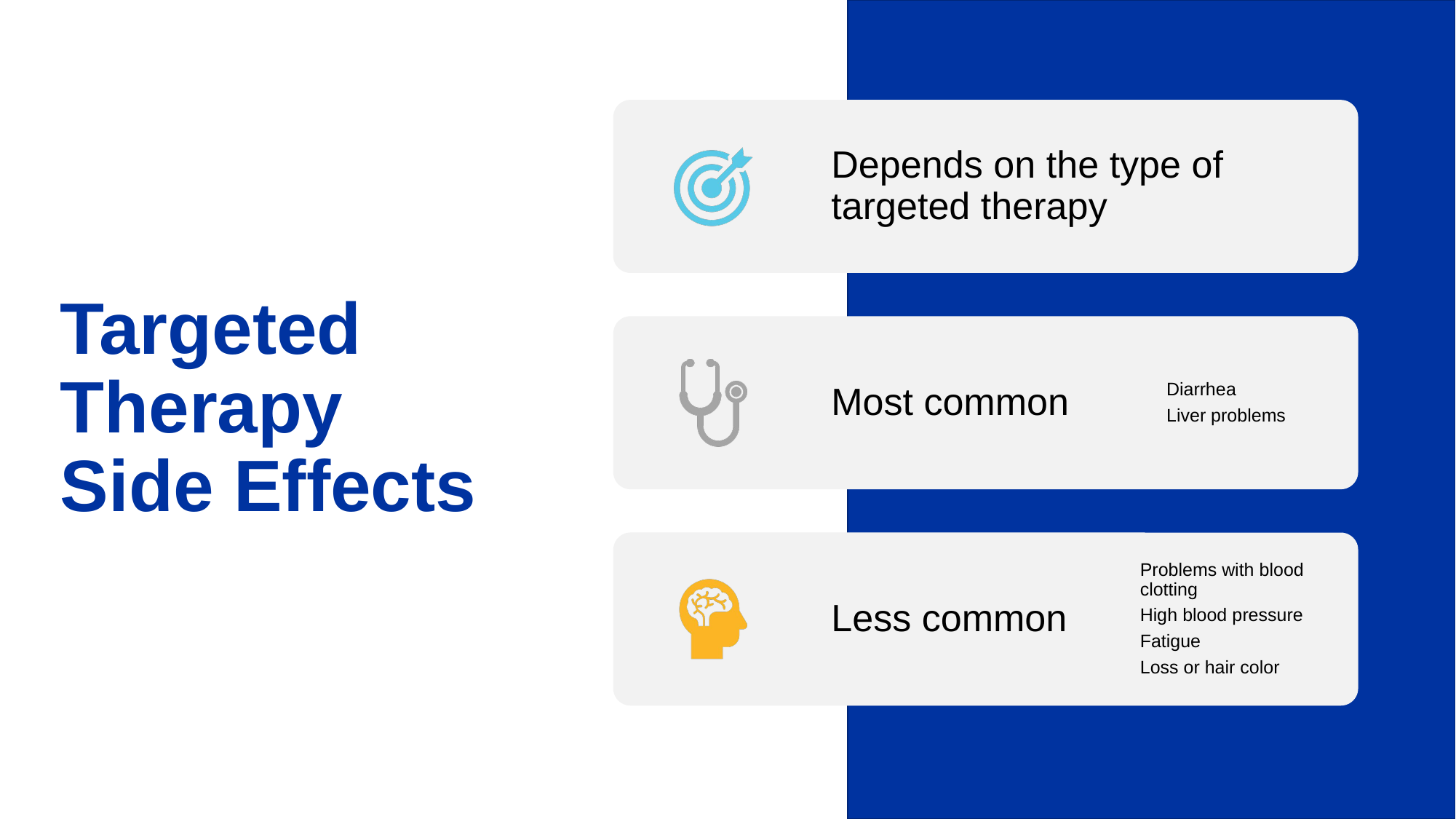

Targeted Therapy
Side Effects

## Slide 10
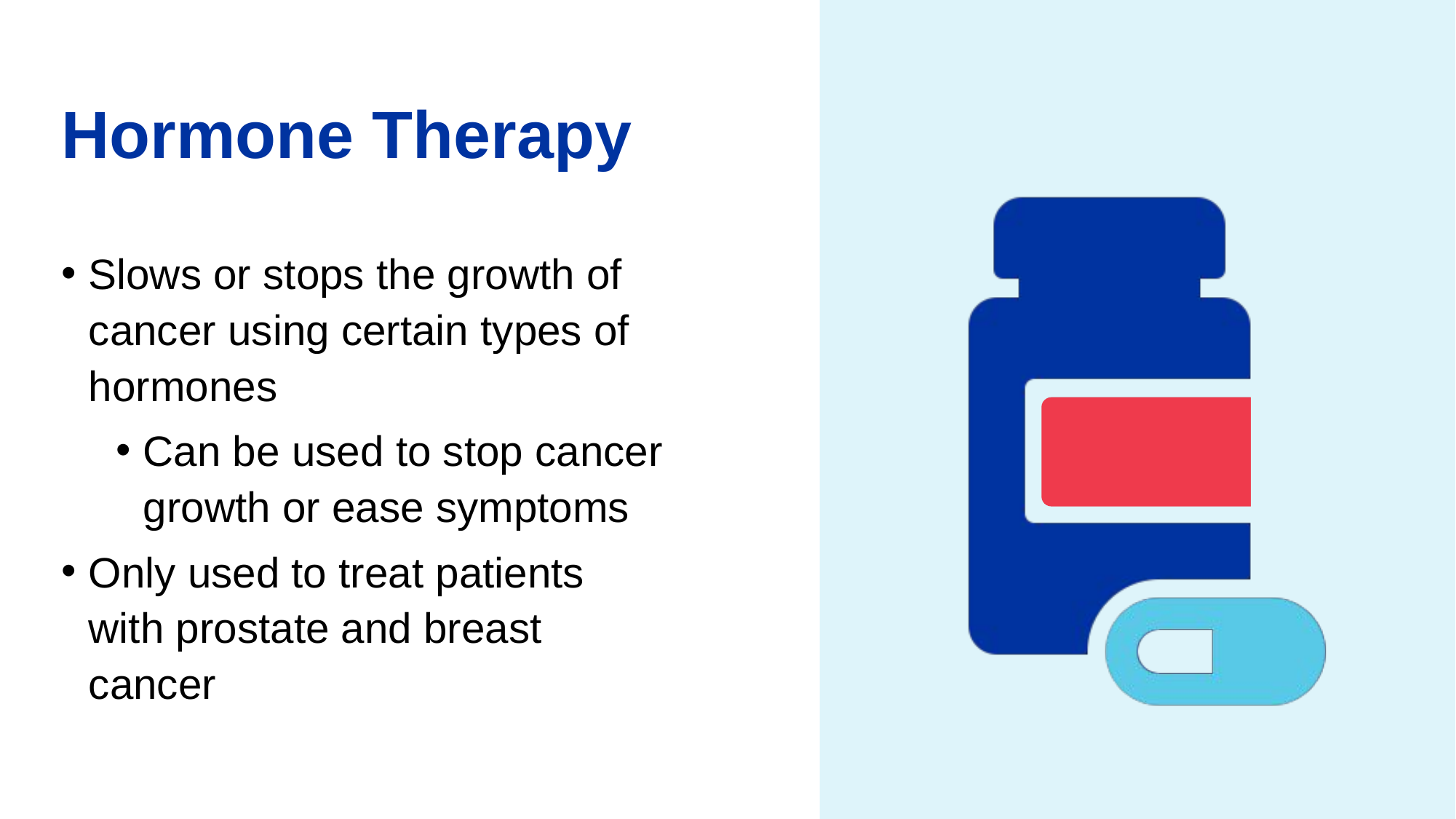

# Hormone Therapy
Slows or stops the growth of cancer using certain types of hormones
Can be used to stop cancer growth or ease symptoms
Only used to treat patients with prostate and breast cancer

## Slide 11
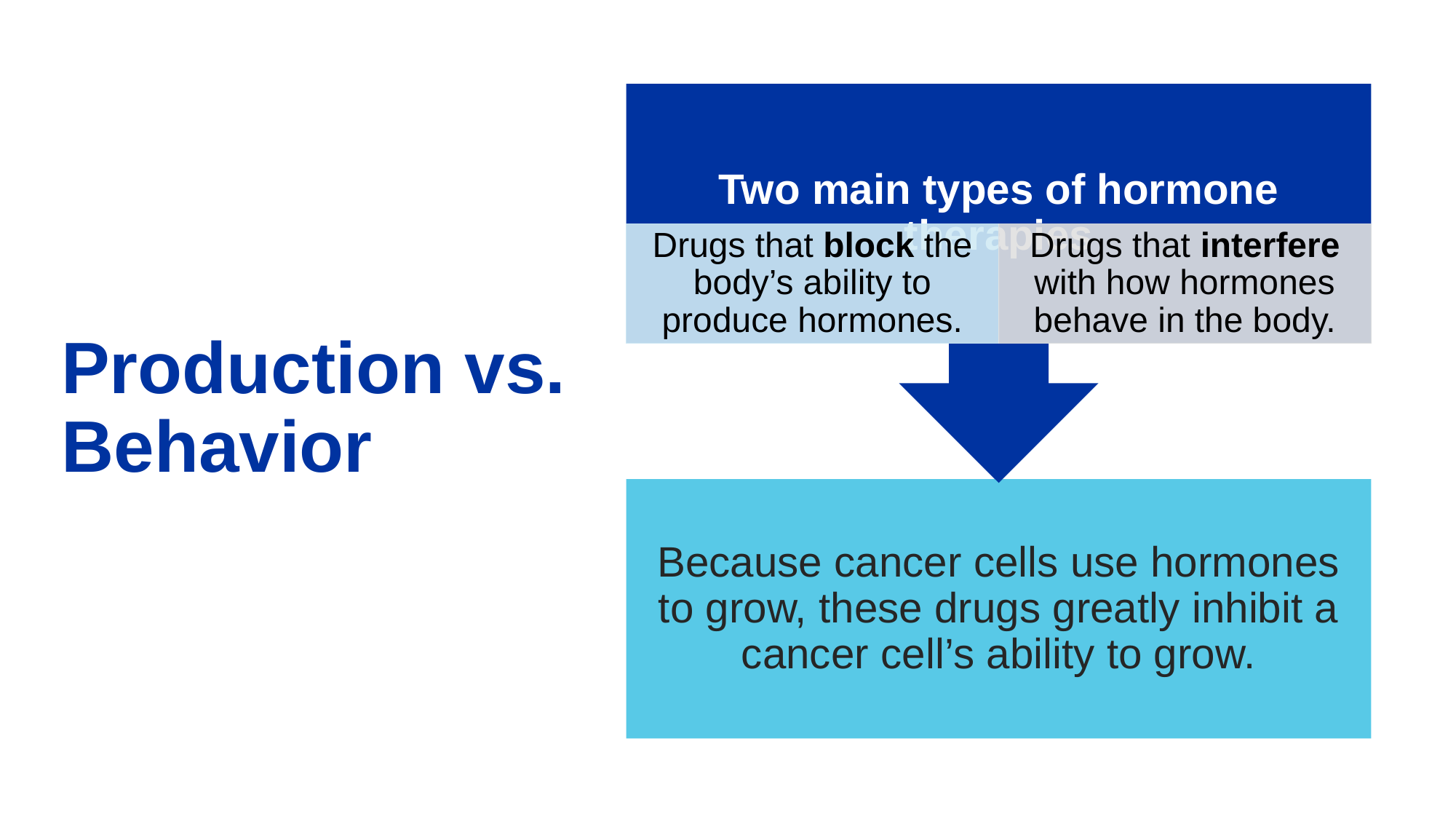

# Production vs. Behavior

## Slide 12
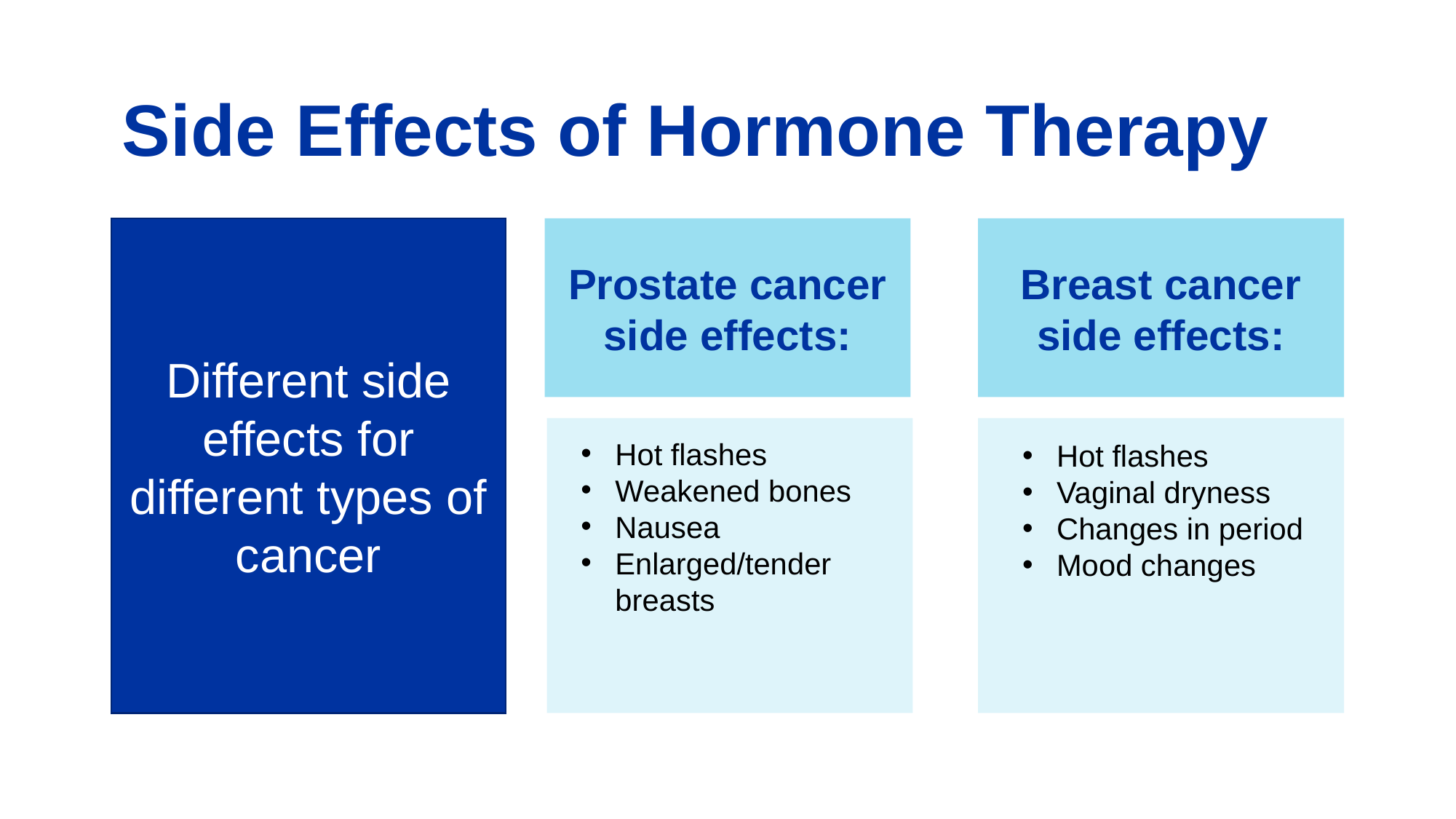

# Side Effects of Hormone Therapy
Different side effects for different types of cancer
Prostate cancer side effects:
Breast cancer side effects:
Hot flashes
Weakened bones
Nausea
Enlarged/tender breasts
Hot flashes
Vaginal dryness
Changes in period
Mood changes

## Slide 13
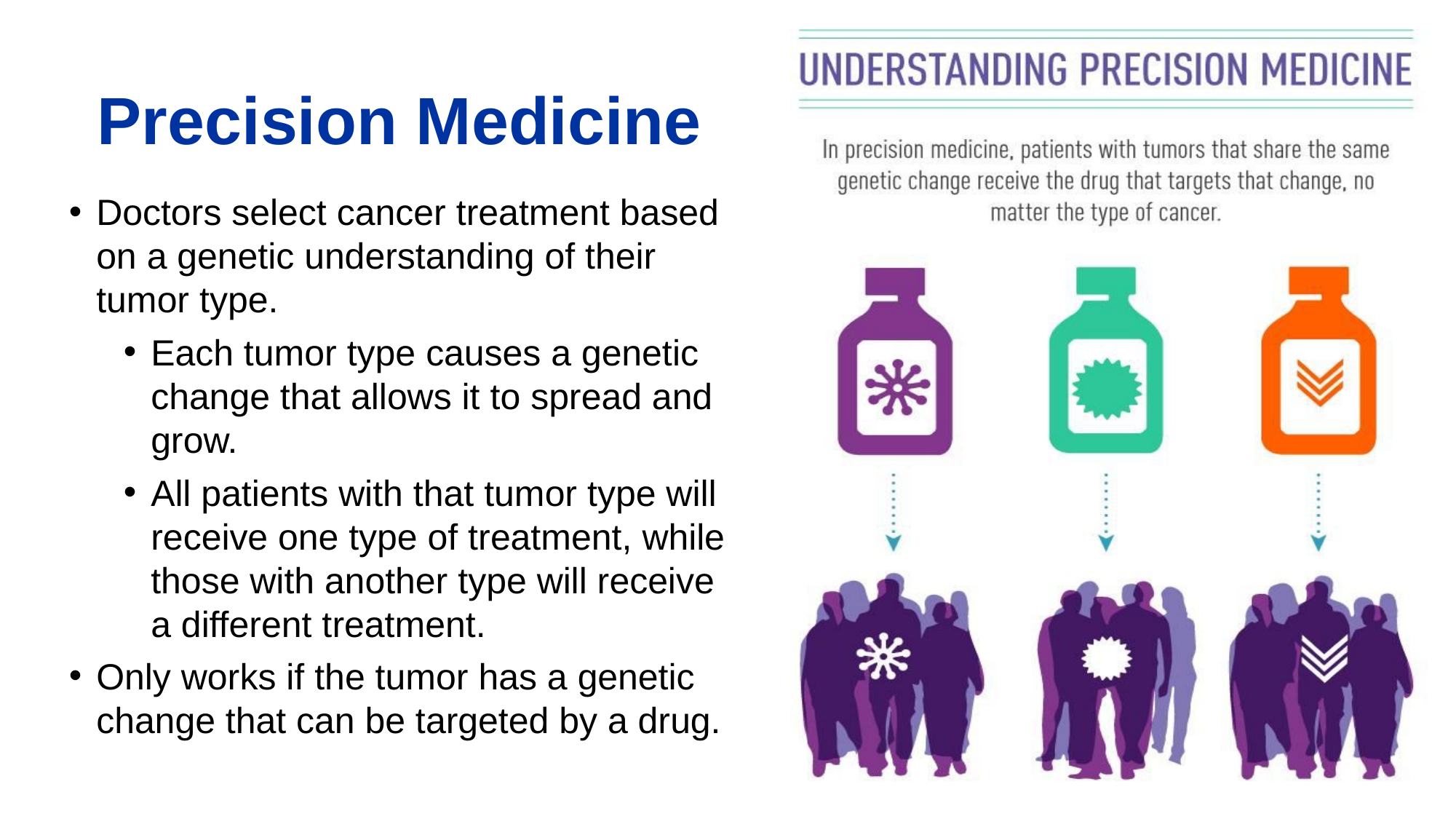

# Precision Medicine
Doctors select cancer treatment based on a genetic understanding of their tumor type.
Each tumor type causes a genetic change that allows it to spread and grow.
All patients with that tumor type will receive one type of treatment, while those with another type will receive a different treatment.
Only works if the tumor has a genetic change that can be targeted by a drug.

## Slide 14
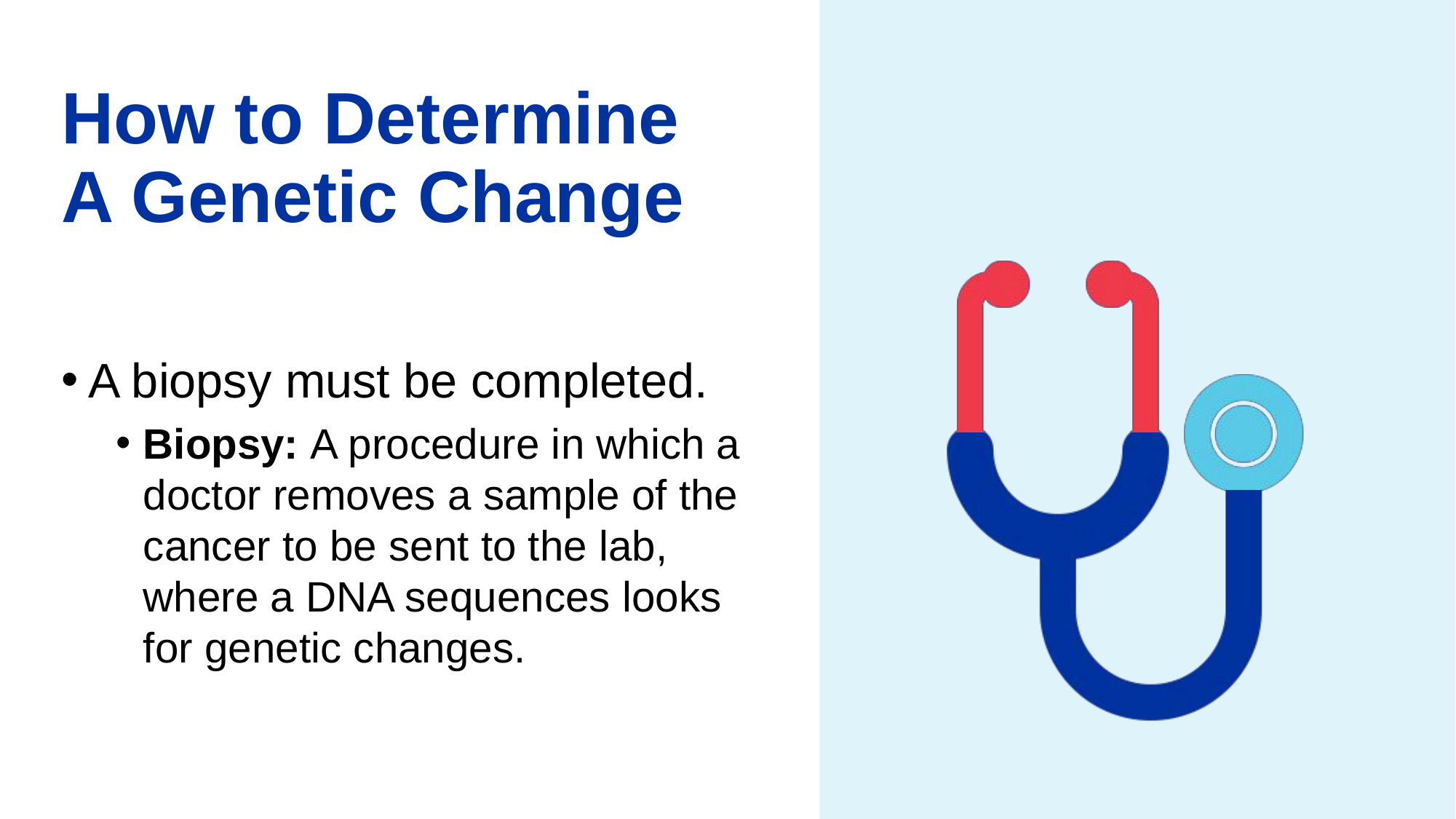

# How to Determine A Genetic Change
A biopsy must be completed.
Biopsy: A procedure in which a doctor removes a sample of the cancer to be sent to the lab, where a DNA sequences looks for genetic changes.

## Slide 15
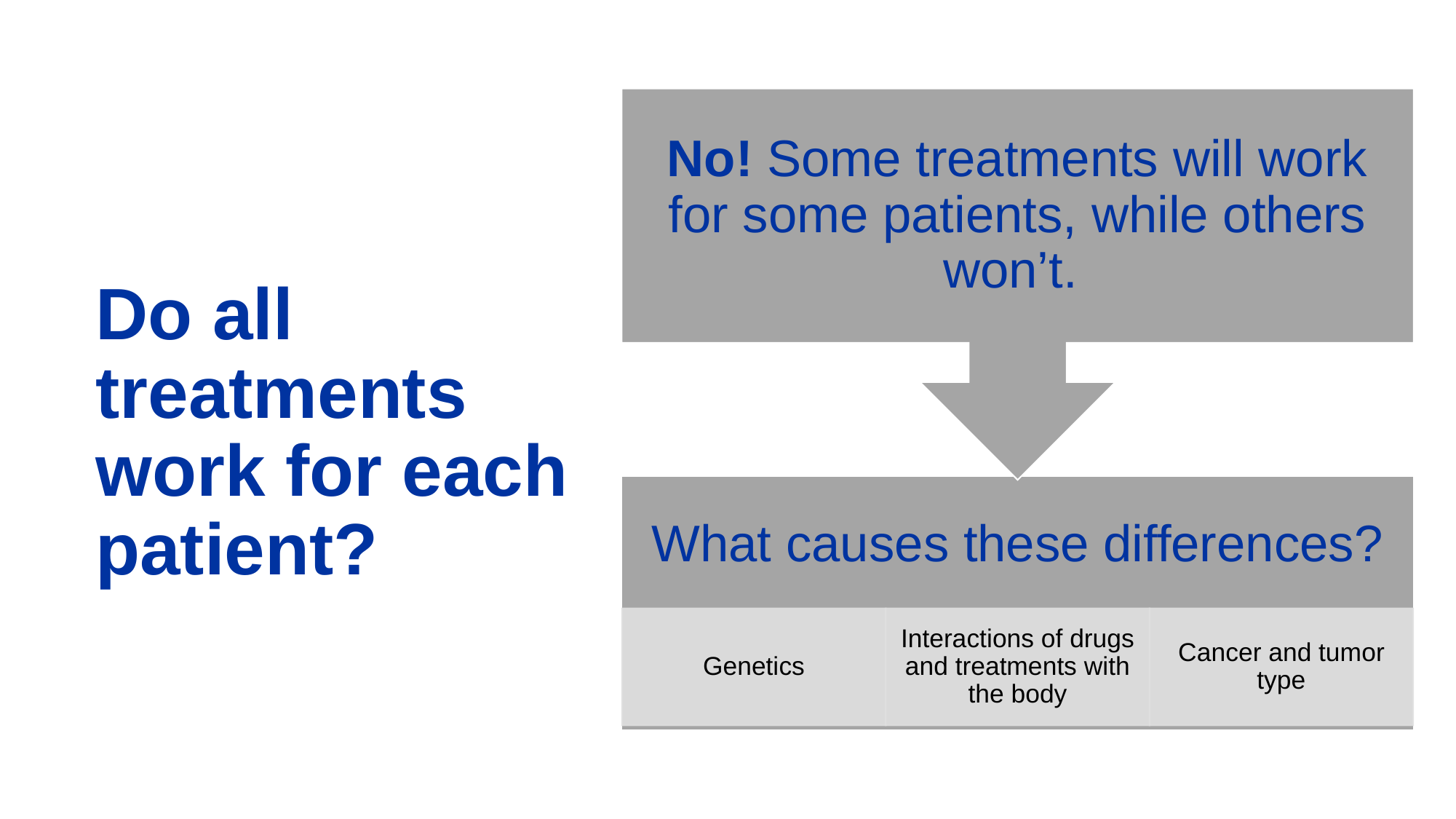

# Do all treatments work for each patient?

## Slide 16
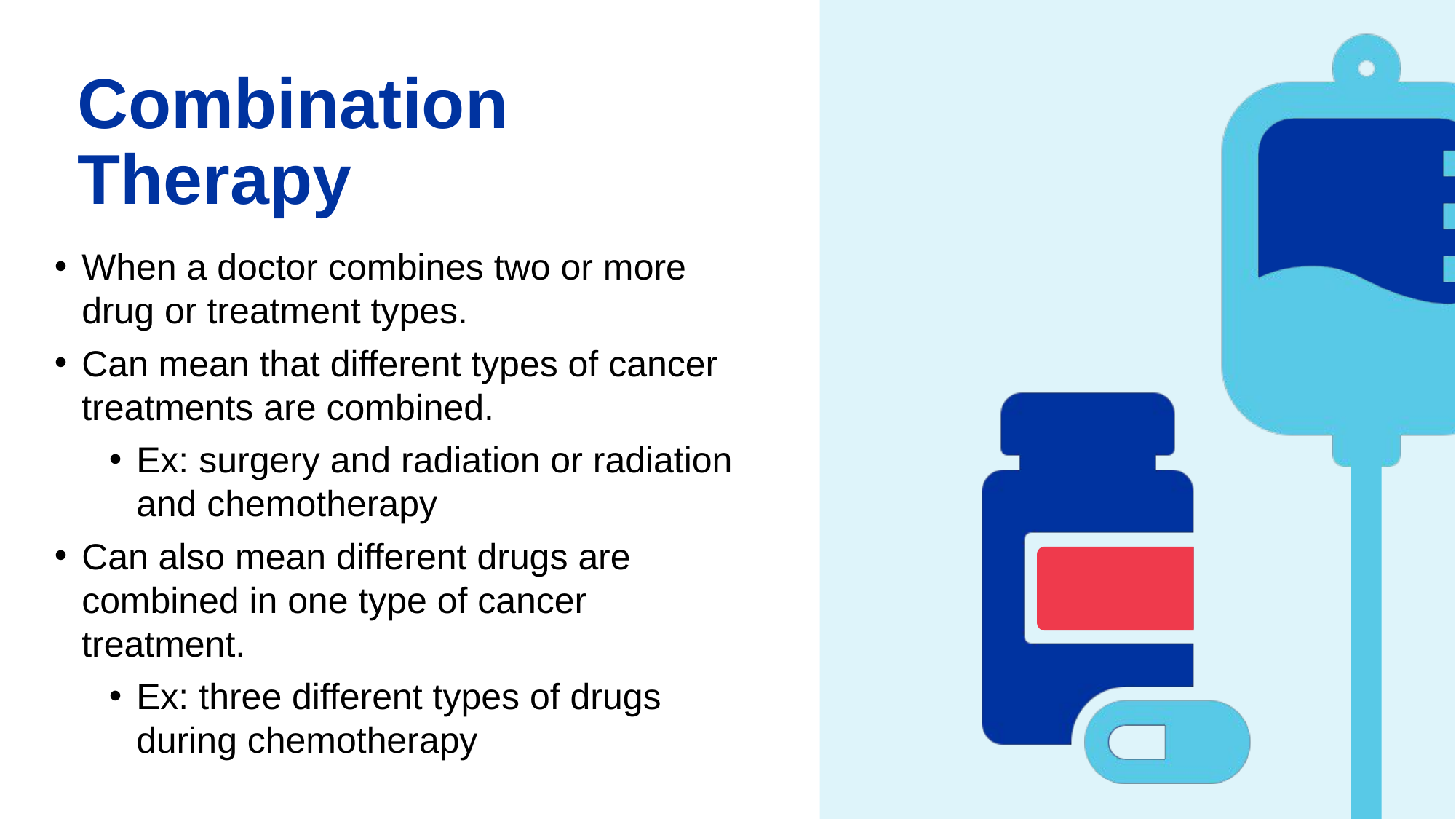

# Combination Therapy
When a doctor combines two or more drug or treatment types.
Can mean that different types of cancer treatments are combined.
Ex: surgery and radiation or radiation and chemotherapy
Can also mean different drugs are combined in one type of cancer treatment.
Ex: three different types of drugs during chemotherapy

## Slide 17
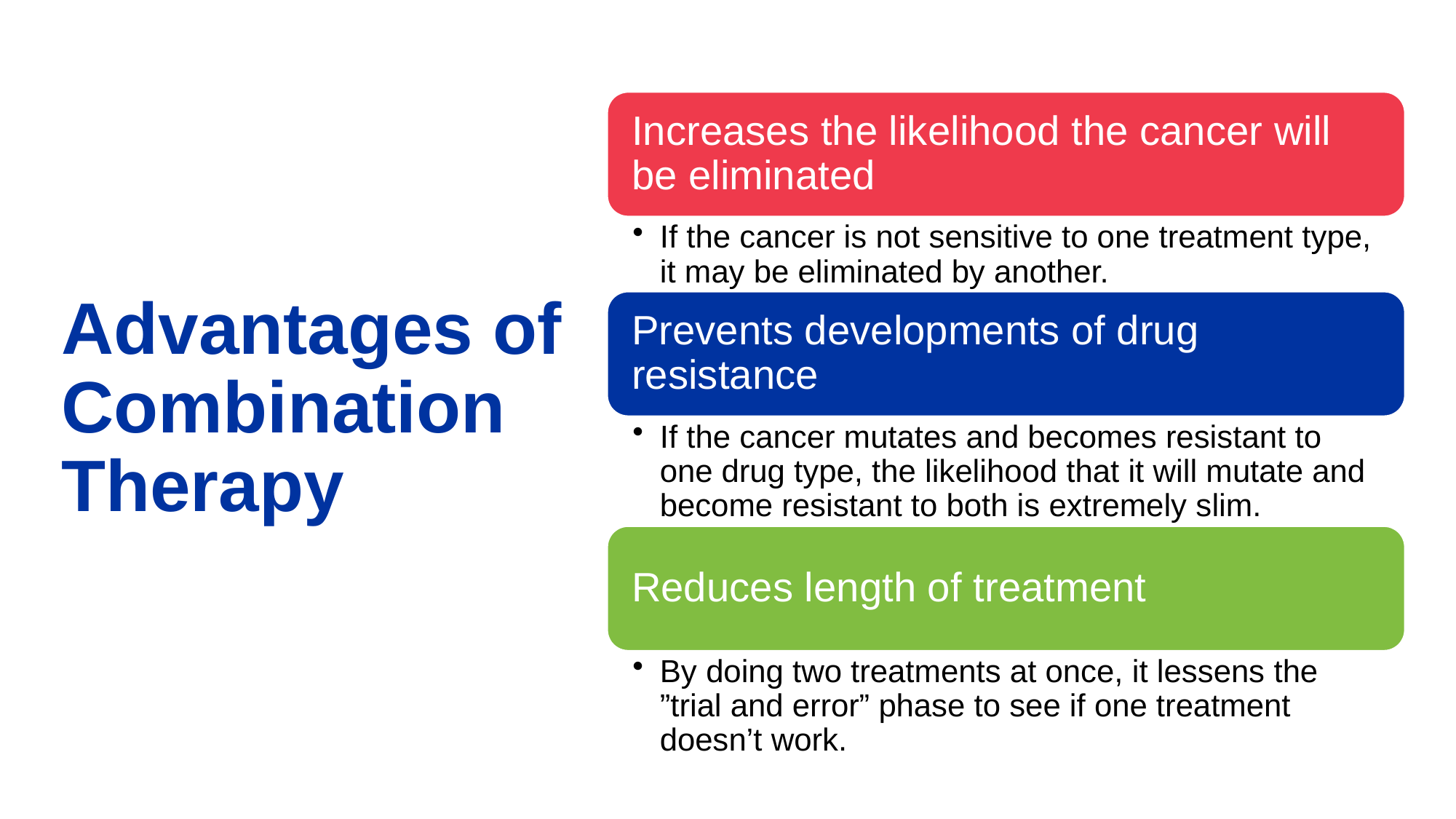

# Advantages of Combination Therapy

## Slide 18
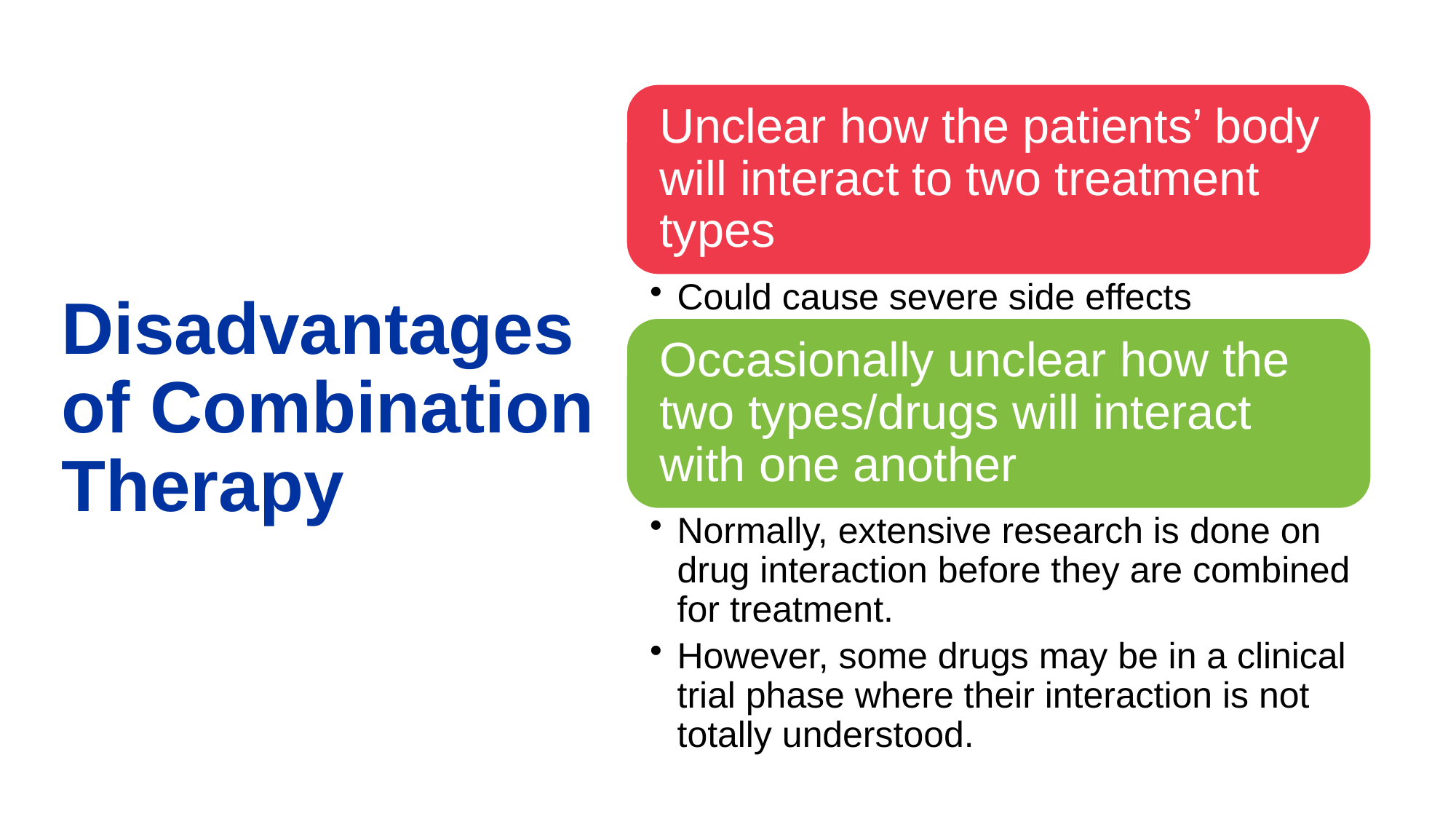

# Disadvantages of Combination Therapy

## Slide 19
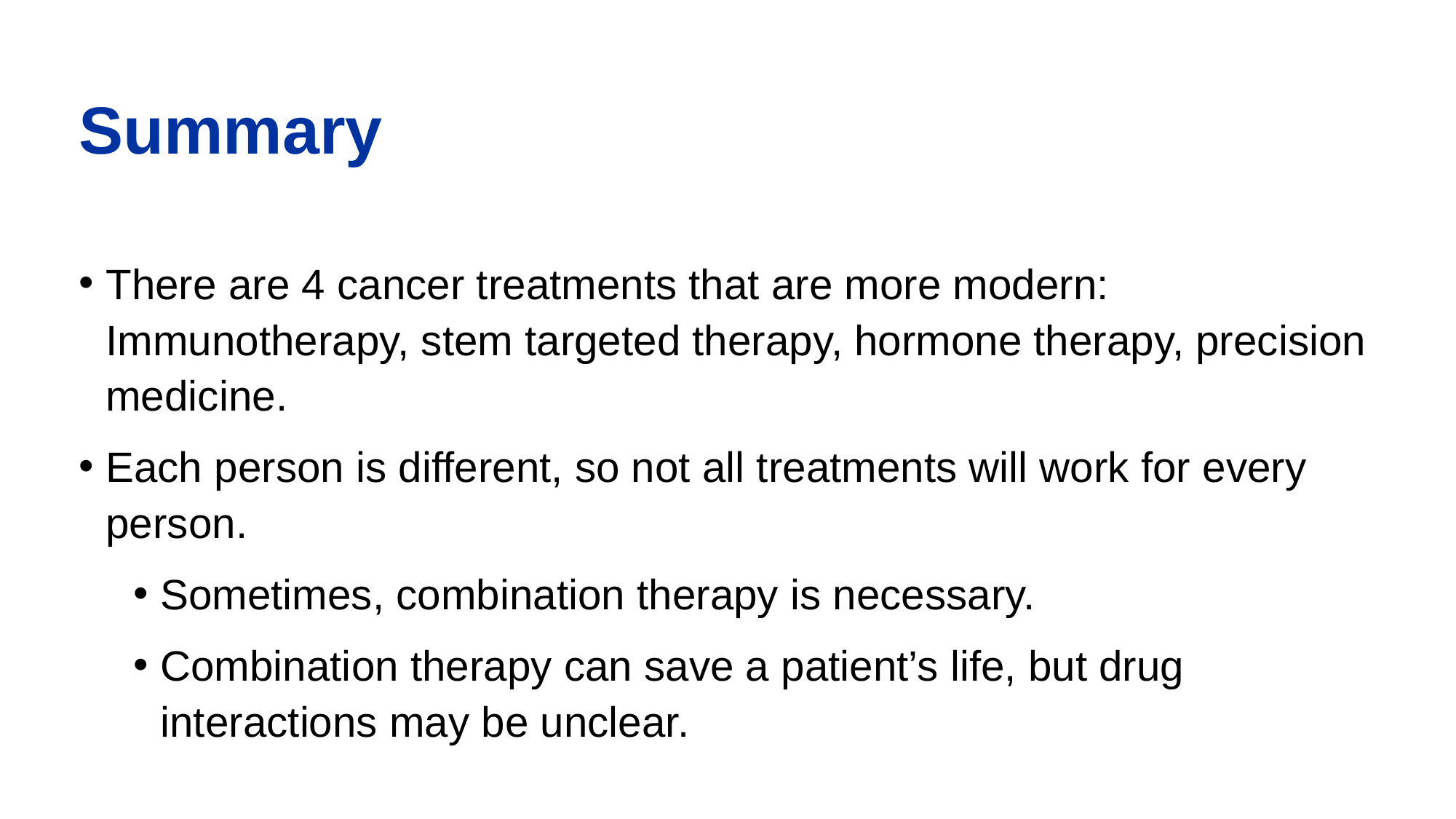

# Summary
There are 4 cancer treatments that are more modern: Immunotherapy, stem targeted therapy, hormone therapy, precision medicine.
Each person is different, so not all treatments will work for every person.
Sometimes, combination therapy is necessary.
Combination therapy can save a patient’s life, but drug interactions may be unclear.

## Slide 20
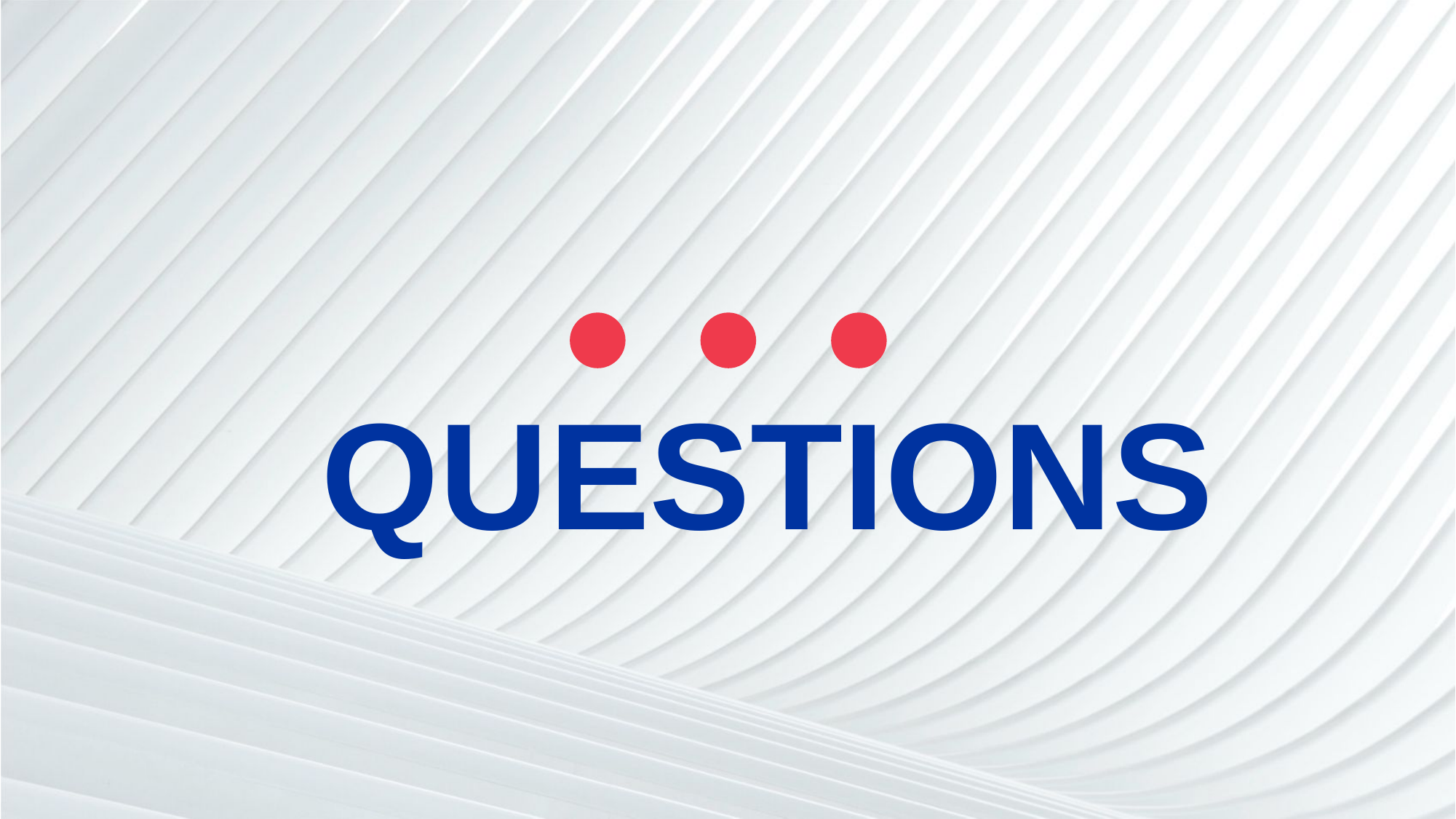

# QUESTIONS
