# Supplementary material for: Cancer Curriculum for Appalachian Kentucky Middle and High Schools
Source: J Appalach Health. 2021 Jan 24;3(1):43–55. doi: 10.13023/jah.0301.05 (PMC8830599; doi:10.13023/jah.0301.05)
Supplement: Supplementary file 6 [file Appendix7-3.1.5Hudson.pptx]

## Slide 1
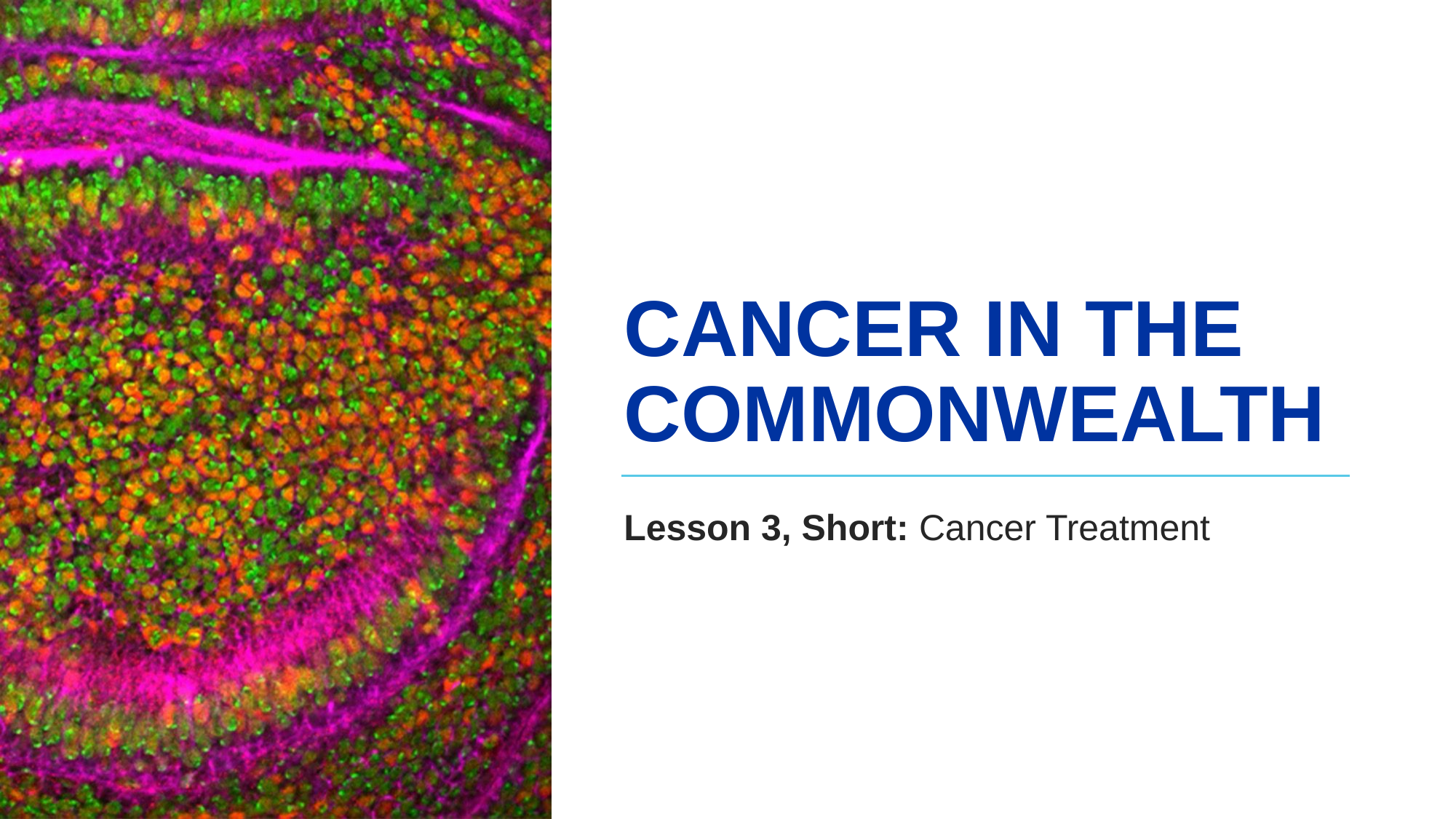

# CANCER IN THE COMMONWEALTH
Lesson 3, Short: Cancer Treatment

## Slide 2
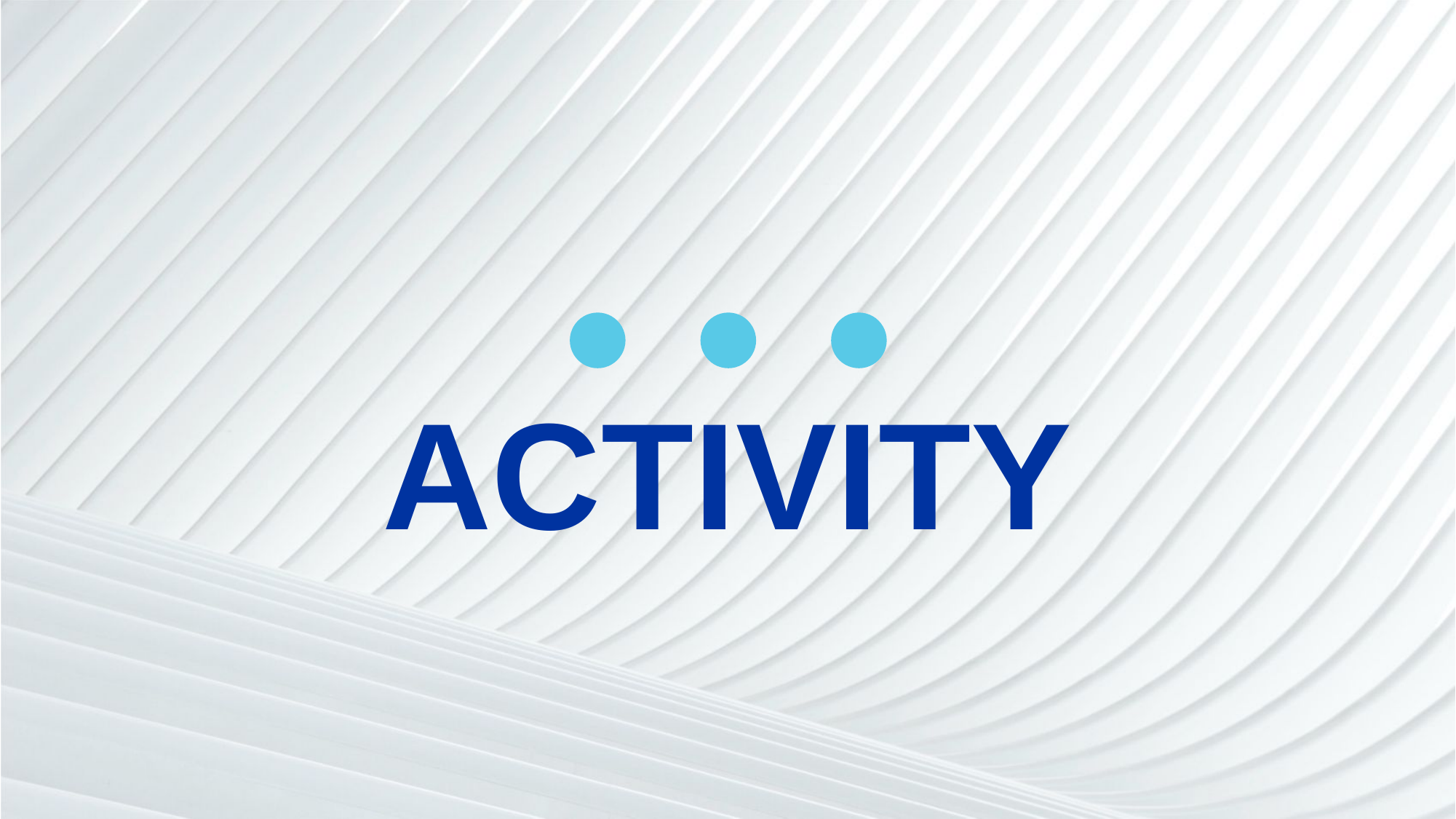

# ACTIVITY

## Slide 3
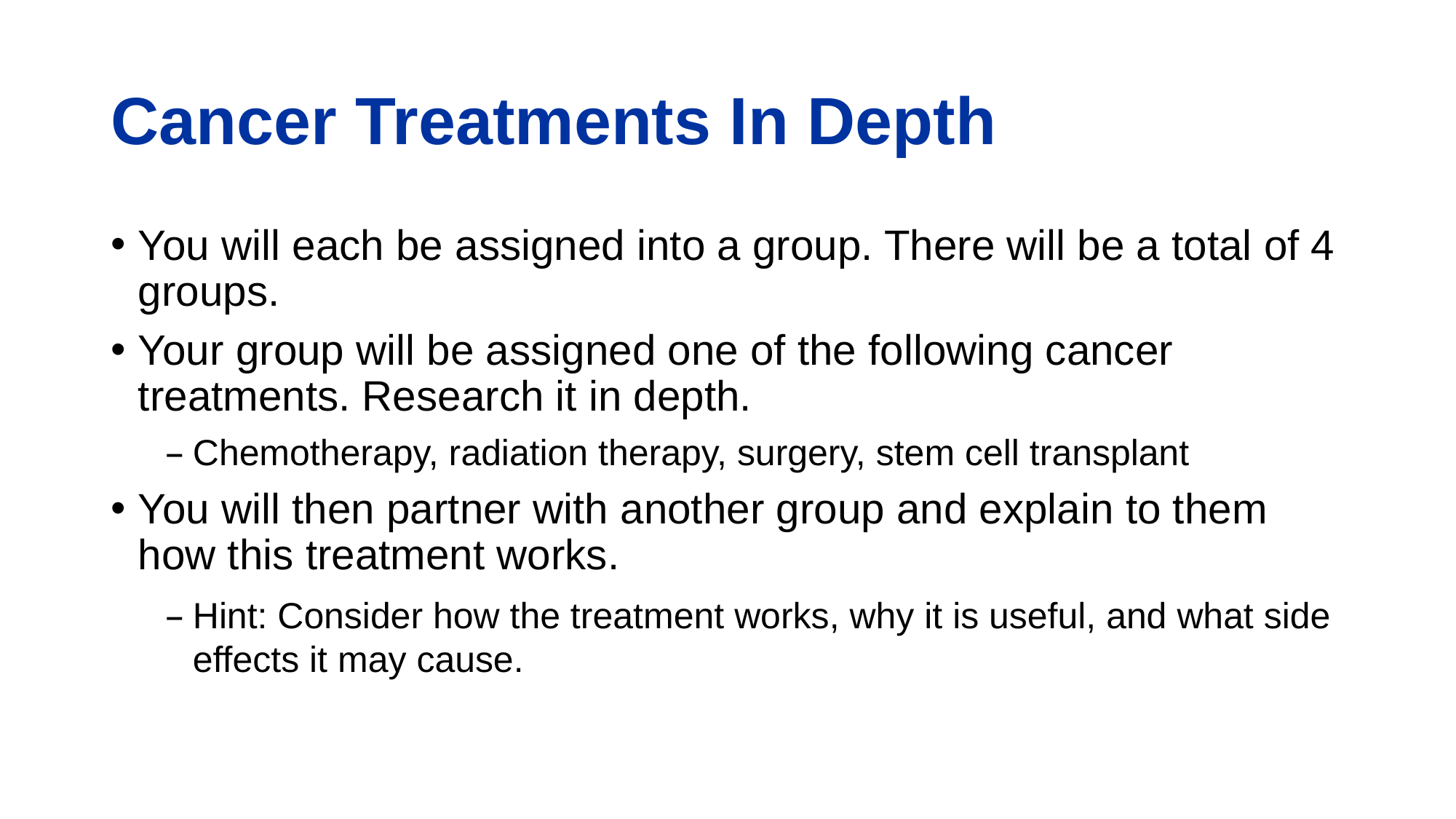

# Cancer Treatments In Depth
You will each be assigned into a group. There will be a total of 4 groups.
Your group will be assigned one of the following cancer treatments. Research it in depth.
Chemotherapy, radiation therapy, surgery, stem cell transplant
You will then partner with another group and explain to them how this treatment works.
Hint: Consider how the treatment works, why it is useful, and what side effects it may cause.

## Slide 4
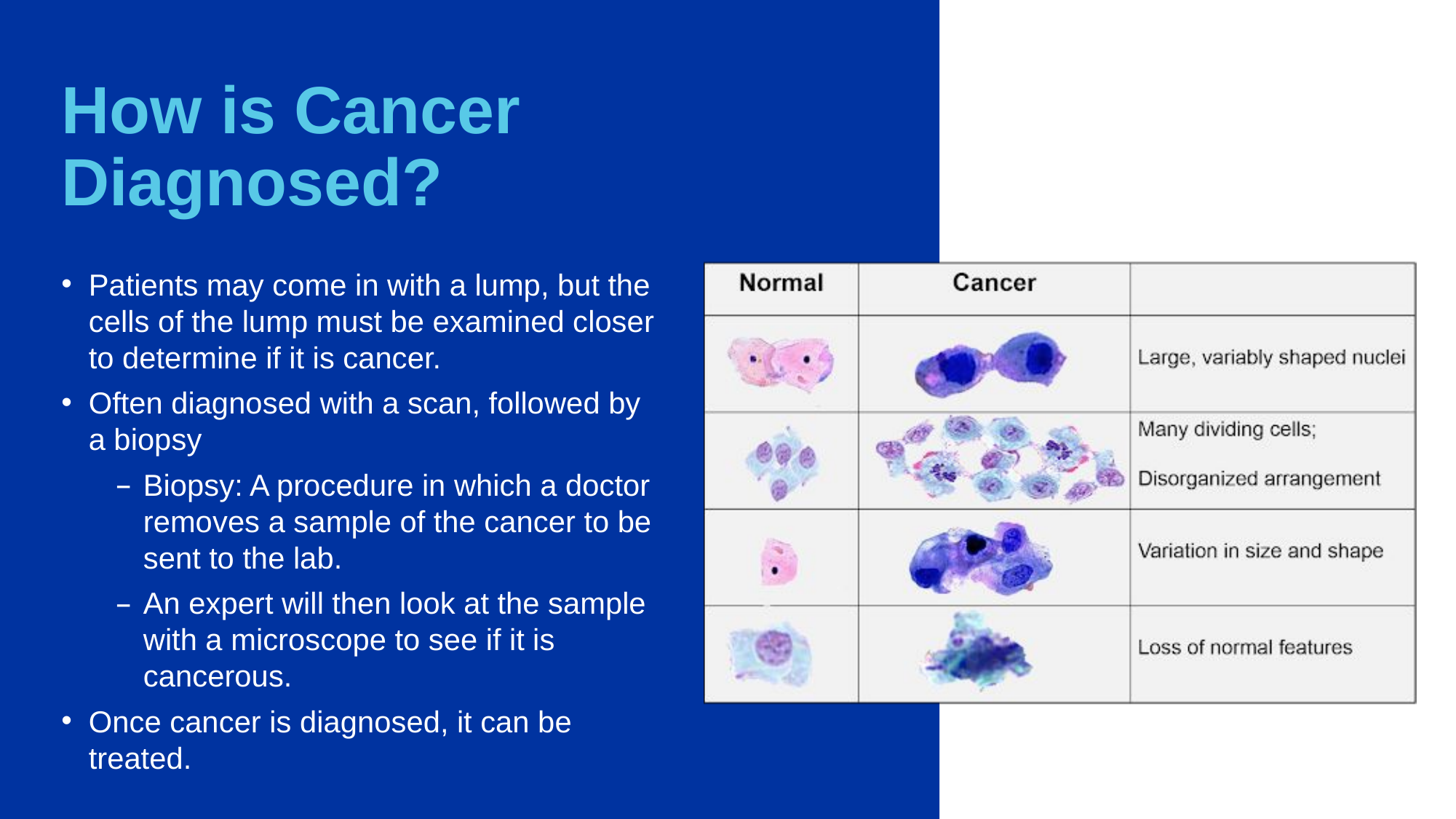

# How is Cancer Diagnosed?
Patients may come in with a lump, but the cells of the lump must be examined closer to determine if it is cancer.
Often diagnosed with a scan, followed by a biopsy
Biopsy: A procedure in which a doctor removes a sample of the cancer to be sent to the lab.
An expert will then look at the sample with a microscope to see if it is cancerous.
Once cancer is diagnosed, it can be treated.

## Slide 5
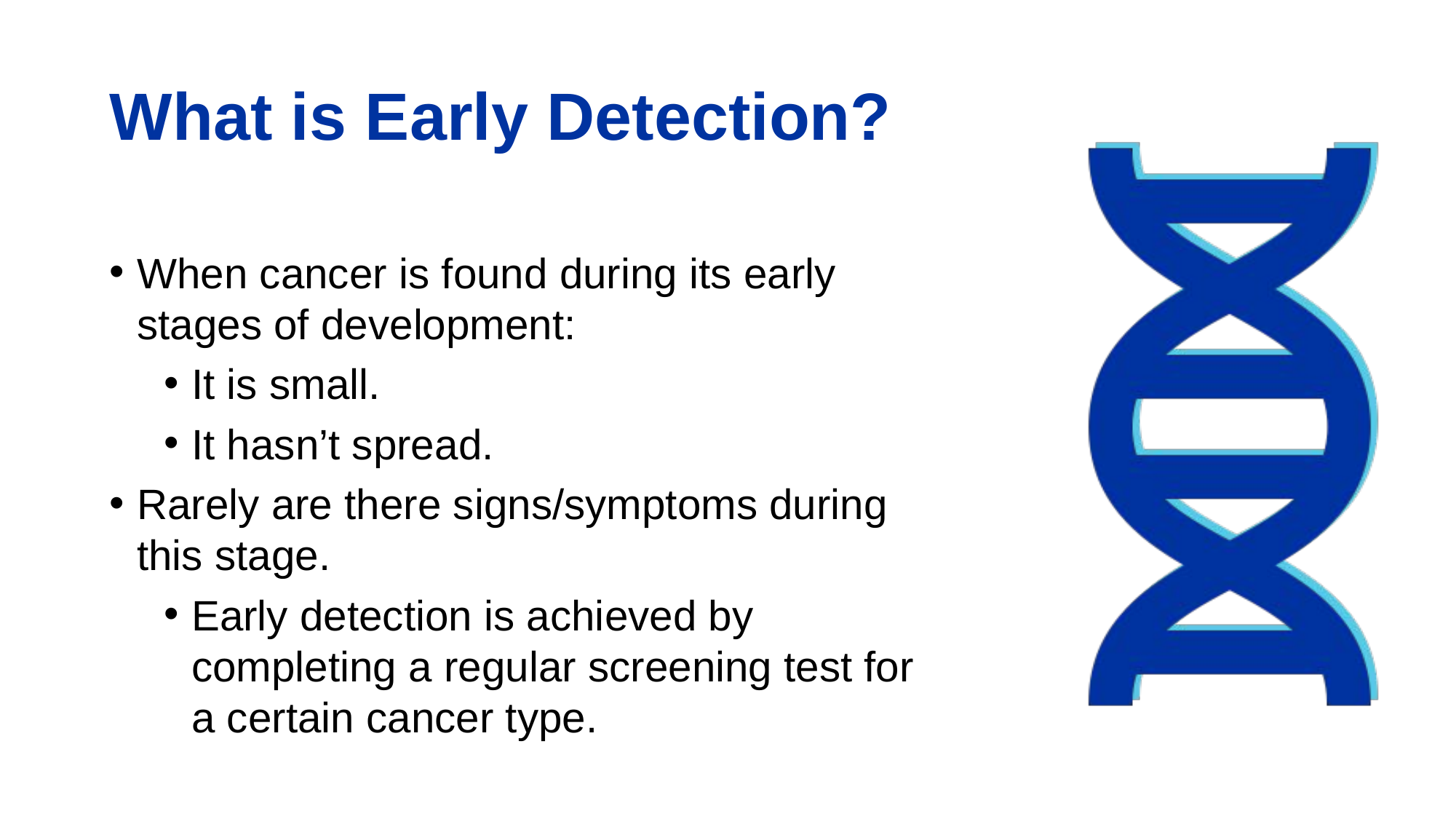

# What is Early Detection?
When cancer is found during its early stages of development:
It is small.
It hasn’t spread.
Rarely are there signs/symptoms during this stage.
Early detection is achieved by completing a regular screening test for a certain cancer type.

## Slide 6
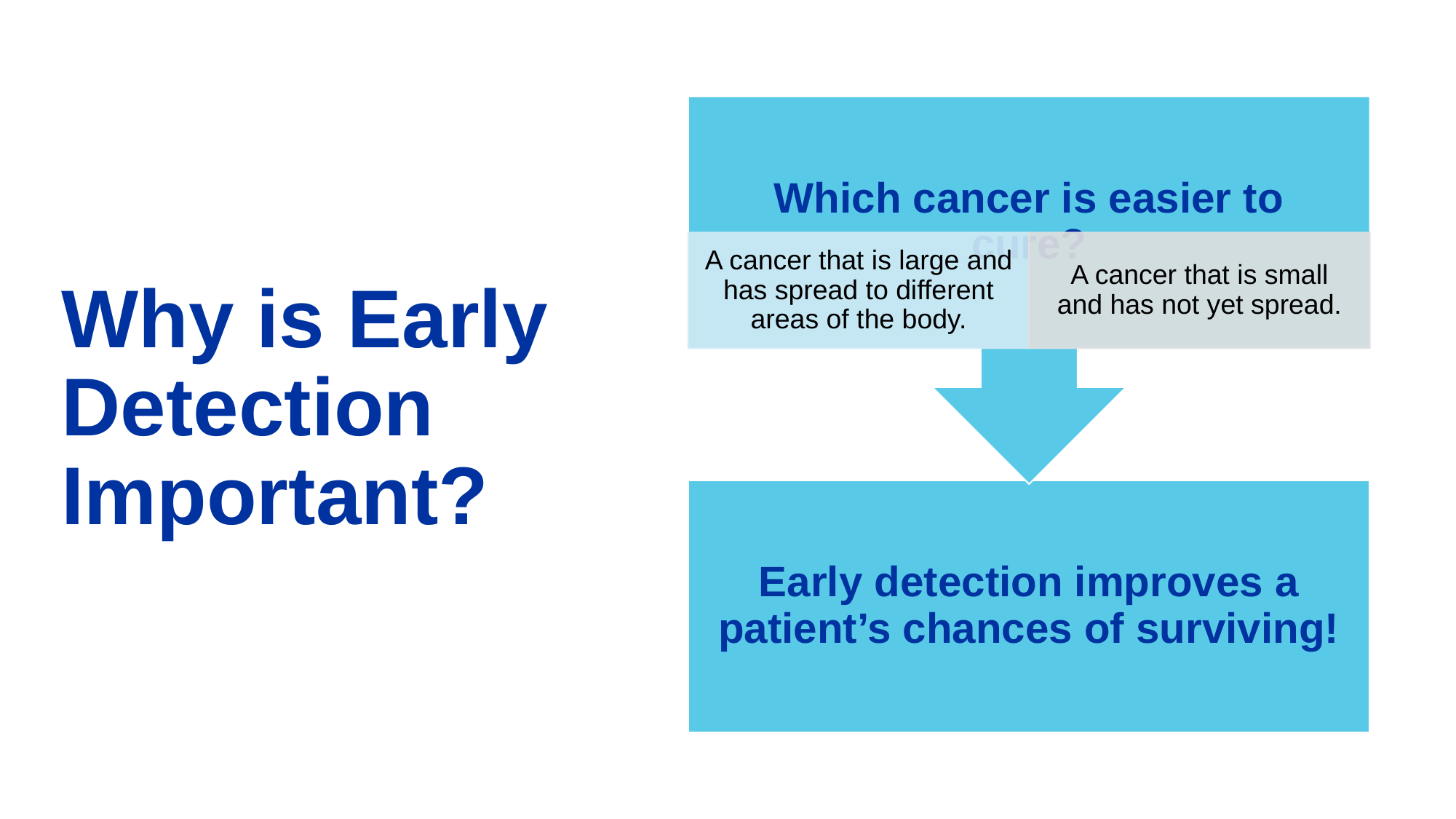

# Why is Early Detection Important?

## Slide 7
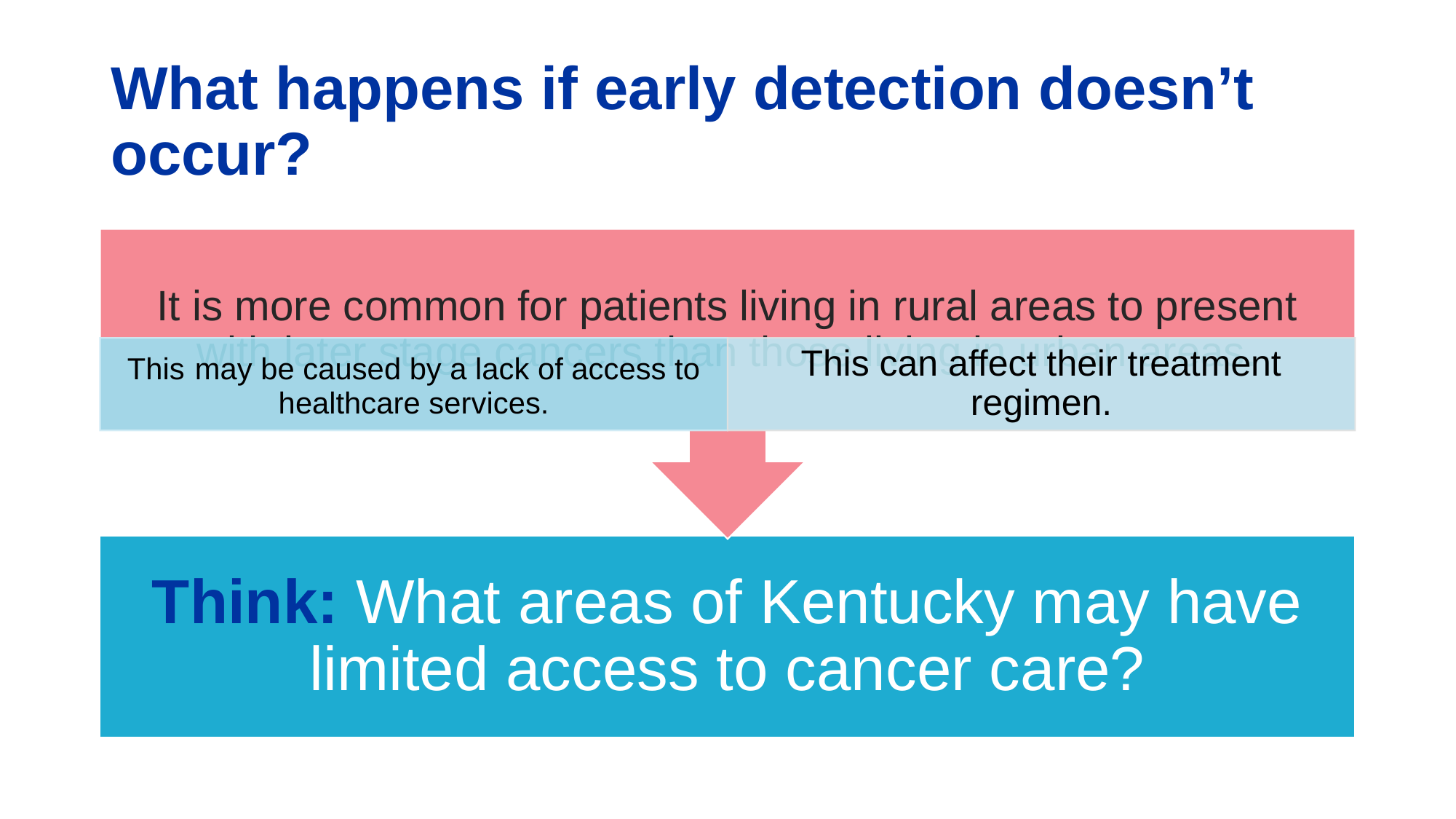

# What happens if early detection doesn’t occur?

## Slide 8
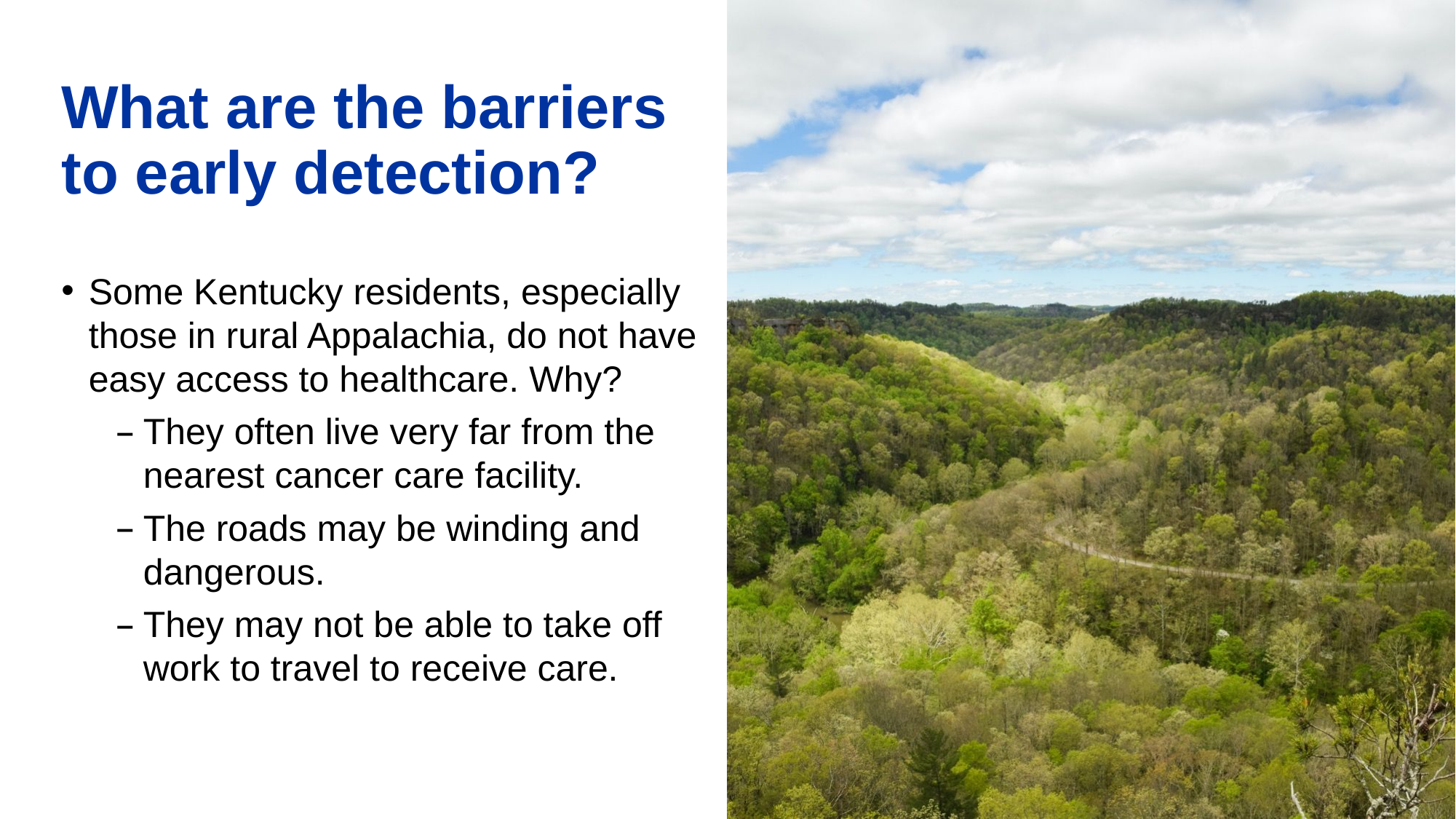

# What are the barriers to early detection?
Some Kentucky residents, especially those in rural Appalachia, do not have easy access to healthcare. Why?
They often live very far from the nearest cancer care facility.
The roads may be winding and dangerous.
They may not be able to take off work to travel to receive care.

## Slide 9
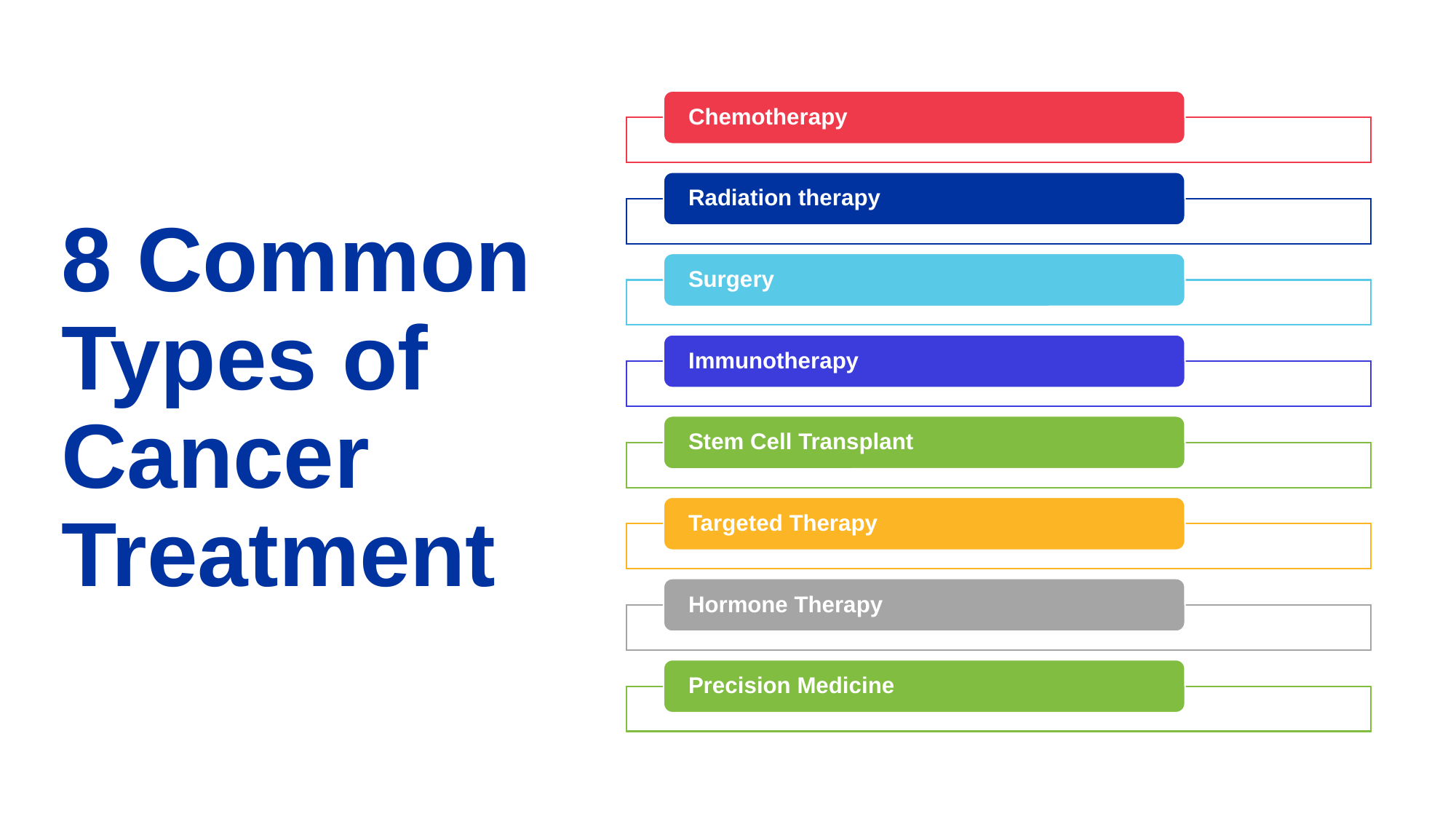

# 8 Common Types of Cancer Treatment

## Slide 10
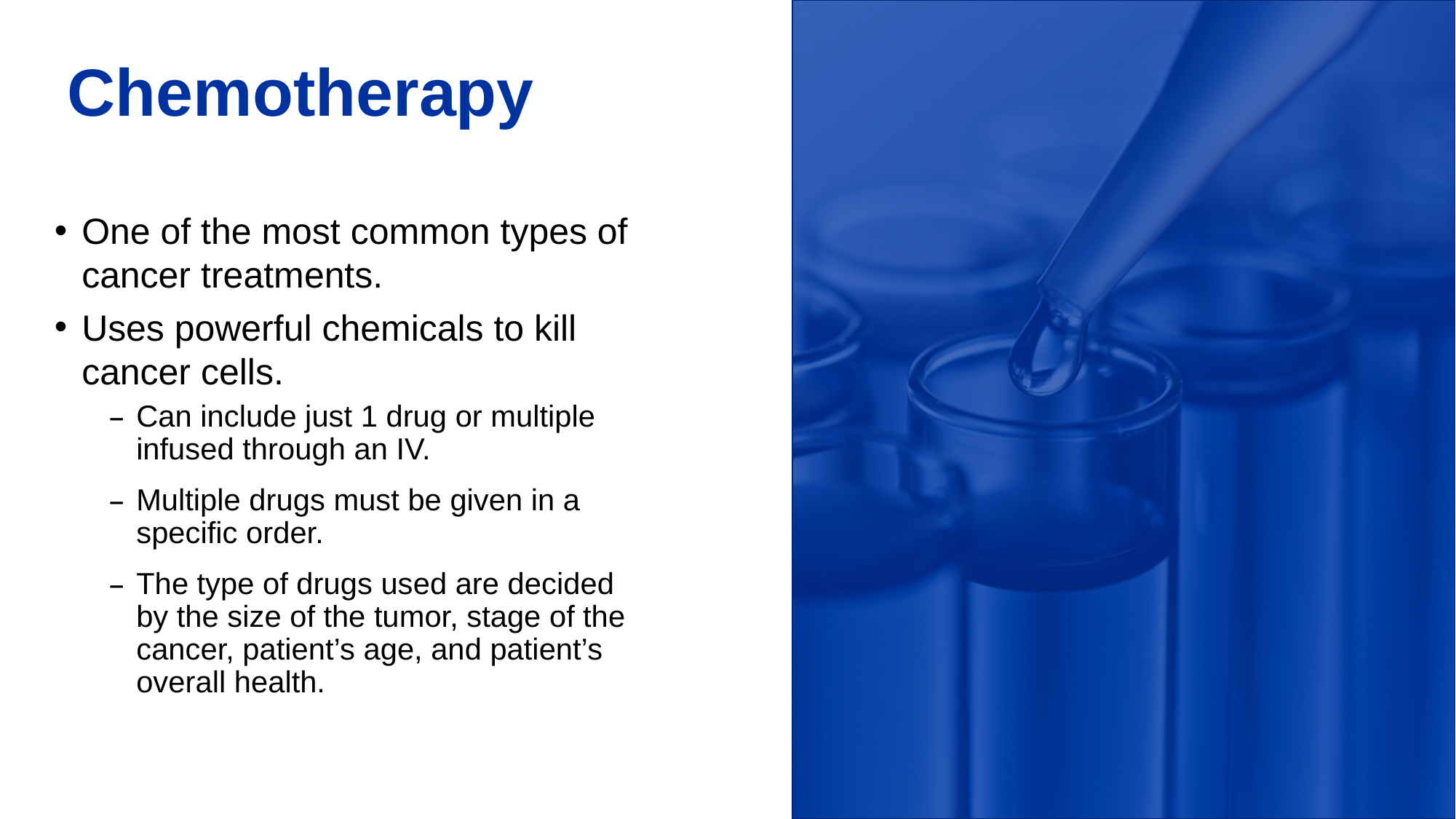

# Chemotherapy
One of the most common types of cancer treatments.
Uses powerful chemicals to kill cancer cells.
Can include just 1 drug or multiple infused through an IV.
Multiple drugs must be given in a specific order.
The type of drugs used are decided by the size of the tumor, stage of the cancer, patient’s age, and patient’s overall health.

## Slide 11
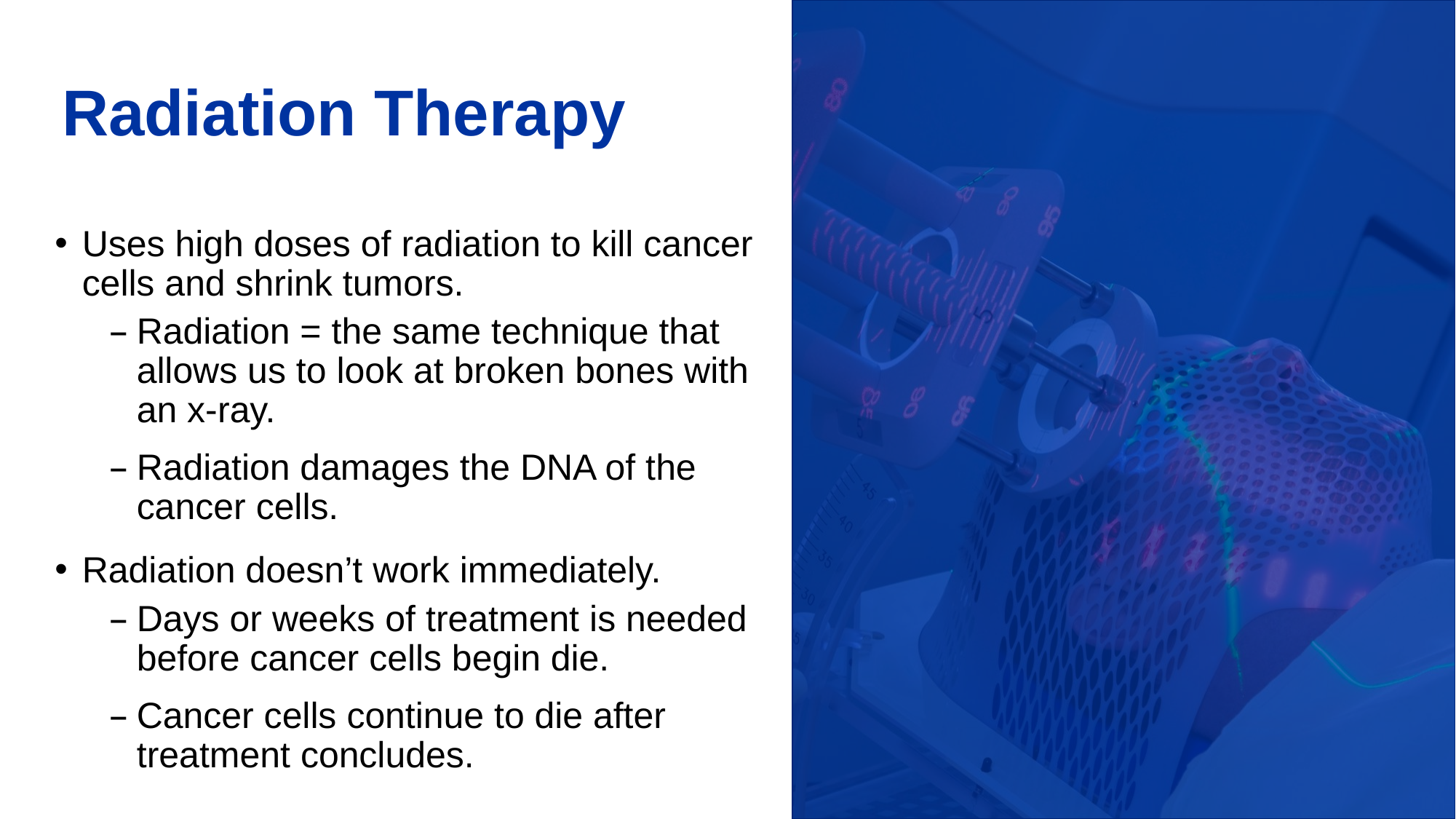

# Radiation Therapy
Uses high doses of radiation to kill cancer cells and shrink tumors.
Radiation = the same technique that allows us to look at broken bones with an x-ray.
Radiation damages the DNA of the cancer cells.
Radiation doesn’t work immediately.
Days or weeks of treatment is needed before cancer cells begin die.
Cancer cells continue to die after treatment concludes.

## Slide 12
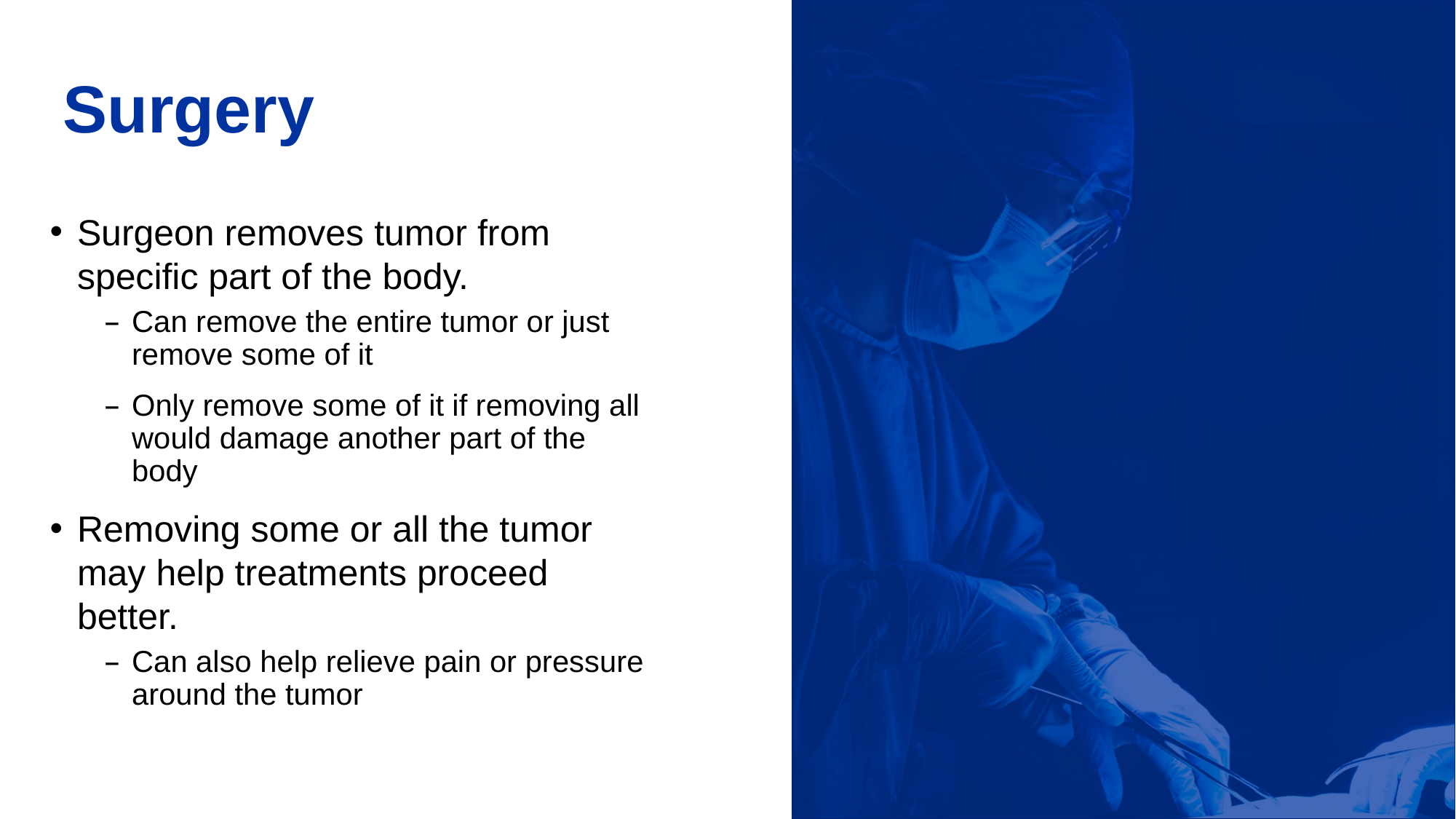

# Surgery
Surgeon removes tumor from specific part of the body.
Can remove the entire tumor or just remove some of it
Only remove some of it if removing all would damage another part of the body
Removing some or all the tumor may help treatments proceed better.
Can also help relieve pain or pressure around the tumor

## Slide 13
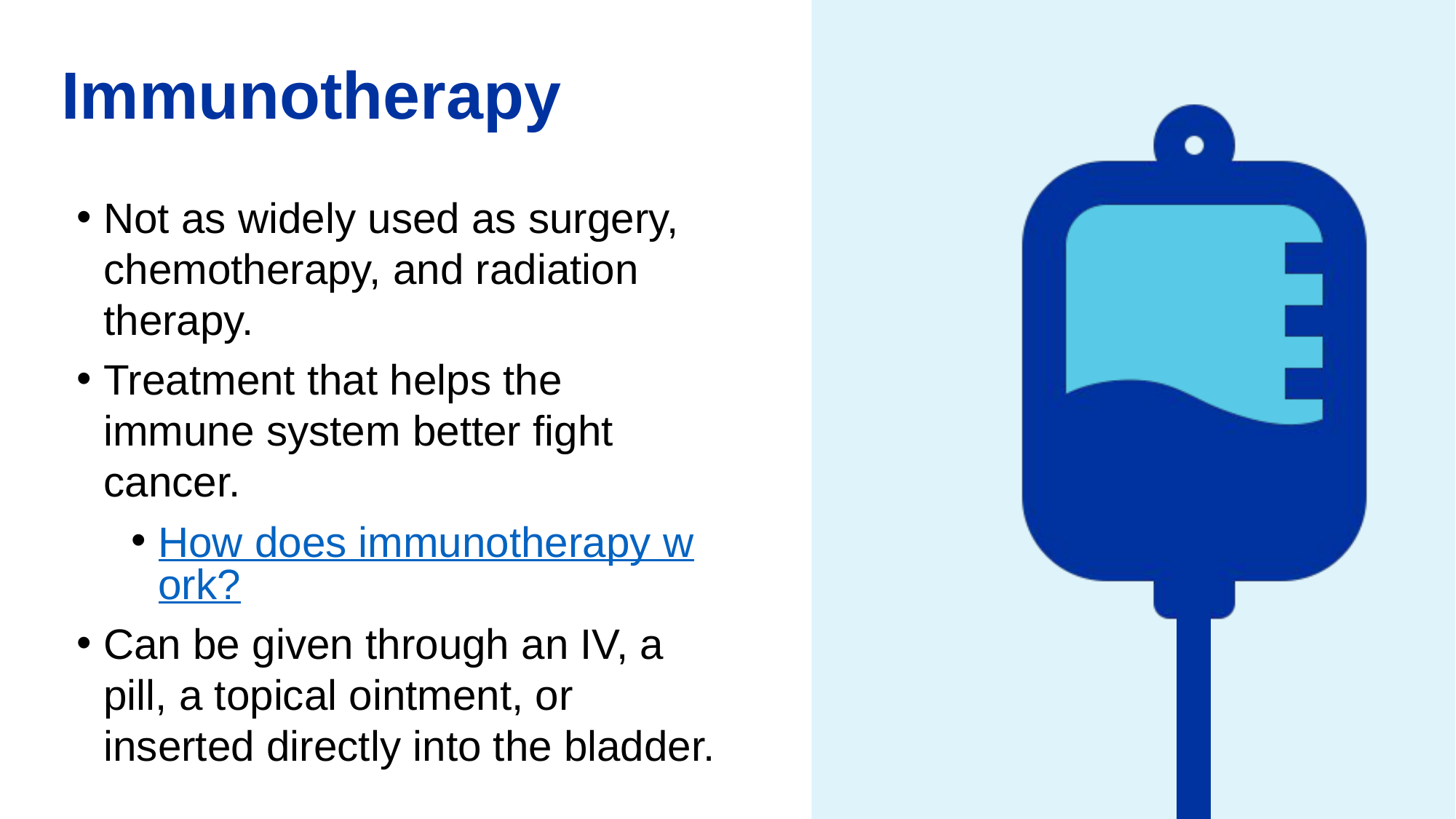

# Immunotherapy
Not as widely used as surgery, chemotherapy, and radiation therapy.
Treatment that helps the immune system better fight cancer.
How does immunotherapy work?
Can be given through an IV, a pill, a topical ointment, or inserted directly into the bladder.

## Slide 14
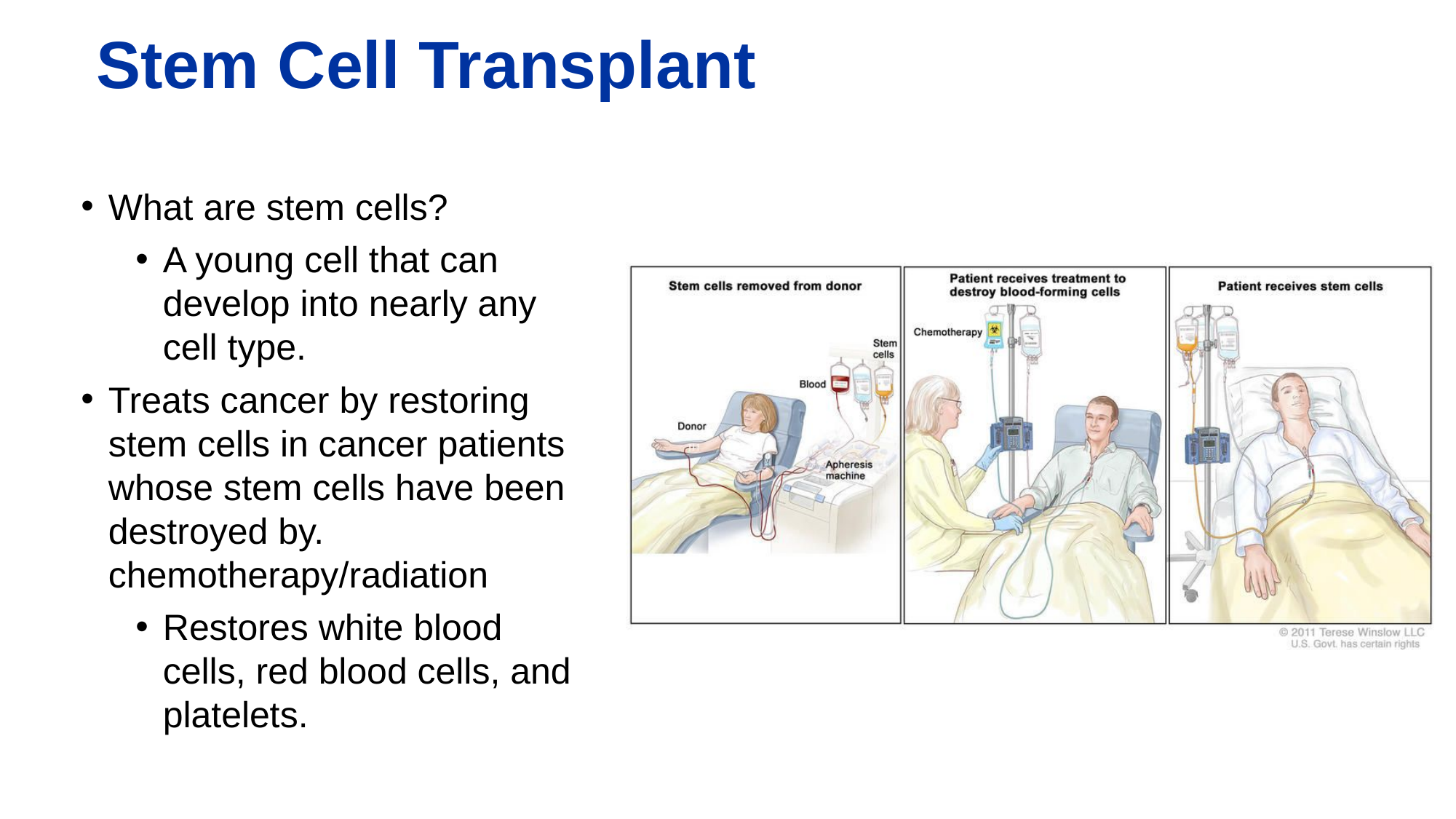

# Stem Cell Transplant
What are stem cells?
A young cell that can develop into nearly any cell type.
Treats cancer by restoring stem cells in cancer patients whose stem cells have been destroyed by. chemotherapy/radiation
Restores white blood cells, red blood cells, and platelets.

## Slide 15
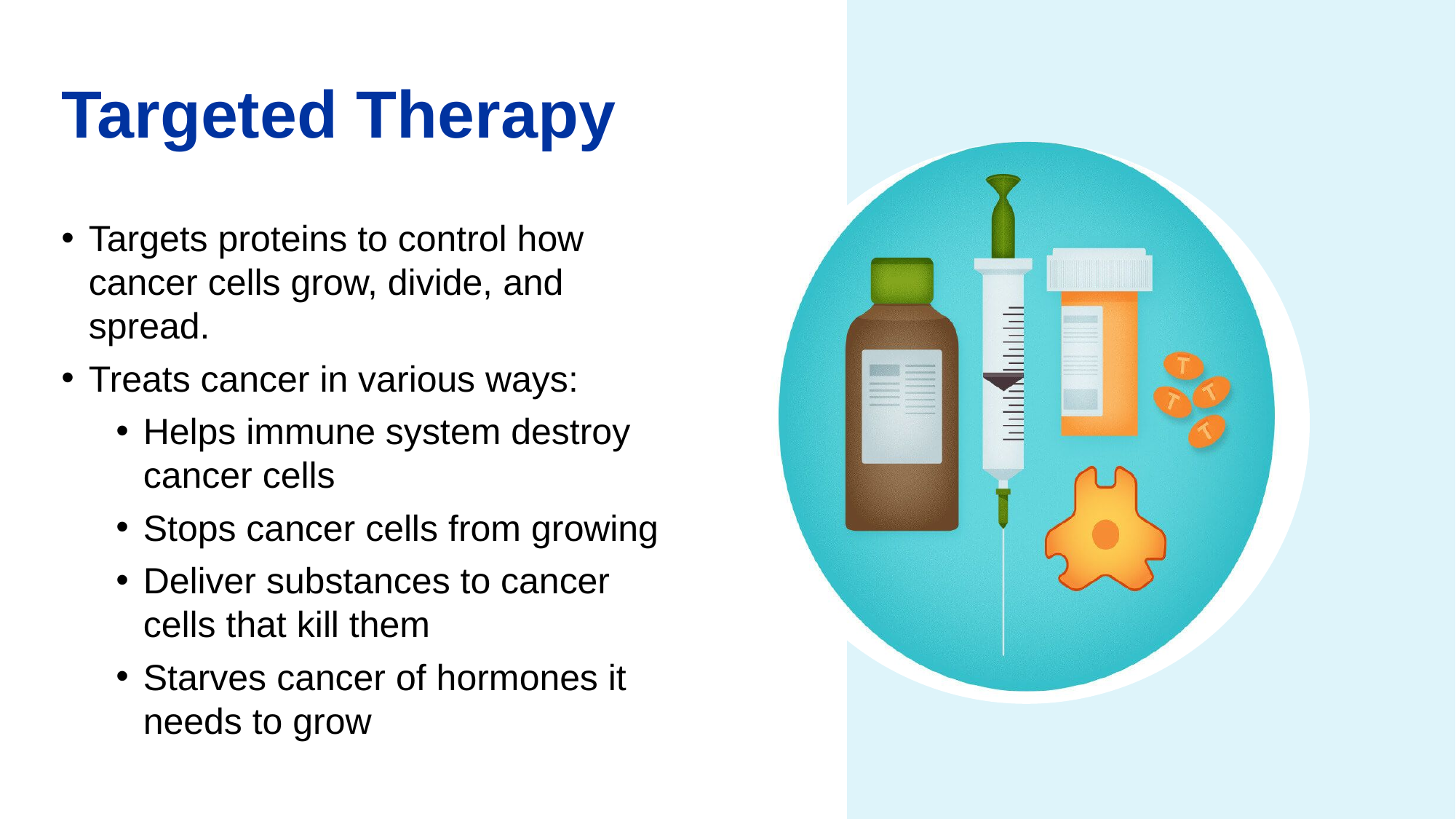

# Targeted Therapy
Targets proteins to control how cancer cells grow, divide, and spread.
Treats cancer in various ways:
Helps immune system destroy cancer cells
Stops cancer cells from growing
Deliver substances to cancer cells that kill them
Starves cancer of hormones it needs to grow

## Slide 16
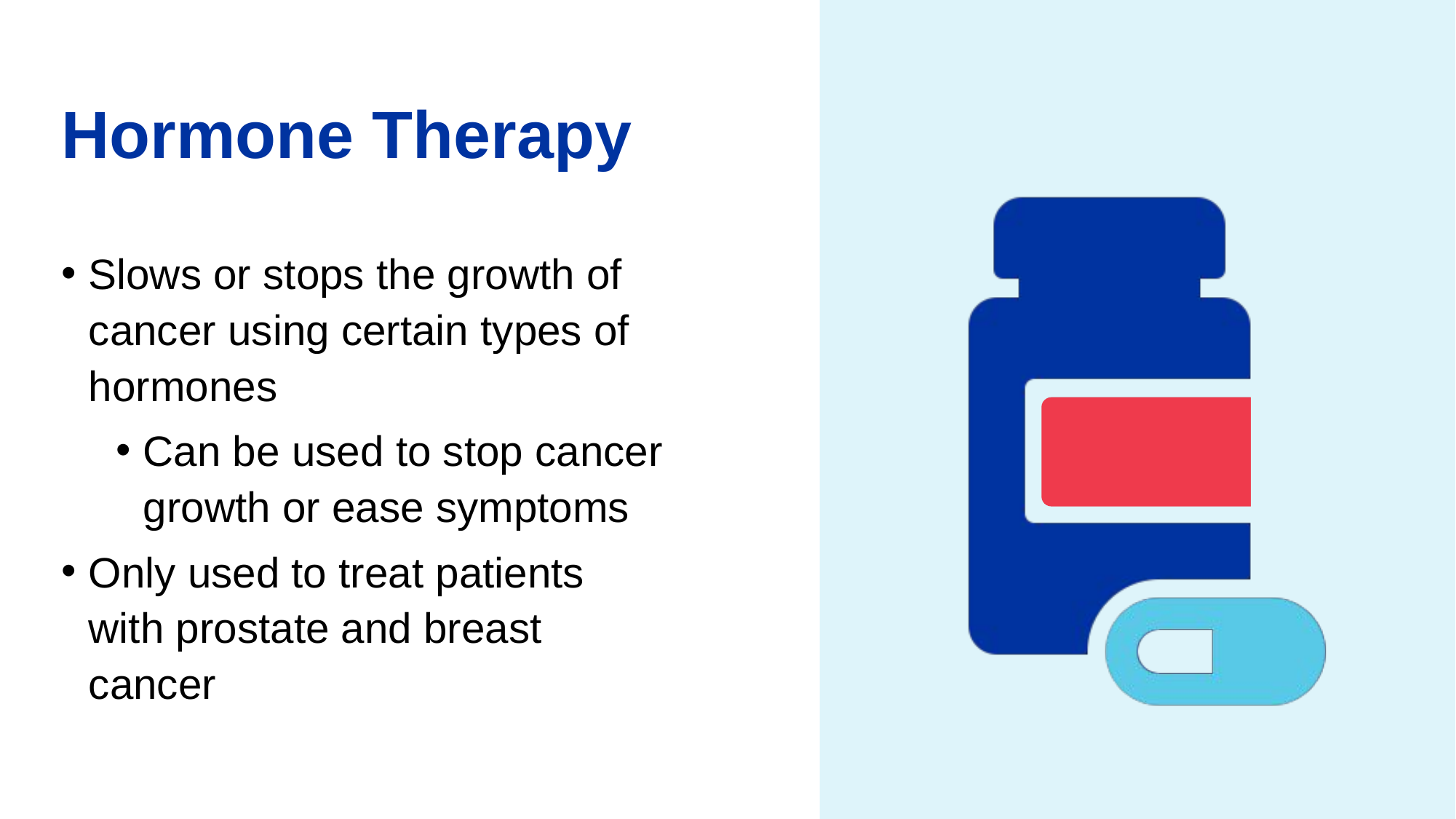

# Hormone Therapy
Slows or stops the growth of cancer using certain types of hormones
Can be used to stop cancer growth or ease symptoms
Only used to treat patients with prostate and breast cancer

## Slide 17
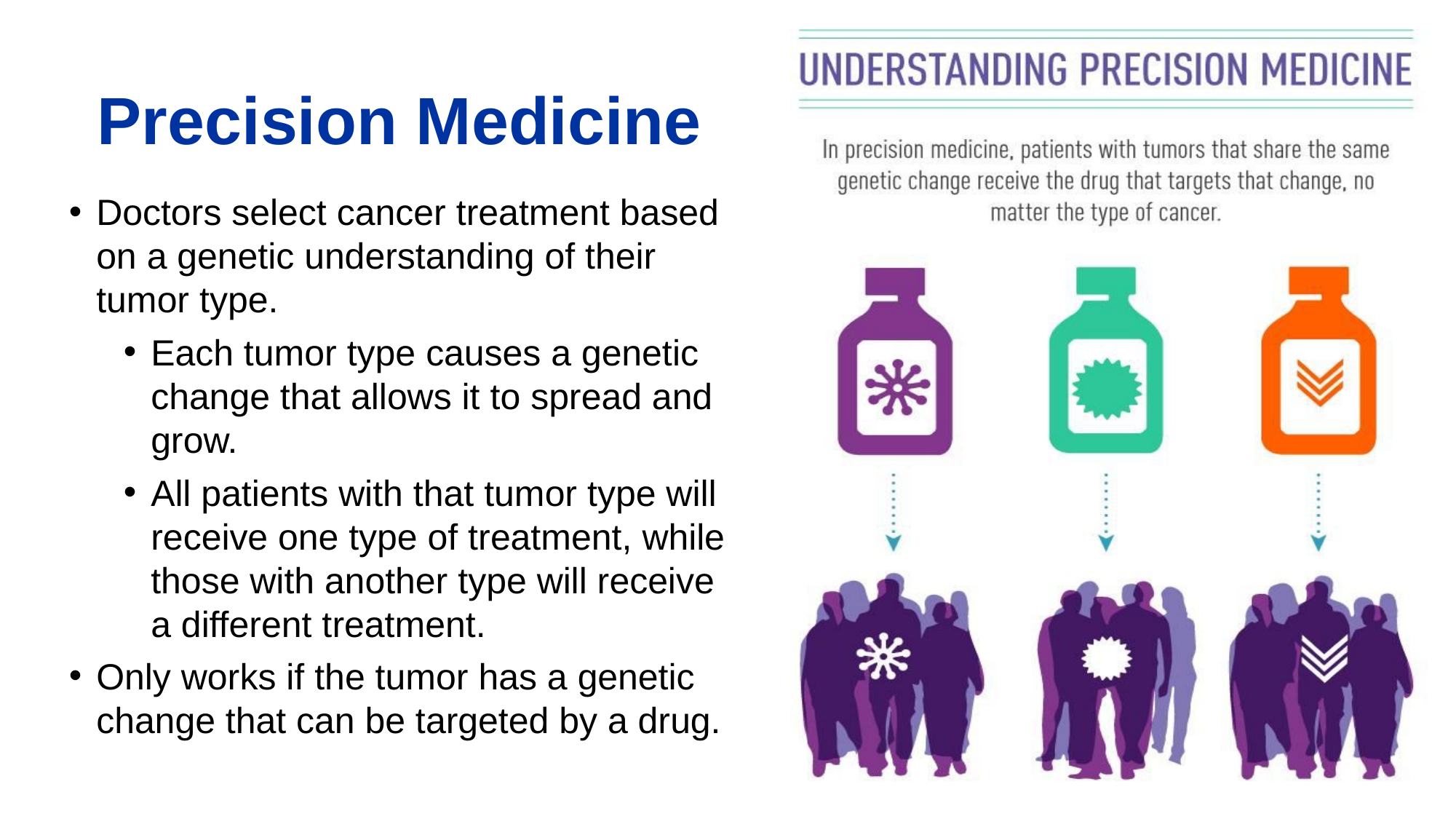

# Precision Medicine
Doctors select cancer treatment based on a genetic understanding of their tumor type.
Each tumor type causes a genetic change that allows it to spread and grow.
All patients with that tumor type will receive one type of treatment, while those with another type will receive a different treatment.
Only works if the tumor has a genetic change that can be targeted by a drug.

## Slide 18
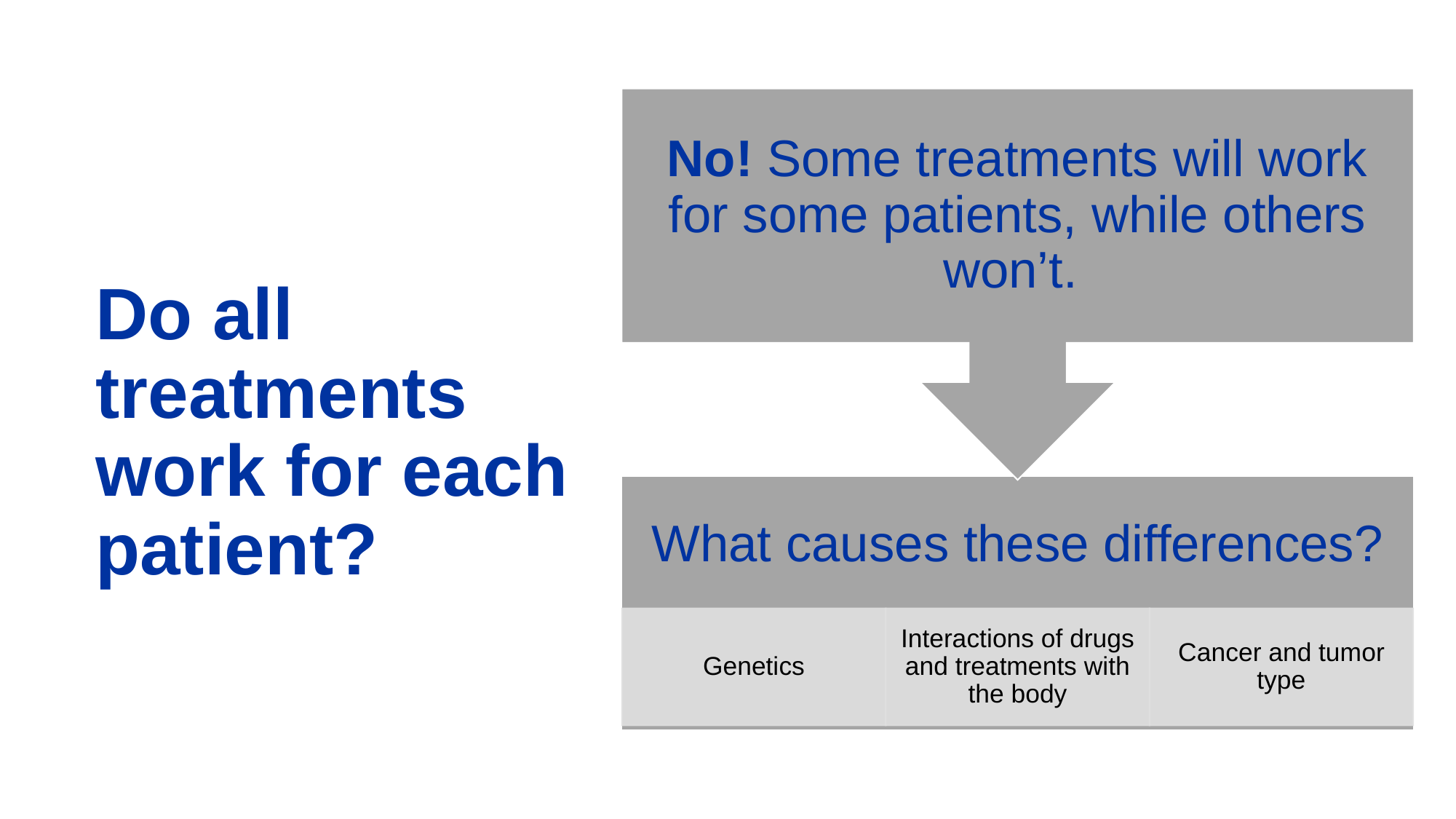

# Do all treatments work for each patient?

## Slide 19
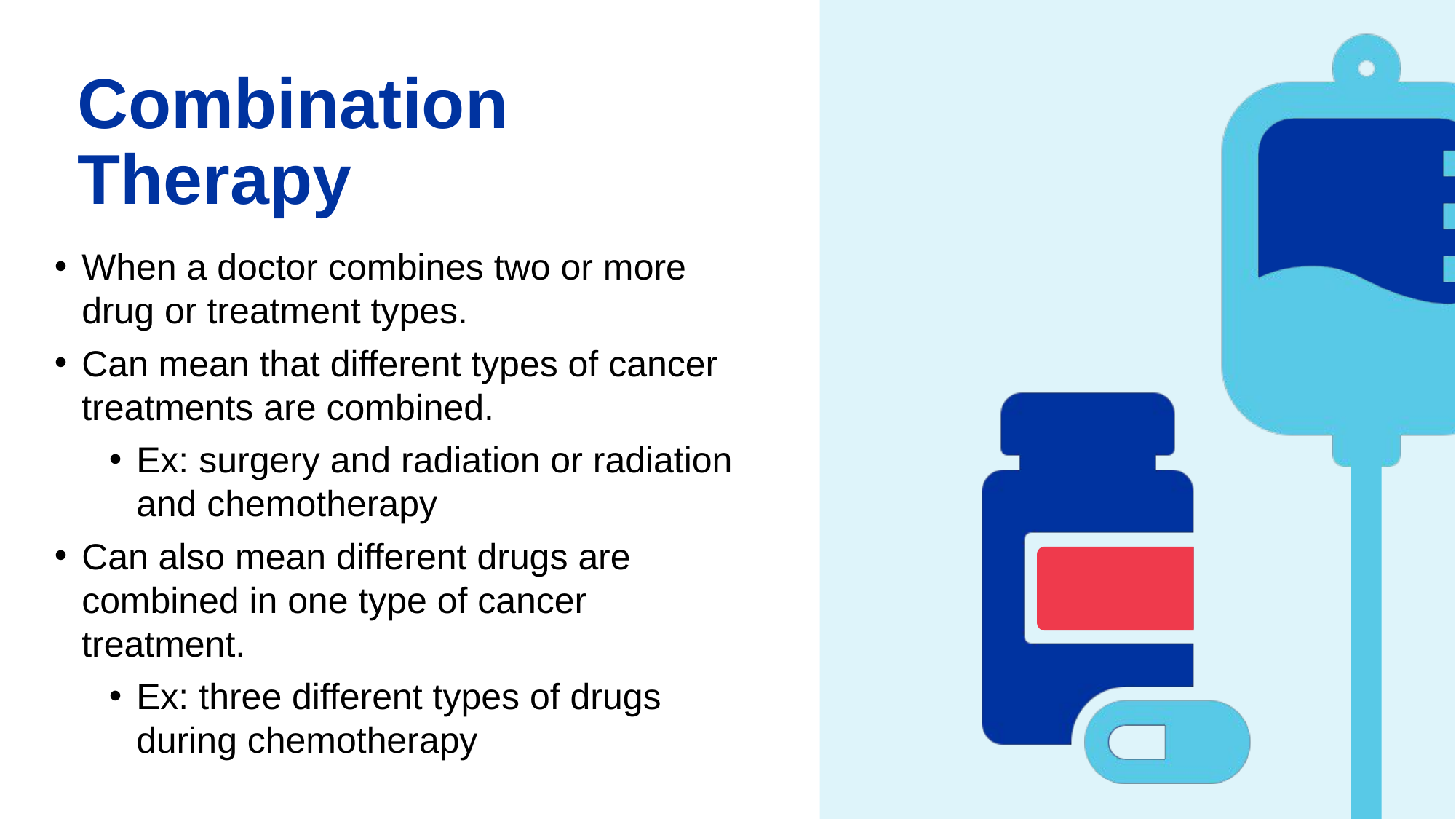

# Combination Therapy
When a doctor combines two or more drug or treatment types.
Can mean that different types of cancer treatments are combined.
Ex: surgery and radiation or radiation and chemotherapy
Can also mean different drugs are combined in one type of cancer treatment.
Ex: three different types of drugs during chemotherapy

## Slide 20
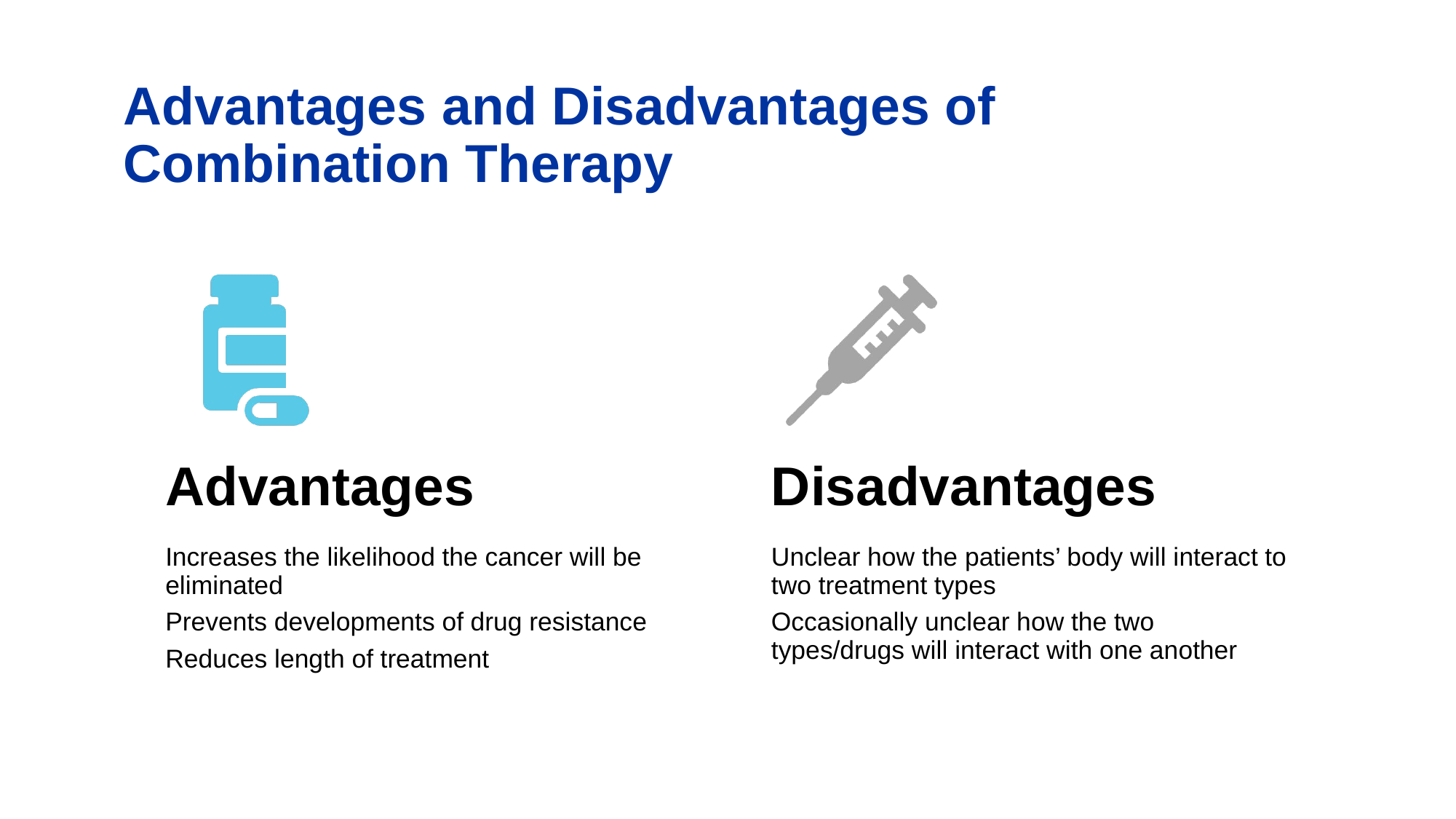

# Advantages and Disadvantages of Combination Therapy

## Slide 21
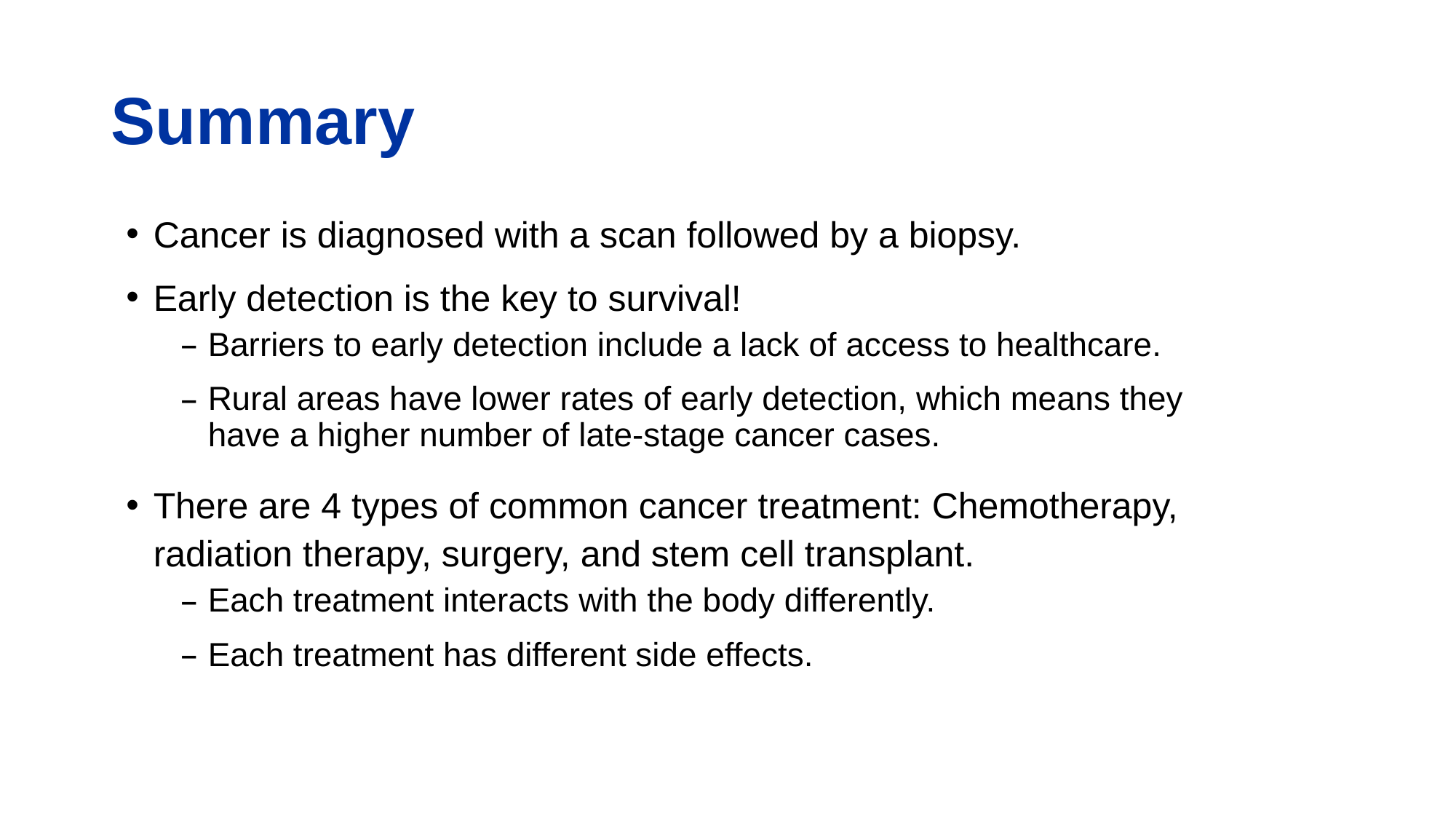

# Summary
Cancer is diagnosed with a scan followed by a biopsy.
Early detection is the key to survival!
Barriers to early detection include a lack of access to healthcare.
Rural areas have lower rates of early detection, which means they have a higher number of late-stage cancer cases.
There are 4 types of common cancer treatment: Chemotherapy, radiation therapy, surgery, and stem cell transplant.
Each treatment interacts with the body differently.
Each treatment has different side effects.

## Slide 22
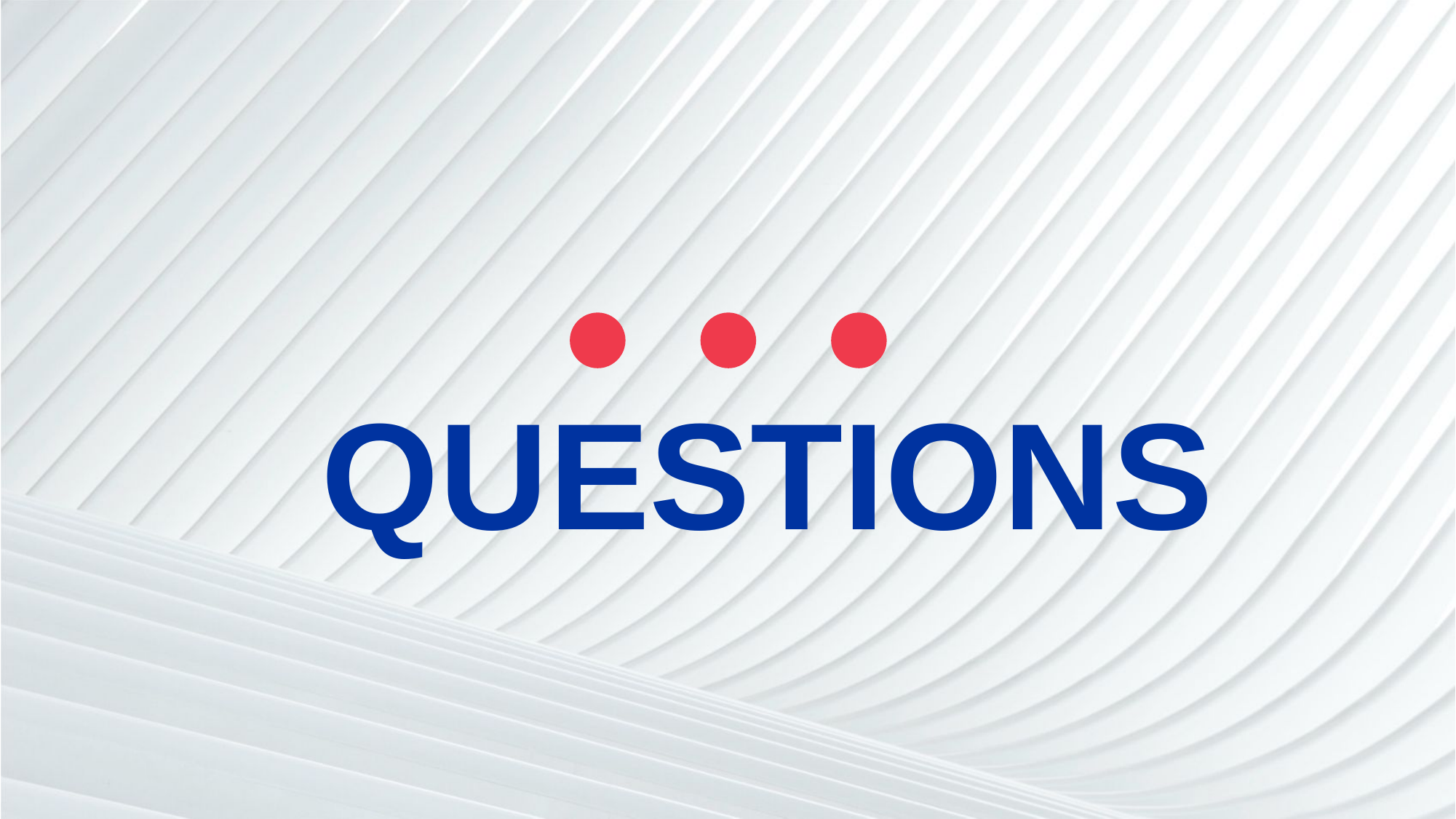

# QUESTIONS
