# Supplementary material for: Cancer Curriculum for Appalachian Kentucky Middle and High Schools
Source: J Appalach Health. 2021 Jan 24;3(1):43–55. doi: 10.13023/jah.0301.05 (PMC8830599; doi:10.13023/jah.0301.05)
Supplement: Supplementary file 7 [file Appendix8-3.1.5Hudson.pdf]

LESSON PLAN OUTLINE

Lesson: Cancer in the Commonwealth - Lesson 1: Cancer Basics

Grade Level: Kentucky middle and high school students

Length: 30-45 minutes

I. Objective:

To encourage students to think critically about cancer, its development, and its disparities within the context of Kentucky and their community.

II. Standards:

Middle School Next Generation Science Standards:

MS-LS1-1: Conduct an investigation to provide evidence that living things are made of cells; either one cell or many different numbers and types of cells. (Cross-Cutting Concepts (CCC): Scale, Proportion, and Quantity; Connections to Nature of Science: Interdependence of Science, Engineering, and Technology).

Science and Engineering Practice (SEP): Planning and carrying out investigations

MS-LS1-5: Environmental and Genetic Growth Factors - Construct a scientific explanation based on evidence for how environmental and genetic factors influence the growth of organisms. (CCC: Cause and Effect)

SEP: Constructing Explanations and Designing Solutions

High School Next Generation Science Standards:

HS-LS1-4: Use a model to illustrate the role of cellular division (mitosis) and differentiation in producing and maintaining complex organisms. (CCC: Systems and System Models)

SEP: Developing and Using Models

HS-LS3-2: Make and defend a claim based on evidence that inheritable genetic variations may result from: (1) new genetic combinations through meiosis, (2) viable errors occurring during replication, and/or (3) mutations caused by environmental factors. (CCC: Cause and Effect)

SEP: Engaging in Argument from Evidence

HS-LS3-3: Apply concepts of statistics and probability to explain the variation and distribution of expressed traits in a population. (CCC: Scale, proportion, and quantity; Connections to Nature of Science: Science is a Human Endeavor)

SEP: Analyze data using tools, technologies, and/or models (e.g., computational, mathematical) in order to make valid and reliable scientific claims or determine an optimal design solution.

Kentucky Academic Standards for Health Education:

Standard 1: Students will comprehend content related to health promotion and disease prevention to enhance health

Standard 2: Analyze the influence of family, peers, culture, media, technology and other factors on health behaviors.

Standard 3: Access valid information, products and services to enhance health.

Standard 4: Use interpersonal communication skills to enhance health and avoid or reduce health risks.

Standard 5: Use decision-making skills to enhance health.

Standard 6: Use goal-setting skills to enhance health.

Standard 7: Practice health-enhancing behaviors and avoid or reduce health risks.

Standard 8: Advocate for personal, family and community health.

III. Preparation

Purpose:

To teach Kentucky middle and high school students what cancer is, how it develops, how it is diagnosed, and where it is most prominent in the United States.

Materials:

Cancer in the Commonwealth, Lesson 1: Cancer Basics and Disparities PowerPoint
White board/SmartBoard
Map of the United States
Map of Kentucky

IV. Procedure

A. Initial Engagement

The first thing you need to do is motivate the students to want to learn this material. To do this, it is important to establish a personal connection with the topic by encouraging students to consider their existing background knowledge or an experience in their lives related to the subject. Share the relevance of the lesson to students' lives. This can be completed while the title slide to the

PowerPoint is up on the screen. One way you can do this is by asking for a show of hands as to how many students have known/do know someone with cancer at the beginning of the lesson. If a student feels comfortable, they can share a quick anecdote about how cancer has affected their life.

B. Pretest Survey

For this curriculum, students must complete both a pre and posttest questionnaire to gauge their knowledge before and after this lesson. Please have students complete the questionnaire before the lesson and then again after you have delivered the lesson. The questions are listed at the end of this lesson plan under "evaluation." The pre and posttests are identical to one another.

C. Body of the Lesson/Input:

This section will go through the PowerPoint slide by slide to provide additional information and sources for each point.

Slide 2: This slide teaches students the basics of cancer biology. A cancer cell can form when a cell begins undergoing uncontrollable division. This uncontrollable division can be caused by mutations in the cell. If the cell signaling pathway is not properly regulated, the cell can enter mitosis (cell division) gain before it is scheduled to do so. It may also miss a signal that tells it to undergo apoptosis (cell death), so it does not die when scheduled.

References:
https://www.cancer.gov/about-cancer/understanding/what-is-cancer

Slide 3 and 4: This activity helps students understand how age affects cell mutations. Who is more likely to develop cancer? The older couple is more likely to develop cancer because as the body ages, the cells accumulate more mutations. This makes it more likely that one of these mutations will lead to uncontrollable division, the formation of a tumor, and a cancerous lesion.

Slide 5: This slide follows up the activity, asking students to look critically at a graph that compares a person's age with the lifetime risk of developing cancer. As a person ages, the number of stem cell divisions increases. As the number of stem cell divisions increases, the lifetime risk of developing cancer also increases. Have students brainstorm and share about this correlation before explaining directly.

References:
https://www.biointeractive.org/classroom-resources/cell-division-and-cancer-risk

Slide 6: This slide helps students understand what metastasis is and why it is important to cancer diagnoses. Metastasis is when cancer cells migrate and spread to another area of the body. This migration does not change the type of cancer. Breast cancer is still breast cancer even if it has metastasized to the bones. Lung cancer is still lung cancer even if it has metastasized to the brain. Some cancers metastasize faster than others. Two of the fastest migrating cancers are pancreatic and liver, but the rate of metastasis also depends on the patient and their body. It's different for everyone.

References:

https://www.cancer.net/navigating-cancer-care/cancer-basics/what-metastasis

Slide 7: Compare the two types of tumors. Use the diagram to help the explanation. Note how the malignant tumor cells have busted through their existing membrane and are beginning to invade other areas.

References:
https://www.verywellhealth.com/what-does-malignant-and-benign-mean-514240

Slide 8: This slide helps students understand the different types of cancer. Cancer is named in two main ways: after the organ/tissue where it originates or after the cell type. Typically, cancers named after cell types are skin cancers, like carcinoma or melanoma, or blood cancers, like leukemia. The most common type of cancer is breast, with lung and prostate following closely behind.

References:
https://www.cancer.gov/about-cancer/understanding/what-is-cancer#cancer-spreads

Slide 9: This slide helps students understand how cancer is diagnosed. There are 3 steps to diagnosing cancer. 1) Cancers are caught when a patient comes in for a regularly scheduled screening or when they are showing symptoms. 2) An oncologist completes a biopsy where they remove a tissue sample before sending it to the lab for analysis. 3) A lab specialist will examine the cells to see if they are abnormally shaped or sized. If so, they may be cancerous. Note: Use this slide to discuss the importance of obtaining cancer screenings, as not all patients show symptoms. By the time they do show symptoms, the cancer may be more difficult or nearly impossible to treat.

References: https://www.cancer.org/treatment/understanding-your-diagnosis/tests/testing-biopsy-and-cytology-specimens-for-cancer/how-is-cancer-diagnosed.html

Slide 10: This slide helps a student understand how a lab specialist would decide if a cell was cancerous by looking at it under a microscope. Cancerous cells will be numerous and abnormally shaped/sized. The cell boundary may be blurred or eliminated altogether. They will also have a larger nucleus:cytoplasm ratio, meaning that there will be a larger nucleus with less cytoplasm. They may also have a different number of chromosomes.

Reference:
https://www.verywellhealth.com/cancer-cells-vs-normal-cells-2248794

Slide 11: This slide shows pictures of cells under a microscope. The picture on the left shows normal pancreatic cells, while the picture on the right shows pancreatic carcinoma. After completing slide 10, students can discuss which picture they believe shows cancerous cells. Remind them to use the characteristics from the previous slide to support their answer.

References:
https://bmccancer.biomedcentral.com/articles/10.1186/1471-2407-4-73

Slide 12 and 13: This activity is designed to help students understand that not all areas of the country and state are affected equally by cancer. Allow them to brainstorm which areas may be more affected. Ask them why they believe the areas they marked see higher numbers of cancer cases and discuss as a class.

Slide 14: This slide teaches students about how economic class and cancer risk are correlated. People who receive fewer years of education have higher cancer rates than those with more years. People with a high school degree or less, on average, make less money than those with a college degree. Less income can mean an inability to pay for healthcare coverage if it is not provided by the employer. Additionally, those with less education tend to have to work more hours to support their family, which can lead to healthcare neglect. They may choose working an extra shift over receiving a cancer screening.

References:
https://www.ncbi.nlm.nih.gov/pmc/articles/PMC2711979/

Slide 15: This slide teaches students about how race and cancer risk are correlated. Black populations see the highest number of cancer cases and deaths in the US. Some of this disparity can be attributed to genetic predisposition. Genetic predisposition means that someone is more likely to develop cancer due to something in their genes. Due to generations of health inequities, some black populations have an engrained mistrust of the healthcare system, making it less likely that they will obtain regular screenings and proper treatment. Lastly, black populations also tend to have lower education levels and less access to health insurance, which also contributes to the low rates of early detection in these communities. Note: If time allows, this is a good slide to stimulate a discussion between classmates. Before explaining, ask them how mistrust of the healthcare system could lead to increased cancer cases and deaths.

References: https://www.mdanderson.org/cancerwise/race-and-ethnicity-as-cancer-risk-factors.h00-158592156.html
https://fortune.com/2020/06/30/black-women-breast-cancer-mortality-racism-healthcare-pandemic/
Q&A: https://www.cancer.net/blog/2020-06/cancer-does-not-affect-all-people-equally-expert-qa-cancer-disparities-and-health-equity

Slide 16: This slide lists the states with the lowest number of cancer cases in the US. Ask the students: does there appear to be a correlate between income per capita and cancer cases?

References:
https://www.cdc.gov/cancer/dcpc/data/index.htm

Slide 17: This slide lists the states with the highest number of cancer cases in the US. Kentucky has the highest number of cancer cases and deaths in the US. Ask the students: is their prediction of the correlation between income per capita and cancer cases affirmed or negated by this information?

References:
https://www.cdc.gov/cancer/dcpc/data/index.htm

Slide 18: This slide describes how some regions of Kentucky are more heavily affected by cancer. Nationwide, rural residents are more likely to develop cancer. Appalachian residents are so heavily affected because of lifestyle choices (smoking, obesity, diet), lack of access to healthcare (fewer facilities, hazardous roads), and low education/socioeconomic levels. Why these factors increase cancer risk will be discussed more in lesson 2.

Slide 19: This slide summarizes what has been covered in lesson 1. Ask if students have clarifying questions before moving into the discussion period.

D. Discussion Questions:

These questions are designed to help students think more critically about the information presented in the PowerPoint. Time permitting, we recommend having them discuss in pairs or small groups before beginning a large group discussion, but you could also go straight to the large group discussion if necessary. Additional information is provided below each question for you to tell students after the discussion.

1.) Is there a genetic component to developing cancer?
    a. Yes! The most commonly mutated gene in people with cancer is p53 or TP53. Most of these mutations occur in somatic cells and cannot be passed down to offspring, but some occur in germline cells. Cancer caused by germline mutations is called inherited cancer, and it accounts for 5-20% of all cancers.
    b. Reference: https://www.cancer.net/navigating-cancer-care/cancer-basics/genetics/genetics-cancer
2.) Are all areas of the country affected equally? Which populations are *less* affected by cancer?
    a. No! White populations, those with higher education levels, and those with higher economic class are less likely to develop cancer.
3.) Create a hypothesis as to why certain areas of the United States are more at risk for developing cancer. Write this hypothesis down and put it somewhere safe. You can see if your hypothesis is correct during the next lesson!

V. Evaluation

Teacher evaluation
Please complete the following evaluation after you have taught the lesson.

- What were the strengths of the lesson?
- What worked well?
- What were problem areas?
- How could you improve the lesson?
- What could you do differently if you were to teach it again?
- What is an alternate way to present the same material?
- Do you have any other comments regarding your experience teaching the lesson?

Student evaluation

1.) What is cancer?
    a. Cancer is a disease caused by mutations that leads to uncontrolled cell growth.
    b. Cancer is a virus that causes abnormal formations in the body.
    c. Cancer is a bacterial infection that causes abnormal processes in the body.
    d. Cancer is a metabolic disorder that causes changes in metabolism.
    e. Cancer is a mental disorder that causes changes in emotions.

2.) A benign tumor is cancerous.
 a. True
 b. False
3.) When cancer has metastasized is means it has:
 a. Spread to other parts of the body
 b. Spread to other parts of the originally affected organs
 c. Stopped spreading
 d. Been cured
 e. None of the above
4.) A biopsy of a tumor is done to:
 a. Remove it
 b. Diagnose it
 c. Treat it
 d. Cure it
 e. None of the above
5.) How does Kentucky compare to other states in cancer rates?
 a. Kentucky is 15th in overall cancer incidence and mortality rates
 b. Kentucky is 1st in the nation in overall cancer incidence and mortality rates
 c. Kentucky has the lowest overall cancer incidence and mortality rates
 d. Kentucky has the same cancer incidence and mortality rates as other states
 e. None of the above
6.) Which race is most affected by cancer?
 a. Black populations
 b. Hispanic populations
 c. White populations
 d. Native American populations
7.) What is the correlation between economic standing and cancer cases?
 a. Households who are in the highest economic tier have higher cancer incidence.
 b. Households who are in the middle economic tier have higher cancer incidence.
 c. Households who are in the lowest economic tier have higher cancer incidence.
8.) How do cancer cell differ from regular cells?
 a. Cancerous cells will be sparse and normally shaped/sized.
 b. Cancerous cell boundaries will be clearly defined.
 c. Cancerous cells will have a larger nucleus:cytoplasm ratio
 d. Cancerous cells may have the same number of chromosomes.
9.) The type of cancer changes after the cancer has metastasized.
 a. True
 b. False
10.) How are cancers named?
 a. After the organ/tissue where it originates
 b. After the cell type
 c. Both A and B
 d. Neither A or B

Student Evaluation Answer Key

1) A

2) B
3) A
4) B
5) B
6) A
7) C
8) C
9) B
10) C
