# Supplementary material for: Cancer Curriculum for Appalachian Kentucky Middle and High Schools
Source: J Appalach Health. 2021 Jan 24;3(1):43–55. doi: 10.13023/jah.0301.05 (PMC8830599; doi:10.13023/jah.0301.05)
Supplement: Supplementary file 8 [file Appendix9-3.1.5Hudson.pdf]

LESSON PLAN OUTLINE

Lesson: Cancer in the Commonwealth - Lesson 2: Risk factors and modifiable behaviors

Grade Level: Kentucky middle and high school students

Length: 30-45 minutes

I. Objective:

To encourage students to think critically about cancer, its risk factors, and modifiable behaviors within the context of Kentucky and their community.

II. Standards

Middle School Next Generation Science Standards:

MS-LS3-1: Develop and use a model to describe why structural changes to genes (mutations) located on chromosomes may affect protein and may result in harmful, beneficial, or neutral effects to the structure and function of an organism (Cross-Cutting Concepts (CCC): Structure and Function).

SEP: Developing and Using Models

High School Next Generation Science Standards:

HS-LS1-1: Construct an evidence-based explanation for how the structure of DNA determines the structure of proteins, which carry out the essential functions of life through systems of specialized cells (CCC: Structure and Function).

SEP: Constructing explanations and Designing Solutions

HS-LS3-1: Ask questions to clarify relationships about the role of DNA and chromosomes in coding the instructions for characteristic traits passed from parents to offspring (CCC: Cause and Effect)

SEP: Asking Questions and Defining Problems

HS-LS3-2: Make and defend a claim based on evidence that inheritable genetic variation may result from: (1) new genetic combinations through mitosis, (2) viable errors occurring during replication, and/or (3) mutations caused by environmental factors (CCC: Cause and Effect).

SEP: Engaging in Argumentation from Evidence

Kentucky Academic Standards for Health Education:

Standard 1: Students will comprehend content related to health promotion and disease prevention to enhance health

Standard 2: Analyze the influence of family, peers, culture, media, technology and other factors on health behaviors.

Standard 3: Access valid information, products and services to enhance health.

Standard 4: Use interpersonal communication skills to enhance health and avoid or reduce health risks.

Standard 5: Use decision-making skills to enhance health.

Standard 6: Use goal-setting skills to enhance health.

Standard 7: Practice health-enhancing behaviors and avoid or reduce health risks.

Standard 8: Advocate for personal, family and community health.

III. Preparation

Purpose:

To teach Kentucky middle and high school students what makes a person more likely to develop cancer and which behaviors to avoid in order to decrease their risk.

Materials:

Cancer in the Commonwealth, Lesson 2: Risk Factors and Modifiable Behaviors PowerPoint
White board/SmartBoard

IV. Procedure

A. Initial Engagement

The first thing you need to do is remind students of what they learned in lesson 1. Share what cancer is, how it develops, and some of its disparities in the United States. Emphasize Kentucky's high cancer rates in order to create a personal connection and remind them why this topic is so important to Appalachia Kentucky in particular. This can be completed on slide 2 of the PowerPoint.

B. Pretest Survey

For this curriculum, students must complete both a pre and posttest questionnaire to gauge their knowledge before and after this lesson. Please have students complete the questionnaire before the lesson and then again after you have delivered the lesson. The questions are listed at the end of this lesson plan under “evaluation.” The pre and posttests are identical to one another.

C. Body of the Lesson/Input:

This section will go through the PowerPoint slide by slide to provide additional information and sources for each point.

Slide 3 and 4: These slides encourage students to brainstorm behaviors that can increase one’s risk of developing cancer. This activity will follow the claim, evidence, reasoning (CER) model. Have each group create a list of behaviors and write their responses, then compile them all on the board. Ask each group to explain why one of their behaviors increases a person’s risk of developing cancer. As you go through the PowerPoint, you can put a check mark by which behaviors increase cancer risk.

Slide 5: This slide explains what a risk factor is. Risk factors, which increase someone’s likelihood of developing a disease, are not always within someone’s control. Genetics, for example, are outside of someone’s control, but many risk factors (ex: smoking, not using sunscreen, poor diet) are within someone’s control.

Reference:
https://www.medicinenet.com/script/main/art.asp?articlekey=5377

Slide 6: This slide lists each of the risk factors that you will discuss in class. You do not need to go into any detail in this slide because the rest of the presentation will focus on each of these behaviors in-depth.

Slide 7: The slide discusses age as a cancer risk factor. As you touched on in lesson 1, older people have a greater chance of developing cancer. 25% of cancer cases are people aged 65-74, and cancer cases increase as individuals age. This occurs because DNA mutations increase with age, making it more likely that a cell will mutate and become cancerous.

References:
https://www.cancer.gov/about-cancer/causes-prevention/risk/age
https://files.eric.ed.gov/fulltext/ED596266.pdf
https://genetics.thetech.org/original_news/news91

Slide 8: This slide discusses alcohol use as a cancer risk factor. Alcohol is an irritant that can cause cell damage, leading to DNA mutations that cause cancer when cells try to repair themselves. Because all alcohols contain ethanol, the type of alcohol does not have an effect on a person’s cancer risk. However, as a person drinks more alcohol than recommended, their risk of developing cancer increases as well.

Reference:

https://www.cancer.gov/about-cancer/causes-prevention/risk/alcohol

Slide 9: This slide discusses immunosuppression as a cancer risk factor. Immunosuppression is when the activation and efficacy of a person's immune system is reduced. Their immune system is very weak, which means their cells are less likely to be able to detect and destroy cancer cells. Specifically, patients who have received an organ transplant are very at risk because they are likely taking immunosuppressive drugs to ensure their body doesn't reject the organ.

Reference:
https://www.cancer.gov/about-cancer/causes-prevention/risk/immunosuppression

Slide 10: This slide discusses environmental carcinogens as a cancer risk factor. A carcinogen is a substance that promotes the formation of cancer, so increased exposure leads to DNA mutations that can lead to uncontrollable cell division. Common carcinogens are pollution, radon, asbestos, heavy metals, and engine exhaust. One carcinogen that is specific to Kentucky is coal. Coal is a very important resource in KY, but excessive exposure to its mining and exhaust can increase the risk of developing a cancerous mutation.

Reference:
https://www.cancer.gov/about-cancer/causes-prevention/risk/substances

Slide 11: This slide discusses hormones as a cancer risk factor. Estrogen and progesterone are hormones that are important for reproductive development in women. Too much of these hormones increase a woman's risk of developing breast cancer. Some women receive hormone replacement therapy to control menopause symptoms, but this therapy puts women at an extremely higher risk of developing breast cancer. Higher hormone levels affect how fast cells divide, making it more likely for a cancer-causing mutation to occur.

Reference:
https://www.cancer.gov/about-cancer/causes-prevention/risk/hormones

Slide 12: This slide discusses obesity as a cancer risk factor. Kentucky is the fifth most obese state in the United States, which leads to a variety of health defects, one of which is cancer. Being overweight increases the risk of nearly all cancer types for three reasons: 1.) Excessive fat starves cells of oxygen, triggering inflammation and increasing the risk for mutations long-term. 2.) High insulin levels stimulate increased cell division and inhibit cell death, increasing the risk for cancerous development. 3.) High cytokine levels drive inflammation and mutations. Cytokines are proteins that are secreted by the immune system to help fight infection.

Reference:
https://www.cancer.gov/about-cancer/causes-prevention/risk/obesity

Slide 13: This slide discusses radiation as a cancer risk factor. Radiation is most commonly associated with X-rays but is also present in gamma rays and neutrons. Excessive exposure can increase a person's risk of developing cancer because it causes DNA damage and can create mutations that lead to cancer, which is why patients must wear lead vests. Lead vests help to prevent too much radiation from entering the body and causing DNA damage.

Reference:
https://www.cancer.gov/about-cancer/causes-prevention/risk/radiation

Slide 14: This slide discusses exposure to the UV rays of the sun as a cancer risk factor. This risk factor is relevant for Kentucky because many farmers and blue-collar workers in KY either forget or choose not to wear sunscreen despite all the hours they spend in the sun. UV rays cause damage to skin cells, resulting in mutations that can cause uncontrollable division and can lead to skin cancer. Artificial sunlight, such as tanning beds, can cause similar damage.

Reference:
https://www.cancer.gov/about-cancer/causes-prevention/risk/sunlight

Slide 15: This slide discusses tobacco use as a cancer risk factor. This risk factor is also relevant to Kentucky because the state relies so heavily on the tobacco industry. As a result, a very high percentage of Kentuckians smoke compared to the national average even though it is the leading cause of cancer death. Tobacco products contain chemicals that can cause DNA damage and create mutations that lead to various types of cancer. Chewing tobaccos can result in increased risk of mouth and esophageal cancers, and cigarette smoke can result in increased risk of lung cancer.

Reference:
https://www.cancer.gov/about-cancer/causes-prevention/risk/tobacco

Slide 16: This slide discusses infectious diseases as a cancer risk factor. Viruses such as Epstein-Barr, Hepatitis, HIV, and HPVs can increase a person's risk of developing cancer for various reasons. Some viruses disrupt cell signaling, which alters the process of cell division and leads to uncontrollable growth. Others weaken the immune system, similar to immunosuppressive drugs, and make it difficult to fight off cancer-causing infections. Some can cause chronic inflammation, resulting in increased risk of mutation.

Reference:
https://www.cancer.gov/about-cancer/causes-prevention/risk/infectious-agents

Slide 17: This slide introduces a three-step plan to alleviate cancer risk. There is no way to completely prevent cancer risk, but there are things you can do to lessen your risk. These next few slides will go into more detail about these steps.

Slide 18: This slide introduces step 1: Avoid. Students (and adults, too) should avoid substances and behaviors that increase the risk for cancer-causing DNA mutations. Substances to avoid include tobacco, alcohol, and carcinogens when possible. Behaviors to avoid include receiving excessive sun exposure while not wearing sunscreen or going to a tanning bed.

Slide 19: This slide introduces step 2: Develop. Those trying to alleviate cancer risk should develop lifestyle habits like a healthy diet and a sustainable exercise regimen. These habits have a variety of positive health impacts, but they also prevent obesity and decrease the likelihood of developing DNA mutations.

Slide 20: This slide introduces the final step: Act. Those trying to alleviate cancer risk should, above all else, educate themselves. The best way to educate oneself is to seek out books, pamphlets, and presentations on cancer and to share this information with everyone else! They should also schedule necessary appointments and follow-ups with their doctors and obtain

preventative cancer screenings. Preventative screenings can catch cancer before it metastasizes, thus improving the survival rates of cancer patients!

Slide 21: This slide summarizes the cancer risk factors and the three-step plan to alleviate cancer risk. Emphasize that students should share this information with others to improve the cancer outlook in Kentucky.

D. Discussion Questions:

These questions are designed to help students think more critically about the information presented in the PowerPoint. Time permitting, we recommend having them discuss in pairs or small groups before beginning a large group discussion, but you could also go straight to the large group discussion if necessary. Additional information is provided below each question for you to tell students after the discussion.

1.) Which person would be more likely to develop cancer: A 20-year-old who avoids excessive alcohol consumption or a 70-year-old alcoholic? Explain your answer.
    a. A 20-year old who avoids excessive alcohol consumption. The 70-year-old alcoholic has two risk factors that increase his chances of developing cancer: age and alcohol consumption. The 20-year-old has none!
2.) Which person would be more likely to develop cancer: a 35-year-old who uses tobacco, but has a healthy diet or a 60-year-old who doesn't use tobacco, but eats junk food and has been exposed to excessive radiation?
    a. This is a tougher one, but it would most likely be the 60-year-old. He has 2 risk factors that increase his chances of developing cancer (age and radiation), while the 35-year-old only has 1 (tobacco).
3.) Using what you learned for lesson 1, choose a risk factor and explain why it makes one more likely to develop cancer.
    a. There are lots of answers for this one! Encourage students to incorporate what they learned about cancer developing from lesson 1 into their answer.

V. Evaluation

Teacher evaluation

Please complete the following evaluation after you have taught the lesson.

- What were the strengths of the lesson?
- What worked well?
- What were problem areas?
- How could you improve the lesson?
- What could you do differently if you were to teach it again?
- What is an alternate way to present the same material?
- Do you have any other comments regarding your experience teaching the lesson?

Student evaluation

1.) What are common cancer risk factors?
   a. Age
   b. Carcinogens including environmental factors
   c. Obesity
   d. Viruses/infectious agents
   e. All of the above
2.) What are some lifestyle choices that increase one's likelihood of developing cancer?
   a. Smoking
   b. Unhealthy diet
   c. Risky behaviors
   d. All of the above
   e. None of the above
3.) Cancer can impact populations or groups of people (for example, men versus women) differently?
   a. True
   b. False
4.) Which of the following are ways that infectious diseases can increase cancer risk?
   a. Viruses disrupt cell signaling, which alters the process of cell division and leads to uncontrollable growth.
   b. Viruses weaken the immune system and make it difficult to fight off cancer-causing infections.
   c. Viruses can cause chronic inflammation, resulting in increased risk of mutation.
   d. All of the above
   e. None of the above
5.) Which person would be *less* likely to develop cancer: A 60-year-old man who smokes and eats poorly or a 30-year-old alcoholic who doesn't smoke and has a healthy diet?
   a. 60-year-old man who smokes and eats poorly
   b. 30-year-old alcoholic who doesn't smoke and has a healthy diet
6.) Which behaviors should a person avoid if they are trying to decrease their cancer risk?
   a. Chewing tobacco
   b. Sunscreen use
   c. Regular exercise
   d. A balanced diet
7.) Regular exercise decreases the risk of obesity and, as a result, increases the risk of developing cancer.
   a. True
   b. False
8.) What is immunosuppression?
   a. When a person's immune system is overactive/strong
   b. When a person's immune system is underactive/weak
   c. When a person's immune system does not work at all
   d. When a person's immune system attacks its own cells
9.) Why are women receiving hormone therapy at a higher risk of developing cancer?
   a. Higher hormone levels affect how fast cells divide, making it more likely for a cancer-causing mutation to occur.

b. Higher hormone levels cause a person's immune system to slow down, making them less likely to be able to fight off cancerous cells.
c. Higher hormone levels can make a person lose their appetite, causing them to lose weight.
d. Higher hormone levels cause damage to skin cells, making it more likely for a skin cancer-causing mutation to occur.

10.) Where does Kentucky rank with respect to obesity?

a. 1st
b. 50th
c. 5th
d. 10th

Student evaluation Answer Key

1) E
2) D
3) A
4) D
5) B
6) A
7) B
8) B
9) A
10) C
