# Supplementary material for: Cancer Curriculum for Appalachian Kentucky Middle and High Schools
Source: J Appalach Health. 2021 Jan 24;3(1):43–55. doi: 10.13023/jah.0301.05 (PMC8830599; doi:10.13023/jah.0301.05)
Supplement: Supplementary file 10 [file Appendix11-3.1.5Hudson.pdf]

LESSON PLAN OUTLINE

Lesson: Cancer in the Commonwealth - Lesson 3, Part 1: Common Treatments

Grade Level: Kentucky middle and high school students

Length: 30 minutes

I. Objective:

To encourage students to think critically about cancer and its treatments within the context of Kentucky and their community.

II. Standards

Middle School Next Generation Science Standards (Engineering Design):

MS-ETS1-1: Define the criteria and constraints of a design problem with sufficient precision to ensure a successful solution, taking into account relevant scientific principles and potential impacts on people and the natural environment that may limit possible solutions.

SEP: Asking Questions and Defining Problems

MS-ETS1-2: Evaluate competing design solutions using a systematic process to determine how well they meet the criteria and constraints of the problem.

SEP: Constructing Explanations and Designing Solutions

High School Next Generation Science Standards (Engineering Design):

HS-ETS1-1: Analyze a major global challenge to specify qualitative and quantitative criteria and constraints for solutions that account for societal needs and wants.

SEP: Asking Questions and Defining Problems

HS-ETS1-2: Design a solution to a complex real-world problem by breaking it down into smaller, more manageable problems that can be solved through engineering.

SEP: Constructing Explanations and Designing Solutions

HS-ETS1-3: Evaluate a solution to a complex real-world problem based on prioritized criteria and trade-offs that account for a range of constraints, including cost, safety, reliability, and aesthetics, as well as possible social, cultural, and environmental impacts.

SEP: Using Mathematics and Computational Thinking

Kentucky Academic Standards for Health Education:

Standard 1: Students will comprehend content related to health promotion and disease prevention to enhance health.

Standard 2: Analyze the influence of family, peers, culture, media, technology and other factors on health behaviors.

Standard 3: Access valid information, products and services to enhance health.

Standard 4: Use interpersonal communication skills to enhance health and avoid or reduce health risks.

Standard 5: Use decision-making skills to enhance health.

Standard 6: Use goal-setting skills to enhance health.

Standard 7: Practice health-enhancing behaviors and avoid or reduce health risks.

Standard 8: Advocate for personal, family and community health.

III. Preparation

Purpose:

To teach Kentucky middle and high school students about how different cancer treatments work and which are the most commonly used.

Materials:

Cancer in the Commonwealth, Lesson 3, Part 1: Treatment PowerPoint
Computers/telephones for students to complete individual research
White board/SmartBoard

IV. Procedure

A. Initial Engagement

The first thing you need to do is remind students of what they learned in lessons 1 and 2. Share about what cancer is, its disparities, and its risk factors in the United States. Emphasize Kentucky's high cancer rates and modifiable behaviors in order to create a personal connection and remind them why this topic is so important to Appalachia Kentucky in particular. This can be completed on the title slide of the PowerPoint.

B. Pretest Survey

For this curriculum, students must complete both a pre and posttest questionnaire to gauge their knowledge before and after this lesson. Please have students complete the questionnaire before part 1 of lesson 2 and then again after you have delivered part 2 of lesson 2. The questions are listed at the end of this lesson plan under “evaluation.” The pre and posttests are identical to one another.

C. Body of the Lesson/Input:

This section will go through the PowerPoint slide by slide to provide additional information and sources for each point.

Slide 2 and 3: These slides encourage students to learn about different cancer treatments in small groups. Assign each group a different type of cancer treatment and ask them to research their treatment in-depth. Things to consider when researching are how the treatment works, why it is useful for patients, and what side effects may occur. Pair them with another group and have each group explain to the other how their treatment works. This will give them a good introduction to two types of cancer treatment. It will deepen their understanding of the assigned cancer treatment by learning about it and then teaching it to another group.

Slide 4: This slide reviews a concept from lesson 1: how cancer is diagnosed. Cancer is diagnosed using a scan followed by a biopsy. A biopsy is a surgical procedure where a doctor removes a small sample of cells in the tumor and sends it to a lab for closer analysis. A researcher will then look at the sample under a microscope to see if it is cancerous. Use the diagram to remind students of the difference between normal and cancerous cells. Treatment can only begin after the cancer has been properly diagnosed.

Reference: https://www.cancer.org/treatment/understanding-your-diagnoses/tests/testing-biopsy-and-cytology-specimens-for-cancer/how-is-cancer-diagnosed.html

Slide 5: This slide discusses the concept of early detection. Early detection has been achieved when the cancer is found when it is small and hasn’t metastasized yet. When cancer is this early in its development, there are rarely signs/symptoms that would lead a person to receive an out-of-the-ordinary screening. Typically, early detection is achieved through a regularly scheduled cancer screening, such as a colonoscopy or mammogram.

Reference:
https://www.cancer.org/healthy/find-cancer-early.html

Slide 6: This slide discusses the importance of early detection. A cancer that is small and has not spread is much easier to treat and control than one that is large and has metastasized. Cancers that have metastasized often require intensive treatment and still don’t always recede. Early detection greatly improves a patient’s chances of surviving.

Reference:
https://www.cancer.gov/about-cancer/screening

Slide 7: The slide discusses early detection in Kentucky. Despite the importance of early detection, not enough citizens actually participate in preventative screenings. 70% of at-risk patients are up to date on GI cancer screenings, which begin at 50; 64% of at-risk women are up to date on mammograms, which begin at 40; 10.3% of at-risk lung cancer patients are up to date on lung cancer screenings, which begin at 55. Emphasize that, ideally, these percentages should be as close to 100% as possible!

Reference:
htttps://www.lung.org/research/state-of-lung-cancer/states/Kentucky

Slide 9: This slide discusses what may occur in regions where early detection is not widespread. Early detection is not as common in rural areas as it is in urban area. Similarly, patients living in rural areas have a greater chance of being diagnosed with later stage cancers. This can be contributed, in part, by a lack of access to healthcare services. Specifically, Appalachia Kentucky has very limited access to cancer care facilities, with the nearest facility being located in Lexington at the Markey Cancer Center. This can greatly affect their ability to obtain screenings and their treatment regimen following diagnosis.

Reference:
https://pubmed.ncbi.nlm.nih.gov/19780916/

Slide 9: This slide discusses barriers to early detection in Appalachia Kentucky. As your students may know, the nearest cancer care facility in rural areas can be 2-3 hours away on hazardous roads. Additionally, adults may not be able to take off work in order to travel to receive care for fear of not being paid or losing their job. They may need the money to pay for food, clothes, or other necessities. As a result, regular doctor visits may be pushed back in favor of more immediate, pressing matters.

Reference:
https://pubmed.ncbi.nlm.nih.gov/19780916/

Slide 10: This slide lists the 4 types of common cancer treatments that will be discussed in the following slides. There is no need to spend much time on this slide, as each treatment will be covered in-depth in the coming slides.

Reference:
https://www.cancer.org/treatment

Slide 11: This slide introduces one of the most common and well-known types of cancer treatment: chemotherapy (chemo). When most students think of cancer treatment, they likely think of chemotherapy. They have likely seen someone they know undergo chemotherapy, resulting in hair loss and fatigue. Chemotherapy uses powerful chemicals to kill cancer cells. The chemicals (either just 1 or a cocktail of different chemicals) are infused through an IV. If multiple chemicals are used, they must be given in a specific order to properly disable the cancer cells. The type and quantity of drugs are decided by the size and stage of the tumor and the patient's age and overall health. If the cancer is smaller and has not metastasized, the chemicals will not be as powerful because they are not necessary to kill the cancer cells. If the patient is older and not healthy, the drugs may not be as powerful for fear of the chemotherapy becoming fatal.

Reference: https://www.cancer.org/treatment/treatments-and-side-effects/treatment-types/chemotherapy/how-is-chemotherapy-used-to-treat-cancer.html

Slide 12: This slide compares two different types of chemotherapy. Neoadjuvant chemo is used to shrink a tumor before another treatment type is used to kill it entirely. Whereas neoadjuvant chemo is used as the first line of defense, adjuvant chemo is used following a different type of treatment. Adjuvant chemo kills any remaining cancer cells after a different treatment, such as radiation or surgery, has already shrunk the tumor.

Reference: https://www.cancer.org/treatment/treatments-and-side-effects/treatment-types/chemotherapy/how-is-chemotherapy-used-to-treat-cancer.html

Slide 13: This slide discusses the side effects of chemotherapy. Because chemotherapy uses chemicals to kill cancer cells, it is very common for these chemicals to also damage normal cells. This is why many chemotherapy patients lose their hair early into the treatment. This damage also causes easy bruising/bleeding, fatigue, changes in appetite, nausea/vomiting, and fluctuations in weight. It's important to mention that this is not a comprehensive list of side effects. Not all patients will experience all symptoms, and some may experience side effects that are not on this list.

Reference: https://www.cancer.org/treatment/treatments-and-side-effects/treatment-types/chemotherapy/how-is-chemotherapy-used-to-treat-cancer.html

Slide 14: This slide discusses radiation therapy as a cancer treatment. Radiation therapy uses high doses of radiation to kill cancer cells and shrink tumors. Interestingly, radiation is also a cancer risk factor because it causes mutations in cells that can lead to cancerous development. The benefits of radiation therapy differ for each patient, and doctors must decide prior to prescribing treatment whether or not the benefits outweigh the risks. Radiation also does damage to the DNA of cancer cells, effectively killing them after continuous treatment. Even after treatment concludes, cells continue to die.

Reference: https://www.cancer.gov/about-cancer/treatment/types/radiation-therapy

Slide 15: This slide compares two different types of radiation: external beam and internal beam. External beam administers radiation from outside of the body. With external beam radiation, the machine does not have to touch the patient at all. With internal beam radiation, however, the radiation is administered through an IV to target the area near the tumor directly. Which type of radiation a doctor recommends is dependent on the type of cancer and resources available.

Reference: https://www.cancer.gov/about-cancer/treatment/types/radiation-therapy

Slide 16: This slide discusses the side effects of radiation therapy. Similar to chemotherapy, radiation damages other, normal cells in the body, which leads it to have many of the same side effects as chemotherapy. However, depending on the area where the radiation is administered, it can also lead to site-specific side effects. In head and neck cancer, radiation can lead to dry mouth and tooth decay. In gastrointestinal cancer, radiation can lead to nausea and diarrhea.

Reference: https://www.cancer.net/navigating-cancer-care/how-cancer-treated/radiation-therapy/side-effects-radiation-therapy

Slide 17: This slide introduces surgery as a cancer treatment. Many students are familiar with the idea of surgery, so it is important to frame it within the context of cancer. Surgery as a cancer treatment occurs when surgeons remove part or all of the tumor in one area of the body. Ideally, the surgeon would remove all the tumor, but in some situations, removing all of the tumor may lead to life-threatening damage to other organs. In these situations, the surgeon will only remove part of the tumor, which can help the treatments proceed faster and smoother. It can also help relieve pain and pressure in the area of the tumor, leading to increased comfort for the patient.

Reference: https://www.cancer.gov/about-cancer/treatment/types/surgery

Slide 18: This slide compares two different surgical styles. Open surgery is what most students think of when they think of surgery. The surgeon makes a large incision in the area of the tumor to remove it and the surrounding area. In reality, minimally invasive surgery is more common and more desired. Minimally invasive surgery includes the use of cameras, which are inserted into the patient through small incisions. The surgeon uses the cameras to see where the tumor is and removes it using instruments which are inserted through small incision sites simultaneously.

Reference: https://www.cancer.gov/about-cancer/treatment/types/surgery

Slide 19: This slide mentions the side effects of surgery as a cancer treatment. The side effects of cancer surgery are similar to those of other surgeries, and many occur immediately following the surgery while the patient is recovering. These side effects may include site-specific pain, swelling, bruising, and infection. As the body tries to heal itself, the patient may also be excessively tired and experience appetite loss.

Reference: https://www.cancer.net/navigating-cancer-care/how-cancer-treated/surgery/side-effects-surgery

Slide 20: This slide introduces stem cell transplants as a type of cancer treatment. This treatment is often used in conjunction with chemotherapy or radiation. Stem cells, as some of your students may be familiar with, are young cells that have yet to differentiate. This means they are able to develop into nearly any cell type. These stems cells help restore the stem cells of cancer patients who have previously undergone chemotherapy. Because chemotherapy has killed many of the patient’s cells, adding stem cells back into the body helps restore white blood cells, red blood cells, and platelets. This will build the patient’s immune system and help their recovery move faster.

Reference: https://www.cancer.gov/about-cancer/treatment/types/stem-cell-transplant

Slide 21: This slide compares three different types of stem cell transplants. Autologous stem cell transplants occur when the cancer patient themselves is the stem cell donor. The stem cells are taken from different areas of the patient’s body and placed around the cancer site. With allogeneic stem cell transplants, the stem cell donor is someone else. Typically, the donor is a relative of the patient. This is important because the closer related the donor is, the less likely the patient’s body is to reject the stem cell donation. Lastly, syngeneic stem cell transplants occur when the stem cell donor is the patient’s twin. This is an ideal donation scenario because

identical twins share identical DNA, meaning the patient's body is not going to recognize the donation as a foreign body. Instead, it is extremely likely to accept the donation.

Reference:
https://www.cancer.gov/about-cancer/treatment/types/stem-cell-transplant

Slide 22: This slide discusses the most common side effect of a stem cell transplant, known as graft-versus-host disease. This disease occurs when the white blood cells from the donor recognize the cells in the patient's body as foreign and begin to attack the patient's cells. This can cause damage to the cells in the skin, liver, intestines, and many other organs. As discussed early, the more closely related a patient is to their donor, the less likely graft-versus-host disease is to occur.

Reference:
https://www.cancer.gov/about-cancer/treatment/types/stem-cell-transplant

Slide 23: This slide summarizes what has already been covered in the presentation. There is a lot of material in this lesson! Go over this slide carefully to remind students of what they have learned before transitioning into the discussion period.

D. Discussion Questions:

These questions are designed to help students think more critically about the information presented in the PowerPoint. Time permitting, we recommend having them discuss in pairs or small groups before beginning a large group discussion, but you could also go straight to the large group discussion if necessary. Additional information is provided below each question for you to tell students after the discussion.

1.) Why is early detection so important? Try to incorporate Kentucky risk-factors and cancer care obstacles in your answer.
    a. Early detection, in short, increases a patient's chances of surviving cancer. By obtaining regular screenings, it is more likely that the cancer will be diagnosed at an early stage before it has metastasized. Cancer that has not spread is much easier to treat. In Kentucky, early detection and cancer literacy rates are extremely low, even though risk factors are so high. These traits create a perfect storm, causing Kentucky to produce some of the highest cancer rates in the country. Early detection is essential to lessening this disparity.
2.) What could we do to increase cancer screening rates in Kentucky? Brainstorm some solutions to the barriers to early detection in our state.
    a. There are lots of great answers to this question, including (but not limited to) increased funding, increased access to healthcare facilities, increased physician access, increased cancer education, increased cancer literacy, and increased trust of the healthcare system.

V. Evaluation

For this two-part lesson, both the teacher and student evaluations will be completed following

part 2 of the cancer treatment lesson. Encourage students to keep this information fresh in their mind as you transition into the next part.
