# Supplementary material for: Cancer Curriculum for Appalachian Kentucky Middle and High Schools
Source: J Appalach Health. 2021 Jan 24;3(1):43–55. doi: 10.13023/jah.0301.05 (PMC8830599; doi:10.13023/jah.0301.05)
Supplement: Supplementary file 11 [file Appendix12-3.1.5Hudson.pdf]

LESSON PLAN OUTLINE

Lesson: Cancer in the Commonwealth - Lesson 3, Part 2: Rare Treatment

Grade Level: Kentucky middle and high school students

Length: 30 minutes

I. Objective:

To encourage students to think critically about cancer and its treatments within the context of Kentucky and their community.

II. Standards

Middle School Next Generation Science Standards (Engineering Design):

MS-ETS1-1: Define the criteria and constraints of a design problem with sufficient precision to ensure a successful solution, taking into account relevant scientific principles and potential impacts on people and the natural environment that may limit possible solutions.

SEP: Asking Questions and Defining Problems

MS-ETS1-2: Evaluate competing design solutions using a systematic process to determine how well they meet the criteria and constraints of the problem.

SEP: Constructing Explanations and Designing Solutions

High School Next Generation Science Standards (Engineering Design):

HS-ETS1-1: Analyze a major global challenge to specify qualitative and quantitative criteria and constraints for solutions that account for societal needs and wants.

SEP: Asking Questions and Defining Problems

HS-ETS1-2: Design a solution to a complex real-world problem by breaking it down into smaller, more manageable problems that can be solved through engineering.

SEP: Constructing Explanations and Designing Solutions

HS-ETS1-3: Evaluate a solution to a complex real-world problem based on prioritized criteria and trade-offs that account for a range of constraints, including cost, safety, reliability, and aesthetics, as well as possible social, cultural, and environmental impacts.

SEP: Using Mathematics and Computational Thinking

Kentucky Academic Standards for Health Education:

Standard 1: Students will comprehend content related to health promotion and disease prevention to enhance health.

Standard 2: Analyze the influence of family, peers, culture, media, technology and other factors on health behaviors.

Standard 3: Access valid information, products and services to enhance health.

Standard 4: Use interpersonal communication skills to enhance health and avoid or reduce health risks.

Standard 5: Use decision-making skills to enhance health.

Standard 6: Use goal-setting skills to enhance health.

Standard 7: Practice health-enhancing behaviors and avoid or reduce health risks.

Standard 8: Advocate for personal, family and community health.

III. Preparation

Purpose:

To teach Kentucky middle and high school students about how different cancer treatments work and which are the most commonly used.

Materials:

Cancer in the Commonwealth, Lesson 3, Part 2: Treatment PowerPoint
Computers/telephones for students to complete individual research
White board/SmartBoard

IV. Procedure

A. Initial Engagement

The first thing you need to do is remind students of what they learned in part 1 of lesson 3. Remind them of the common types of cancer treatment (chemotherapy, radiation, surgery, and stem cell transplant). Ask students to recall what they know about each of these treatments and their side effects. This can be completed on the title slide of the PowerPoint.

B. Body of the Lesson/Input:

This section will go through the PowerPoint slide by slide to provide additional information and sources for each point.

Slide 2 and 3: These slides encourage students to learn about different cancer treatments in small groups. Assign each group a different type of cancer treatment and ask them to research their treatment in-depth. Things to consider when researching are how the treatment works, why it is useful for patients, and what side effects may occur. Pair them with another group and have each group explain to the other how their treatment works. This will give them a good introduction to two types of cancer treatment. It will deepen their understanding of their assigned cancer treatment by teaching it to another group.

Slide 4: This slide lists the 4 types of rare cancer treatments that will be discussed in the following slides. There is no need to spend much time on this slide, as each treatment will be covered in-depth in the coming slides.

Reference:
https://www.cancer.org/treatment

Slide 5: This slide introduces immunotherapy as a cancer treatment. This treatment assists the immune system in fighting the cancer. The video linked in the PowerPoint will better explain how this treatment works, as visuals are essential in understanding immunotherapy. Immunotherapy can be administered through an IV, a pill, a rub-on ointment, or inserted directly into the bladder for bladder cancer patients.

Reference:
https://www.cancer.gov/about-cancer/treatment/types/immunotherapy

Slide 6: This slide discusses several of the side effects of immunotherapy. When immunotherapy is administered via an IV, there can be negative reactions at the needle prick site, including swelling, pain, soreness, and a rash. It can also lead to flu-like symptoms (fevers, chills, vomiting) and additional side effects like weight gain, irregular heart rhythms, and inflammation.

Reference:
https://www.cancer.gov/about-cancer/treatment/types/immunotherapy/side-effects

Slide 7: This slide introduces targeted therapy as a type of cancer treatment. Targeted therapy targets proteins produced in certain areas of the body to control the growth, division, and spread of cancer cells. This helps treat cancer in 4 ways: 1) It assists the immune system in destroying cancer cells; 2) It stops cancer cells from growing by halting division; 3) It directly delivers substances that are able to kill the cancer cells; 4) It starves cancer cells of the hormones they need to grow, stopping the spread of the disease.

Reference:
https://www.cancer.gov/about-cancer/treatment/types/targeted-therapies

Slide 8: This slide mentions two different types of methods of targeted therapy. Small molecule drugs are small enough to slip through the rigid cell membranes of the cell and enter. Once inside, they can target specific proteins produced within the cell to control its growth. Monoclonal antibodies are proteins designed to attach to specific targets on the membranes of cancer cells. These antibodies tag the cells, making it easier for the immune system to recognize the cells as ones that need to be destroyed.

Reference:
https://www.cancer.gov/about-cancer/treatment/types/targeted-therapies

Slide 9: This slide talks about the various side effects of targeted therapy. The type and degree of side effects depend on the method of therapy used. The most common side effects include

gastrointestinal issues, like diarrhea, and liver problems. Less common side effects include blood clotting problems, high blood pressure, fatigue, and loss of hair color.

Reference:
https://www.cancer.gov/about-cancer/treatment/types/targeted-therapies

Slide 10: This slide introduces hormone therapy as a cancer treatment. As mentioned in lesson 2, high estrogen and progesterone levels can be a breast cancer risk factor for women. However, other hormones are also to slow or stop the growth of cancer. Hormone therapy is only used to treat patients with prostate and breast cancer because these cancers often feed off hormones produced in the body.

Reference:
https://www.cancer.gov/about-cancer/treatment/hormone-therapy

Slide 11: This slide talks about the two main types of hormone therapies. The first type blocks the body's ability to produce hormones, and the second interferes with how hormones behave within the body. Cancer cells use hormones produced by the body to grow. Thus, interfering with or blocking the production of hormones inhibits the growth and division of cancer cells.

Reference:
https://www.cancer.gov/about-cancer/treatment/types/hormone-therapy

Slide 12: This slide mentions different side effects of hormone therapy, which are dependent on the type of cancer that the patient has. Prostate cancer side effects may include hot flashes, weakened bones, nausea, and enlarged breasts. Breast cancer side effects may include vaginal dryness, hot flashes, changes in menstrual cycle, and mood changes. All of these side effects are produced by the rapid changes in the body's hormone levels.

Reference:
https://www.cancer.gov/about-cancer/treatment/types/hormone-therapy

Slide 13: This slide introduces precision medicine as a method of selecting the proper cancer treatment. Prevision medicine involves the doctor having a deeper understanding of the genetics behind the tumor. Each tumor causes a genetic change within the body that allows it to grow and metastasize. All patients with one particular tumor type will receive a certain treatment type, while all patients with a different type of tumor will receive a different treatment. It's important to note that this treatment only works if the tumor has a genetic change that can be properly targeted by an existing drug. If the change cannot be targeted, then it cannot be treated using precision medicine.

Reference:
https://www.cancer.gov/about-cancer/treatment/types/precision-medicine

Slide 14: This slide discusses how doctors using precision medicine are able to determine a genetic change. A genetic change can only be determined via genetic sequencing, which can only be done after a part of the tumor has been removed via a biopsy. Genetic sequencing is a complex technique. You and your students can learn more here: https://www.genome.gov/about-genomics/fact-sheets/DNA-Sequencing-Fact-Sheet

Reference:
https://www.cancer.gov/about-cancer/treatment/types/precision-medicine

Slide 15: This slide discusses how differences in patients can result in different treatment regimens. Not all treatments will work for every patient; some treatment that kill cancerous cells

in one patient may have no effect on another. This, like many other things in science, can be attributed, in part, to genetics. Everyone's DNA is different, which can interfere with how the treatment interacts with their body. Some patient's body may reject certain drugs entirely, while others may be allergic to a certain drug type. Lastly, cancer and tumor type play a very important role in which type of cancer treatment. Ultimately, part of being an oncologist is developing a care plan that will work for the patient's physical and emotional needs.

Reference:
https://www.cancer.gov/about-cancer/treatment/types/precision-medicine

Slide 16: This slide introduces combination therapy as a part of a cancer treatment plan. Combination therapy is when a doctor combines two or more drug or treatment types to better fight the cancer. This could mean that surgery is coupled with radiation or that a stem cell transplant is combined with chemotherapy. It could also mean that multiple different drugs are used for targeted therapy. It is extremely common for combination therapy to be incorporated into a cancer treatment plan.

Reference:
https://pubmed.ncbi.nlm.nih.gov/28410237/

Slide 17: This slide lists a few of the reasons why doctors choose to recommend combination therapy. There are three main advantages. First, combination therapy increases the likelihood that the cancer will be eliminated entirely. If one treatment type does not cause the cancer to recede, another might. Second, combination therapy prevents the development of drug resistance. Some tumors may develop a mutation that allows them to become resistant to the one type of drug. However, it is far less likely that a tumor will develop two random mutations that makes it resistant to two drugs. Lastly, combination therapy reduces the length of treatment. Instead of testing one treatment and waiting to see if the cancer recedes, combination therapy saves the patient precious time by trying different treatments at one time.

Reference:
https://www.sciencedirect.com/topics/medicine-and-dentistry/combination-therapy

Slide 18: This slide lists a few reasons why doctors may avoid recommending combination therapy. First, there is always a risk when combining different treatment types. It is unclear how the patient's body will react to two competing treatments. There could be severe, adverse side effects. Additionally, it is unclear how two different drugs will interact with one another. To diminish this unknown, extensive research is done on the drugs that are used in combination therapy before they are combined. However, drugs also must go through a clinical trial phase, in which it may be unclear how they will interact with one another.

Reference:
https://www.sciencedirect.com/topics/medicine-and-dentistry/combination-therapy

Slide 19: This slide summarizes what has already been covered in the presentation. There is a lot of material in this lesson! Go over this slide carefully to remind students of what they have learned before transitioning into the discussion period.

C. Discussion Questions:

These questions are designed to help students think more critically about the information presented in the PowerPoint. Time permitting, we recommend having them discuss in pairs or small groups before beginning a large group discussion, but you could also go straight to the

large group discussion if necessary. Additional information is provided below each question for you to tell students after the discussion.

1.) Why do different patients require different treatments?
    a. Each patient is very different. Genetics, cancer type, and cancer size can all be different for patients.
2.) How do doctors decide which treatment regimen is best for their patient?
    a. There are a lot of factors to consider when deciding on a treatment regimen. All of the factors mentioned in the previous question are important things to consider. Other important considerations include preexisting health conditions, cost of care, emotional well-being, and whether or not the patient has a strong support system at home.
3.) Is cancer care a single-person job or a team effort? Explain all those involved in cancer treatment both directly and indirectly.
    a. Cancer care is very much a team effort! Here are just a few of the people that may be involved: doctors, pharmacists, nurses, dieticians, spouses, parents, children, friends, physical therapists, phycologists, social workers, and many, many more. Each one has a specific role. Ask your students to brainstorm how some of these people may be involved!

V. Evaluation

Teacher evaluation

Please complete the following evaluation after you have taught the lesson.

- What were the strengths of the lesson?
- What worked well?
- What were problem areas?
- How could you improve the lesson?
- What could you do differently if you were to teach it again?
- What is an alternate way to present the same material?
- Do you have any other comments regarding your experience teaching the lesson?

Student evaluation

1.) Which of these is NOT a cancer treatment?
    a. Chemotherapy
    b. Precision medicine
    c. Targeted therapy
    d. Surgery
    e. All of these are cancer treatments
2.) What is a stem cell?
    a. A cell that is completely differentiated
    b. A cell that is fully developed
    c. A cell that is young and undifferentiated
    d. A cell that is undergoing mitosis
3.) Which of these is a disadvantage of combination therapy?
    a. It speeds up the cancer treatment process
    b. It prevents the development of drug resistance
    c. It increases the likelihood that the cancer will be eliminated

   d. *It is unclear how the different drugs may interact with one another and could lead to adverse side effects.*

4.) Which of these is NOT a type of stem cell transplant?
   a. Autologous
   b. *Transgenic*
   c. Syngeneic
   d. Allogeneic

5.) What instrument does a minimally invasive laparoscopic surgery use?
   a. *Camera*
   b. Hammer
   c. Screw
   d. Retractor

6.) Radiation therapy…
   a. Is not a type of cancer treatment
   b. Is a type of cancer treatment
   c. Uses high doses of radiation to kill cancer cells and shrink tumors
   d. *Both b and c are correct*

7.) Hormone therapy is only used for breast cancer, prostate cancer, and liver cancer patients.
   a. True
   b. *False*

8.) How does targeted therapy work?
   a. It assists the immune system in destroying cancer cells
   b. It stops cancer cells from growing by halting division
   c. It directly delivers substances that are able to kill the cancer cells
   d. Only b and c are correct
   e. *A, b, and C are all correct*

9.) What is graft-versus-host disease?
   a. Where the stem cell donor cells enter into the patient's body and properly integrate themselves with the other cells
   b. *Where stem cell donor cells recognize the patient's cells as foreign and begin to attack them*
   c. Where the stem cell donor cells do not help the body fight cancer at all
   d. Where the stem cell donor cells help the body fight cancer

10.) How does a doctor determine a genetic change within a tumor?
   a. They complete a biopsy and simply look at the tumor under a microscope
   b. They complete a biopsy and do not look at the tumor under a microscope
   c. They do not complete a biopsy at all
   d. *They complete a biopsy and run genetic sequencing on the tumor*

Student Evaluation Answer Key

1) E
2) C
3) D
4) B
5) A
6) D
7) B
8) E
9) B

10) D
