# Supplementary material for: Cancer Curriculum for Appalachian Kentucky Middle and High Schools
Source: J Appalach Health. 2021 Jan 24;3(1):43–55. doi: 10.13023/jah.0301.05 (PMC8830599; doi:10.13023/jah.0301.05)
Supplement: Supplementary file 12 [file Appendix13-3.1.5Hudson.pdf]

LESSON PLAN OUTLINE

Lesson: Cancer in the Commonwealth - Lesson 3, Short: Treatment

Grade Level: Kentucky middle and high school students

Length: 30-45 minutes

I. Objective:

To encourage students to think critically about cancer and its treatments within the context of Kentucky and their community.

II. Standards

Middle School Next Generation Science Standards (Engineering Design):

MS-ETS1-1: Define the criteria and constraints of a design problem with sufficient precision to ensure a successful solution, taking into account relevant scientific principles and potential impacts on people and the natural environment that may limit possible solutions.

SEP: Asking Questions and Defining Problems

MS-ETS1-2: Evaluate competing design solutions using a systematic process to determine how well they meet the criteria and constraints of the problem.

SEP: Constructing Explanations and Designing Solutions

High School Next Generation Science Standards (Engineering Design):

HS-ETS1-1: Analyze a major global challenge to specify qualitative and quantitative criteria and constraints for solutions that account for societal needs and wants.

SEP: Asking Questions and Defining Problems

HS-ETS1-2: Design a solution to a complex real-world problem by breaking it down into smaller, more manageable problems that can be solved through engineering.

SEP: Constructing Explanations and Designing Solutions

HS-ETS1-3: Evaluate a solution to a complex real-world problem based on prioritized criteria and trade-offs that account for a range of constraints, including cost, safety, reliability, and aesthetics, as well as possible social, cultural, and environmental impacts.

SEP: Using Mathematics and Computational Thinking

Kentucky Academic Standards for Health Education:

Standard 1: Students will comprehend content related to health promotion and disease prevention to enhance health.

Standard 2: Analyze the influence of family, peers, culture, media, technology and other factors on health behaviors.

Standard 3: Access valid information, products and services to enhance health.

Standard 4: Use interpersonal communication skills to enhance health and avoid or reduce health risks.

Standard 5: Use decision-making skills to enhance health.

Standard 6: Use goal-setting skills to enhance health.

Standard 7: Practice health-enhancing behaviors and avoid or reduce health risks.

Standard 8: Advocate for personal, family and community health.

III. Preparation

Purpose:

To teach Kentucky middle and high school students about how different cancer treatments work and which are the most commonly used.

Materials:

Cancer in the Commonwealth, Lesson 3: Treatment PowerPoint
Computers/telephones for students to complete individual research
White board/SmartBoard

IV. Procedure

A. Initial Engagement

The first thing you need to do is remind students of what they learned in lesson 1 and 2. Share about what cancer is, its disparities, and its risk factors in the United States. Emphasize Kentucky's high cancer rates and modifiable behaviors in order to create a personal connection and remind them why this topic is so important to Appalachia Kentucky in particular. This can be completed on the title slide of the PowerPoint.

B. Pretest Survey

For this curriculum, students must complete both a pre and posttest questionnaire to gauge their knowledge before and after this lesson. Please have students complete the questionnaire before the lesson and then again after you have delivered the lesson. The questions are listed at the end of this lesson plan under “evaluation.” The pre and posttests are identical to one another.

C. Body of the Lesson/Input:

This section will go through the PowerPoint slide by slide to provide additional information and sources for each point.

Slide 2 and 3: These slides encourage students to learn about different cancer treatments in small groups. Assign each group a different type of cancer treatment and ask them to research their treatment in-depth. Things to consider when researching are how the treatment works, why it is useful for patients, and what side effects may occur. Pair them with another group and have each group explain to the other how their treatment works. This will give them a good introduction to two types of cancer treatment. It will deepen their understanding of their assigned cancer treatment by teaching it to another group.

Slide 4: This slide reviews a concept from lesson 1: how cancer is diagnosed. Cancer is diagnosed using a scan followed by a biopsy. A biopsy is a surgical procedure where a doctor removes a small sample of cells in the tumor and sends it to a lab for closer analysis. A researcher will then look at the sample under a microscope to see if it is cancerous. Use the diagram to remind students of the difference between normal and cancerous cells. Treatment can only begin after the cancer has been properly diagnosed.

Reference: https://www.cancer.org/treatment/understanding-your-diagnoses/tests/testing-biopsy-and-cytology-specimens-for-cancer/how-is-cancer-diagnosed.html

Slide 5: This slide discusses the concept of early detection. Early detection has been achieved when the cancer is found when it is small and hasn’t metastasized yet. When the cancer is this early in its development, there are rarely signs/symptoms that would lead a person to receive an out-of-the-ordinary screening. Typically, early detection is achieved through a regularly scheduled cancer screening, such as a colonoscopy or mammogram.

Reference:
https://www.cancer.org/healthy/find-cancer-early.html

Slide 6: The slide discusses the importance of early detection. A cancer that is small and has not spread is much easier to treat and control than one that is large and has metastasized. Cancers that have metastasized often require intensive treatment and still don’t always recede. Early detection greatly improves a patient’s chances of surviving.

References:
https://www.cancer.gov/about-cancer/screening

Slide 7: This slide discusses what may occur in regions where early detection is not widespread. Early detection is not as common in rural areas as it is in urban areas. Similarly, patients living in rural areas have a greater chance of being diagnosed with later stage cancers. This can be contributed, in part, by a lack of access to healthcare services. Specifically, Appalachia Kentucky has very limited access to cancer care facilities, with the nearest facility being located in Lexington at the Markey Cancer Center. This can greatly affect their ability to obtain screenings and their treatment regimen following diagnosis.

Reference:
https://pubmed.ncbi.nlm.nih.gov/19780916/

Slide 8: This slide discusses barriers to early detection in Appalachia Kentucky. As your students may know, the nearest cancer care facility in rural areas can be 2-3 hours away on hazardous roads. Additionally, adults may not be able to take off work in order to travel to receive care for fear of not being paid or losing their job. They may need the money to pay for food, clothes, or other necessities. As a result, regular doctor visits may be pushed back in favor of more immediate, pressing matters.

Reference:
https://pubmed.ncbi.nlm.nih.gov/19780916/

Slide 9: This slide lists the 8 types of cancer treatments that will be discussed in the following slides. There is no need to spend much time on this slide, as each treatment will be covered in-depth in the coming slides.

Reference:
https://www.cancer.org/treatment

Slide 10: This slide introduces one of the most common and well-known types of cancer treatment: chemotherapy. When most students think of cancer treatment, they likely think of chemotherapy (chemo). They have likely seen someone they know undergo chemotherapy, resulting in hair loss and fatigue. Chemotherapy uses powerful chemicals to kill cancer cells. The chemicals (either just 1 or a cocktail of different chemicals) are infused through an IV. If multiple chemicals are used, they must be given in a specific order to properly disable the cancer cells. The type and quantity of drugs are decided by the size and stage of the tumor and the patient's age and overall health. If the cancer is smaller and has not metastasized, the chemicals will not be as powerful because they are not necessary to kill the cancer cells. If the patient is older and not healthy, the drugs may not be as powerful for fear of the chemotherapy becoming fatal.

Reference: https://www.cancer.org/treatment/treatments-and-side-effects/treatment-types/chemotherapy/how-is-chemotherapy-used-to-treat-cancer.html

Slide 11: This slide discusses radiation therapy as a cancer treatment. Radiation therapy uses high doses of radiation to kill cancer cells and shrink tumors. Interestingly, radiation is also a cancer risk factor because it causes mutations in cells that can lead to cancerous development. Radiation also does damage to the DNA of cancer cells, effectively killing them after continuous treatment. Even after treatment concludes, cells continue to die.

Reference:
https://www.cancer.gov/about-cancer/treatment/types/radiation-therapy

Slide 12: This slide introduces surgery as a cancer treatment. Many students are familiar with the idea of surgery, so it is important to frame it within the context of cancer. Surgery as a cancer treatment occurs when surgeons remove part or all of the tumor in one area of the body. Ideally, the surgeon would remove all the tumor, but in some situations, removing all of the tumor may lead to life-threatening damage to other organs. In these situations, the surgeon will only remove part of the tumor, which can help the treatments proceed faster and smoother. It can also help relieve pain and pressure in the area of the tumor, leading to increased comfort for the patient.

Reference:
https://www.cancer.gov/about-cancer/treatment/types/surgery

Slide 13: This slide introduces immunotherapy as a cancer treatment. This treatment assists the immune system in fighting the cancer. The video linked in the PowerPoint will better explain how this treatment works, as visuals are essential in understanding immunotherapy. Immunotherapy can be administered through an IV, a pill, a rub-on ointment, or inserted directly into the bladder for bladder cancer patients.

Reference:
https://www.cancer.gov/about-cancer/treatment/types/immunotherapy

Slide 14: This slide introduces stem cell transplants as a type of cancer treatment. This treatment is often used in conjunction with chemotherapy or radiation. Stem cells, as some of your students may be familiar with, are young cells that have yet to differentiate. This means they are able to develop into nearly any cell type. These stems cells help restore the stem cells of cancer patients who have previously undergone chemotherapy. Because chemotherapy has killed many cells of the patients, adding stem cells back into the body helps restore white blood cells, red blood cells, and platelets. This will build the patient’s immune system and help their recovery move faster.

Reference:
https://www.cancer.gov/about-cancer/treatment/types/stem-cell-transplant

Slide 15: This slide introduces targeted therapy as a type of cancer treatment. Targeted therapy targets proteins produced in certain areas of the body to control the growth, division, and spread of cancer cells. This helps treat cancer in 4 ways: 1) It assists the immune system in destroying cancer cells; 2) It stops cancer cells from growing by halting division; 3) It directly delivers substances that are able to kill the cancer cells; and 4) It starves cancer cells of the hormones they need to grow, stopping the spread of the disease.

Reference:
https://www.cancer.gov/about-cancer/treatment/types/targeted-therapies

Slide 16: This slide introduces hormone therapy as a cancer treatment. As mentioned in lesson 2, high estrogen and progesterone levels can be a breast cancer risk factor for women. However, other hormones are also to slow or stop the growth of cancer. Hormone therapy is only used to treat patients with prostate and breast cancer because these cancers often feed off hormones produced in the body.

Reference:
https://www.cancer.gov/about-cancer/treatment/hormone-therapy

Slide 17: This slide introduces precision medicine as a method of selecting the proper cancer treatment. Prevision medicine involves the doctor having a deeper understanding of the genetics behind the tumor. Each tumor causes a genetic change within the body that allows it to grow and metastasize. All patients with one particular tumor type will receive a certain treatment type, while all patients with a different type of tumor will receive a different treatment. It's important to note that this treatment only works if the tumor has a genetic change that can be properly targeted by an existing drug. If the change cannot be targeted, then it cannot be treated using precision medicine. A genetic change can only be determined via genetic sequencing, which can only be done after a part of the tumor has been removed via a biopsy. Genetic sequencing is a complex technique. You and your students can learn more here: https://www.genome.gov/about-genomics/fact-sheets/DNA-Sequencing-Fact-Sheet

Reference:
https://www.cancer.gov/about-cancer/treatment/types/precision-medicine

Slide 18: This slide discusses how differences in patients can result in different treatment regimens. Not all treatments will work for every patient; some treatments that kill cancerous cells in one patient may have no effect on another. This, like many other things in science, can be attributed, in part, to genetics. Everyone's DNA is different, which can interfere with how the treatment interacts with their body. Some patient's bodies may reject certain drugs entirely, while others may be allergic to a certain drug type. Lastly, cancer and tumor type play a very important role in which type of cancer treatment. Ultimately, part of being an oncologist is developing a care plan that will work for the patient's physical and emotional needs.

Reference:
https://www.cancer.gov/about-cancer/treatment/types/precision-medicine

Slide 19: This slide introduces combination therapy as a part of a cancer treatment plan. Combination therapy is when a doctor combines two or more drug or treatment types to better fight the cancer. This could mean that surgery is coupled with radiation or that a stem cell transplant is combined with chemotherapy. It could also mean that multiple different drugs are used for targeted therapy. It is extremely common for combination therapy to be incorporated into a cancer treatment plan.

Reference:
https://pubmed.ncbi.nlm.nih.gov/28410237/

Slide 20: This slide lists the advantages and disadvantages of combination therapy. There are three main advantages. First, combination therapy increases the likelihood that the cancer will be eliminated entirely. If one treatment type does not cause the cancer to recede, another might. Second, combination therapy prevents the development of drug resistance. Some tumors may develop a mutation that allows them to become resistant to the one type of drug. However, it is far less likely that a tumor will develop two random mutations that makes it resistant to two drugs. Lastly, combination therapy reduces the length of treatment. Instead of testing one treatment and waiting to see if the cancer recedes, combination therapy saves the patient precious time by trying different treatments at one time.

There are two main disadvantages. First, there is always a risk when combining different treatment types. It is unclear how the patient's body will react to two competing treatments. There could be severe, adverse side effects. Additionally, it is unclear how two different drugs

will interact with one another. To diminish this unknown, extensive research is done on the drugs that are used in combination therapy before they are combined. However, drugs also must go through a clinical trial phase, in which it may be unclear how they will interact with one another.

Reference:
https://www.sciencedirect.com/topics/medicine-and-dentistry/combination-therapy

Slide 21: This slide summarizes what has already been covered in the presentation. There is a lot of material in this lesson! Go over this slide carefully to remind students of what they have learned before transitioning into the discussion period.

D. Discussion Questions:

These questions are designed to help students think more critically about the information presented in the PowerPoint. Time permitting, we recommend having them discuss in pairs or small groups before beginning a large group discussion, but you could also go straight to the large group discussion if necessary. Additional information is provided below each question for you to tell students after the discussion.

1.) Why do different patients require different treatments?
    a. Each patient is very different. Genetics, cancer type, and cancer size can all be different for patients.
2.) How do doctors decide which treatment regimen is best for their patient?
    a. There are a lot of factors to consider when deciding on a treatment regimen. All of the factors mentioned in the previous question are important things to consider. Other important considerations include preexisting health conditions, cost of care, emotional well-being, and whether or not the patient has a strong support system at home.
3.) Is cancer care a single-person job or a team effort? Explain all those involved in cancer treatment both directly and indirectly.
    a. Cancer care is very much a team effort! Here are just a few of the people that may be involved: doctors, pharmacists, nurses, dieticians, spouses, parents, children, friends, physical therapists, phycologists, social workers, and many, many more. Each one has a specific role. Ask your students to brainstorm how some of these people may be involved!

V. Evaluation

Teacher evaluation
Please complete the following evaluation after you have taught the lesson.

- What were the strengths of the lesson?
- What worked well?
- What were problem areas?
- How could you improve the lesson?
- What could you do differently if you were to teach it again?
- What is an alternate way to present the same material?

- Do you have any other comments regarding your experience teaching the lesson?

Student evaluation

1.) Which of these is NOT a cancer treatment?
    a. Chemotherapy
    b. Precision medicine
    c. Targeted therapy
    d. Surgery
    e. All of these are cancer treatments
2.) What is a stem cell?
    a. A cell that is completely differentiated
    b. A cell that is fully developed
    c. A cell that is young and undifferentiated
    d. A cell that is undergoing mitosis
3.) Which of these is a disadvantage of combination therapy?
    a. It speeds up the cancer treatment process
    b. It prevents the development of drug resistance
    c. It increases the likelihood that the cancer will be eliminated
    d. It is unclear how the different drugs may interact with one another and could lead to adverse side effects.
4.) Which of these is one of the most common types of cancer treatments?
    a. Chemotherapy
    b. Hormone therapy
    c. Targeted therapy
    d. Stem cell transplants
5.) Combination therapy means combining at least three or more types of treatments or drugs.
    a. True
    b. False
6.) Radiation therapy…
    a. Is not a type of cancer treatment
    b. Is a type of cancer treatment
    c. Uses high doses of radiation to kill cancer cells and shrink tumors
    d. Both b and c are correct
7.) Hormone therapy is only used for breast cancer, prostate cancer, and liver cancer patients.
    a. True
    b. False
8.) How does targeted therapy work?
    a. It assists the immune system in destroying cancer cells
    b. It stops cancer cells from growing by halting division
    c. It directly delivers substances that are able to kill the cancer cells
    d. Only b and c are correct
    e. A, b, and C are all correct
9.) Why do surgeons sometimes decide to remove only some of a cancerous tumor as opposed to the whole tumor?

a. They never make this decision. They always remove the entire tumor
b. It is too expensive for the surgeon to remove the entire tumor
c. Removing the entire tumor would cause the patient additional pain
d. Removing the entire tumor could cause damage to another part of the body.

10.) In which scenario would precision medicine <u>not</u> work?
a. If the tumor is too large to be targeted by a drug
b. If the tumor is too small to be targeted by a drug
c. If the tumor has a genetic change that cannot be targeted by a drug
d. If the tumor has metastasized already

Student Evaluation Answer Key

1) E
2) C
3) D
4) A
5) B
6) D
7) B
8) E
9) D
10) C
