# Supplementary material for: Cancer Curriculum for Appalachian Kentucky Middle and High Schools
Source: J Appalach Health. 2021 Jan 24;3(1):43–55. doi: 10.13023/jah.0301.05 (PMC8830599; doi:10.13023/jah.0301.05)
Supplement: Supplementary file 13 [file Appendix14-3.1.5Hudson.pdf]

Appendix 14. Cancer curricula alignment with national and Kentucky science and health education academic standards.

Lesson	Next Generation Science Standards	Kentucky Academic Standards for Health Education
**Lesson 1: Cancer Basics**	• **MS-LS1-1.** Conduct an investigation to provide evidence that living things are made of cells; either one cell or many different numbers and types of cells. (Cross-Cutting Concepts (CCC): Scale, Proportion, and Quantity; Connections to Nature of Science: Interdependence of Science, Engineering, and Technology).<br>**Science and Engineering Practice (SEP):** Planning and carrying out investigations.<br>• **MS-LS1-5.** Construct a scientific explanation based on evidence for how environmental and genetic factors influence the growth of organisms. (CCC: Cause and Effect)<br>**SEP:** Constructing explanations and designing solutions.<br>• **HS-LS1-4.** Use a model to illustrate the role of cellular division (mitosis) and differentiation in producing and maintaining complex organisms. (CCC: Systems and System Models)<br>**SEP:** Developing and Using Models<br>• **HS-LS3-2.** Make and defend a claim based on evidence that inheritable genetic variations may result from: (1) new genetic combinations through meiosis, (2) viable errors occurring during replication, and/or (3) mutations caused by environmental factors. (CCC: Cause and Effect)<br>**SEP:** Engaging in Argument from Evidence<br>• **HS-LS3-3.** Apply concepts of statistics and probability to explain the variation and distribution of expressed traits in a population. (CCC: Scale, proportion, and	• **Standard 1.** Comprehend content related to health promotion and disease prevention to enhance health.<br>• **Standard 2.** Analyze the influence of family, peers, culture, media, technology and other factors on health behaviors.<br>• **Standard 3**. Access valid information, products and services to enhance health.<br>• **Standard 4.** Use interpersonal communication skills to enhance health and avoid or reduce health risks.<br>• **Standard 5.** Use decision-making to enhance health<br>• **Standard 6.** Use goal-setting skills to enhance health.<br>• **Standard 7.** Practice health-enhancing behaviors and avoid or reduce health risks.<br>• **Standard 8.** Advocate for personal, family and community health.

	quantity; Connections to Nature of Science: Science is a Human Endeavor)<br><br>**SEP:** Analyze Data Using Tools, Technologies, and/or Models (e.g., computational, mathematical) in order to make valid and reliable scientific claims or determine an optimal design solution.	
**Lesson 2: Risk factors and modifiable behaviors**	• **MS-LS3-1.** Develop and use a model to describe why structural changes to genes (mutations) located on chromosomes may affect protein and may result in harmful, beneficial, or neutral effects to the structure and function of an organism (CCC: Structure and Function).<br><br>**SEP:** Developing and Using Models<br><br>• **HS-LS1-1.** Construct an explanation based on evidence for how the structure of DNA determines the structure of proteins, which carry out the essential functions of life through systems of specialized cells (CCC: Structure and Function).<br><br>**SEP:** Constructing explanations and Designing Solutions<br><br>• **HS-LS3-1.** Ask questions to clarify relationships about the role of DNA and chromosomes in coding the instructions for characteristic traits passed from parents to offspring (CCC: Cause and Effect)<br><br>**SEP:** Asking Questions and Defining Problems<br><br>• **HS-LS3-2.** Make and defend a claim based on evidence that inheritable genetic variation may result from (1) new genetic combinations through mitosis, (2) viable errors occurring during replication, and/or (3) mutations caused by environmental factors (CCC: Cause and Effect).<br><br>**SEP:** Engaging in Argumentation from Evidence	• **Standard 1.** Comprehend content related to health promotion and disease prevention to enhance health.<br><br>• **Standard 2.** Analyze the influence of family, peers, culture, media, technology and other factors on health behaviors.<br><br>• **Standard 3.** Access valid information, products and services to enhance health.<br><br>• **Standard 4.** Use interpersonal communication skills to enhance health and avoid or reduce health risks.<br><br>• **Standard 5.** Use decision-making skills to enhance health.<br><br>• **Standard 6.** Use goal-setting skills to enhance health.<br><br>• **Standard 7.** Practice health-enhancing behaviors and avoid or reduce health risks.<br><br>• **Standard 8.** Advocate for personal, family and community health.

**Lesson 3: Treatment**	• **MS-ETS1-1.** Define the criteria and constraints of a design problem with sufficient precision to ensure a successful solution, taking into account relevant scientific principles and potential impacts on people and the natural environment that may limit possible solutions.<br>**SEP:** Asking Questions and Defining Problems<br>• **MS-ETS1-2.** Evaluate competing design solutions using a systematic process to determine how well they meet the criteria and constraints of the problem.<br>**SEP:** Constructing Explanations and Designing Solutions<br>• **HS-ETS1-1.** Analyze a major global challenge to specify qualitative and quantitative criteria and constraints for solutions that account for societal needs and wants.<br>**SEP:** Asking Questions and Defining Problems<br>• **HS-ETS1-2.** Design a solution to a complex real-world problem by breaking it down into smaller, more manageable problems that can be solved through engineering.<br>**SEP:** Constructing Explanations and Designing Solutions<br>• **HS-ETS1-3.** Evaluate a solution to a complex real-world problem based on prioritized criteria and trade-offs that account for a range of constraints, including cost, safety, reliability, and aesthetics, as well as possible social, cultural, and environmental impacts.<br>**SEP:** Using Mathematics and Computational Thinking	• **Standard 1.** Students will comprehend content related to health promotion and disease prevention to enhance health.<br>• **Standard 2.** Analyze the influence of family, peers, culture, media, technology and other factors on health behaviors.<br>• **Standard 3.** Access valid information, products and services to enhance health.<br>• **Standard 4.** Use interpersonal communication skills to enhance health and avoid or reduce health risks.<br>• **Standard 5.** Use decision-making skills to enhance health.<br>• **Standard 6.** Use goal-setting skills to enhance health.<br>• **Standard 7.** Practice health-enhancing behaviors and avoid or reduce health risks.<br>• **Standard 8.** Advocate for personal, family and community health.
