# Supplementary material for: Cancer Curriculum for Appalachian Kentucky Middle and High Schools
Source: J Appalach Health. 2021 Jan 24;3(1):43–55. doi: 10.13023/jah.0301.05 (PMC8830599; doi:10.13023/jah.0301.05)
Supplement: Supplementary file 14 [file Table2-3.1.5Hudson.pdf]

Table 2. Current incorporation of cancer education in school curriculum if any

Variable	Course Topic	Teacher Response	Teacher Grade Level
Do you or your school do anything to incorporate cancer education into the curriculum?	Medicine/ Health	• One unit in my medical interventions course focuses on cancer types, treatment, and prevention.	11th
		• In health classes.	10th
		• Personally, when I taught anatomy, I used real-life examples of cancer based upon the subject that was being covered. Now, I only teach chemistry	11th
		• Project Lead The Way Medical Interventions. This is a biomedical course for Juniors.	10th
		• Health club and projects in health class and health sciences classes	12th
		• We discuss cancer briefly but focus more on family medical history, risk factors, and causes of cancer in general. i.e., melanoma caused by damaging sun, lung cancer caused by toxins in lungs; etc.	9th
	Biology	• Some is taught in biology.	10th
		• We teach about mitosis and briefly mention cancer.	10th
		• We use it as an example of the cell cycle/mitosis "gone wrong" as well as incorporate it into genetics units with expression of melanin (skin cancer).	10th
		• My students learned briefly about cancer when we were learning about mitosis.	10th
		• We discuss cell mutations and how they affect growth and function.	11th
		• Although I don't teach biology or biomedical technology, both of those classes do delve into cancer.	11th
		• Incorporate next-generation sequencing.	9th
		• I mention it when we cover genetics but not in much detail.	8th
		• Biology includes teaching about cancer and causes.	9th
		• Information on mutagen and carcinogens is discussed. In biology, a chapter on cancer/ cell division/ genetics is covered.	10th
		• I teach several different courses in biology, chemistry, and biomedical sciences. We talk about the cell cycle and how cancer is a result of loss of control of cell cycle. In some of my classes, we talk about HeLa cells and how that line has	10th

		been used to do a lot of cancer research and the ethical issues that have arisen from that.	
		• Our biomedical classes and biology classes study cancer mechanisms.	9th
	Chemistry	• During the nuclear chemistry unit, we talk in-depth about treatment modalities that utilize nuclear chemistry (gamma knife, linear accelerator, radioactive iodine for thyroid tumors, brachytherapy). Students research these topics and present them to their peers.	11th
		• In my course, we study the electromagnetic spectrum and atomic structure. We discuss radiation and cancer then. We also study basic nuclear chemistry - and again reinforce radiation/cancer links. We the atmosphere - and by that time student draw their own conclusions about depletion of ozone's effects on humans (we use maps of ozone and melanoma incidence). We also study human impact and at various points incidence of cancer.	9th
		• I touch on skin cancer briefly when talking about ozone and chlorofluorocarbons and electromagnetic spectrum.	11th
	No existing cancer curriculum/ subject not specified	• Not that I am aware of	9th
		• I teach the standards. I only have my students every other day so content is all I can get to complete.	6th
		• I touch on it some in my classes, but it is not taught school wide.	11th
