# Supplementary material for: Cancer Curriculum for Appalachian Kentucky Middle and High Schools
Source: J Appalach Health. 2021 Jan 24;3(1):43–55. doi: 10.13023/jah.0301.05 (PMC8830599; doi:10.13023/jah.0301.05)
Supplement: Supplementary file 15 [file Table3-3.1.5Hudson.pdf]

Table 3. Belief in the benefit of cancer education in schools

Variable	Topic	Teacher Response	Teacher Grade Level
Do you believe cancer education in schools would be beneficial?	Prevention	• Cancer can be preventable in not all but a lot of ways, so it is necessary to teach.	10th
		• I think it gives students a great understanding of how a very common disease works and it is beneficial to them knowing preventions and maybe future studies.	11th
		• Students need to know about prevention and treatment for cancer. The more students know, the more empowered they are to make healthy lifestyle choices and potentially even help their family members who might be undergoing cancer treatment.	11th
		• Students need to be educated about cancer to encourage early detection.	9th
		• Students need to be informed of potential harm in order to avoid substances & behaviors that are harmful.	10th
		• If students are aware of the risk factors, causes, and the link to family history, it may prevent them from engaging in risky unhealthy behaviors. It is easier to change habits while young than older. They may take the information back to their homes and help change habits for their families.	9th
		• If students knew risk factors, screenings they should get etc.	11th
	Cancer Prevalence	• Cancer affects so many people in our community, and I believe it will help students understand a cancer diagnosis.	11th
		• It is beneficial because it is a disease that impacts all of them.	10th
		• Cancer is a disease that affects so many, including myself, and it would be beneficial for students to understand why and how it is happening.	10th

		• Many students, if not all students, are impacted by cancer at some point in their life.	9th
		• Sadly, cancer touches so many lives.	11th
		• Kentucky has high rates of cancer; students are familiar with it because of their families. They are ready to be more informed and make better choices when they have the opportunity, education, and means.	9th
		• Affects many families in the area.	9th
		• Cancer is something that touches most families. I think education about it is beneficial.	8th
		• Cancer affects everyone. While I teach about it from a scientific standpoint, I think it would be beneficial to address it from a public health standpoint. On the other hand, high school students feel almost invincible at this age, so I am not sure they would take it to heart.	10th
	General awareness/ Education	• Awareness is always important.	11th
		• There are a lot of misconceptions when it comes to cancer. Many students do not understand why there is not a cure all for cancer. Many students have relatives or know someone with cancer, and it is information that could help them with their families and their futures.	10th
		• I think students would benefit from knowing what external things might contribute to cancer.	6th
		• Health is not emphasized in curriculum.	12th
		• I think any real-world application engages the students more.	10th
		• It could also provide interest that they may want to explore further in the future.	10th
		• They should be informed on the different types.	10th
