# Supplementary material for: Cancer Curriculum for Appalachian Kentucky Middle and High Schools
Source: J Appalach Health. 2021 Jan 24;3(1):43–55. doi: 10.13023/jah.0301.05 (PMC8830599; doi:10.13023/jah.0301.05)
Supplement: Supplementary file 16 [file Table4-3.1.5Hudson.pdf]

Table 4. Feasibility of incorporating cancer education into school curriculum

Variable	Topic	Teacher Response	Teacher Grade Level
Do you believe incorporating cancer topics into the curriculum would be feasible within your classroom/ school?	Medicine/Health	• Maybe even place that into our health class as well.	10th
		• In science and health classes.	10th
		• I think that certain subjects (such as science, health, and agriculture) can easily incorporate cancer topics into the curriculum. Because cancer is ubiquitous, it is our duty to inform students of risk factors and prevention strategies. In addition, students value what they learn in school more if they see relevance to real life. Sadly, cancer is an incredibly relevant topic.	11th
		• It is already a part of many science topics, so studies involving data analysis, life-style choices, etc. would be considered part of the curriculum.	9th
		• I personally can add cancer topics in several areas of my class. I set my class up by body systems: anatomy, function, ailments, and treatments. Adding cancer to that would be no issue at all.	9th
	Biology	• It is feasible when teaching on genetics.	10th
		• When I taught biology, I used the NIH curriculum for cancer and the cell cycle. It can be included in chemistry because of radiation treatments as well.	11th
		• Yes, because we learn about the replication of cells!	10th
		• Potentially into biology class discussion of cell growth.	9th
		• Usually at the end of each semester, there is time to complete STEM activities and we could incorporate the cancer education during this time.	6th
		• Biology classes.	9th

		• I have room in my [biology] curriculum for a small unit.	8th
	Chemistry	• I could discuss this topic in biology or even chemistry terms.	11th
		• There are so many chemistry concepts to cover in the curriculum, I don't feel like I would have adequate time to incorporate a lot of cancer topics.	11th
	Dependent upon materials provided	• If we were supplied with material that prepared and understandable for high school students.	10th
		• If resources are provided and aligned with standards.	9th
	Already incorporate cancer curriculum	• I try to incorporate information while teaching mitosis to general biology students. I also have a curriculum from Project Lead The Way that has an entire unit on cancer to cover with my junior biomedical students.	10th
		• We already do this in biology classes - we could probably elaborate on it more.	10th
		• I already do :)	10th
		• I teach health science 9-12 grade, and I already touch on it some. It would be easy to incorporate it more.	11th
